# Dual Nickel Photocatalysis for *O*-Aryl Carbamate Synthesis from Carbon Dioxide

**DOI:** 10.1021/acs.joc.3c00023

**Published:** 2023-02-27

**Authors:** Aleksi Sahari, Jukka Puumi, Jere K. Mannisto, Timo Repo

**Affiliations:** Department of Chemistry, University of Helsinki, FI-00014 Helsinki, Finland

## Abstract

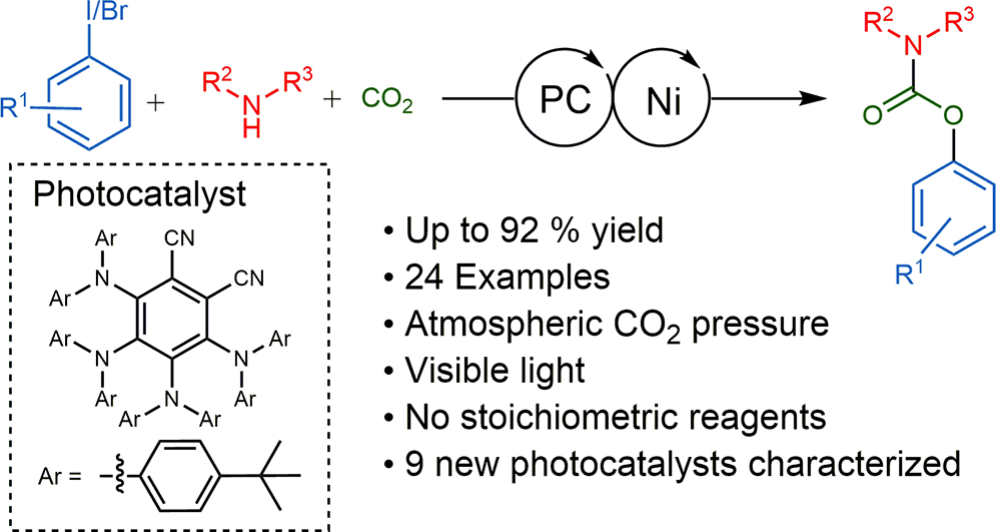

We report the use of dual nickel photocatalysis in the synthesis of *O*-aryl carbamates from aryl iodides or bromides, amines, and carbon dioxide. The reaction proceeded in visible light, at ambient carbon dioxide pressure, and without stoichiometric activating reagents. Mechanistic analysis is consistent with a Ni(I–III) cycle, where the active species is generated by the photocatalyst. The rate-limiting steps were the photocatalyst-mediated reduction of Ni(II) to Ni(I) and subsequent oxidative addition of the aryl halide. The physical properties of the photocatalyst were critical for promoting formation of *O*-aryl carbamates over various byproducts. Nine new phthalonitrile photocatalysts were synthesized, which exhibited properties that were vital to achieve high selectivity and activity.

## Introduction

**Scheme 1:**
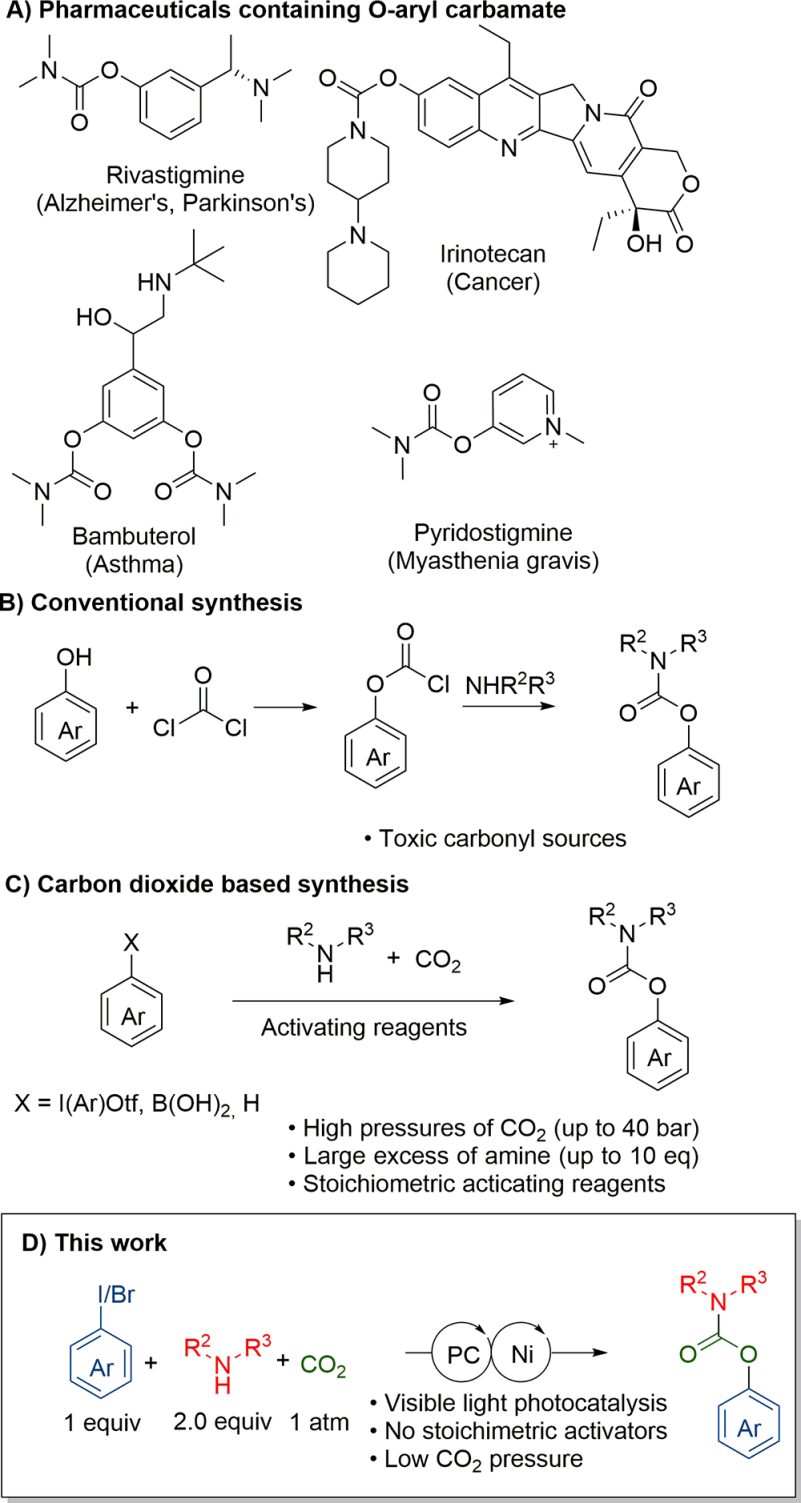
(A) Examples of High Value Compounds Containing an *O*-Aryl Carbamate Group;^7^ (B) Conventional *O*-Arylcarbamate Syntheses Use Phosgene Derivatives as a Carbonyl Sources;^2a,8^ (C) Previous Work on the *O*-Aryl Carbamate Synthesis Using Carbon Dioxide;^6^ (D) The Method Described in This Article

*O*-Aryl carbamates are found in pharmaceuticals and natural products (Scheme 1A).^1^ Traditionally, they are synthesized starting from amines, alcohols, and toxic phosgene or its derivatives (Scheme 1B).^2^ In such synthesis, phosgene sequentially reacted with the amine and the alcohol in either order. While the reaction worked well for the synthesis of a variety of carbamates, electron-poor or heteroaromatic phenols tended to react in poor yields.^2b,3^ Additionally, a reaction route without toxic reagents is desired. Carbon dioxide is a potential replacement for phosgene in carbamate formation. It is a nontoxic, renewable, and easily available C1 reagent, but its use is limited by its thermodynamic stability.^4^ Its use has already been well demonstrated in carbamate synthesis with aliphatic alcohols, where an oxygen-abstracting reagent was often used to drive the reaction.^4c,5^ However, *O*-aryl carbamates are exceedingly difficult to obtain from phenols due to the instability of the phenol–acyl bond.

An alternative approach is to directly couple the carbamate anion, formed from an amine reacting with carbon dioxide, with an aromatic group (Scheme 1C). Previously, this strategy was used with aryl iodium, aryl sulfonium, oxidative coupling of boronic esters, and C–H activation with directing group activated copper catalysis.^6^ These methods required large excesses of amine, high temperatures and pressures, or stoichiometric reagents. The employed high temperatures were especially problematic, as this disfavors carbamate formation, which must be compensated for with elevated pressures of CO_2_.

Recently, dual nickel photocatalysis has attracted attention in heteroatom–carbon coupling reactions of aryl (pseudo)halides with various nucleophiles, such as amines,^9^ alcohols,^10^ carboxylic acids,^11^ amides,^12^ and thiols.^13^ Photocatalysis has been typically conducted at moderate temperatures, which we reasoned would enable CO_2_-based synthesis of *O*-aryl carbamates at ambient pressure, while simultaneously providing sufficient driving force to couple the carbamate anion with aryl halides (Scheme 1D).^14^

## Results and Discussion

We used previous studies on dual nickel-photocatalyzed aryl halide heteroatom couplings as a starting point,^15^ and after initial optimization we managed to get up to a 78% yield, with 4-iodobenzotrifluoride, morpholine, and CO_2_ (Table 1, entry 1). The reactions required the nickel complex, photocatalyst, and visible light to proceed (entries 2–4). Compact fluorescent lamps (CFL) gave the product in higher selectivity than blue LEDs (entry 5). The 4,4′-di-*tert*-butyl-2,2′-bipyridine (dtbbpy) ligand was important for this reaction as was reported also in the previous studies (entry 6). Amine and TMG loading could be further reduced with a minor decrease in yield (entries 7 and 8). The used conditions significantly influenced the yield of carbamate **3** by changing the selectivity of competing side reactions (Table 1, entries 9–17). These were *N*-arylation **4**, phenol formation **5**, and protodehalogenation **6**. *N*-arylated products **4** are formed from direct amine coupling,^16^ which was likely caused by residual amounts of free amine from the amine–carbamate equilibrium. Stronger bases such as Cs_2_CO_3_, 1,8-diazabicyclo(5.4.0)undec-7-ene (DBU), or tetramethylguanidine (TMG) pushed this equilibrium toward the carbamate formation, thus giving higher selectivity compared to weaker amine bases (Table 1, entries 9–12, Supporting Information (SI) Table S5). TMG and DBU may also have an additional role as a reducing agent, even though their reduction potentials are usually slightly higher than secondary amines (DBU 1.26 V, TMG 1.24 V (SI Figure S23) vs secondary amines 0.9–1.1 V).^17^ Phenol **5** was mainly formed by side reactions with either residual water or oxygen as they both increase phenol formation from ca. 3% to ca. 10% (Table 1, entries 13–14, SI Table S13).^18^ Oxygen is a known triplet quencher, but it did not have a large impact in this reaction, nor did added stilbene (entries 14–15). Organic photocatalyst, tetradiphenylaminophthalonitrile (**7a**, 4DPAPN), provided better results than classical Ir(ppy)_3_ (Table 1, entry 16). This was mainly due to the lower amount of protodehalogenation product **6**. Another organic photocatalyst, a more reductive tetracarbazolisophthalonitrile (4CzIPN), also gave more dehalogenation product (entry 17). Protodehalogenation likely proceeds through photoreduction of aryl halide to form an aryl radical followed by hydrogen atom transfer.^19^ It took place in the absence of nickel and was affected by the reductive properties of the photocatalyst. To further increase the selectivity, we synthesized and characterized a series of tetradiarylaminephthalonitriles^15a,20^ with different electron-donating substituents to improve the photocatalytical properties (Table 2). We observed that a large E(PC/PC^–^) correlates with an increased activity, while a smaller E*(PC*/PC^–^) roughly correlates with increased selectivity. However, for photocatalysts with a very low E*, the reaction stops almost completely as was seen with MeO-substituted **7b**, **8b**, and **9b**. This was likely because the photocatalysts were not able to oxidize the amine or TMG at this point. From the studied photocatalysts, 4DPAPN-^*t*^Bu **7c** had the highest selectivity with high activity and therefore was chosen for further studies.

**Table 1:**
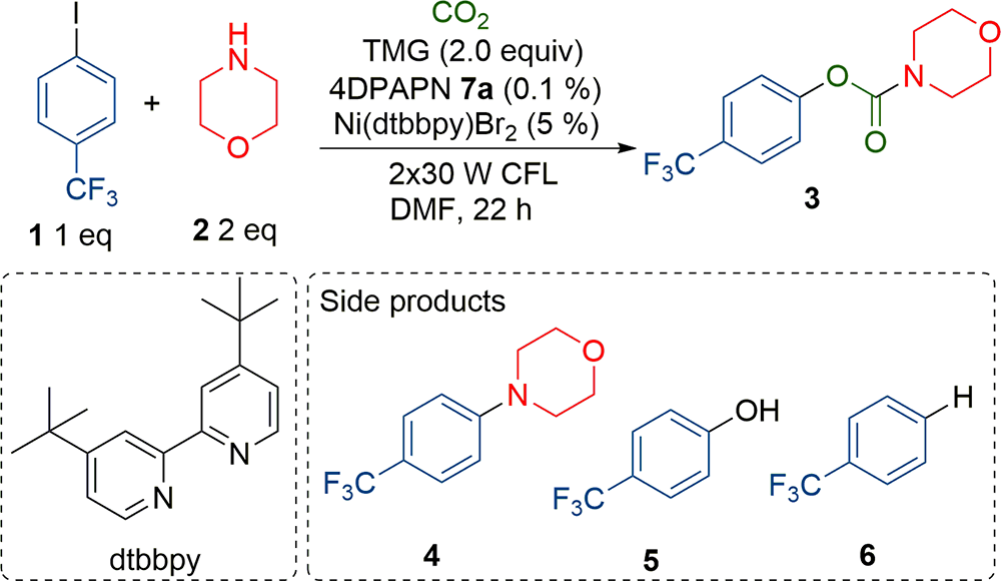
Effect of Selected Alterations to the Reaction

Entry	Deviation from above	Yield (%)^a^
1	None	78
2	No nickel	0
3	No photocatalyst (4DPAPN)	<1
4	Dark	0
5	Blue LEDs	66
6	Bipyridine instead of dtbbpy	28
7	1.1 equiv of morpholine	72
8	1.1 equiv of morpholine and TMG	62
9	*i*-Pr_2_NH as a base	44
10	*i*-Pr_2_NEt as a base	9
11	Cs_2_CO_3_ as a base	77
12	DBU as a base	69
13	Added 1 equiv of H_2_O	65
14	Added 1 equiv of O_2_	68
15	Added 1 equiv of stilbene	76
16	Ir(ppy)_3_ instead of 4DPAPN	71
17	4CzIPN instead of 4DPAPN	62

a Yield determined by ^19^F{^1^H}-NMR with hexafluorobenzene as internal standard.

**Table 2:**
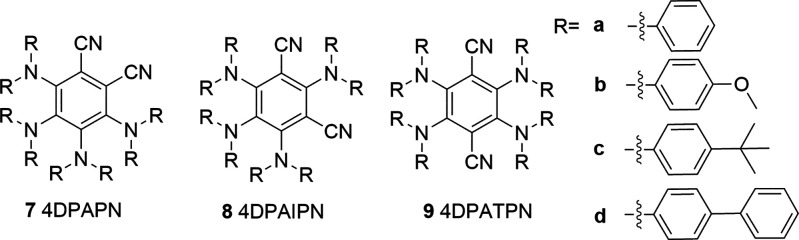
Photocatalyst Ground and Exited State Reduction Potentials and Yields of *O*-Aryl Carbamate **3**

PC	Acronym	*E*	E*	Yield^a^	Selectivity^b^
**7a**	4DPAPN	–1.54^c^	0.90	78%	78%
**8a**	4DPAIPN	–1.66^c^	0.84	72%	72%
**9a**	4DPATPN	– 1.45	0.82	59%	59%
**7b**	4DPAPN-OMe	–1.64	0.73	0%	–
**8b**	4DPAIPN-OMe	–1.80^c^	0.51	16%	78%
**9b**	4DPATPN-OMe	–1.54	0.54	8%	74%
**7c**	4DPAPN-^*t*^Bu	–1.60^c^	0.74	87%	87%
**8c**	4DPAIPN-^*t*^Bu	–1.67^c^	0.71	79%	79%
**9c**	4DPATPN-^*t*^Bu	–1.36	0.80	50%	86%
**7d**	4DPAPN-Ph	–1.44^c^	0.87	41%	58%
**8d**	4DPAIPN-Ph	–1.56^c^	0.83	72%	72%
**9d**	4DPATPN-Ph	–1.49	0.69	19%	70%

a Reaction conditions: 0.4 mmol of 4-iodobenzotrifluoride, 0.8 mmol of TMG, 0.8 mmol of morpholine, 0.02 mmol of NiBr_2_dtbbpy, 0.004 mmol of photocatalyst in DMF (0.1 M). Flushed with CO_2_. 2 × 30W CFL, 22 h, 43 °C. Yield measured by ^19^F{^1^H}-NMR with hexafluorobenzene as internal standard.

b Selectivity = Yield/Conversion.

c Measured in acetonitrile instead of DMF due to low solubility.

**Scheme 2:**
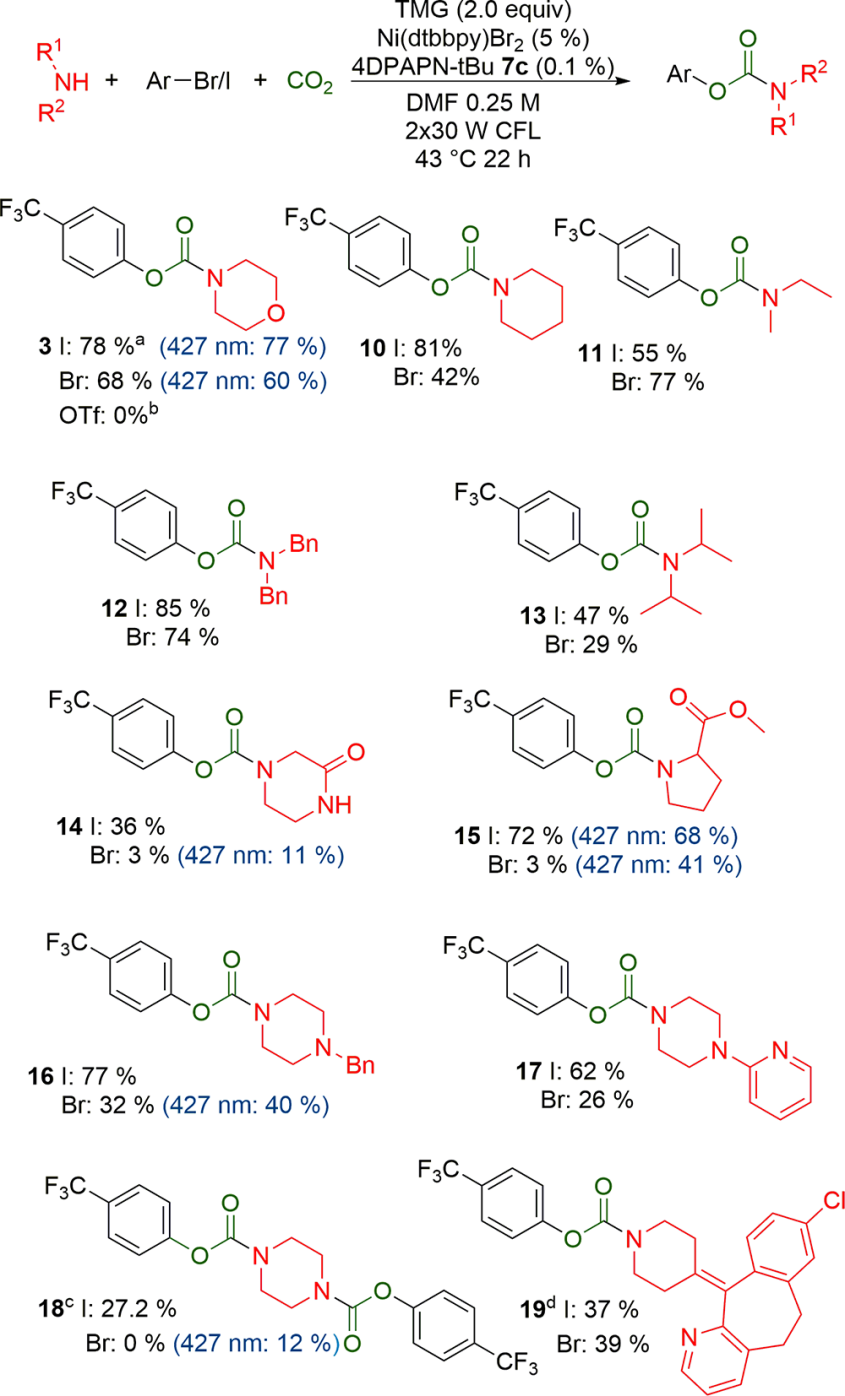
Reaction Scope with Different Amines Reaction conditions: 1.0 mmol of 4-iodo/bromobenzotrifluoride, 2.0 mmol of TMG, 2.0 mmol of amine, 0.05 mmol of Ni(dtbbpy)Br_2_, 1.0 μmol of 4DPAPN-tBu **7c**, diluted to 4 mL with DMF. Flushed with CO_2_. 2 × 30W CFL, 22 h, 43 °C. Isolated yield for iodides, NMR-yield in parentheses, NMR-yield for bromides. Blue LEDs instead of CFL in breackets. (a) Isolated yield, NMR-yield was 86%. (b) Only phenol is formed. (c) 1.0 mmol of piperazine, 2.0 mmol of iodobenzotrifluoride. (d) Diluted to 0.05 M for solubility.

With the optimized conditions, we studied the substrate scope of the reaction (Scheme 2). A variety of secondary alkyl amines, both cyclic and acyclic, were used in the reaction with 4-iodobenzotrifluoride **1** providing the product carbamate in good to moderate yields. Typically, **1** was completely consumed overnight, and protodehalogenation selectivity limited the yield. 4-Bromobenzotrifluoride was also studied, which generally gave slightly poorer yields than with 4-iodobenzotrifluoride, but for carbamates **14**, **15**, and **18**, only traces were observed. In these cases, also the conversion was very low, indicating that the amine or carbamate anion inhibits the catalyst. Compounds **14**, **15**, and **18** contain an additional carbonyl group which may have caused competitive coordination to the metal. Increased amine/carbamate coordination to the metal could inhibit oxidative addition, which was more significant for the less reactive bromo compounds. Using more intense blue LEDs gave a higher yield than with white light CFLs. With amines that have high conversion, using blue LEDs provided a slightly lower yield due to increased dehalogenation. The analogous triflate yielded no carbamate **3**, only the corresponding phenol. Primary amines yielded no carbamate product; instead, the corresponding *N*-arylated amine was the major product. Primary aryl amines also yielded N-arylated product, but secondary aryl amines were unreactive (SI, Figure S7).

**Scheme 3:**
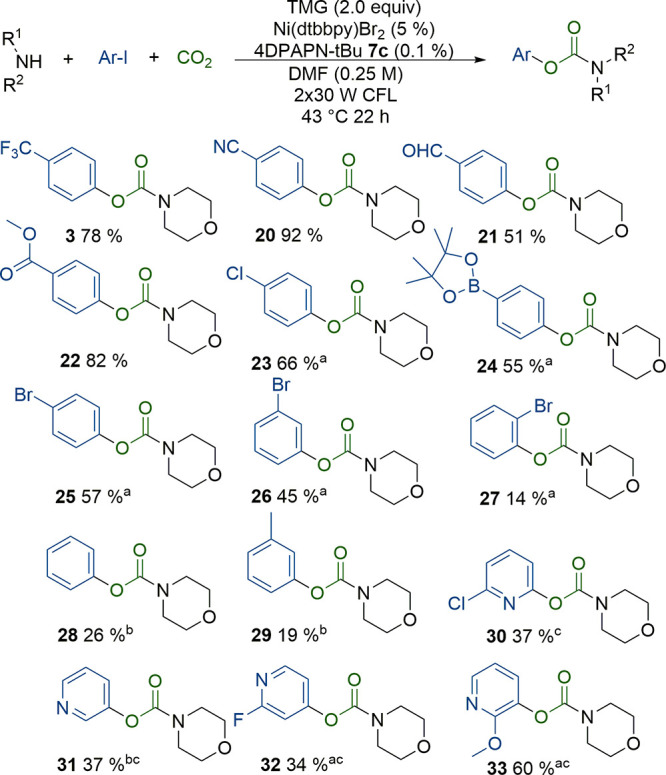
Reaction Scope with Different Aryl Iodides Reaction conditions: 1.0 mmol of aryl iodide, 2.0 mmol of tetramethylguanidine, 2.0 mmol of amine, 0.05 mmol of Ni(dtbbpy)Br_2_, 1.0 μmol of 4DPAPN-tBu **7c**, diluted to 4 mL with DMF. Flushed with CO_2_. 2 × 30W CFL, 22 h, 43 °C. (a) 44 h reaction time. (b) 66 h reaction time. (c) 1.1 mmol of morpholine.

Various electron-deficient aryl iodides were compatible, and the yield was increased by the more electron-withdrawing substituents (Scheme 3). High selectivity was observed for iodo reactivity over bromo (**25**–**27**), and only traces of bromo coupled product were observed for bromoiodobenzenes. However, some dehalogenation of the product was still observed. More electron-rich aryl iodides required a longer reaction time and very electron-rich substituents are unreactive (**28**, **29**).

Iodopyridines could also be used as substrates (Scheme 3, **30**–**33**). Corresponding products are known to be be difficult to prepare from the phenol starting material due to tautomerization.^3^ Moderate yields were obtained for substituted 2-, 3-, or 4-iodopyridines. It was observed that the desired carbamates **30**–**33** could react further with excess amine to form unwanted ureas, reducing the overall yield. The use of only 1.1 equiv of the amine suppressed the urea formation. Of the studied pyridines, the highest yield was obtained for 2-methoxy-3-iodopyridine **33**, which was likely because of the electron-donating methoxy-substituent stabilized carbamate **33** against urea formation.

**Scheme 4:**
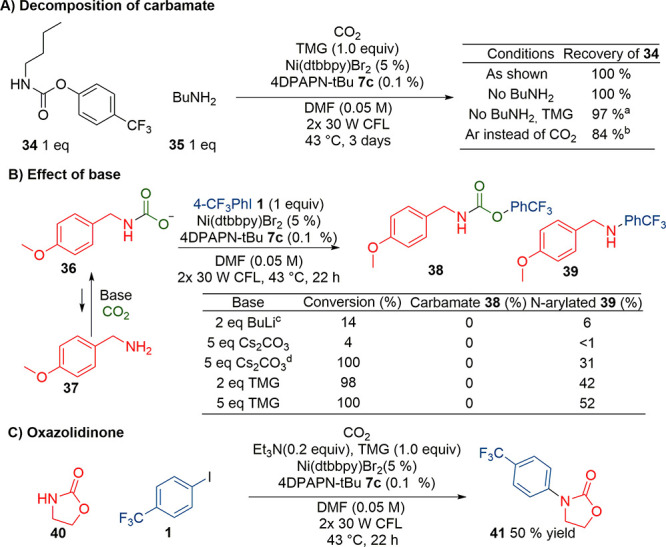
Investigation on to Primary Amine Reactivity: (A) Study of Primary Amine Derived Carbamate Decomposition under Reaction Conditions; (B) Effect of Base to the Reaction Selectivity; (C) Reaction with Cyclic Carbamate **40** Shows That N-Reactivity of Carbamates Is Possible 3% N-arylated product. 13% N-arylated product. Lithium carbamate of **36** was premade in THF. Added 0.2 equiv of Et_3_N.

We further investigated why primary amines yielded N-arylated amine instead of the carbamate. We first studied if *O*-aryl carbamates derived from primary amines decomposed under the reaction conditions. Independently synthesized *N*-butyl-carbamate **34** was placed under the reaction conditions, but even after 3 days there was no observable decomposition (Scheme 4A). We then repeated the reaction in the absence of carbon dioxide. This led to the formation of *N*-arylated product in 13% yield which indicates that this was not the cause for poor reactivity. We also studied if carbamate–amine equilibrium was causing the direct amine reactivity (Scheme 4B; see SI Table S16 for details). With stronger base Cs_2_CO_3_ or BuLi, the reactivity was nearly fully suppressed, which may have been caused by a lack of suitable reducing agent in the absence of TMG or secondary amine. To study this, we added 0.2 equiv of trimethylamine as a reductant, which provided full conversion and yielded 31% N-arylated product **39**. However, no carbamate **38** was formed. Increasing TMG from 2 to 5 equiv also did not afford carbamate **38**. Instead, even higher selectivity toward *N*-arylation was observed. This effect could be explained by the carbamate anion **36** reacting directly from the nitrogen instead of having to dissociate to the amine **37**. Direct N-arylation of carbamate under standard reaction conditions was supported by cyclic carbamate **40** reacting in 50% yield (Scheme 4C).^12^*O*-Arylation of primary carbamates was therefore heavily disfavored in the reaction conditions as not even traces of the product were detected, while *N*-arylation was even more favored since it could take place even without decarboxylation.

Previous studies on other nickel catalyzed carbon–heteroatom coupling reaction have suggested a small number of alternative mechanisms.^11a,21^ A major difference was how the reductive elimination proceeds. One of two mechanisms was usually suggested in C–N and C–O coupling reactions: Energy transfer from photocatalyst to Ni(II) complex; or alternatively, a Ni(I–III) cycle that is propagated by the photoredox cycle. Overall, both of these have been shown to be plausible mechanisms and can likely take place concurrently. The major pathway is dependent on the minute details of the reaction. Compared to the most related esterification reaction using the 4DPAPN photocatalyst, which was thought to proceed through the energy transfer mechanism,^15a^ they observed significant quenching of reactivity when the reaction was conducted in the presence of air. In Table 1, entry 14, it was shown that addition of pure oxygen has only a very modest effect on the yield, which suggests that our reaction proceeds through a different mechanism.

**Figure 1:**
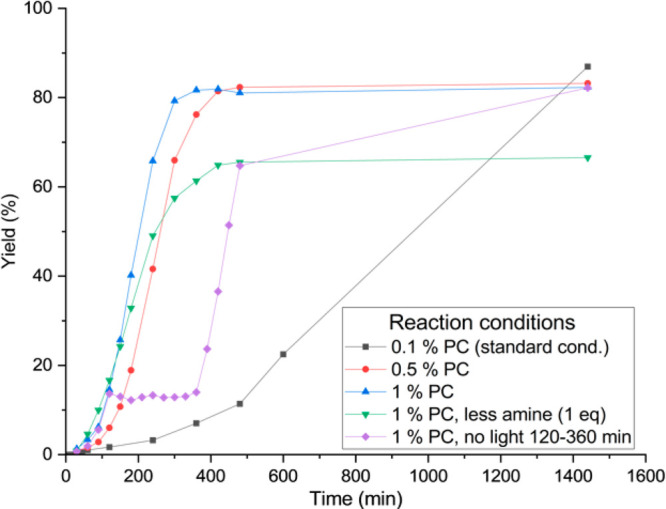
Carbamate formation over time (min). 0.2 mmol of 4-iodobenzotrifluoride, 0.4 mmol TMG and morpholine, 0.01 mmol Ni(dtbbpy)Br2, 0.2 umol 4DPAPN-tbu in 8 ml of DMF. Yield was measured with ^19^F{^1^H}-NMR with hexafluorobezene as internal standard.

To gain insight into the mechanism of this reaction, we measured the reaction profile as a function of time with ^19^F-NMR (Figure 1; see SI part 13 for details). There was a ca. 6 h induction period when using 0.1% of the photocatalyst 4DPAPN-^*t*^Bu **7c**, and the reaction did not reach the maximum rate. When the loading of photocatalyst was increased to 0.5%, the induction period was reduced to 2 h. A loading of 1.0% minimized the induction to a mere 30 min. These observations suggest that the photocatalyst facilitated the reduction of nickel to the active form. With only 1 equiv of tetramethylguanidine and morpholine the reaction rate was roughly the same as with 2 equiv, but the conversion only reaches around 80% conversion. When 5% nickel was used, the maximum reaction rate was reached with 0.5% of 4DPAPN-^t^Bu **7c**, and the reaction reaches completion after 7 h. However, less dehalogenation was observed with 0.1% of 4DPAPN-^t^Bu **7c**, which resulted in higher yield. When irradiation was stopped after the activation period, the reaction stopped very quickly (Figure 1) and there was no detected conversion in the dark. After the irradiation was continued, the reaction proceeded without a new activation period, indicating that the catalyst resting state was different from the initial inactivated form. While this behavior was indicative of the energy transfer mechanism, similar results were also observed in C–N coupling, which proceeded through the Ni(I–III) cycle.^21^ This observation could be explained by fast deactivation by comproportionation, which requires constant reactivation by the photocatalyst.

**Scheme 5:**
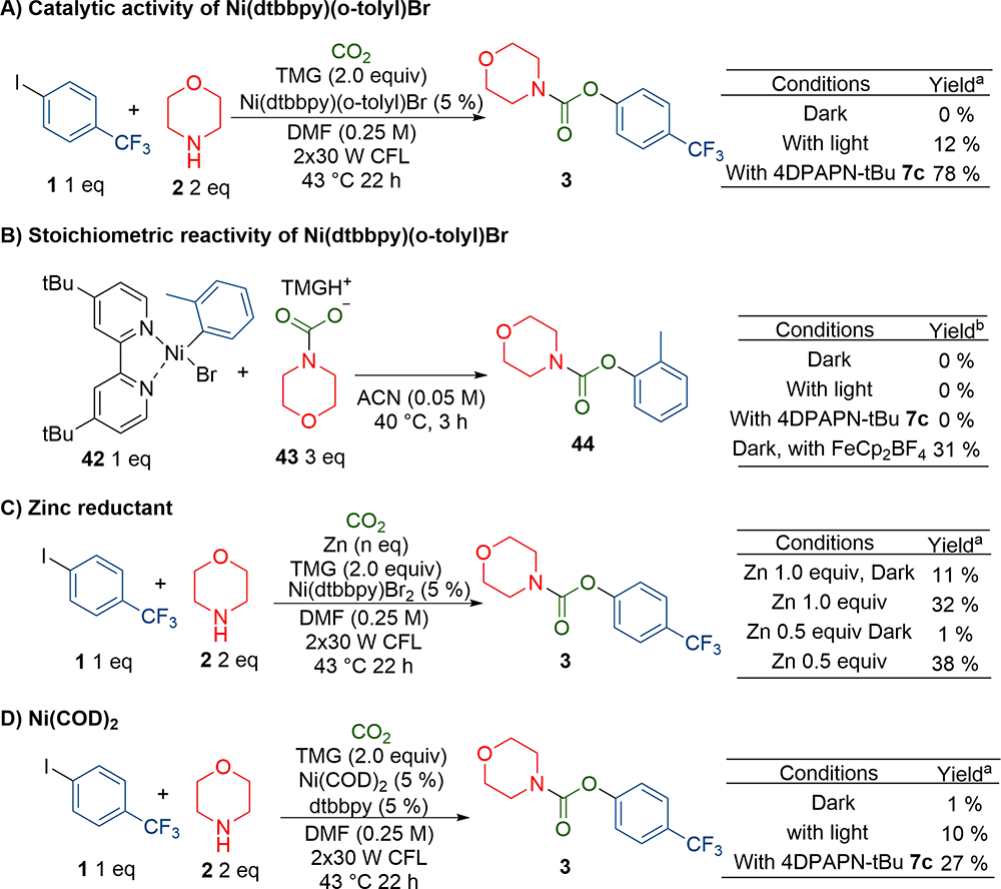
Study of Catalytic Activity and Stoichiometric Reactivity of Ni(dtbbpy)(*o*-tolyl)Br Measured with ^19^F{^1^H}-NMR, with hexafluorobenzene as internal standard. Measured with GC-MS yields with mesitylene as internal standard calibrated with a genuine sample.

The potential intermediate dtbbpyNi(II)(*o*-tolyl)Br **42** was synthesized to determine whether reductive elimination was mediated by the excited state or by Ni(III). It was catalytically active and yielded 78% of carbamate **3** with photocatalyst, 12% without, and 0% in the dark (Scheme 5A); however, no *o*-tolyl carbamate **44** was detected.

When compound **42** was mixed with 3 equiv of tetramethyl guanidium morpholine carboxylate and irradiated with or without photocatalyst, only toluene was observed. With the addition of an oxidant, ferrocenium tetrafluoroborate, formation of *o*-tolyl morpholine-*N*-carboxylate was observed (Scheme 5B). This suggested that the reductive elimination proceeded through a Ni(III) species instead of an excited Ni(II)*. With only 1 equiv of tetramethyl guanidium morpholine carboxylate, no product was generated. Instead, some bromotoluene was observed, suggesting incomplete replacement by the carbamate ion. This would also explain why a moderate excess of the carbamate anion was beneficial for the reaction.

To further study if light had an additional effect beyond reducing the nickel, we used zinc as a reductant in the dark (Scheme 5C). With 1 equiv of zinc, full conversion was obtained, but only 10% carbamate **3** formed. When this reaction was repeated in light, but without photocatalyst, 32% carbamate was obtained (see SI, Table S17 for further details). Alternatively, when already reduced Ni(COD)_2_ was used as the nickel source instead to synthesize carbamate **3**, the reaction gave <1% yield in dark, 10% yield in light, and, with 4DPAPN-^t^Bu**7c**, a 27% carbamate yield (Scheme 5D). However, even in the presence of the photocatalyst, the Ni(COD)_2_-derived catalyst deactivates long before reaching full conversion (SI, Figures S52–54). Overall, these findings suggest that while the carbamate was formed only through the nickel(III) complex instead of direct photoexcitation, light could still facilitate the reaction in some way. Irradiation of similar nickel(II) complexes to **42** was reported to promote formation of Ni(I) and Ni(III) species via photoinduced disproportionation, which could explain the effect of light when Zn or Ni(COD)_2_ was used.^22^ This could also be a minor pathway in the reaction, but since the yield was quite low, it is unlikely to be the main mechanism.

**Scheme 6:**
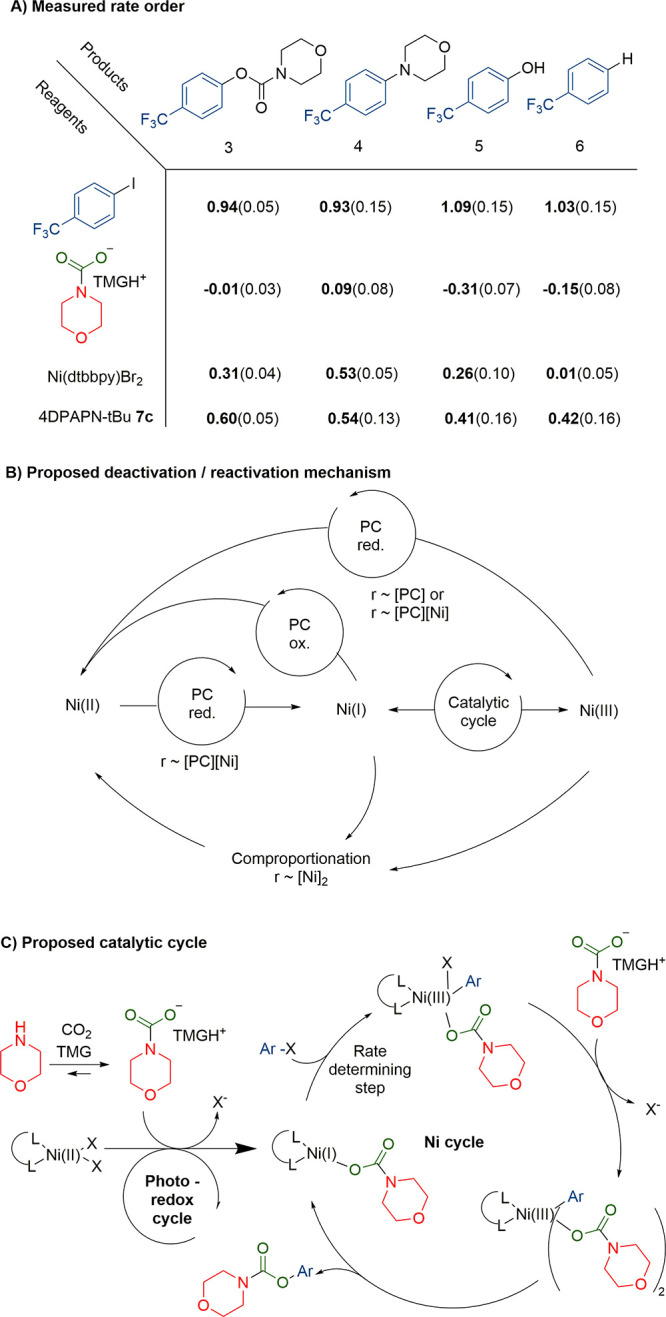
(A) Measured Rate Order Coefficients for Each Reagents (Root Mean Square Error in Brackets); (B) Plausible Mechanisms for Catalyst Deactivation and Reactivation Which Lead to Fractional Rate Orders; (C) Possible Catalytic Cycle

Reaction profiles were measured for different concentrations of the reagents 4-iodobenzotrifluoride **1**, tetramethylguanadinium morpholine-*N*-carbaxylate, 4DPAPN-^t^BU **7c**, and Ni(dtbbpy)Br_2_ (Scheme 6A; see SI, chapter 13 for details), and approximate rate orders were determined from the maximum rate of the sigmoidal reaction curve. The substrate 4-iodobenzotrifluoride had first rate order, while the tetramethylguanadinium morpholine-*N*-carbaxylate was zeroth order. The nickel complex had 0.3 rate order, and the photocatalyst had a 0.5–0.6 rate order. Fractional rate order constants for the nickel and the photocatalyst suggest that they affect the concentration of the active nickel catalyst in a complex way. This could be explained by the active nickel I or III becoming deactivated Ni(II) via comproportionation or in a reaction with single electron transfer species generated by the photocatalyst (Scheme 6B). The nickel is then reactivated by the photocatalyst in a slow step. Table 1, entry 11 and Scheme 4B suggest that either TMG or the secondary amine can act as the terminal reductant. The side product formation had roughly the same rate orders as carbamate formation, taking in account error due to low concentration. An exception was dehalogenation reaction, which was zeroth order in terms of nickel concentration, suggesting it to be a solely photocatalyzed reaction.

Based on the mechanistic studies, the reaction behaved similarly to the amine-aryl halide coupling studied more thoroughly by MacMillan et al.,^21^ which proceeds through the Ni(I–III) cycle initiated and kept active by the photocatalyst (Scheme 6C). Compared to the reactions where energy transfer is the main mechanism, in this reaction we could not observe product with photoexcitation of the direct intermediate (Scheme 5B) and triplet quenchers had nearly no effect on the reaction (Table 1, entries 14, 15).^15a^ Overall, photocatalytic reduction of the initial Ni(II) to Ni(I) was followed by an oxidative addition of aryl halide to yield the Ni(III) complex. The coordinated halide was then replaced by the carbamate ion, as an excess of carbamate ion was vital for product formation in stoichiometric experiments (Scheme 5B). Reductive elimination yielded the product carbamate and regenerated the Ni(I) complex.

## Conclusions

We have developed a method to synthesize *O*-aryl carbamates under mild conditions using visible light dual nickel photocatalysis, which avoids toxic phosgene, stoichiometric reagents, or high pressures of CO_2_. Several new photocatalysts were synthesized and characterized, which had a critical role in improving the selectivity of the reaction. Mechanistically, the reaction proceeded through the Ni(I–III) cycle, which was sustained by the photocatalyst. Our results support the mechanistic understanding of dual nickel photocatalyzed heteroatom–carbon coupling reactions.

## Data Availability

The data underlying this study are available in the published article and its Supporting Information.

## Supporting Information Available

Experimental details, NMR and IR-spectra, voltammograms, absorption and fluorescence spectra (PDF)

## References

[ref1] Ghosh A. K.; Brindisi M. Organic Carbamates in Drug Design and Medicinal Chemistry. J. Med. Chem. 2015, 58 (7), 2895–2940. 10.1021/jm501371s. 25565044 PMC4393377

[ref2] aSlocombe R. J.; Hardy E. E.; Saunders J. H.; Jenkins R. L. Phosgene Derivatives. The Preparation of Isocyanates, Carbamyl Chlorides and Cyanuric Acid. J. Am. Chem. Soc. 1950, 72 (5), 1888–1891. 10.1021/ja01161a009.

[ref3] Breugst M.; Mayr H. Ambident reactivities of pyridone anions. J. Am. Chem. Soc. 2010, 132 (43), 15380–15389. 10.1021/ja106962u. 20942421

[ref4] aNiemi T.; Repo T. Antibiotics from Carbon Dioxide: Sustainable Pathways to Pharmaceutically Relevant Cyclic Carbamates. Eur. J. Org. Chem. 2019, 2019 (6), 1180–1188. 10.1002/ejoc.201801598.

[ref5] aFarshbaf S.; Fekri L. Z.; Nikpassand M.; Mohammadi R.; Vessally E. Dehydrative condensation of β-aminoalcohols with CO2: An environmentally benign access to 2-oxazolidinone derivatives. J. CO2 Util. 2018, 25, 194–204. 10.1016/j.jcou.2018.03.020.

[ref6] aLuo X.; Song X.; Xiong W.; Li J.; Li M.; Zhu Z.; Wei S.; Chan A. S. C.; Zou Y. Copper-Catalyzed C–H Carbamoyloxylation of Aryl Carboxamides with CO2 and Amines at Ambient Conditions. Org. Lett. 2019, 21 (7), 2013–2018. 10.1021/acs.orglett.9b00122. 30817162

[ref7] Retrieved from go.drugbank.com.

[ref8] Watson R. B.; Butler T. W.; DeForest J. C. Preparation of Carbamates, Esters, Amides, and Unsymmetrical Ureas via Brønsted Acid-Activated N-Acyl Imidazoliums. Org. Process Res, Dev. 2021, 25 (3), 500–506. 10.1021/acs.oprd.0c00445.

[ref9] aCorcoran E. B.; Pirnot M. T.; Lin S.; Dreher S. D.; DiRocco D. A.; Davies I. W.; Buchwald S. L.; MacMillan D. W. C. Aryl amination using ligand-free Ni(II) salts and photoredox catalysis. Science 2016, 353 (6296), 279–283. 10.1126/science.aag0209. 27338703 PMC5027643

[ref10] aMacQueen P. M.; Tassone J. P.; Diaz C.; Stradiotto M. Exploiting Ancillary Ligation To Enable Nickel-Catalyzed C–O Cross-Couplings of Aryl Electrophiles with Aliphatic Alcohols. J. Am. Chem. Soc. 2018, 140 (15), 5023–5027. 10.1021/jacs.8b01800. 29601188

[ref11] aWelin E. R.; Le C.; Arias-Rotondo D. M.; McCusker J. K.; MacMillan D. W. C. Photosensitized, energy transfer-mediated organometallic catalysis through electronically excited nickel(II). Science 2017, 355 (6323), 380–385. 10.1126/science.aal2490. 28126814 PMC5664923

[ref12] Reddy L. R.; Kotturi S.; Waman Y.; Ravinder Reddy V.; Patel C.; Kobarne A.; Kuttappan S. N-Arylation of Carbamates through Photosensitized Nickel Catalysis. J. Org. Chem. 2018, 83 (22), 13854–13860. 10.1021/acs.joc.8b02182. 30299099

[ref13] aEscobar R. A.; Johannes J. W. A Unified and Practical Method for Carbon–Heteroatom Cross-Coupling using Nickel/Photo Dual Catalysis. Chem.—Eur. J. 2020, 26 (23), 5168–5173. 10.1002/chem.202000052. 32065838

[ref14] aIshida N.; Masuda Y.; Imamura Y.; Yamazaki K.; Murakami M. Carboxylation of Benzylic and Aliphatic C-H Bonds with CO(2) Induced by Light/Ketone/Nickel. J. Am. Chem. Soc. 2019, 141 (50), 19611–19615. 10.1021/jacs.9b12529. 31775498

[ref15] aLu J.; Pattengale B.; Liu Q.; Yang S.; Shi W.; Li S.; Huang J.; Zhang J. Donor-Acceptor Fluorophores for Energy-Transfer-Mediated Photocatalysis. J. Am. Chem. Soc. 2018, 140 (42), 13719–13725. 10.1021/jacs.8b07271. 30277771

[ref16] Kudisch M.; Lim C.-H.; Thordarson P.; Miyake G. M. Energy Transfer to Ni-Amine Complexes in Dual Catalytic, Light-Driven C–N Cross-Coupling Reactions. J. Am. Chem. Soc. 2019, 141 (49), 19479–19486. 10.1021/jacs.9b11049. 31714761 PMC6941585

[ref17] aNicewicz D.; Roth H.; Romero N. Experimental and Calculated Electrochemical Potentials of Common Organic Molecules for Applications to Single-Electron Redox Chemistry. Synlett 2016, 27 (05), 714–723. 10.1055/s-0035-1561297.

[ref18] Phenols are also formed when the carbamate product further reacts with another equivalent of amine to form the corresponding urea, which was observed in larger amounts with iodopyridines or *tert*-butylamine.

[ref19] Elhage A.; Costa P.; Nasim A.; Lanterna A. E.; Scaiano J. C. Photochemical Dehalogenation of Aryl Halides: Importance of Halogen Bonding. J. Phys. Chem. A 2019, 123 (47), 10224–10229. 10.1021/acs.jpca.9b06716. 31661275

[ref20] Ou W.; Zou R.; Han M.; Yu L.; Su C. Tailorable carbazolyl cyanobenzene-based photocatalysts for visible light-induced reduction of aryl halides. Chin. Chem. Lett. 2020, 31 (7), 1899–1902. 10.1016/j.cclet.2019.12.017.

[ref21] Till N. A.; Tian L.; Dong Z.; Scholes G. D.; MacMillan D. W. C. Mechanistic Analysis of Metallaphotoredox C–N Coupling: Photocatalysis Initiates and Perpetuates Ni(I)/Ni(III) Coupling Activity. J. Am. Chem. Soc. 2020, 142 (37), 15830–15841. 10.1021/jacs.0c05901. 32786779

[ref22] Shields B. J.; Kudisch B.; Scholes G. D.; Doyle A. G. Long-Lived Charge-Transfer States of Nickel(II) Aryl Halide Complexes Facilitate Bimolecular Photoinduced Electron Transfer. J. Am. Chem. Soc. 2018, 140 (8), 3035–3039. 10.1021/jacs.7b13281. 29400956 PMC6698182

