# Supplementary material for: Dual Nickel Photocatalysis for O-Aryl Carbamate Synthesis from Carbon Dioxide
Source: J Org Chem. 2023 Feb 27;88(6):3822–9. doi: 10.1021/acs.joc.3c00023 (PMC10028690; doi:10.1021/acs.joc.3c00023)
Supplement: Supplementary file 1 — jo3c00023_si_001.pdf [file jo3c00023_si_001.pdf]

Supporting information

Dual nickel photocatalysis for *O*-aryl carbamate synthesis from carbon dioxide

Aleksi Sahari†, Jukka Puumi†, Jere K. Mannisto, Timo Repo*

Department of Chemistry

University of Helsinki

FI-00014 University of Helsinki, Finland

E-mail: timo.repo@helsinki.fi

* Corresponding author

† These authors contributed equally to this work

1 CONTENTS

2 Abbreviations S3
3 General information S4
3.1 Materials and methods S4
3.2 Absorption- and fluorescence spectroscopy S4
3.3 Cyclic voltammetry S4
4 Photocatalytic setup S5
5 Initial optimization with an aryl bromide coupling partner S7
5.1 Common side-products S7
5.2 Optimization of the solvent S8
5.3 Optimization of the photocatalyst S9
5.4 Optimization of the ligand S11
5.5 Optimization of the nickel source S12
5.6 Optimization of the catalyst loading S13
5.7 Optimization of the base S13
5.8 Further attempts to improve coupling with the aryl bromide coupling partner S15
5.9 Miscallenous optimization experiments S15
5.10 Replication of published methods S17
5.11 Testing of the modified 4DPAPN-like catalysts S19
6 Optimization with an aryl iodide coupling partner S21
6.1 Iodides vs bromides and optimization of the base S21
6.2 Optimization of the other components to arrive at the optimal conditions S23
6.3 Effect of concentration S24

6.4 Table 1 and 2 side product distribution S26
7 Unsuccessful substrates S27
7.1 List of unsuccessful substrates S27
7.2 Attempts to make primary amines react: coupling with dicarbamate species S30
8 Experiments with zinc as a reductant S32
9 Synthesis of the photocatalysts S33
9.1 General procedure S33
9.2 Synthesis S34
10 Synthesis of O-aryl carbamates S43
10.1 General procedure S43
10.2 Amine coupling partners S44
10.3 Aryl iodide coupling partners S50
11 Other synthesis S57
12 Calculation of the reduction potentials of the photocatalysts S62
13 Absorption- and fluorescence spectra and voltammograms S64
14 Reaction kinetics S71
14.1 Procedure S71
14.2 Linear fit of product formation to reagent concentration S72
14.3 Reaction profiles S75
14.3.1 Morpholine and TMG S75
14.3.2 Iodobenzotrifluoride S77
14.3.3 $NiBr_2$dtbbpy S79
14.3.4 DPAPN-tBu S81
14.3.5 $Ni(COD)_2$ S83
14.3.6 Miscellaneous S84
15 (DTBBPY)Ni(o-tolyl)Br S85
16 References S87
17 NMR and IR spectra S89
17.1 NMR and IR spectra of photocatalysts S89
17.2 NMR and IR spectra of O-aryl carbamates S108
17.3 Other NMR spectra S145

2 ABBREVIATIONS

4CzIPN = tetracarbazoloisophtalonitrile
4DPAPN = tetradiphenylaminophtalonitrile
4DPAIPN = tetradiphenylaminoisophtalonitrile
4DPATPN = tetradiphenylaminoterephtalonitrile
ACN = Acetonitrile
CFL = Compact fluorescent lamp
CV = Cyclic voltammetry
DBU = 1,8-diazabicyclo(5.4.0)undec-7-ene
DCM = Dichloromethane
DIPA = Diisopropylamine
DIPEA = Diisoproylethyl amine
DMAc = Dimethylacetamide
DMF = Dimethylformamide
DMSO = Dimethylsulphoxide
dtbbpy = ditertbutylbipyridine
FTIR = Fourier transform infrared (spectroscopy)
GC = Gas chromatography
HRMS = High resolution mass spectrometry
NMP = N-methylpyrrolidone
NMR = Nuclear magnetic resonance
MS = Mass spectrometry
PC = Photocatalyst
ppy = phenylpyridine
SCE = Saturated calomel electrode
TBAI = Tetrabutylammonium iodide
t-BIPA = Tertbutylisopropylamine
TMG = Tetramethylguanidine

3 GENERAL INFORMATION

3.1 MATERIALS AND METHODS

All reagents were purchased from commercial sources and were not purified further if not stated otherwise. Amines, aryl halides and substrates for photocatalyst synthesis were supplied by Fluorochem and Merck. Morpholine was distilled over KOH and all solvents were dried over 3Å molecular sieves.

NMR experiments were run with Bruker AS400 or AS500 NMR in $CDCl_3$. Chemical shifts are reported relative to residual $CHCl_3$ (δ = 7.26 for ^{1}H and 77.16 for ^{13}C or C_6F_6 (δ = –164.9 for ^{19}F). GC-MS experiments were run with Agilent 6890N Network Gas Chromatograph coupled with Agilent 5973 Network Mass Selective Detector. HRMS (ESI) was recorded with Bruker Micro TOF system. IR spectra was recorded with Bruker Alpha Platinum-ATR FT-IR.

3.2 ABSORPTION- AND FLUORESCENCE SPECTROSCOPY

Absorption spectra were measured using Ocean Optics USB4000 spectrophotometer. Fluorescence measurements were done by using a Horiba Jobin Yvon FluoroMax-4 spectrofluorometer. In both measurements, 0.025 mg/ml DMF solutions were used. In the case of photocatalysts 4DPATPN-^{t}But and 4DPATPN-Ph, a saturated solution was used as these photocatalyst were not soluble enough to yield 0.025 mg/ml). With photocatalysts 4DPAIPN and 4DPAIPN-Ph 0.001 mg/ml solutions were used as 0.025 mg/ml concentration lead to the saturation of the detector. 450 nm light corresponding to blue light was used for the excitation as most of the PC:s had strong absorption in that range. PC:s 4DPATPN-OMe and 4DPATPN-^{t}Bu were excited with 390 and 380 nm respectively because these PC:s had no absorption with 450 nm.

3.3 CYCLIC VOLTAMMETRY

Cyclic voltammetry (CV) measurements were performed as follows: Photocatalyst and ferrocene (Fc, sublimed under atmospheric pressure) were dissolved (both 1 mM) using an electrolyte solution (0.2 M Bu_4NPF_6 in acetonitrile or DMF pre-dried with 3Å molecular sieves and distilled over CaH_2). Autolab PGSTAT 20 potentiostat along with ALS SVC-3 voltammetry cell with glassy carbon working electrode (WE) with 3 mm diameter, platinum wire counter electrode (CE) and Ag/Ag^+ (0.01 M $AgNO_3$ in electrolyte solution) reference electrode (RE) were used. The electrochemical cell was degassed by bubbling nitrogen for about 10 minutes. A pre-bubbler was used to avoid concentration

and possible precipitation of the photocatalyst.[1] The scan rate was set to 50 mV/s with a 10 mV step potential. In most scans, the scan area vs SCE (saturated calomel electrode) was –2.6 - 2.9 V with ACN or -2.1 – 1.4 V with DMF (with ACN, some solvent reactions was observed at around +2 V). Only one scan per photocatalyst was run. Between runs, the system was thoroughly rinsed with distilled ACN or DMF, the WE was re-polished using 0.05 µm polishing alumina if necessary and a background run (2 cycles at 100 mV/s) was run. The voltammograms were rescaled vs SCE by setting Fc^+/Fc at 0.38 V.[2] The reversible reaction at around -1.5 V vs SCE visible for all photocatalysts was taken to be the PC/PC^- conversion.

4 PHOTOCATALYTIC SETUP

Optimization- and scope experiments were performed with two identical photoreactors. The setup and the emission spectrum of the lights is shown in Figure S1. The reactor consists of a box (length 24.5 cm, width 15.5 cm and height 11.5 cm) with both ends open to allow the use of a cooling fan. A rack that can hold three 8 ml vials is placed between two 30 W 6700K CFL lights about 1.8 cm from the lights. When using the reactor, the reactor is placed on a magnetic stirrer, the vials are placed in the racks and a lid is put on the box. The proximity of the lights raises the temperature of the reaction solutions to about 43 °C.

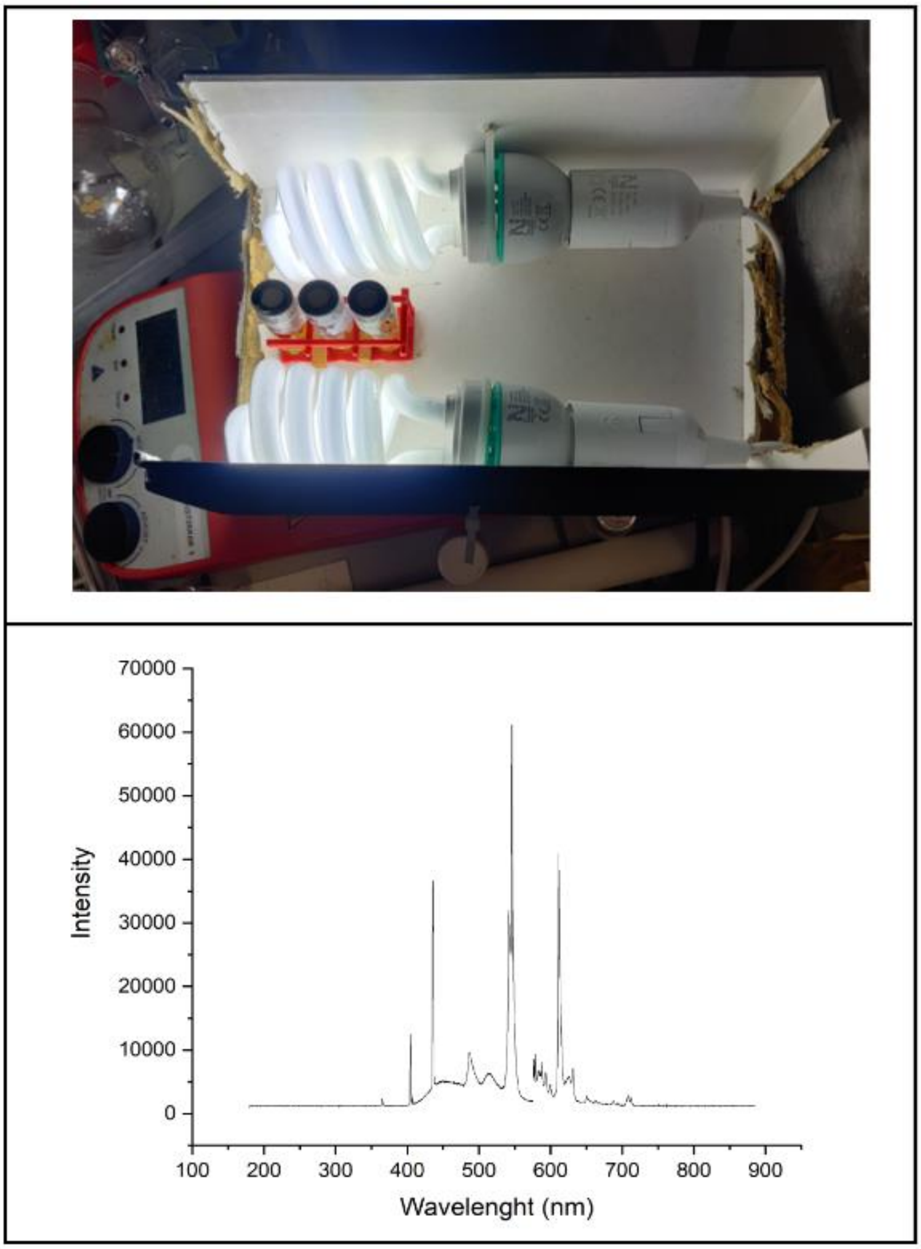

Figure S1. The photoreactor and the spectrum of the CFL lights used.

5 INITIAL OPTIMIZATION WITH AN ARYL BROMIDE COUPLING PARTNER

Optimization of the dual catalyst system was done by selecting morpholine (**1**) as the amine reacting with CO_2 to form the corresponding carbamate. 4-bromobenzotrifluoride (**2**) was selected as the model aryl halide, since it is convenient to analyze with ^{19}F NMR and it is quite electron poor, which is an indicator of high reactivity in dual nickel/photocatalyst cross-couplings. DBU, TMG and other superbases have been shown to be beneficial in forming carbamate salts in high yields, especially with less basic amines like aniline.[3-4] For this reason, DBU was selected as the initial base. Ir(ppy)$_3$ (Tris(2-phenylpyridine)iridium(III)) was selected as the initial PC because it has been used successfully in coupling of carboxylic acids which are similar in structure and nucleophilicity to carbamates.[5-6]

In a typical optimization reaction in an 8 ml vial were added DMF (3 ml), 4-bromobentsotrifluoride (0.2 mmol, 0.05 M), morpholine (0.4 mmol, 2 equiv), $NiBr_2$ and dtbbpy (10-20 µmol, 5-10 mol%) and photocatalyst (2 µmol, 1 mol %). The reaction mixture was diluted to 4 ml and capped with a septum cap. CO_2 was bubbled for 10 sec-10 min. The vial was placed between two 30 W white CFL lights and stirred vigorously (1200 rpm) at 43 °C (due to proximity of the lights) for 18-22 hours. Yields were determined either with GC-MS or ^{19}F NMR. GC-yields were determined by adding mesitylene (0.2 mmol) as an internal standard and then extracting a sample with ethyl acetate and analyzing with GC-MS. ^{19}F NMR yields were determined by adding trifluoroethanol (0.2 mmol) as an internal standard and directly analyzing the resulting solution with ^{19}F NMR using self-made DMSO-d6 inserts to get lock signal.

5.1 COMMON SIDE-PRODUCTS

For convenience, most of the initial optimization studies were analyzed with GC-MS. In the cross-couplings, three side products are commonly observed (Figure S2). The aniline **5** and phenol **6** side products are usually minor. They were not isolated and therefore their exact yields were not evaluated with GC-MS. The dehalogenated side product **4** is very significant in the optimization experiments, almost making up the remainder of species originating from the starting material (Yield of **4** ≈ yield of **3** – unreacted **2**). In optimization studies analyzed with GC-MS, the yield of **4** was not evaluated because it elutes faster than DMF and, since the used GC device does not have FID, removal of DMF would be required making the sample preparation laborious.

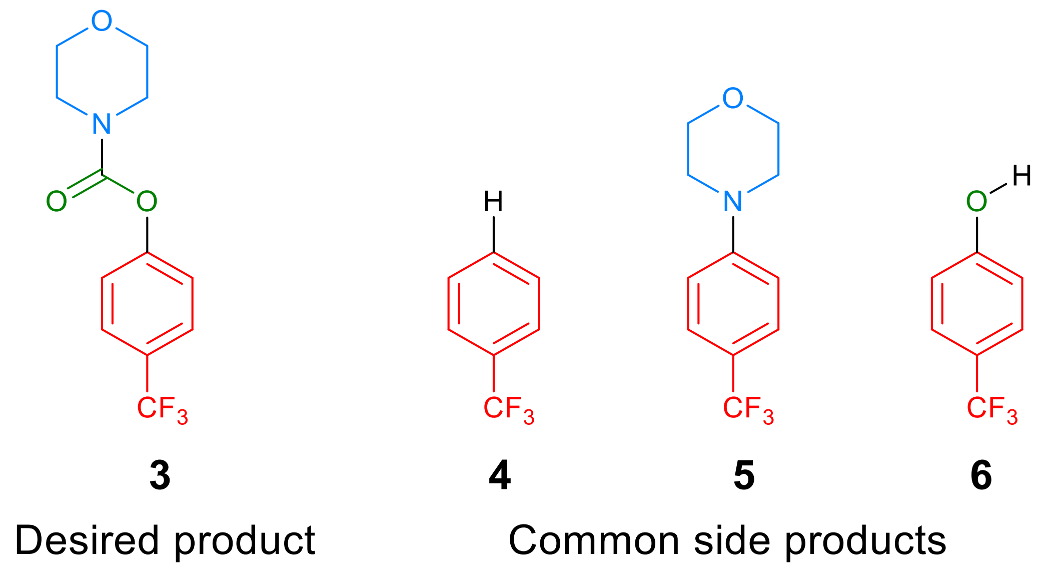

Figure S2. The desired product and common side products.

5.2 Optimization of the Solvent

Optimization results are shown in Table S1. Less polar solvents failed to completely dissolve the catalytic system and consequently failed to give almost any product at all (entries **1** and **2**). In the first set of reactions, DMAc and NMP seemed to work much better than DMF (first set of yields in entries **4-6**). However, when these reactions were repeated, the yield with DMF more than quadruplet, yield with DMAc also increased and the yield with NMP slightly decreased (second set of yields in entries **4-6**). This led us to suspect that variation in the CO_2 bubbling time and pressure led to the loss of 4-bromobenzotrifluoride and consequently to bad reproducibility. This was corrected in the next optimization step by decreasing the bubbling time to exactly 5 minutes and reducing the flow of CO_2.

Table S1. Optimization of the solvent.

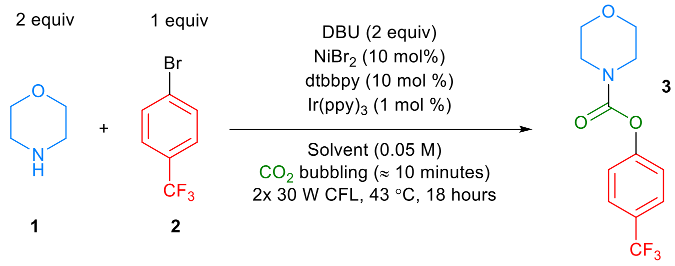

Entry	Solvent	Yield of 3 (%)
**1**[a]	THF	1.1
**2**[a]	$PhCF_3$	0
**3**	ACN	5.3
**4**	NMP	21.1 and 19.3
**5**	DMAc	23.0 and 37.0
**6**	DMF	7.6 and 33.5

Conditions: In an 8 ml vial were added $NiBr_2$ (4.4 mg, 0.02 mmol, 10 mol %), dtbbpy (5.4 mg, 0.02 mmol, 10 mol %) and 3 ml of solvent. The mixture was stirred in an 80 °C water bath until no solids were present. $Ir(ppy)_3$ (1.3 mg, 2 µmol 1 mol%) dissolved in 1 ml of solvent was added followed by 4-bromobenzotrifluoride (28.0 µl, 0.2 mmol, 0.05 M), morpholine (34.8 µl, 0.4 mmol, 2 equiv) and DBU (59.7 µl, 0.4 mmol, 2 equiv). CO_2 was bubbled for about 10 minutes (for some vials significantly longer), after which the vial was capped under CO_2 flow. The vial was placed between two 30 W white CFL lights and stirred vigorously (1200 rpm) at 43 °C for 18 hours. Yields were determined with GC-MS using mesitylene as an internal standard.[a] Nickel complex formation was incomplete

5.3 Optimization of the photocatalyst

Optimization results are shown in Table S2. As suspected, when decreasing the bubbling time and CO_2 flow the yields went up and were more reproducible. The PC is essential for product formation (entry **1**). DMF proved to be superior to DMAc. Only $Ir(ppy)_3$ and an organic photocatalyst 4DPAPN[7] were able to yield complete transformation of the starting material. Cooling down the vial with a fan, resulted in less complete reaction (entries **2** and **9**). Since 4DPAPN worked as well as $Ir(ppy)_3$, if not better, and it is much cheaper and free of rare metals, it was chosen as the optimal photocatalyst (entry **3** vs entry **5**). Even though using higher catalyst loading of 4DPAPN (entry **8**) did improve the yield slightly, the lower catalyst loading of 1 mol% was kept to conserve 4DPAPN.

Table S2. Initial optimization of the photocatalyst.

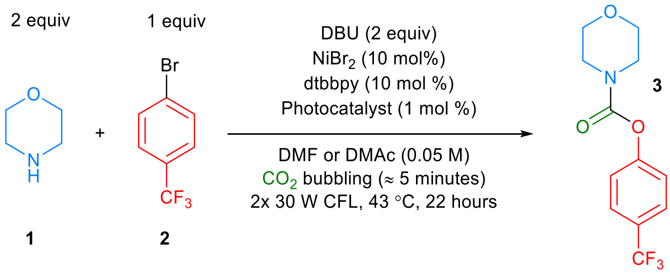

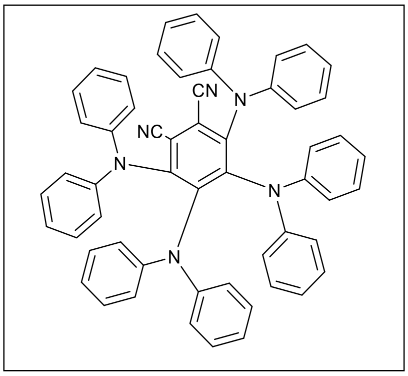

4DPAPN
(3,4,5,6-tetrakis(diphenylamino)phthalonitrile)

**Entry**	**Photocatalyst**	**Solvent**	**Yield of 3 (%)**	**2 remaining (%)**
**1**	None	DMF	0.0	92.8
**2**[a]	Ir(ppy)$_3$ (1 mol%)	DMF	16.4	47.3
**3**	Ir(ppy)$_3$ (1 mol%)	DMF	49.6	0
**4**	Ir(ppy)$_3$ (1 mol%)	DMAc	41.8	0
**5**	4DPAPN (1 mol%)	DMF	50.8	0
**6**	4DPAPN (1 mol%)	DMAc	42.3	0
**7**	4DPAPN (0.5 mol%)	DMF	47.5	0
**8**	4DPAPN (2 mol%)	DMF	52.6	0
**9**[a]	Benzophenone (5 mol%)	DMF	5.0	80.4
**10**	Benzophenone (5 mol%)	DMF	32.4	22.2
**11**	Benzophenone (5 mol%)	DMAc	34.9	39.7
**12**	Michler's ketone (5 mol%)	DMF	43.6	14.5
**13**	Michler's ketone (5 mol%)	DMAc	27.3	49.0
**14**	9-fluorenone (5 mol %)	DMF	1.8	85.4
**15**	Acetophenone (8.6 mol %)	DMF	2.7	86.8
**16**	Ru(bpy)$_3$Cl$_2$·6H$_2$O (1 mol%)	DMF	0.0	94.1

Conditions: In an 8 ml vial were added the photocatalyst (as shown) and DMF or DMAc (3 ml). The mixture was stirred until no solids were present. 0.2 ml of a 1:1 NiBr$_2$:dtbbpy solution (0.1 M DMF or DMAc solution, 0.02 mmol, 10 mol %) was added followed by 4-bromobenzotrifluoride (28.0 µl, 0.2 mmol, 0.05 M), morpholine (34.8 µl, 0.4 mmol, 2 equiv) and DBU (59.7 µl, 0.4 mmol, 2 equiv). The solution was diluted to 4 ml. CO_2 was bubbled for 5 minutes and the vial was capped under CO_2 flow. The vial was placed between two 30 W white CFL lights and stirred vigorously (1200 rpm) at 43 °C for 22 hours. Yields were determined with GC-MS using mesitylene as internal standard.[a] With fan cooling at 22 °C for 15 hours.

5.4 OPTIMIZATION OF THE LIGAND

Optimization results are shown in Table S3. When the ligand is excluded, very small yields are observed (entry **1**). 4,4’-substituted bipyridines were superior to all tested ligands with dtbbpy being optimal (entries **2-5**). Other nitrogen ligands performed poorly (entries **6** and **7**). All phosphine based ligands failed to give any product (entries **8-11**).

Table S3. Optimization of the ligand.

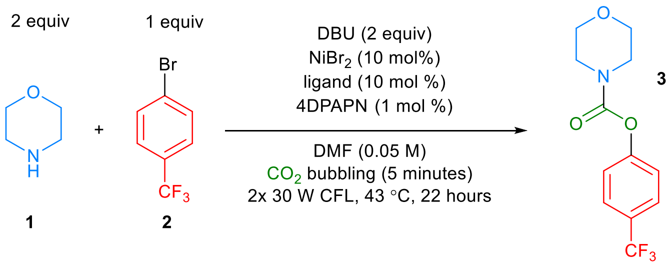

Test	Ligand	Yield of 3 (%)	2 remaining (%)
**1**	No ligand	4.0	83.8
**2**	dtbbpy	50.8	0
**3**	2,2’-bipyridine	22.8	0
**4**	2,2’-bipyridine-4,4’-dicarboxylic acid	41.0	0
**5**	4,4’-Dimethoxy-2,2’-bipyridine	41.0	0
**6**	1,10-phenantroline	16.5	44.9
**7**	Bathocuproine	0.0	60.1
**8**	PPh_3	0.0	81.5
**9**	dppf	0.0	106.1
**10**	Xantphos	0.0	80.7
**11**	BINAP	0.0	73.2
**12**	1,1 Dipyrazolylmethane	0.0	97
**13**	2-(pyrrolidine-1-ylmethyl)pyridine	0.0	98

Conditions: In an 8 ml vial were added $NiBr_2$ (4.4 mg, 0.02 mmol, 10 mol %), ligand (0.02 mmol, 10 mol %) and DMF (3 ml). The mixture was stirred and heated if necessary until no solids were present. 0.1 ml of a 4DPAPN solution (0.02 M in DMF, 2 µmol, 1 mol %) was added followed by 4-bromobenzotrifluoride (28.0 µl, 0.2 mmol, 0.05 M), morpholine (34.8 µl, 0.4 mmol, 2 equiv) and DBU (59.7 µl, 0.4 mmol, 2 equiv). The solution was diluted to 4 ml. CO_2 was bubbled for 5 minutes and the vial was capped under CO_2 flow. The vial was placed between two 30 W white CFL lights and stirred vigorously (1200 rpm) at 43 °C for 22 hours. Yields were determined with GC-MS using mesitylene as internal standard.

5.5 OPTIMIZATION OF THE NICKEL SOURCE

Optimization results are shown in Table S4. Nickel is essential for product formation (entry **1**). Inorganic nickel salts gave very similar results with anhydrous nickel halides and $NiCl_2$·glyme working best (entries **2**, **4** and **6**). Nickel salts with organic anions did not work as well (entries **9** and **11**). The initially selected anhydrous $NiBr_2$ proved to be the optimal nickel source. However, it should be noted that complexation of anhydrous nickel salts with dtbbpy requires heating to about 80 °C for 5 minutes whereas other salts in Table S4 were complexed at room temperature. This extra heating step might not be worth the trouble if one wants to use a freshly made catalyst solution for each experiment. In our experience, however, precomplexed $NiBr_2$ dtbbpy retains its catalytic ability for at least 2 months as a 0.2 M DMF stock solution when stored in the fridge.

Table S4. Optimization of the nickel source.

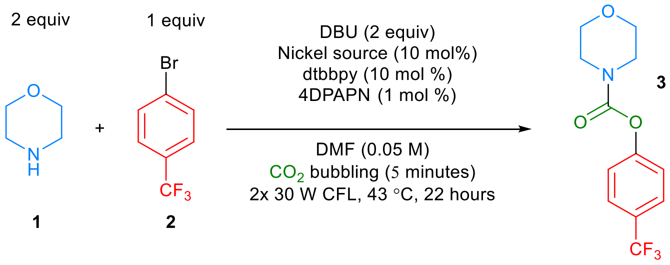

Test	Ni source	Yield of 3 (%)	2 remaining (%)
**1**	No nickel	0	101.2
**2**	$NiBr_2$	55.1	0
**3**	$NiBr_2$·3H_2O	49.3	0
**4**	$NiCl_2$	49.8	8.4
**5**	$NiCl_2$·6H_2O	49.8	0
**6**	$NiCl_2$·glyme	53.6	0
**7**	$Ni(NO_3)$·6H_2O	43.8	0
**8**	$NiSO_4$·6H_2O	46.3	0
**9**	$Ni(OAc)_2$·4H_2O	35.5	0
**11**	$Ni(acac)_2$	9.8	35.7
**12**	$NiCl_2PPh_3IPr^a$	0	94

Conditions: In an 8 ml vial were added nickel source (0.02 mmol, 10 mol %), dtbbpy (5.4 mg, 0.02 mmol, 10 mol %) and DMF (3 ml). The mixture was stirred and heated if necessary until no solids were present. 0.1 ml of a 4DPAPN solution (0.02 M in DMF, 2 μmol, 1 mol %) was added followed by 4-bromobenzotrifluoride (28.0 μl, 0.2 mmol, 0.05 M), morpholine (34.8 μl, 0.4 mmol, 2 equiv) and DBU (59.7 μl, 0.4 mmol, 2 equiv). The solution was diluted to 4 ml. CO_2 was bubbled for 5 minutes and the vial was capped under CO_2 flow. The vial was placed between two 30 W white CFL lights and stirred vigorously (1200 rpm) at 43 °C for 22 hours. Yields were determined with GC-MS using mesitylene as internal standard. a) No dtbbpy

5.6 Optimization of the catalyst loading

Next, the effect of catalyst loading of the 1:1 $NiBr_2$:dtbbpy catalyst was scoped by two reaction series (Figure S3). It was discovered that a lower catalyst loading of 5 mol % gave slightly higher yields than the initially selected 10 mol %.

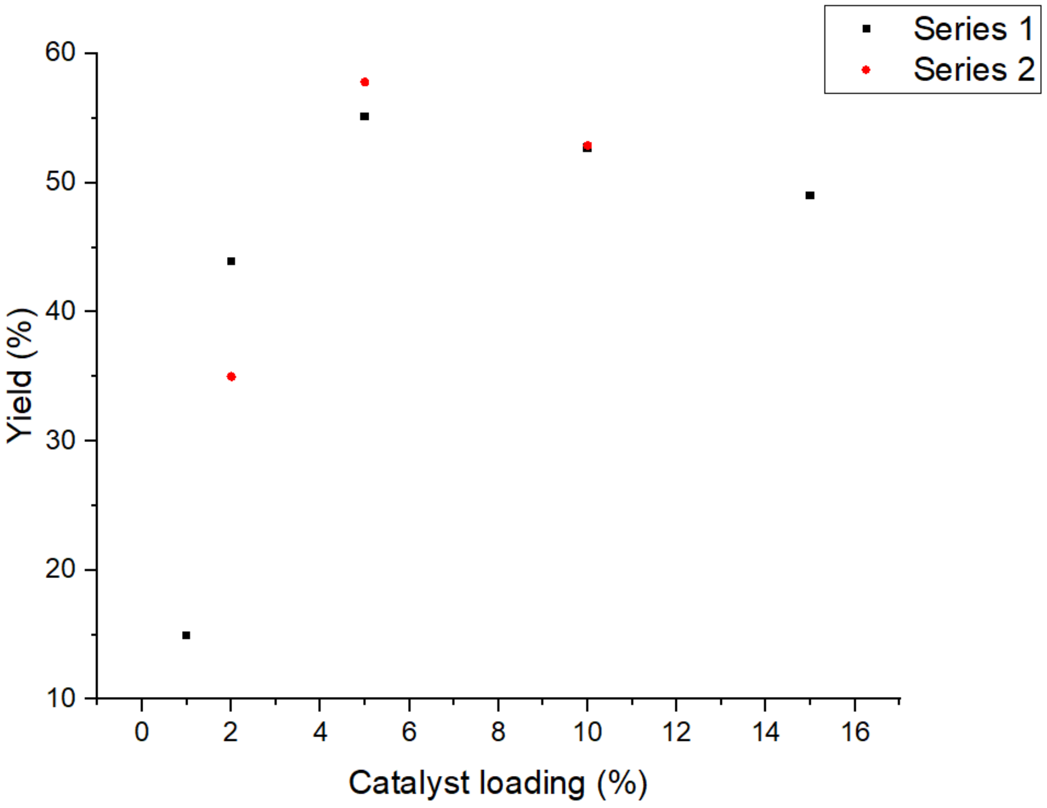

Figure S3. Effect of [$NiBr_2$(dtbbpy)] loading. Conditions: In an 8 ml vial were added 0.1 ml of a 1:1 NiBr2:dtbbpy solution (0.1 M in DMF, 0.01 mmol, 5 mol %), 0.1 ml of a 4DPAPN solution (0.02 M in DMF, 2 µmol, 1 mol %), DMF (3 ml), 4-bromobenzotrifluoride (28.0 µl, 0.2 mmol, 0.05 M), morpholine (34.8 µl, 0.4 mmol, 2 equiv) and DBU (59.7 µl, 0.4 mmol, 2 equiv). The solution was diluted to 4 ml and the vial was capped with a septum cap. CO2 was bubbled for 10 seconds after which the vial was sealed with parafilm. The vial was placed between two 30 W white CFL lights and stirred vigorously (1200 rpm) at 43 °C for 22 hours. Yields were determined with GC-MS using mesitylene as internal standard.

5.7 Optimization of the base

When starting the base optimization, the CO_2 bubbling time was further reduced to only 10 seconds. Such a short bubbling time probably will not reduce the oxygen levels to the levels usually needed for oxygen sensitive reactions like palladium cross-couplings. However, as can be seen from the results on Table S5, this coupling protocol is not sensitive to small amounts of oxygen and full degassing is not required.

Table S5. Optimization of the base.

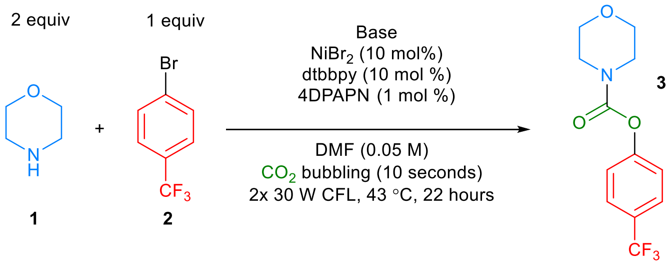

Entry	Base	Base equivalents	Yield of 3(%)	2 remaining (%)
**1**	None	0	6.9	83.7
**2**	DBU	2	57.6	0
**3**	DBU	3	7.7	0
**4**	TMG	2	55.8	0.0
**5**	TMG	3	56.9	0
**6**	TMG (distilled, see **note!**)	2	68.5	17.7
**7**	K_2CO_3	2	18.5	42.1
**8**	Cs_2CO_3	2	56.9	0.0
**9**	Cs_2CO_3	3	57.3	0
**10**	CsF	2	25.1	49.4
**11**	K_3PO_4	2	11.1	50.3
**12**	t-BuOK	2	4.4	40.5
**13**	Et_3N	2	6.2	42.3
**14**	DIPEA	2	4.9	35.0
**15**	DIPA	2	26.3	41.9
**16**	DIPA	5	35.5	41.3
**17**	t-BIPA	2	27.4	52.9
**18**	t-BIPA	4	37.3	27.3

Conditions: In a 8 ml vial was added 0.1 ml of a 1:1 $NiBr_2$:dtbbpy solution (0.1 M in DMF, 0.01 mmol, 5 mol %), 0.1 ml of a 4DPAPN solution (0.02 M in DMF, 2 µmol, 1 mol %), DMF (3 ml), 4-bromobenzotrifluoride (28.0 µl, 0.2 mmol, 0.05 M), morpholine (34.8 µl, 0.4 mmol, 2 equiv) and base (0.4 mmol, 2 equiv). %). The solution was diluted to 4 ml and the vial was capped with a septum cap. CO_2 was bubbled for 10 seconds after which the vial was sealed with parafilm. The vial was placed between two 30 W white CFL lights and stirred vigorously (1200 rpm) at 43 °C for 22 hours. Yields were determined with GC-MS using mesitylene as internal standard.

Use of an external base is required (entry **1**). Amine bases and most inorganic bases performed poorly (entries **7**, **10-18**). Initially, results with DBU and TMG were essentially identical with DBU being slightly better (entries **2-5**). Interestingly, adding three equivalents of DBU resulted in very poor yield

and GC-MS showed significant amounts of various side products (entry **3**). Cs_2CO_3 also gave identical yields to DBU and TMG (entries **8** and **9**). Based on the initial optimization experiments, DBU was selected as the optimal base. **Note!** Unfortunately, the used TMG had some impurity that decreased the yield significantly. Later, when TMG was distilled, better results were achieved with TMG than with DBU. For this reason, DBU was erroneously selected as the optimal base at this stage.

5.8 FURTHER ATTEMPTS TO IMPROVE COUPLING WITH THE ARYL BROMIDE COUPLING PARTNER

While yields close to 60 %, achieved with the model reagents, were adequate taking into consideration the added complexity of the amine-carbamate equilibrium, other tested secondary amine coupling partners gave significantly diminished yields and primary amines yielded only the corresponding directly aminated coupling products (Table S6). For this reason, further optimization was attempted.

Table S6. Initial testing with other carbamate coupling partners.

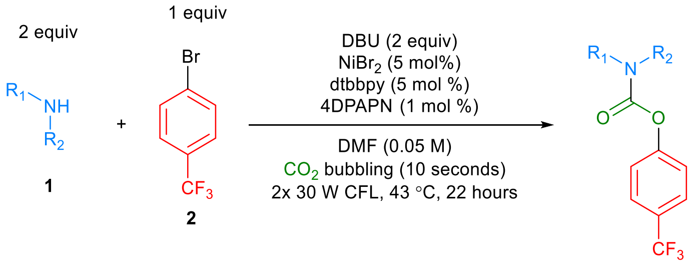

Entry	Amine	Product yield (%)	2 remaining (%)
**1**	Piperidine	35.9	4.6
**2**	Diethylamine	24.5	23.3
**3**	Benzylamine	0	0
**4**	Aniline	0	9.7

Conditions: In an 8 ml vial was added 0.1 ml of a 1:1 $NiBr_2$:dtbbpy solution (0.1 M in DMF, 0.01 mmol, 5 mol %), 0.1 ml of a 4DPAPN solution (0.02 M in DMF, 2 µmol, 1 mol %), DMF (3 ml), 4-bromobenzotrifluoride (28.0 µl, 0.2 mmol, 0.05 M), amine (0.4 mmol, 2 equiv) and DBU (59.7 µl, 0.4 mmol, 2 equiv). The solution was diluted to 4 ml and the vial was capped with a septum cap. CO_2 was bubbled for 10 seconds after which the vial was sealed with parafilm. The vial was placed between two 30 W white CFL lights and stirred vigorously (1200 rpm) at 43 °C for 22 hours. Yields were determined with GC-MS using mesitylene as internal standard.

5.9 MISCALLENOUS OPTIMIZATION EXPERIMENTS

It was recognized that the poor yields were probably due to a competing dehalogenation reaction that is independent of the nickel cycle (Figure S4).[8] In the next optimization attempts we tried to eliminate this side reaction (Table S7).

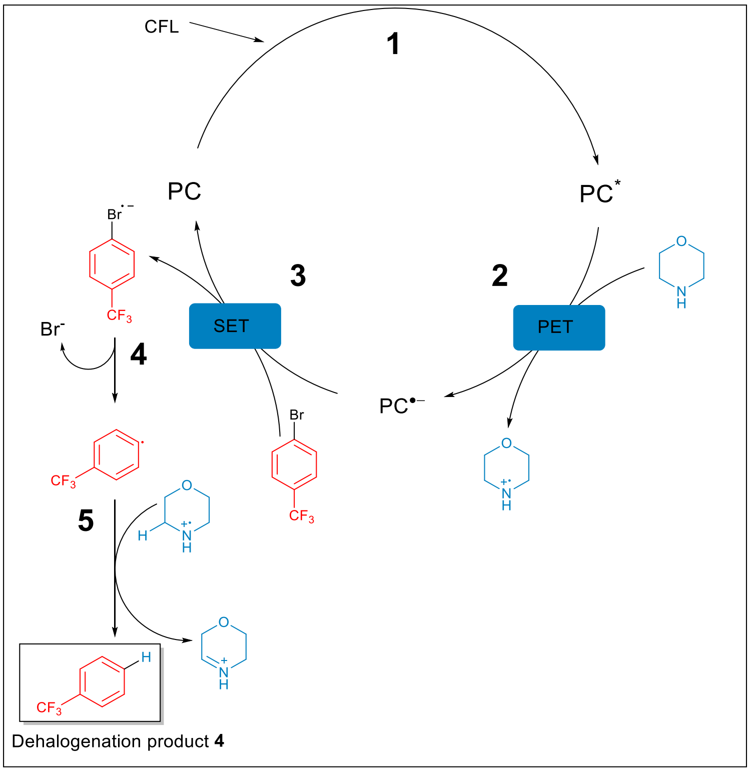

Figure S4. Possible mechanism for the formation of the dehalogenated product **4**.[8] 1 PC is excited with light. 2 reductant, morpholine (or base), reduces the excited PC in a PET step and is oxidized to morpholine●+. 3 the reduced PC acts as a powerful reductant and transfers an electron to the halide in an SET step. 4 the aryl halide radical dissociates forming a halide anion and an aryl radical. 5 the aryl radical abstracts a hydrogen atom from morpholine●+ forming the dehalogenation product.

Table S7. Miscellaneous optimization experiments.

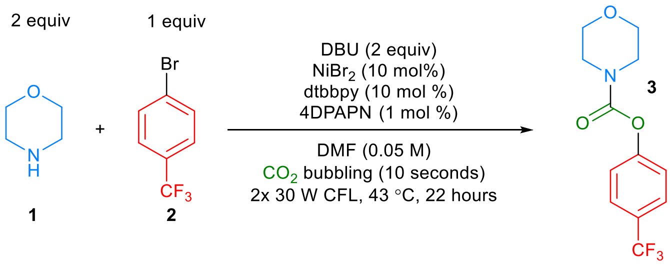

**Entry**	**Variation to the method**	**Yield of 3 (%)**	**2 remaining (%)**
**1**	Cs_2CO_3, no morpholine	0.0	100
**2**	DBU, no morpholine	0.0	73.8
**3**	1 equiv of **1** and DBU, 2 equiv of **2**	59.3 (average of two runs)	98.1
**4**[a]	Slow addition of Morpholine-DBU	59.5	0

	solution (2 equiv) over 10 hours		
**6**[a]	Slow addition of Morpholine-DBU solution (1 equiv) over 10 hours	51.8	0
**7**	83 °C	≈4	0
**8**	51 °C	48.4	0

Conditions: In an 8 ml vial were added 0.1 ml of a 1:1 $NiBr_2$:dtbbpy solution (0.1 M in DMF, 0.01 mmol, 5 mol %), 0.1 ml of a 4DPAPN solution (0.02 M in DMF, 2 μmol, 1 mol %), DMF (3 ml), 4-bromobenzotrifluoride (28.0 μl, 0.2 mmol, 0.05 M), morpholine (34.8 μl, 0.4 mmol, 2 equiv) and base (0.4 mmol, 2 equiv). The solution was diluted to 4 ml and the vial was capped with a septum cap. CO_2 was bubbled for 10 seconds after which the vial was sealed with parafilm. The vial was placed between two 30 W white CFL lights and stirred vigorously (1200 rpm) at 43 °C for 22 hours. Yields were determined with GC-MS using mesitylene as internal standard.[a] Morpholine and DBU were dissolved in 1 ml of DMF and added with a syringe pump over 10 hours to a 3 ml solution of 4-bromobenzotrifluoride sparged with CO_2.

First, we tested that DBU can cause the dehalogenation but Cs_2CO_3 does not. This makes sense since carbonate-ion is not easily oxidized. However, as is evident from the base optimization (Table S5), Cs_2CO_3 gives no advantage over DBU (Table S5, compare entries **2-3** with **7-8**). Therefore, morpholine is most likely oxidized much more easily than DBU or Cs_2CO_3, making it the principle reductant. This also implies that the amine bases like Et_3N performed poorly because they could reduce **2** more readily than morpholine.

We hypothesized that it would be beneficial to add the carbamate to the reaction solution slowly so that its concentration would remain low during the course of the reaction and reduce the unwanted dehydrohalogenation. Unfortunately, similar results were obtained than without slow addition. Finally, we increased the temperature to see if the coupling reaction would be accelerated with respect to the dehalogenation. Unfortunately, increase from 43 °C to 51 °C was already detrimental to the yields and presumably high amounts of dehaloganated species **4** was formed.

5.10 Replication of published methods

Since the catalytic protocol was not working very well, we replicated a couple published methods to make sure that our equipment was not to blame (Table S8).[6, 9] Comparable yields were achieved even though the published methods were slightly modified due to shortage of some chemicals (entries **1** and **2**). We also tested if the published amination method could be directly applied to carbamates but no coupling was observed (entry **3**).[9]

Table S8. Replication of published methods using our dual CFL photoreactor.

Entry 1

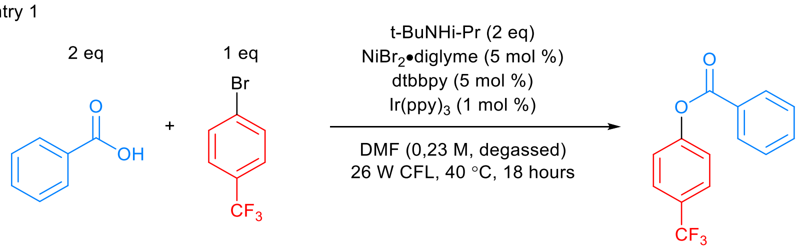

Entry 2

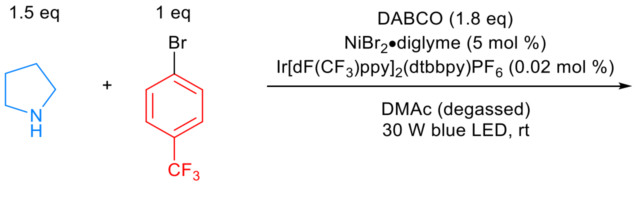

**Entry**	**Reference**	**Changes to the method**[a]	**Yield of reference (isolated)**	**Own yield (^{19}F NMR)**
**1**	28	With our photoreactor, $NiBr_2·3H_2O$, no sonication, degassed by bubbling with Ar (vs freeze, pump, thaw)	86	88.6
**2**	31	With our photoreactor, $NiBr_2·3H_2O$, $Ir(ppy)_3$, no sonication, degassed by bubbling with Ar (vs freeze, pump, thaw)	96	95.3
**3**	-	As in entry 2, but with morpholine and CO_2	-	Only starting material

[a] Refer to the references for further information on the reaction conditions. Yields were determined with ^{19}F NMR using trifluoroethanol as internal standard.

5.11 Testing of the modified 4DPAPN-like catalysts

To decrease the formation of the dehalogenated product **4**, a set of donor-acceptor 4DPAPN-like phthalo-, isophtalo-, and terephtalonitrile-based photocatalyst with electron donating groups were synthetized (Figure S5). The idea was to reduce the oxidizing power of the photocatalyst to suppress oxidation of the amine/carbamate and consequently reduce the unwanted dehalogenation reaction.[7, 10] We also tested a phenoxazine based PC. The optimization results are shown in Table S9.

	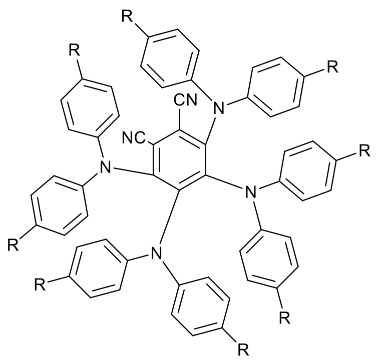	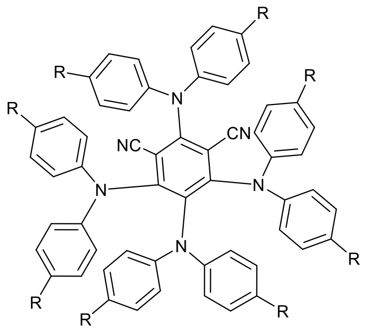	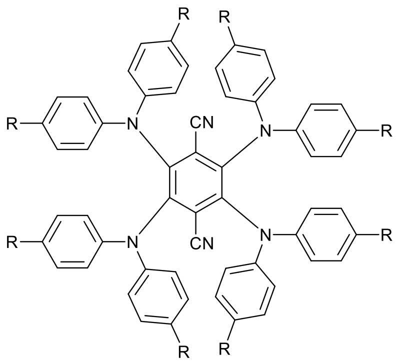
R = H	4DPAPN	4DPAIPN	4DPATPN
R = OMe	4DPAPN-OMe	4DPAIPN-OMe	4DPATPN-OMe
R = ^{t}Bu	4DPAPN-^{t}Bu	4DPAIPN-^{t}Bu	4DPATPN- ^{t}Bu
R = Ph	4DPAPN-Ph	4DPAIPN-Ph	4DPATPN-Ph

Figure S5. Synthetized donor-acceptor photocatalysts based on 4DPAPN.

As hoped, some of the new photocatalysts were more effective than 4DPAPN. Both 4DPAPN-^{t}Bu and 4DPAIPN-^{t}Bu increased the yields about 10 percentage units (entries **5** and **6**). 4DPATPN-^{t}Bu underperformed the latter PC:s probably because its solubility in DMF is poor (entry **7**). 4DPAPN-OMe and 4DPATPN-OMe could not catalyze the coupling reaction (entries **3** and **4**). 4DPAIPN-Ph also worked better than 4DPAPN (entry **11**). Decreasing the catalyst loading of 4DPAPN-^{t}Bu to 0.1 mol % did not decrease the yields significantly (entry **14**) and better yields were observed if given more time to react completely (entry **15**). Further reduction to only 0.02 mol % decreased the reaction rate further (entry **16**). Carbamates of piperidine and ethylmethylamine reacted slower and gave significantly increased amounts of the dehaloganated product **4** as compared to morpholine carbamate (entries **12** and **13**). It was tested that in the absence of $NiBr_2$ and dtbbpy even the improved photocatalyst, 4DPAPN-^{t}Bu, produces significant amounts of **4** over time (entries **14** and **15**).

Table S9. Further optimization of the photocatalyst.

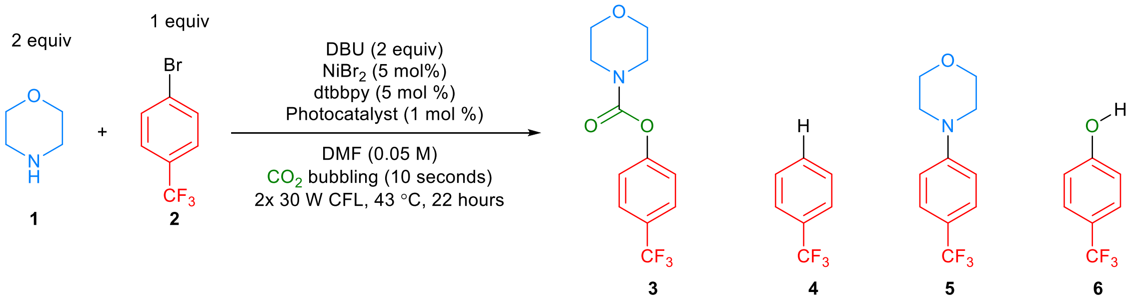

Entry	Photocatalyst	Change	3 (%)	2 remaining (%)	4 (%)	5 + 6 (%)
**1**[a]	4DPAPN	none	57.6	0	no info	no info
**2**	4DPAIPN	none	53.9	0	36.7	9.3
**3**	4DPATPN	none	38.5	trace	56.3	5.5
**4**	4DPAPN-OMe	none	Trace	97.6	0	0
**5**	4DPAIPN-OMe	none	7.3	90.8	0	0.6
**6**[b]	4DPATPN-OMe	none	5.8	97.0	0	0
**7**	4DPAPN-^{t}Bu	none	68.3	6.5	13.8	9.8
**8**	4DPAIPN-^{t}Bu	none	67.7	1.95	18.9	10.2
**9**[c]	4DPATPN-^{t}Bu	none	48.7	28.1	7.9	9.9
**10**	4DPAPN-Ph	none	46.2	29.8	17.1	5.4
**11**	4DPAIPN-Ph	none	63.7	0	24.9	7.7
**12**	4DPATPN-Ph	none	13.2	64.3	18.2	2.6
**13**	Phenoxazine PC	none	19.1	76.9	2.0	0.9
**14**	4DPAPN-^{t}Bu	0.1 mol % PC	62.5	21.2	7.8	7.4
**15**	4DPAPN-^{t}Bu	0.1 mol % PC, 40 hours	71.0	3.1	15.6	10.7
**16**	4DPAPN-^{t}Bu	0.02 mol % PC, 40 hours	42.2	50.2	5.8	7.4
**17**	4DPAPN-^{t}Bu	Piperidine instead of morpholine	43.2	30.1	28.8	5.0
**18**	4DPAPN-^{t}Bu	Ethylmethylamine instead of morpholine	28.5	35.3	30.5	6.9
**19**	4DPAPN-^{t}Bu	No Ni or ligand	0	91.6	10.1	0
**20**	4DPAPN-^{t}Bu	No Ni or ligand, 1 mol % PC, 72 h	0	27.9	71.5	0

Conditions: In an 8 ml vial were added photocatalyst (as shown) and DMF (3 ml). The mixture was stirred until no solids were present. 0.1 ml of a 1:1 $NiBr_2$:dtbbpy (0.1 M in DMF, 0.01 mmol, 5 mol %) was added followed by 4-bromobenzotrifluoride (28.0 µl, 0.2 mmol, 0.05 M), morpholine (34.8 µl, 0.4 mmol, 2 equiv) and DBU (59.7 µl, 0.4 mmol, 2 equiv). The solution was diluted to 4 ml and the vial was capped with a septum cap. CO_2 was bubbled for 10 seconds after which the vial was sealed with parafilm. The vial was placed between two 30 W white CFL lights and stirred vigorously (1200 rpm) at 43 °C for 22 hours. Yields were determined with ^{19}F NMR using trifluoroethanol as internal standard. [a]Yields determined by GC-MS using mesitylene as internal standard. [b]Average of two runs. [c]PC did not dissolve completely.

6 OPTIMIZATION WITH AN ARYL IODIDE COUPLING PARTNER

Since the formation of the dehalogenation product **4** is independent from the Ni catalyst (Figure S4), the rate of the Ni-catalyzed cross-coupling should be increased to decrease its formation. Iodides are known to react faster than bromides in dual nickel/photocatalyst protocols, so 4-bromobenzotrifluoride was replaced with 4-iodobenzotrifluoride in the optimization. The concentration was also increased from 0.05 M to 0.1 M to better match the concentration needed for synthesis.

6.1 IODIDES VS BROMIDES AND OPTIMIZATION OF THE BASE

Table S10: Comparison of Bromobenzotrifluoride and Iodobenzotrifluoride with different amines.

Entry	Amine	Carbamate (%)	CF_3PhBr (%)	CF_3Ph (%)	Aniline + phenol (%)	Comparison to Aryl iodide (%)
**1**	Morpholine	68.3	6.5	13.8	9.8	**78**
**2**	Piperidine	41.0 (49.4) [b]	41.8	12.4	3.5	**81**
**3**	EtMeNH	**77.0**	0	15.1	1.6	55
**4**	i-Pr_2NH	30.1	7.1	31.4	8.2	**47**
**5**	Bn_2NH	73.7	0	12.0	8.4	**85**
**6**	Proline	2.8 (7.8) [b]	93.3	2.2	0.4	**72**
**7**	Desloratadin	**38.9** (40.3)[b]	52.4	5.1	2.1	37
**8**	N-Pyridyl piperazine	26.0 (23.7) [b]	48.1	9.9	10.9	**62**
**9**	N-Benzyl piperazine	32.0 (39.6)[b]	59.5	6.5	0	**77**
**10**	Piperazinone	3.5 (10.9)[b]	83.4	2.1	2.8	**36**

Conditions: In an 8 ml vial, 0.5 ml of a 1:1 $NiBr_2$:dtbbpy solution (0.1 M in DMF, 0.05 mmol, 5 mol %), 0.5 ml of a 4DPAPN-^{t}Bu solution (0.002 M in DMF, 1.0 μmol, 0.1 mol %), DMF (3 ml), 4-Bromobenzotrifluoride (140.0 μl, 1.0 mmol, 0.1 M), amine (2.0 mmol, 2 equiv) and TMG (251 μl 2.0 mmol, 2 equiv). Solution was diluted to 4 ml and the vial was capped with a septum cap. CO_2 was bubbled for 10 seconds after which the vial was sealed with parafilm. The vial was placed between two 30 W white CFL lights and stirred vigorously (1200 rpm) at 43 °C for 22 hours. Yields were determined using C_6F_6 as standard. a)1.1 mmol of amine and TMG instead. b) ^{19}F-NMR yield after 46 hours in brackets

Switching from 4-bromobenzotrifluoride **2** to 4-iodobenzotrifluoride **7** did indeed substantially improve the reaction (Table S10). By using 0.1 mol % loading of 4DPAPN-^{t}Bu, reactions with **7** were complete after the 22 hour period (Table S11, entries **1** and **2**) whereas coupling with **2** was incomplete (entries **3** and **4**). As was hypothesized, both morpholine and piperidine gave less of the dehalogenation product when 4-iodobenzotrifluoride was used (entries **1** and **2** vs **3** and **4**). However, with DBU, both amines showed significant increase of the corresponding directly aminated species

as well as the phenol **6** when using **7** as the aryl halide. When DBU was replaced with TMG (non-contaminated, see section 5.7), this problem was eliminated and yields increased accordingly. Cs_2CO_3 and the more basic version of TMG, 2-cyclohexyl-1,1,3,3-tetramethylguanidine (Cy-TMG), give comparable yield to DBU (entries **5** and **8**).

Table S11. 4-bromobenzotrifluoride vs 4-iodobenzotrifluoride and optimization of the base.

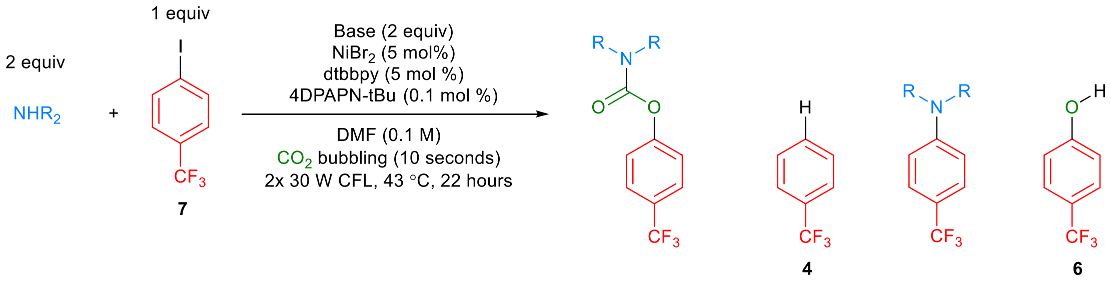

**Entry**	**Amine**	**Base**	**Change**	**Carbamate (%)**	**7 or 2 (%)**	**4 (%)**	**Aniline (%)**	**6 (%)**
**1**	Morpholine	DBU		78.6	0	6.9	11.0	6.9
**2**	Piperidine	DBU		66.7	0	19.1	11.5	3.5
**3**[a]	Morpholine	DBU	**2** instead of **7**	63.1	21.5	8.1	4.9	2.9
**4**[a]	Piperidine	DBU	**2** instead of **7**	40.5	29.8	25.4	2.8	1.8
**5**	Piperidine	Cs_2CO_3		67.4	7.4	13.3	Trace	7.8
**6**	Morpholine	TMG		85.3	0	5.9	4.9	4.0
**7**	Piperidine	TMG		84.0	1.2	13.8	3.1	1.9
**8**	Piperidine	TMG	3 equiv base	81.0	0	14.9	3.9	3.3
**9**	Piperidine	DIPA	2	43.2	43.8	6.4	1.6	2.6
**10**	Ethylmethylamine	TMG		83.9	0	10.1	7.2	trace
	Piperidine	Cy-TMG		68.4	0.7	28.5	4.8	1.6
**11**	Piperidine	DIPA	2	43.2	43.8	6.4	1.6	2.6
**12**	Piperidine	DBU	$PhCF_3Br$ + 15 mol % TBAI	30.0	50.6	15.0	3.1	1.1
**13**	Piperidine	DBU	15 mol % of CuI + KI	0	101.7	0	0	0

Conditions: In an 8 ml vial were added 0.2 ml of a 1:1 $NiBr_2$:dtbbpy solution (0.1 M in DMF, 0.02 mmol, 5 mol %), 0.2 ml of a 4DPAPN-^{t}Bu solution (0.002 M in DMF, 0.4 µmol, 0.1 mol %), DMF (3 ml), 4-iodobenzotrifluoride (58.8 µl, 0.4 mmol, 0.1 M), amine (0.8 mmol, 2 equiv) and a base (0.8 mmol, 2 equiv). Solution was diluted to 4 ml and the vial was capped with a septum cap. CO_2 was bubbled for 10 seconds after which the vial was sealed with parafilm. The vial was placed between two 30 W white CFL lights and stirred vigorously (1200 rpm) at 43 °C for 22 hours. [a] Same method but 0.05 M instead of 0.1 M. Yields were determined with ^{19}F NMR using trifluoroethanol as internal standard.

We tested that the increased yields when using TMG as the base are also observed when a non-cyclic secondary amine, ethylmethylamine, is used. Before discovering TMG as the optimal base, we also unsuccessfully tried to use **2** with TBAI (tetrabutylammonium iodide) to get the benefits of increased reaction rate without using the more expensive aryl iodide. In a similar attempt, CuI with KI was used. The idea was that Cu would enable the replacement of Br with I to form the more reactive aryl iodide, which unfortunately turned out not to be the case.

6.2 OPTIMIZATION OF THE OTHER COMPONENTS TO ARRIVE AT THE OPTIMAL CONDITIONS

Lastly, a quick optimization of the other components of the coupling protocol was conducted to make sure that all other components were still optimal after switching from aryl bromides to iodides. In these optimization experiments piperidine was used as it produces more dehalogenated side-product and could therefore give more pronounced differences between the components. As can be seen from Table S12, no further changes were necessary.

Table S12. Optimization of the other components of the coupling protocol.

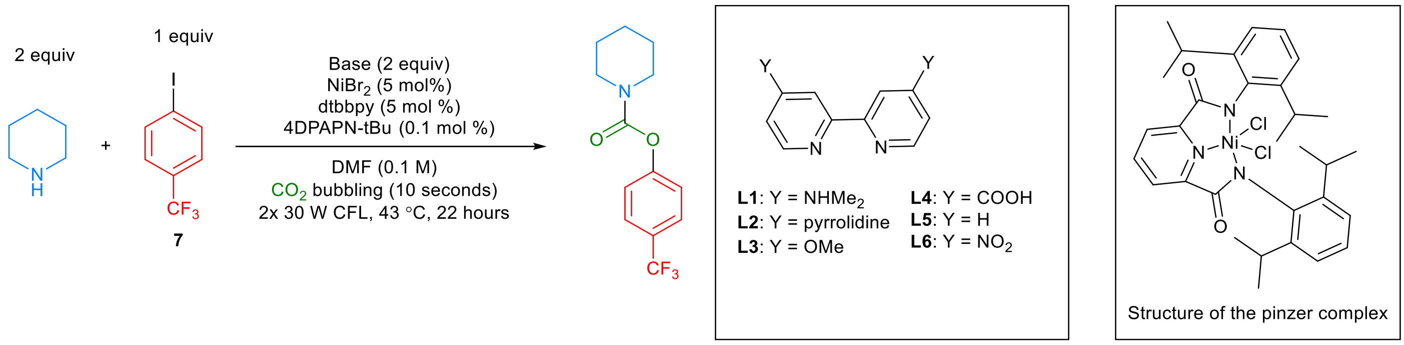

Entry	Change	Carbamate (%)	Iodobenzo-trifuoride (%)	Dehalogenated (%)	Aniline (%)	Phenol (%)
**1**	None	84.0	1.2	13.8	3.1	1.9
**2**	DMAc as the solvent	83.3	trace	13.3	3.9	2.0
**3**	DMSO as the solvent	68.8	0	22.3	6.7	1.7
**4**	NMP as the solvent	70.6	15.9	9.9	2.0	1.0
**5**	No PC	trace	100	0	0	0
**6**	4DPAIPN-^{t}Bu as PC	79.1	0	13.9	4.3	2.2
**7**	Ir(ppy)$_3$ as PC	71.4	6.1	18.3	2.1	2.2
**8**	No dtbbpy	1.2	67.1	26.5	3.1	0
**9**	2x dtbbpy	44.5	49.0	2.8	1.2	2.5
**10**	**L1** as ligand	17.5	58.5	18.9	1.0	1.5
**11**	**L2** as ligand	11.0	61.8	26.1	0.9	2.0

**12**	**L3** as ligand	69.4	4.2	16.0	3.4	2.9
**13**	**L4** as ligand	60.8	18.8	13.1	3.8	3.1
**14**	**L5** as ligand	49.1	33.9	10.8	2.7	0.8
**15**	**L6** as ligand	9.1	61.1	34.8	0.6	0.2
**16**	No Ni or dtbbpy	0	32.3	69.1	0	0
**17**	$NiBr_2 \cdot 3H_2O$ as nickel source	76.4	3.2	11.8	3.5	3.1
**18**	Pinzer complex as the catalyst	0	99.7	0	0	0
**19**	Added 0.4 µmol H_2O	66.1	5.5	7.5	5.4	15.1
**20**	Added 0.4 µmol D_2O	70.7	0	7.5	5.1	16.7
**21**	Added 9 ml O_2 after CO_2 flush	69.9	2.6	9.3	3.9	14.2

Conditions: In a 8 ml vial was added 0.2 ml of a 1:1 $NiBr_2$:dtbbpy solution (0.1 M in DMF, 0.02 mmol, 5 mol %), 0.2 ml of a 4DPAPN-^{t}Bu solution (0.002 M in DMF, 0.4 µmol, 0.1 mol %), DMF (3 ml), 4-iodobenzotrifluoride (58.8 µl, 0.4 mmol, 0.1 M), piperidine (39.5 µl, 0.8 mmol, 2 equiv) and TMG (100.4 µl, 0.8 mmol, 2 equiv). The solution was diluted to 4 ml and the vial was capped with a septum cap. CO_2 was bubbled for 10 seconds after which the vial was sealed with parafilm. The vial was placed between two 30 W white CFL lights and stirred vigorously (1200 rpm) at 43 °C for 22 hours. Yields were determined with ^{19}F NMR using trifluoroethanol as internal standard. [a]Same method but 0.05 M instead of 0.1 M.

In a 2020 study it was discovered that increasing the donor ability of the substituent at the 4,4'-position of a 2,2'-bipyridine-based ligand resulted in increased yields (with alcohols).[5] Unfortunately, no such effect was observed with our protocol and both strongly donating ($NHMe_2$ and pyrrolidine, entries **11** and **12**) and strongly withdrawing (NO_2, entry **16)** substituents underperformed the optimal ^{t}Bu substituent. The reaction has a moderate sensitivity towards water and oxygen (entries **19-21**) as they increase the formation of the corresponding phenol.

6.3 Effect of concentration

For the actual synthesis, a more concentrated solution is beneficial to allow a higher volume of synthesis with the limited space of our photoreactors. This is why we scoped the effect of concentration on the reaction yields. As can be seen in Figure S6, the yields decrease with increasing concentration. This drop in yields is caused by decrease in the overall conversion of the starting material, which is likely caused by poorer light penetration in the more concentrated solutions resulting in slower reaction. For the synthesis of aryl carbamates, a concentration of 0.25 M was chosen because it allowed us to synthetize 3 compounds at once in 2 mmol scale with an acceptable drop in reaction rate.

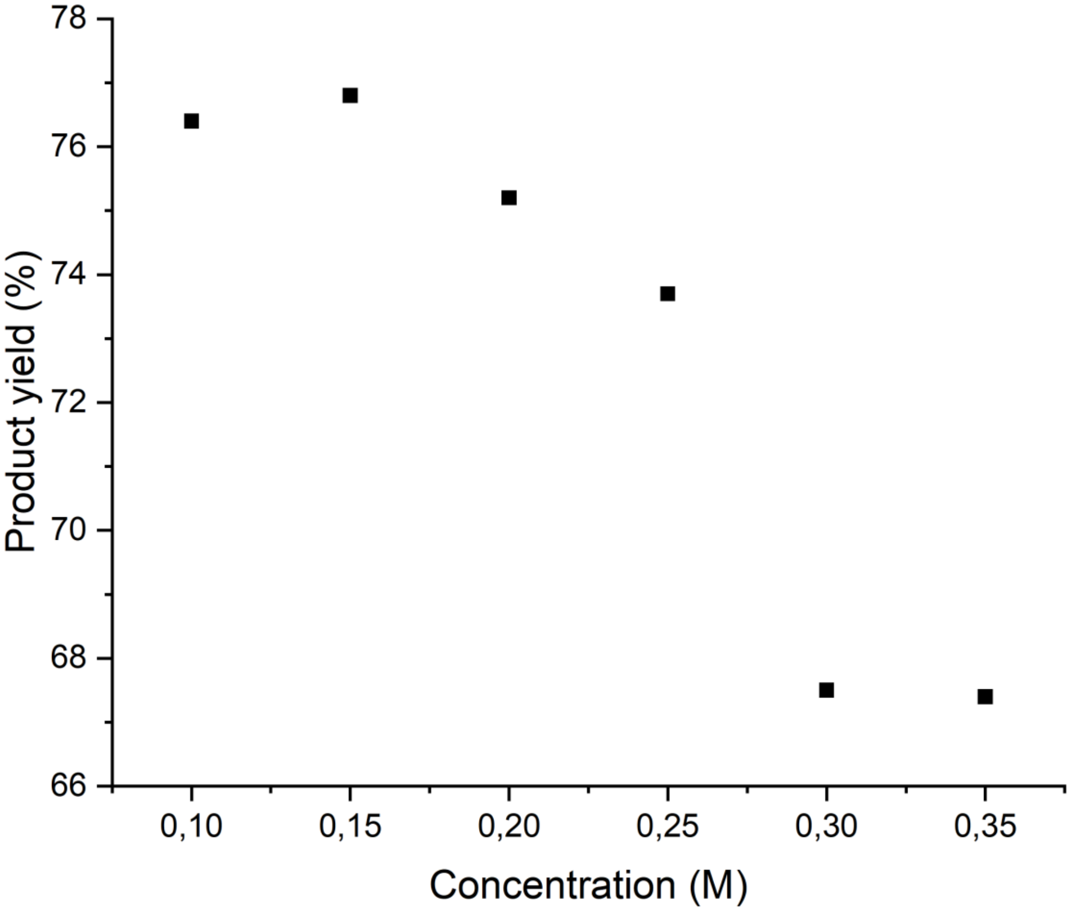

Figure S6. Effect of concentration on the reaction yield after 15 hours. **Conditions:** Optimal coupling protocol (see Table S12). 4-iodobenzotrifluoride (0.4-1.4 mmol, 0.1-0.35 M), piperidine (2 equiv) and TMG (2 equiv), 1:1 $NiBr_2$:dtbbpy (5 mol %), 4DPAPN-^{t}Bu (0.1 mol %). Yields were determined with ^{19}F NMR using trifluoroethanol as internal standard.

6.4 TABLE 1 AND 2 SIDE PRODUCT DISTRIBUTION

Table S13: Deviation from optimized conditions with side product distribution determined by ^{19}F-NMR with hexafluorobenzene as internal standard. Reaction conditions; 0.4 mmol iodobenzotrifluoride, 0.8 mmol TMG, 0.8 mmol morpholine, 0.02 mmol $NiBr_2$dtbbpy, 0.004 mmol DPAPN in DMF. Flushed with CO_2. 2x30W CFL, 22 hours, 43 °C,

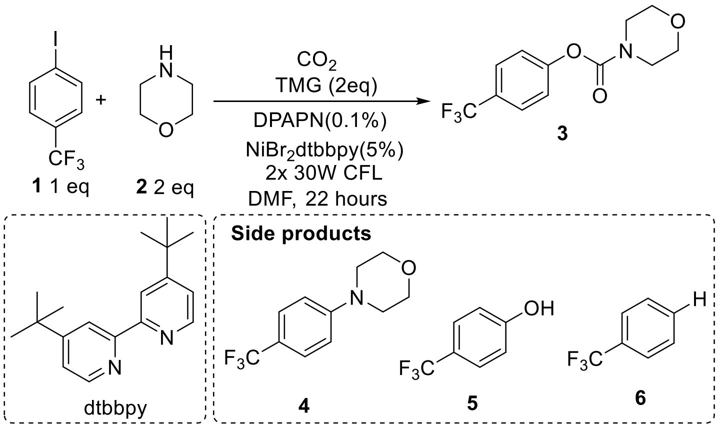

#	Deviation from above	Carbamate **3**	Iodobenzo-trifluoride **1**	Dehalo-genated **6**	Aniline **4**	Phenol **5**
**1**	None	78 %	0 %	16 %	3 %	3 %
**2**	No nickel	0 %	37 %	62 %	0 %	0 %
**3**	No photocatalyst	<1 %	99 %	< 1 %	0 %	0 %
**4**	Dark	0 %	100 %	0 %	0 %	0 %
**5**	Blue led	66 %	0 %	8 %	6 %	20 %
**6**	bipy instead of dtbbpy	28 %	15 %	5 %	35 %	18 %
**7**	1.1 eq morpholine	72 %	7 %	13 %	3 %	5 %
**8**	1.1 eq morpholine and TMG	62 %	23 %	4 %	3 %	6 %
**9**	iPr_2NH as a base	44 %	22 %	21 %	8 %	5 %
**10**	Et_2iPrN as a base	9 %	81 %	3 %	5 %	2 %
**11**	Cs_2CO_3 as a base	77 %	2 %	16 %	3 %	3 %
**12**	DBU as a base	69 %	0 %	20 %	4 %	7 %
**13**	Added 1 eq H_2O	65 %	0 %	19 %	4 %	11 %
**14**	Added 1 eq O_2	68 %	0 %	18 %	4 %	9 %
**15**	Added 1 eq stilbene	76 %	8 %	6 %	3 %	6 %
**16**	$Ir(ppy)_3$ instead of DPAPN	71 %	6 %	18 %	2 %	2 %
**17**	4CzIPN instead of 4DPAPN	62 %	24 %	6 %	3 %	5 %

Table S14: Product distribution with different photocatalysts determined by ^{19}F-NMR with hexafluorobenzene as internal standard. Reaction conditions; 0.4 mmol iodobenzotrifluoride, 0.8 mmol TMG, 0.8 mmol morpholine, 0.02 mmol $NiBr_2$dtbbpy, 0.004 mmol photocatalyst in DMF. Flushed with CO_2. 2x30W CFL, 22 hours, 43 °C,

Photocatalyst	Acronym	Yield	Iodobenzotrifluoride	Dehalogenated	Aniline	Phenol
**1a**	4DPAPN	78 %	0 %	16 %	3 %	3 %
**2a**	4DPAIPN	72 %	0 %	19 %	3 %	5 %
**3a**	4DPATPN	59 %	0 %	30 %	4 %	7 %
**1b**	4DPAPN-OMe	0 %	100 %	0 %	0 %	0 %
**2b**	4DPAIPN-OMe	16 %	80 %	2 %	1 %	2 %
**3b**	4DPATPN-OMe	8 %	89 %	2 %	1 %	1 %
**1c**	4DPAPN-^{t}Bu	87 %	0 %	5 %	5 %	4 %
**2c**	4DPAIPN-^{t}Bu	79 %	0 %	14 %	43 %	2 %
**3c**	4DPATPN-^{t}Bu	50 %	41 %	3 %	2 %	3 %
**1d**	4DPAPN-Ph	41 %	30 %	25 %	3 %	1 %
**2d**	4DPAIPN-Ph	72 %	0 %	25 %	4 %	1 %
**3d**	4DPATPN-Ph	19 %	72 %	7 %	<1%	<1%

7 Unsuccessful substrates

7.1 List of unsuccessful substrates

Unfortunately, many of the tested substrates did not give the desired coupling product or did not couple at all. Iodobenzenes with electron donating substituents are too unreactive and only give trace amounts of coupling products (desired and directly aminated) even with extended reaction times (**19-21**). While slightly adjusting the conditions allowed us to couple various iodopyridines, other tested heterocycles failed to react under the standard or modified conditions.

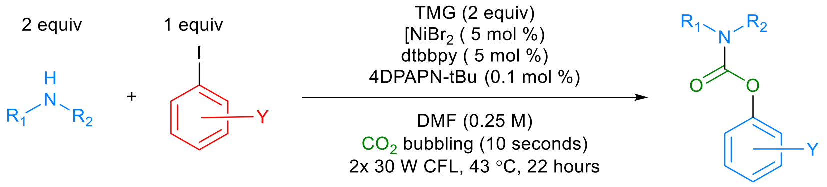

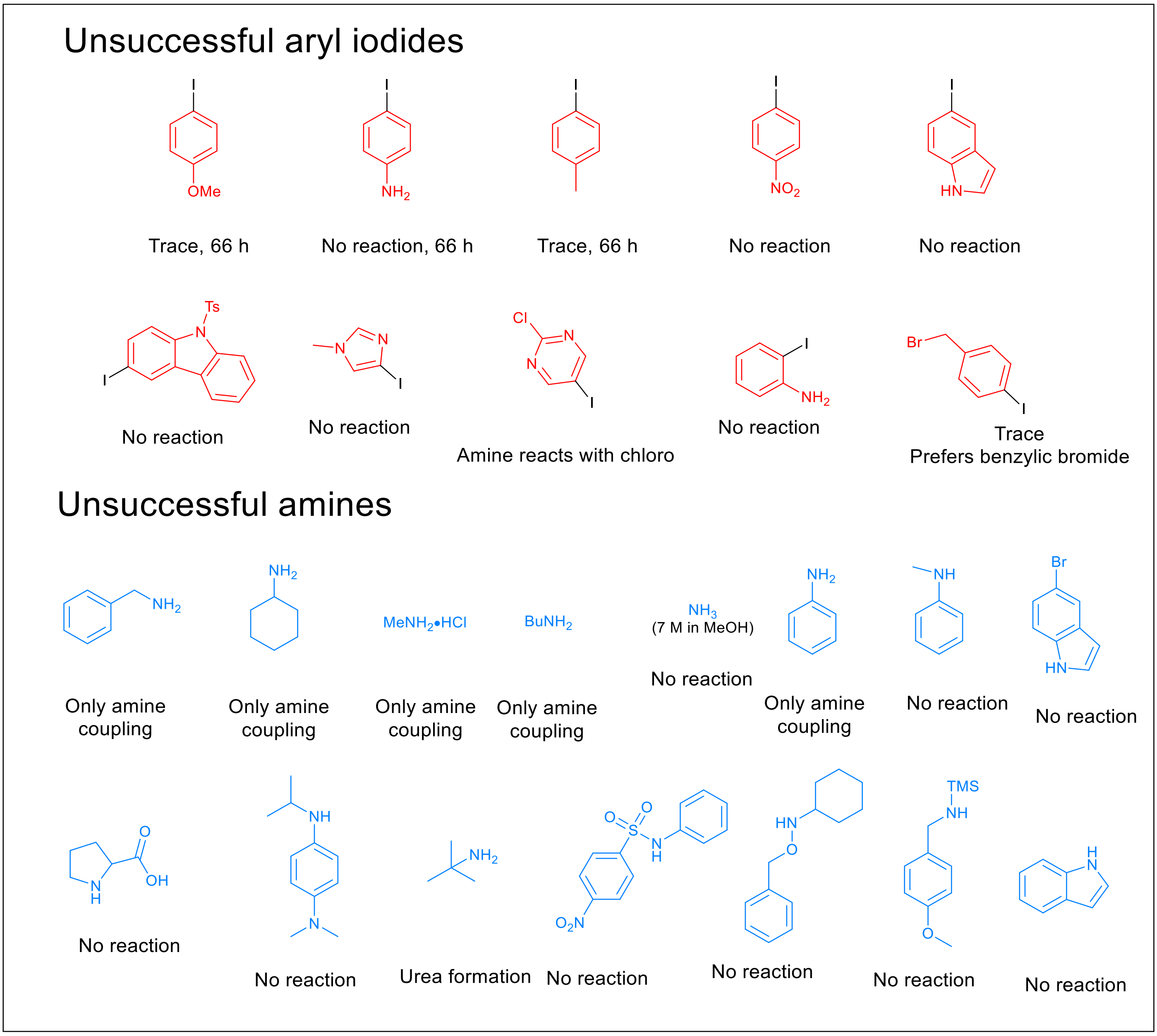

Figure S7. Unsuccessful substrates. Morpholine was used as the amine with the aryl iodides and 4-iodobentsotrifluoride was used as the aryl iodide with the amines. Optimal coupling protocol (see Table S12). 4-iodobenzotrifluoride (1 mmol, 1 eq) was used for coupling with the amines and morpholine (2 mmol, 2 eq) for coupling with the aryl iodides.

While most secondary amines work well, some are too unreactive. These include amino acids (proline gives neither carbamate, ester or amine coupling product) and aromatic secondary amines (**29-31**), which slowly react to give directly aminated product (carbamate formation is incomplete). Ammonia was too unreactive to give neither coupling product (**27**).

Primary amines only produce directly aminated coupling products and do not give any of the desired O-aryl carbamate (**24-26** and **28**). This behavior was also observed with copper catalyzed coupling of carbamates and arylboronic acids.[11] We believe that with primary amines the formed carbamate is more reactive from the N-side than from the O⁻-side and therefore only aminated product is observed as is illustrated in Figure S8. Reactivity on the nitrogen is supported by oxazolidinones forming N-coupled product in the reaction conditions. Additionally, we tested alternative hypothesis that the primary amine product decomposes in the reaction conditions. However, 4-(trifluoromethyl)phenyl butylcarbamate does not dissociate in the reaction conditions (Table S15) in meaningful amounts.

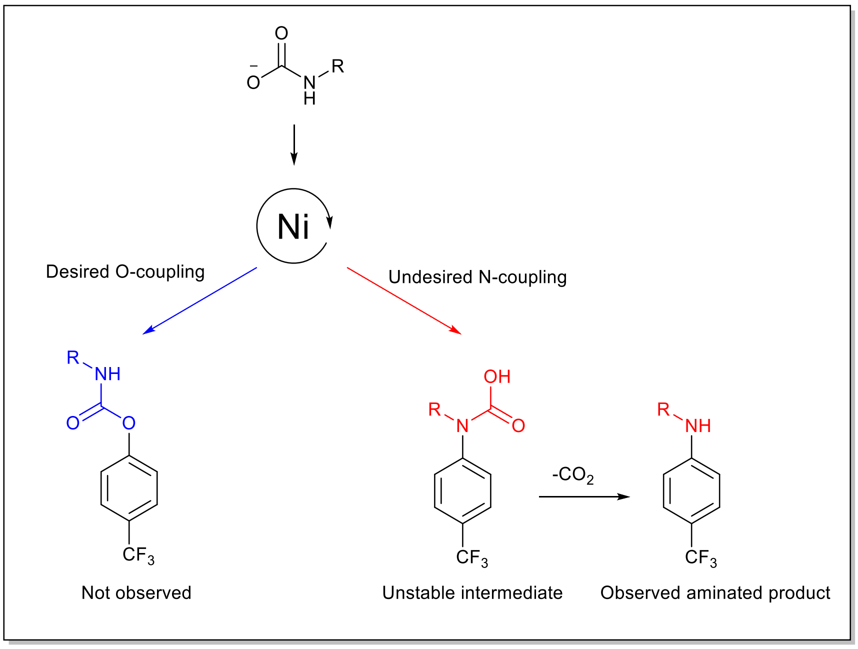

Figure S8. The hypothesized mechanism of formation of directly aminated species when primary amines are used to form the carbamate.

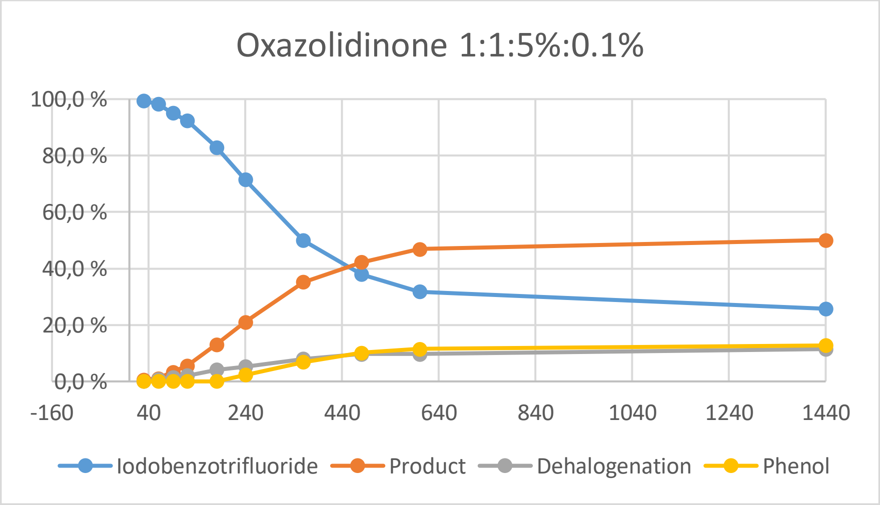

50

Figure S 9: Reaction with iodobenzotrifluoride and oxazolidinone. 0.2 mmol oxazolidinone, 0.2 mmol TMG, 0.2 mmol iodobenzotrifluoride, 0.01 mmol $NiBr_2dtbbpy$, 0.2 µmol DPAPN-tbu, 0.2 mmol Et_3N (to reduce nickel), diluted to 7 ml in DMF.

Table S15. Subjecting a primary amine to the reaction conditions.

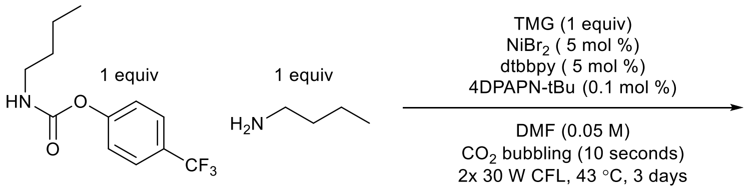

Conditions	Results
As shown	No conversion
No butyl amine	No conversion
No butylamine or TMG	3 % conversion[a]
Flushed with Ar instead of CO_2	16 % conversion[b]

Conditions: 4-(trifluoromethyl)phenyl butylcarbamate (0.2 mmol, 52.2 mg) was mixed with butyl amine (0.2 mmol), TMG (0.2 mmol), $NiBr_2$dtbbpy (0.01mmol) , DPAIPN-^{t}Bu (0.0002 mmol) in DMF (4 ml) and mixture was flushed with CO2. The mixture was irradiated with 2x 30W CFL for 3 days. Trifluoroethanol (0.2 mmol) was added to the mixture as a reference and 19F NMR was measured. a) ^{19}F-NMR for product is -61.6 ppm, which matches corresponding aniline. b) 13 % ^{19}F-NMR -61.6 ppm, which matches corresponding aniline, 3% -76.6 ppm unknown compound.

7.2 Attempts to make primary amines react: coupling with dicarbamate species

To achieve reactivity with primary amines, the amine should be protected to avoid the coupling from the N-side. We postulated that the simplest way to achieve this is to add more than two equivalents of TMG as opposed to the amine to form a dicarbamate species (Table S16).[3] Upon bubbling of CO_2 into the reaction solution, precipitation was observed and the magnet was jammed. After a couple minutes of irradiation in the photoreactor, the mixture started to liquefy and the magnet started spinning again. After the reaction period, undissolved salt was still present. Unfortunately, only aminated species was observed by ^{19}F NMR. It seems that the formation of the dicarbamate species was successful (with 1:1 TMG:amine no precipitation is observed) but it was too unreactive and only aminated coupling product, formed by coupling of the "normal" carbamate as shown in Figure S8, was observed.

Table S16. Cross-coupling with high excess of TMG to form dicarbamate species that could possibly act as a easily removable protecting group.

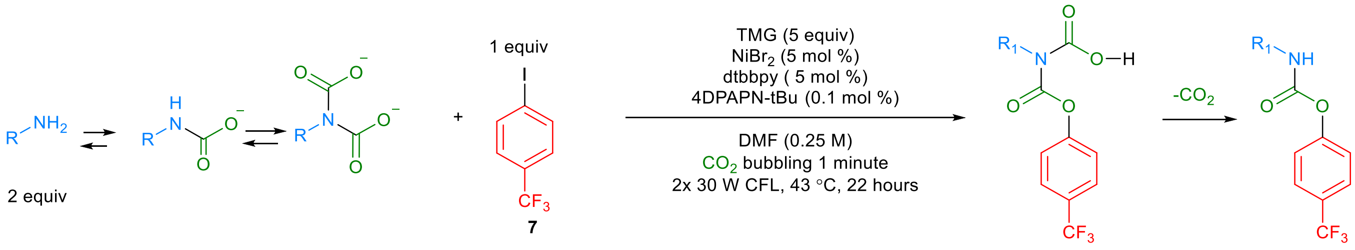

Entry	Amine	Base	Carbamate	$PhCF_3I$	$PhCF_3$	N-arylated	Phenol
1	4-MeOBenzylamine	TMG 2 eq	0	2	10	41	43
2	4-MeOBenzylamine	TMG 5 eq	0	0	23	53	7
3	4-MeOBenzylamine	TMG 20 eq[a]	0	29	4	48	4
4	4-MeOBenzylamine	BuLi 2 eq[b]	0	86	3	5	2
5	4-MeOBenzylamine	BuLi 4 eq[c]	0	92	2	1	2
6	4-MeOBenzylamine	Cs_2CO_3 5 eq	0	95	4	<1	<1
7	4-MeOBenzylamine	Cs_2CO_3 5 eq[d]	0	0	34	31	<1
8	Aniline	TMG 5 eq	0	28	4	71	0

Optimal coupling protocol (see section Table S12). 4-iodobenzotrifluoride (0.4 mmol), amine (0.8 mmol, 2 equiv) and TMG (2 mmol, 5 equiv), 1:1 $NiBr_2$:dtbbpy (5 mol %), 4DPAPN-^{t}Bu (0.1 mol %). a) Partially insoluable, which may explain incomplete reactivity. b) Lithium carbamate was made by mixing amine and equivalent amount of BuLi in THF, then adding CO_2 and evaporated. Resulting crude solid was used directly in the reaction c) Lithium carbamate was made by mixing amine and equivalent amount of BuLi in THF, then adding CO_2 and evaporated. Resulting solid was redissolved in THF and another equivalent amount of BuLi was added, followed by CO_2. Mixture was evaporated and the remaining solid was directly used in the reaction. d) Added 0.2 eq Et_3N

8 EXPERIMENTS WITH ZINC AS A REDUCTANT

If the mechanism of the reaction follows the thermal Ni(I)/Ni(III) pathway, using some other form of reduction to reduce Ni(II) to Ni(I) should allow access to this cycle. This was demonstrated by Sung, Qin and Nocera who were able to efficiently couple amines, alcohols and carboxylic acids by using zinc as the reductant.[12] With their coupling protocols, yields up to 95 % were recovered after 18-hour reaction time at room temperature or 40-60 °C. We sought to replicate this with the carbamate coupling partner.

Table S17. Cross-coupling using zinc as a reductant to access the thermal Ni(I)/Ni(III) cycle.

Entry	Zn equiv reaction time	Product	$PhCF_3I$	$PhCF_3$	Amine and phenol	$PhCF_3COOH$[a]
1	0 eq., 5 days	0	98.8	0	0	0
2	0.5 eq., 22 hours	0.7	58.9	29.5	0	16.3
3	0.8 eq., 22 hours	8.3	22.7	41.6	0	19.8
4	1.0 eq., 22 hours	11.1	0	76.5	1.3	11.1
5	0.5 eq., 5 days	1.8	54.7	35.7	0	7.6
6	1 eq., 5 days	19.7	0.6	62.6	0.8	16.3
7	0.5eq.,22 hours, in light	38.2	14.4	35.7	1.3	8.6
8	1.0 eq. 22 hours, in light	32.4	0	55.7	0.6	11.2
9	1.5 eq 22 hours, in light	26.4	0	58.2	0.2	13.6

With optimal coupling protocol (see Table S12) using morpholine as the amine without photocatalyst. Zinc dust activated by washing with HCl, drying under vacuum and storing in glove box.[a] Formation confirmed with GC-MSL.

Zinc as the reductant gives poor results with the carbamate coupling partner even when using high zinc loading. This is in contrary to the results of carboxylic acid coupling partner used by Sun, Qin and Nocera.[12] For example the published yield with 0.5 eq of zinc for coupling benzoic acid with 4-bromobenzotrifluoride gave a yield of 67 %. Nevertheless, our results seem to indicate that in the absence of light, zinc can promote the coupling 4-iodobenzotrifluoride with morpholine derived

carbamate (entries **2**-**5**), but with poor selectivity. With light the selectivity is moderately improved. No coupling was observed without zinc (entry **1**).

9 SYNTHESIS OF THE PHOTOCATALYSTS

9.1 GENERAL PROCEDURE

Here the general procedure for the synthesis of photocatalysts at 1 mmol scale is presented. Note that the synthesis of most photocatalyst were scaled down to 0.545, 0.5 or 0.4 mmol by reducing the reagents accordingly. Warning! Mixing NaH with DMSO can lead to a runaway exothermic reaction. Take care that the temperature stays in control by using high stirring speed, using cooling bath and adding NaH in small portions.

In a Shchlenk flask 5,5 mmol of appropriate diphenyl amine (diphenyl amine, Bis(4-methoxyphenyl)amine, 4,4'-Di-tert-butyldiphenylamine or di([1,1'-biphenyl]-4-yl)amine) was dissolved in 10 ml of dry DMSO under argon (deprotonated diphenyl amines are very oxygen sensitive, which is seen by reaction turning green in presence of oxygen). 5 mmol of NaH was added to the Schlenk flask in portions. The mixture was stirred until hydrogen evolution had stopped (usually around 1 hour). Phtalonitrile, isophtalonitrile or terephtalonitrile (1 mmol) was added to the mixture and the mixture was stirred at room temperature for 1 day for H-, ^{t}Bu- and Ph substituted, and 5 days for OMe substituted diphenyl amines. The mixture was poured into 100 ml of H_2O, producing a colored precipitate. The precipitate was filtered and washed with water and then with EtOH, until nothing seemed to dissolve. Further purification, if needed, was done by column chromatography or by recrystallization from DCM/EtOH by slow evaporation.

9.2 SYNTHESIS

4DPAPN (3,4,5,6-tetrakis(diphenylamino)phthalonitrile) (7a)

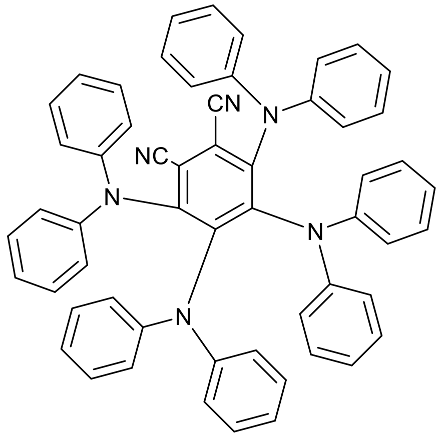

Note! Following the literature method with 40 ml anhydrous THF at room temperature did not yield any product.[7] Increasing the temperature to 60 °C resulted in little product that was not isolated.

In the glove box, in an oven dried 120 ml Schlenk flask were added diphenylamine (464 mg, 2,75 mmol, 5.5 equiv), NaH (66 mg, 2.75 mmol, 5.5 equiv) and DMSO (10 ml). The mixture was stirred in the glove box for about 30 minutes until bubbling had seized. Tetrafluorophthalonitrile (100 mg, 0.5 mmol) was added outside the glove box under argon flow and another 10 ml batch of dry DMSO was used to rinse the sides of the flask. The flask was then put to a 50 °C oil bath and stirred for 18 hours. After this period, the reaction mixture was poured to a beaker of water (about 100 ml) and the precipitated yellow product was filtered and washed with water and ethanol. The crude yellow product was purified by column chromatography with 1:1 DCM:hex. Yield 44 % (183 mg), bright yellow solide. NMR spectra matches the published spectra.[7]

^{1}H NMR (400 MHz, $CDCl_3$) δ 7.18 (t, *J* = 7.9 Hz, 10H), 6.96 (tt, *J* = 7.4, 1.3 Hz, 5H), 6.85 (t, *J* = 7.9 Hz, 10H), 6.77 (dd, *J* = 7.6, 1.1 Hz, 10H), 6.69 (tt, *J* = 7.3, 1.5 Hz, 5H), 6.43 (dd, *J* = 7.6, 1.1 Hz, 10H).

^{13}C{^{1}H} NMR (101 MHz, $CDCl_3$) δ 150.4, 148.0, 144.9, 143.9, 128.9, 127.7, 123.8, 123.5, 122.4, 122.3, 116.9, 113.5.

IR (ATR), cm^{-1}: 3060, 3037, 2227, 1678, 1588, 1489, 1402, 1361, 1321, 1290, 1264, 1247, 1226, 1178, 1154, 1100, 1080, 1029, 924, 890, 838, 773, 741, 692, 624, 605, 572, 509, 492, 429, 408.

HRMS-ESI (pos): $[M+Na]^+$ calculated for $C_{56}H_{40}N_6Na$ 819.3207. Found 819.3185.

4DPAIPN (2,4,5,6-tetrakis(diphenylamino)isophthalonitrile) (8a)

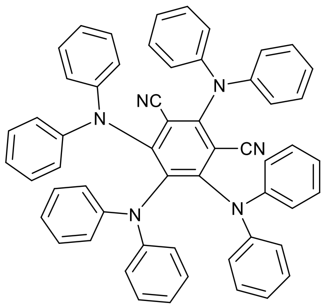

Synthetized with the general procedure at 1 mmol scale. Purified by recrystallizing from DCM/EtOH by slow evaporation. Yield 76 % (587.9 mg), orange crystals.

^{1}H NMR (400 MHz, $CDCl_3$) δ 7.33 – 7.24 (m, 4H), 7.16 – 7.05 (m, 12H), 7.03 (td, *J* = 7.4, 1.2 Hz, 2H), 6.97 – 6.84 (m, 8H), 6.75 – 6.66 (m, 10H), 6.65 – 6.52 (m, 4H).

^{13}C NMR (101 MHz, $CDCl_3$) δ 154.31, 151.86, 145.66, 144.79, 143.28, 140.42, 129.50, 128.72, 127.69, 124.30, 124.08, 123.05, 122.76, 122.72, 121.20, 113.28, 113.15.

IR (ATR), cm^{-1}: 3056, 3033, 3009, 2220, 1588, 1530, 1485, 1418, 1402, 1312, 1268, 1240, 1177, 1155, 1077, 1030, 828, 745, 690, 581, 512, 487, 435, 415.

HRMS-ESI (neg): $[M+HCOO]^-$ calculated for $C_{57}H_{41}N_6O_2$ 841.3291. Found 841.3307.

4DPATPN (2,3,5,6-tetrakis(diphenylamino)terephthalonitrile) (9a)

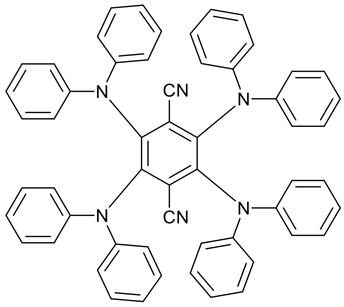

Synthetized with the general procedure at 1 mmol scale. Purified by recrystallizing from DCM/EtOH by slow evaporation. Yield 55 % (440.3 mg), red solid.

^{1}H NMR (400 MHz, $CDCl_3$) δ 7.12 (t, *J* = 7.7 Hz, 16H), 6.89 (t, *J* = 7.4 Hz, 8H), 6.79 (d, *J* = 8.0 Hz, 16H).

^{13}C{^{1}H} NMR (101 MHz, $CDCl_3$) δ 146.0, 144.8, 128.8, 123.6, 122.6, 122.1, 113.3.

IR (ATR), cm^{-1}: 3063, 3037, 2236, 1942, 1586, 1489, 1421, 1273, 1260, 1222, 1079, 1031, 924, 752, 735, 691, 653, 598, 584, 568, 497, 436, 418.

HRMS-ESI (pos): $[M+Na]^+$ calculated for $C_{56}H_{40}N_6Na$ 819.3207. Found 819.3218.

4DPAPN-OMe (3,4,5,6-tetrakis(bis(4-methoxyphenyl)amino)phthalonitrile) (7b)

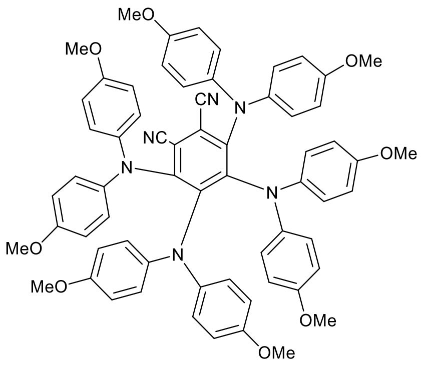

Synthetized with the general procedure at 0.4 mmol scale. Isolated as brown-red solid. Purified by recrystallizing from DCM/EtOH by slow evaporation. Yield 42 % (173 mg).

^{1}H NMR (400 MHz, $CDCl_3$) δ 6.74 – 6.61 (m, 16H), 6.45 – 6.31 (m, 16H), 3.74 (s, 12H), 3.60 (s, 12H).

^{13}C{^{1}H} NMR (101 MHz, $CDCl_3$) δ 155.7, 155.5, 149.5, 147.5, 139.3, 138.3, 123.5, 123.3, 115.9, 114.1, 114.0, 113.0, 55.6, 55.5.

IR (ATR), cm^{-1}: 3040, 2995, 2933, 2906, 2832, 2223, 1608, 1588, 1498, 1464, 1432, 1397, 1355, 1300, 1281, 1233, 1170, 1109, 1089, 1030, 820, 792, 751, 723, 710, 686, 658, 639, 600, 575, 562, 519, 465, 436, 417

HRMS-ESI (pos): $[M+Na]^+$ calculated for $C_{64}H_{56}N_6O_8Na$ 1059.4052. Found 1059.4086

4DPAIPN-OMe (2,4,5,6-tetrakis(bis(4-methoxyphenyl)amino)isophthalonitrile) (8b)

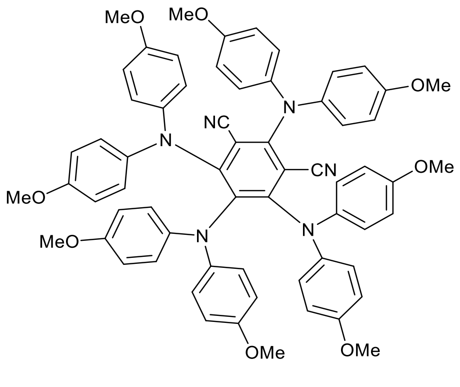

Synthetized with the general procedure at 1 mmol scale. Isolated as bright red solid. Purified by recrystallizing from DCM/EtOH by slow evaporation. Yield 76 % (789 mg).

^{1}H NMR (400 MHz, $CDCl_3$) δ 7.00 – 6.91 (m, 4H), 6.81 – 6.72 (m, 4H), 6.60 (s, 16H), 6.46 (s, 8H), 3.75 (s, 6H), 3.68 (s, 12H), 3.63 (s, 6H).

^{13}C{^{1}H} NMR (101 MHz, $CDCl_3$) δ 156.0, 155.9, 154.7, 154.1, 152.0, 139.7, 139.1, 138.8, 137.6, 124.3, 123.9, 122.1, 114.6, 113.9, 113.9, 112.9, 110.9, 55.5, 55.5, 55.4.

IR (ATR), cm^{-1}: 3056, 2992, 2930, 2909, 2835, 2221, 1608, 1588, 1532, 1501, 1463, 1435, 1409, 1281, 1229, 1177, 1108, 1030, 950, 920, 883, 820, 780, 752, 722, 709, 687, 678, 639, 558, 521, 470, 446, 415

HRMS-ESI (pos): [M+Na]$^+$ calculated for $C_{64}H_{56}N_6O_8Na$ 1059.4052. Found 1059.4027.

4DPATPN-OMe (2,3,5,6-tetrakis(bis(4-methoxyphenyl)amino)terephthalonitrile) (8c)

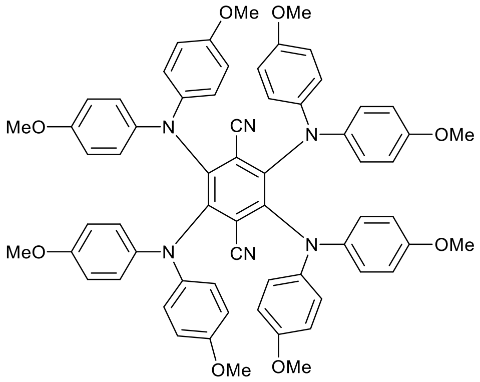

Synthetized with the general procedure at 0.4 mmol scale. Isolated as purple solid. Purified by recrystallizing from DCM/EtOH by slow evaporation. Yield 87 % (361 mg).

^{1}H NMR (400 MHz, $CDCl_3$) δ 6.72 – 6.60 (m, 32H), 3.70 (s, 24H).

^{13}C{^{1}H} NMR (101 MHz, $CDCl_3$) δ 155.5, 145.3, 139.1, 123.2, 121.3, 114.0, 113.9, 55.4.

IR (ATR), cm^{-1}: 3041, 2994, 2964, 2932, 2910, 2835, 1611, 1589, 1502, 1462, 1432, 1409, 1333, 1280, 1236, 1179, 1169, 1109, 1041, 1023, 920, 821, 779, 738, 720, 709, 683, 667, 638, 594, 564, 524, 491, 469, 448, 424, 412, 405

HRMS- ESI (pos): [M+Na]$^+$ calculated for $C_{64}H_{56}N_6O_8Na$ 1059.4052. Found 1059.4015.

4DPAPN-^{t}Bu (3,4,5,6-tetrakis(bis(4-(tert-butyl)phenyl)amino)phthalonitrile (7c)

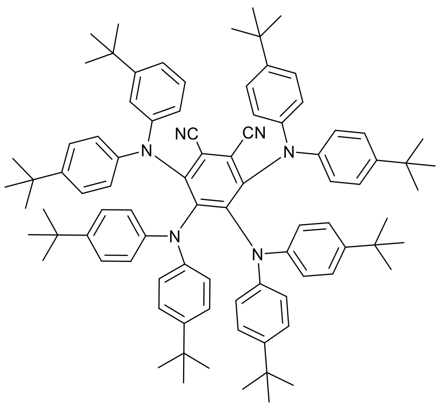

In the glove box in 120 ml Schlenk flask 4,4'-Di-tert-butyldiphenylamine (735.5 mg, 2,75 mmol, 5.5 equiv), NaH (66 mg, 2.75 mmol, 5.5 equiv) and DMSO (10 ml). The mixture was stirred in the glove box for about 30 minutes until bubbling had seized. Tetrafluorophthalonitrile (100 mg, 0.5 mmol)

was added outside the glove box under argon flow and another 10 ml batch of dry DMSO was used to rinse the sides of the flask. The flask was then put to a 50 °C oil bath and stirred for 18 hours. After this period, the reaction mixture was poured to a beaker of water (about 100 ml) and the precipitated orange product was filtered and washed with water and ethanol. The crude orange product was purified by column chromatography with 1:1 DCM:hex. Yield 79 % (494 mg).

^{1}H NMR (400 MHz, $CDCl_3$) δ 7.15 (dt, *J* = 8.8, 2.0 Hz, 8H), 6.78 (dt, *J* = 8.8, 2.9 Hz, 8H), 6.68 (dt, *J* = 8.7, 3.1 Hz, 8H), 6.28 (dt, *J* = 8.7, 2.0 Hz, 8H), 1.22 (s, 36H), 1.00 (s, 36H).

^{13}C{^{1}H} NMR (101 MHz, $CDCl_3$) δ 151.7, 148.3, 145.8, 145.7, 141.9, 141.2, 125.4, 124.1, 121.6, 121.4, 116.8, 114.2, 34.2, 34.0, 31.5, 31.3.

IR (ATR), cm^{-1}: 3036, 2958, 2903, 2865, 2228, 1607, 1508, 1460, 1427, 1393, 1363, 1316, 1288, 1267, 1196, 1114, 1016, 925, 823, 774, 728, 654, 617, 578, 562, 494, 437

HRMS-ESI (pos): $[M+Na]^+$ calculated for $C_{88}H_{104}N_6Na$ 1267.8215. Found 1267.8203.

4DPAIPN-^{t}Bu (2,4,5,6-tetrakis(bis(4-(tert-butyl)phenyl)amino)isophthalonitrile) (7b)

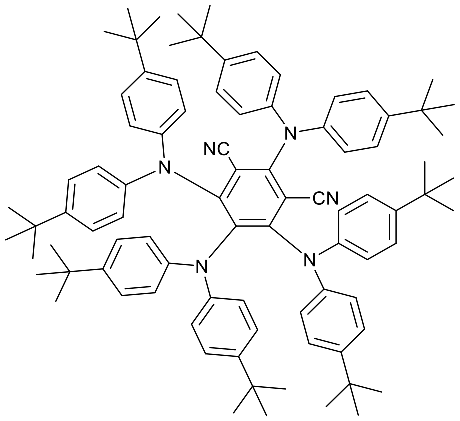

Synthetized by the same method as 4DPAPN-^{t}Bu. The crude orange-yellow product was purified by column chromatography with 2:1 $CHCl_3$:hex. Yield 32 % (200 mg). Low yield is due to many impure fractions that were collected but not purified further as there was no need for more product.

^{1}H NMR (400 MHz, $CDCl_3$) δ 7.29 (d, *J* = 8.6 Hz, 4H), 7.05 (t, *J* = 8.5 Hz, 12H), 6.82 (d, *J* = 8.6 Hz, 4H), 6.61 (d, *J* = 8.4 Hz, 8H), 6.40 (d, *J* = 8.6 Hz, 4H), 1.27 (s, 20H), 1.15 (s, 36H), 1.03 (s, 18H).

^{13}C{^{1}H} NMR (101 MHz, $CDCl_3$) δ 154.7, 151.4, 146.3, 146.0, 144.8, 143.2, 141.9, 140.9, 126.1, 125.2, 124.0, 122.2, 121.7, 120.6, 114.2, 113.4, 34.4, 34.2, 33.9, 31.5, 31.4, 31.3.

IR (ATR), cm^{-1}: 3036, 2959, 2903, 2865, 1606, 1508, 1460, 1428, 1402, 1363, 1315, 1288, 1268, 1243, 1193, 1114, 1016, 824, 728, 666, 645, 624, 577, 558, 507, 436, 406

HRMS-ESI (pos): $[M+Na]^+$ calculated for $C_{88}H_{104}N_6Na$ 1267.8215. Found 1267.8187.

4DPATPN-tBu (2,3,5,6-tetrakis(bis(4-(tert-butyl)phenyl)amino)terephthalonitrile) (9c)

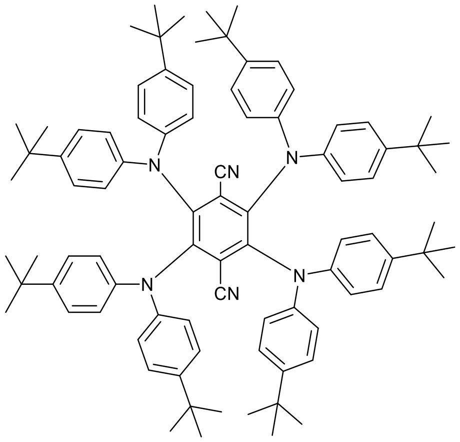

Synthetized by the same method as 4DPAPN-tBu and 4DPAIPN-tBu. The crude red product was purified by column chromatography with 2:1 $CHCl_3$:hex. Yield 39 % (243 mg). Low yield is due to many impure fractions that were collected but not purified further as there was no need for more product.

^{1}H NMR (400 MHz, $CDCl_3$) δ 7.12 – 6.34 (m, 32H), 3.70 (s, 34H).

^{13}C{^{1}H} NMR (101 MHz, $CDCl_3$) δ 155.5, 145.3, 139.1, 123.2, 121.4, 114.1, 113.9, 55.5.

IR (ATR), cm^{-1}: 3036, 2959, 2902, 2865, 1606, 1510, 1460, 1430, 1409, 1363, 1329, 1315, 1289, 1268, 1232, 1192, 1113, 1039, 1016, 925, 824, 730, 691, 665, 614, 577, 555, 483, 442, 406.

HRMS-ESI(pos): $[M]^+$ calculated for $C_{64}H_{56}N_6O_8Na$ 1036.4154. Found 1036.4128.

4DPAPN-Ph (3,4,5,6-tetrakis(di([1,1'-biphenyl]-4-yl)amino)phthalonitrile) (7d)

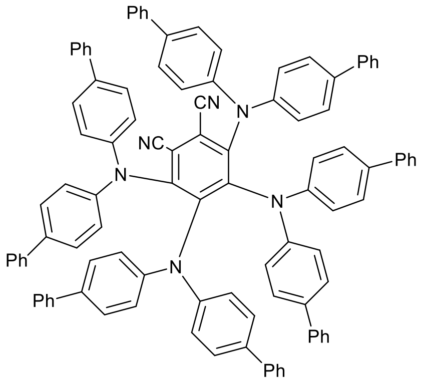

Synthetized with the general procedure at 0.545 mmol scale. Isolated as red solid. Purified by recrystallizing from DCM/EtOH by slow evaporation. Yield 71 % (547.8 mg).

^{1}H NMR (400 MHz, $CDCl_3$) δ 7.52 – 7.45 (m, 16H), 7.44 – 7.37 (m, 8H), 7.35 – 7.21 (m, 24H), 7.17 – 7.11 (m, 8H), 7.03 – 6.97 (m, 8H), 6.72 – 6.66 (m, 8H).

$^{13}C\{^{1}H\}$ NMR (101 MHz, $CDCl_3$) δ 150.1, 147.3, 143.8, 143.2, 140.5, 140.4, 136.9, 136.8, 128.8, 128.7, 127.8, 127.1, 127.0, 126.9, 126.8, 126.7, 122.7, 122.5, 117.6, 113.7.

IR (ATR), cm^{-1}: 3028, 1600, 1514, 1482, 1450, 1422, 1393, 1352, 1311, 1275, 1255, 1229, 1190, 1109, 1074, 1040, 1006, 909, 827, 757, 727, 718, 692, 607, 586, 549, 500, 436.

HRMS-ESI (neg): $[M+HCOO]^-$ calculated for $C_{105}H_{73}N_6O_2$ 1450.5879. Found 1450.5871.

4DPAIPN-Ph (2,4,5,6-tetrakis(di([1,1'-biphenyl]-4-yl)amino)isophthalonitrile) (8d)

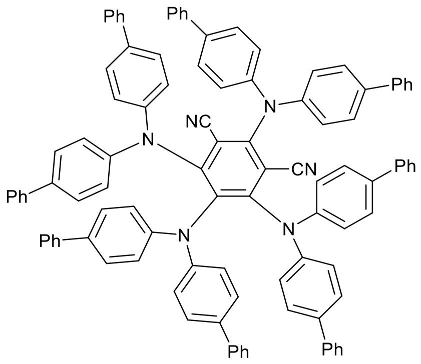

Synthetized with the general procedure at 0.545 mmol scale. Isolated as orange solid. Purified by recrystallizing from DCM/EtOH by slow evaporation.

Yield 79 % (602.5 mg).

^{1}H NMR (400 MHz, $CDCl_3$) δ 7.64 – 7.56 (m, 4H), 7.55 – 7.47 (m, 4H), 7.45 – 7.20 (m, 52H), 6.99 – 6.91 (m, 8H), 6.86 – 6.78 (m, 4H).

$^{13}C\{^{1}H\}$ NMR (101 MHz, $CDCl_3$) δ 154.0, 152.2, 144.8, 144.1, 142.3, 140.5, 140.4, 140.4, 139.0, 137.3, 137.2, 135.5, 128.8, 128.8, 128.8, 128.3, 127.6, 127.2, 127.1, 127.0, 126.9, 126.9, 126.7, 126.5, 123.5, 123.2, 121.3, 113.3, 112.6.

IR (ATR), cm^{-1}: 3057, 3029, 2221, 1600, 1514, 1482, 1450, 1422, 1393, 1311, 1274, 1257, 1236, 1193, 1113, 1075, 1042, 1007, 909, 828, 758, 725, 693, 640, 607, 587, 547, 504, 452, 434, 405.

HRMS-ESI (neg): $[M+HCOO]^-$ calculated for $C_{105}H_{73}N_6O_2$ 1450.5879. Found 1450.5902.

4DPATPN-Ph (2,3,5,6-tetrakis(di([1,1'-biphenyl]-4-yl)amino)terephthalonitrile) (9d)

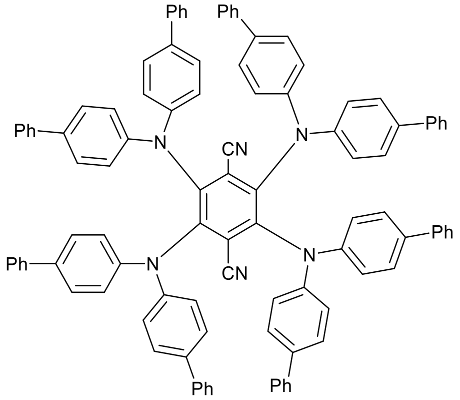

Synthetized with the general procedure at 0.545 mmol scale. Isolated as red solid. Purified by recrystallizing from DCM/EtOH by slow evaporation. Yield 74 % (570.0 mg).

^{1}H NMR (400 MHz, $CDCl_3$) δ 7.52 – 7.38 (m, 32H), 7.37 – 7.19 (m, 24H), 7.05 – 6.92 (m, 16H).
^{13}C NMR: Too low solubility

IR (ATR), cm^{-1}: 3031, 2226, 1603, 1516, 1483, 1453, 1432, 1404, 1318, 1285, 1277, 1235, 1191, 1116, 1074, 1045, 1007, 995, 911, 831, 761, 728, 719, 696, 668, 597, 582, 549, 505, 458, 447, 406.

HRMS-ESI (pos): $[M+Na]^+$ calculated for $C_{104}H_{72}N_6Na$ 1427.5716. Found 1427.5776.

10-(naphthalen-1-yl)-10H-phenoxazine

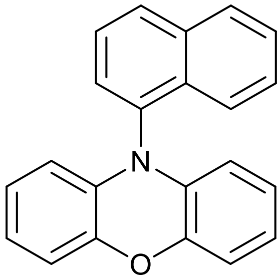

In a 120 ml oven dried Schlenk flask was added a stirring magnet, 10H-phenoxazine (1.464 g, 8 mmol, 1 equiv), grinded Cs_2CO_3 (5.20 g, 16 mmol, 2 equiv), CuI (304 mg, 1.6 mmol, 20 mol %) and N,N-Dimethylglycine hydrochloride (224 mg, 1.6 mmol, 20 mol %). The flask was stoppered and cycled onto the Schlenk line using argon as the inert gas. 20 ml of degassed anhydrous DMSO was added under argon flow and the mixture was stirred for a few minutes. Finally, 1-iodonaphthalene (1.400 ml, 9.6 mmol, 1.2 equiv) was added under argon flow. The mixture was stirred at 90 °C for 48 hours. The reaction mixture was then diluted with DCM, transferred to a separatory funnel and washed with water (1x 20 ml), 0.25 M EDTA in 2M NH_3 (2x 10 ml) and brine (1x 20 ml). The organic phase was dried over anhydrous $MgSO_4$ and the solvent was evaporated. The crude product was dissolved in DCM, layered with hexane (about 1:3 DCM:Hex) and placed in the freezer

overnight. After filtering, a white solid was recovered. Yield 46 %, 1.14 g. NMR spectrum match that published previously.[13]

^{1}H NMR (400 MHz, $CDCl_3$) δ 8.09 (d, *J* = 8.4 Hz, 1H), 7.99 (dd, *J* = 8.2, 3.2 Hz, 2H), 7.67 (t, *J* = 7.8 Hz, 1H), 7.60 – 7.52 (m, 2H), 7.52 – 7.43 (m, 1H), 6.73 (dd, *J* = 6.5, 1.4 Hz, 2H), 6.64 (td, *J* = 7.6, 1.5 Hz, 2H), 6.50 (td, *J* = 6.1, 1.5 Hz, 2H), 5.71 (dd, *J* = 8.0, 1.5 Hz, 2H).

10 SYNTHESIS OF O-ARYL CARBAMATES

10.1 GENERAL PROCEDURE

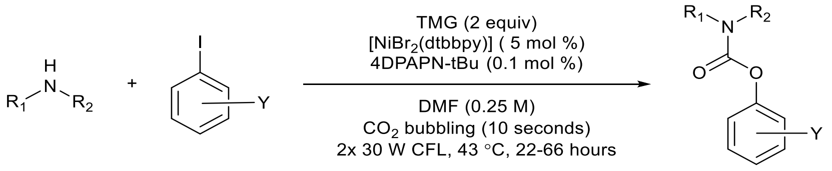

$NiBr_2$dtbbpy was used as 0.1 M stock solution in DMF and was prepared by mixing 437.0 mg of anhydrous $NiBr_2$ and 536.8 mg of dtbbpy in 15 ml of dry DMF, and then heating at 60°C for 1 hour, during which green solution forms. The solution was then diluted to 20 ml. 4DPAPN-^{t}Bu was used as 0.002 M stock solution in DMF that was prepared by weighting 49.8 mg of 4DPAPN-^{t}Bu, which was dissolved in 20 ml of DMF.

In an 8 ml vial were added DMF (sufficient amount to get 4 ml final volume), $NiBr_2$dtbbpy (0.5 ml of 0.1 M solution, 0.05 mmol, 5 mol %), 4DPAPN-^{t}Bu (0.5 ml of 0.002 M solution, 0.001 mmol, 0.1 mol %), ArI (1 mmol, 0.25 M), amine (2 mmol, 2 equiv OR 1.1 mmol, 1.1 eq in case of pyridines) and TMG (0.251 ml, 2 mmol, 2 equiv). The vial was capped with a septum cap after which CO_2 was bubbled for 10 seconds with a needle. The vial was sealed with parafilm, placed between two 30 W white CFL lights and stirred vigorously (1200 rpm) at 43 °C for 22 hours. After the reaction period, a sample was taken for GC-MS. If significant amounts of starting material was detected (> 10 % of total peak areas), the reaction was continued for further 22-48 hours (depending on the completeness of the reaction after initial 22 hours). After the reaction was complete, the contents of one reaction mixture, two identical reaction mixtures (1 or 2 mmol scale) or contents of a Schlenk flask (20 ml) were isolated by extraction. Two similar extraction methods were employed.

Method 1: Reaction mixture was added to a separatory funnel containing 20 ml of water. The reaction mixture was extracted with three 10 ml portions of ethyl acetate. The combined organic phases were washed with two 10 ml portions of water and one 10 ml portion of brine. The organic phase was dried over anhydrous $MgSO_4$, filtered and evaporated to give the crude product.

Method 2: The reaction mixture was poured into 30 ml of 2 M Na_2CO_3(aq) solution and extracted with 30 ml EtOAc. Organic layer was then washed with 30 ml H_2O and 30 ml of brine. Organic layer was dried over Na_2SO_4, filtered and evaporated to give the crude product.

The crude product was purified by column chromatography (about 50 g of 43-63 μ silica gel) or in the case of 4-(trifluoromethyl)phenyl 3-oxopiperazine-1-carboxylate by recrystallization.

Hex:EtOAc solvent mixtures were used as eluent. After purification, the solvent of the combined product containing fractions was evaporated and the product was further dried in an oil pump if necessary.

Note: In the NMR, the carbamate has hindered rotation around the amide bond. This causes splitting of the amine ^{1}H and ^{13}C signals. With unsymmetric amines 11 and 15, also the aryl carbons are splitted.[14-16]

10.2 AMINE COUPLING PARTNERS

4-(trifluoromethyl)phenyl morpholine-4-carboxylate (3)

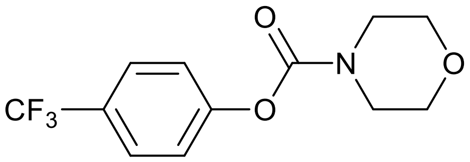

Synthetized using the general procedure in 2 mmol scale. Isolation with method 1. Reaction time 22 hours. Column run with gradient starting with 1:8 EtOAc:hex with 0.5 V% isopropyl alcohol. Product was recovered as a white solid. Yield 78 %, 429 mg. NMR spectra matches the published spectra.[17]

^{1}H NMR (400 MHz, CDCl3) δ = 7.63 (d, J=8.4, 2H), 7.24 (d, J=8.4, 2H), 3.76 (t, J=4.8, 4H), 3.71 – 3.63 (bs, 2H), 3.62 – 3.51 (bs, 2H).

$^{13}C\{^{1}H\}$ NMR (101 MHz, $CDCl_3$) δ = 153.8 (q, J=1.4), 153.01, 127.72 (q, J=32.9), 126.7 (q, J=3.8), 124.0 (q, J = 271.8 Hz), 122.1, 66.6, 66.5, 45.0, 44.3.

^{19}F NMR (376 MHz, $CDCl_3$) δ -65.32.

HRMS-ESI (pos): $[M+Na]^+$ calculated for $C_{12}H_{12}F_3NO_3Na$ 298.0661. Found 298.0651.

4-(trifluoromethyl)phenyl piperidine-1-carboxylate (10)

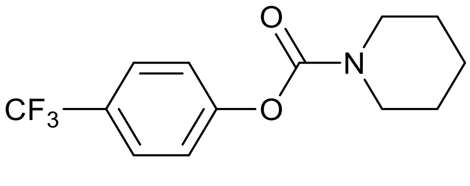

Synthetized using the general procedure at 2 mmol scale. Isolation with method 1. Reaction time 22 hours. Column run with gradient starting with 1:15 EtOAc:hex with 0.5 V% isopropyl alcohol.

Product was recovered as a white solid. Yield 81 %, 440 mg. NMR spectra matches the published spectra.[18]

^{1}H NMR (400 MHz, $CDCl_3$) δ = 7.61 (d, *J* = 8.8 Hz, 2H), 7.23 (d, *J* = 8.3 Hz, 2H), 3.60 (t, *J* = 5.0 Hz, 2H), 3.51 (t, *J* = 5.1 Hz, 2H), 1.70 – 1.57 (m, *J* = 5.3, 6H).

$^{13}C\{^1H\}$ NMR (101 MHz, $CDCl_3$) δ 154.2 (q, *J*=1.4), 152.9, 127.2 (q, *J*=32.7), 126.5 (q, *J*=3.8), 124.1 (q, J = 271.7), 122.1, 45.6, 45.2, 25.9, 25.5, 24.2.

^{19}F NMR (376 MHz, $CDCl_3$) δ = -65.06.

HRMS-ESI (pos): $[M+Na]^+$ calculated for $C_{13}H_{14}NO_2Na$ 296.0869. Found 296.0863.

4-(trifluoromethyl)phenyl ethyl(methyl)carbamate (11)

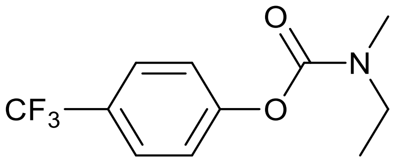

Synthetized using the general procedure at 2 mmol scale. Isolation with method 1. Reaction time 22 hours. Column run with gradient starting with 1:15 EtOAc:hex with 0.5 V% isopropyl alcohol. Product was recovered as a light-yellow oil. Yield 55 %, 270 mg.

^{1}H NMR (400 MHz, $CDCl_3$) δ 7.62 (d, *J* = 8.5 Hz, 2H), 7.24 (dd, *J* = 8.8, 3.5 Hz, 2H), 3.44 (dq, *J* = 17.3, 7.2 Hz, 2H), 3.01 (d, *J* = 29.7 Hz, 3H), 1.23 (dt, *J* = 19.4, 7.2 Hz, 3H).

$^{13}C\{^1H\}$ NMR (101 MHz, $CDCl_3$) δ 154.2, 153.9, 153.7, 127.3 (q, J = 4.4 Hz), 126.6 (q, *J* = 3.7 Hz), 124.1 (q, *J* = 271.8 Hz), 122.2, 44.3, 44.2, 34.4, 33.9, 13.3, 12.5.

^{19}F NMR (376 MHz, $CDCl_3$) δ -65.17.

IR (ATR), cm^{-1}: 2978, 2940, 1720, 1614, 1456, 1400, 1323, 1286, 1215, 1154, 1118, 1102, 1062, 1015, 940, 866, 751, 654, 591, 505, 412.

HRMS-ESI (pos): $[M+Na]^+$ calculated for $C_{11}H_{12}F_3NO_2Na$ 270.0712. Found 270.0703.

4-(trifluoromethyl)phenyl dibenzylcarbamate (12)

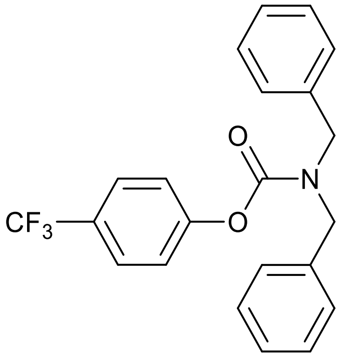

Synthetized using the general procedure at a 1 mmol scale. Isolation with method 1. Reaction time 22 hours. Column run with gradient starting with 1:8 EtOAc:hex with 0.5 V% isopropyl alcohol. Product was recovered as a light-yellow oil. Yield 85 %, 329 mg.

^{1}H NMR (400 MHz, $CDCl_3$) δ 7.65 (d, *J* = 8.4 Hz, 2H), 7.44 – 7.37 (m, 4H), 7.37 – 7.32 (m, 4H), 7.33 – 7.25 (m, 4H), 4.57 (d, *J* = 11.1 Hz, 4H).

^{13}C{^{1}H} NMR (101 MHz, $CDCl_3$) δ 154.6, 154.0, 136.8, 136.8, 129.0, 128.9, 128.5, 127.9, 127.9, 127.6, 126.8 (q, *J*=3.7), 124.1 (q, *J*=271.8), 122.2, 50.2, 49.8.

^{19}F NMR (376 MHz, $CDCl_3$) δ -65.30.

IR (ATR), cm^{-1}: 3082, 3062, 3028, 2928, 1718, 1611, 1601, 1492, 1450, 1420, 1367, 1332, 1306, 1232, 1207, 1164, 1103, 1087, 1066, 1052, 1021, 1013, 954, 934, 899, 871, 851, 747, 727, 699, 675, 651, 631, 603, 592, 576, 515, 448, 431, 411.

HRMS- ESI (pos): $[M+Na]^+$ calculated for $C_{22}H_{18}F_3NO_2Na$ 408.1182. Found 408.1185.

4-(trifluoromethyl)phenyl diisopropylcarbamate (13)

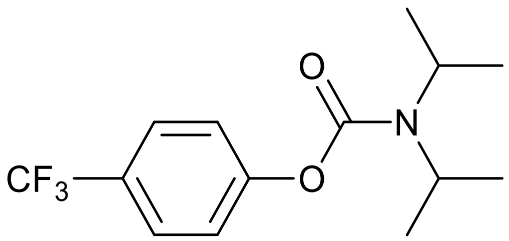

Synthetized using the general procedure at a 1 mmol scale. Isolation with method 1. Reaction time 22 hours. Column run with gradient starting with 1:25 EtOAc:hex with 0.5 V% isopropyl alcohol. Product was recovered as a light-yellow oil. Yield 47 %, 135 mg. NMR spectra matches the published spectra.[19]

^{1}H NMR (CDCl$_3$): ^{1}H NMR (400 MHz, CDCl$_3$) δ 7.62 (d, *J* = 8.4 Hz, 2H), 7.24 (d, *J* = 8.4 Hz, 2H), 4.22 – 4.03 (bs, 1H), 4.03 – 3.81 (bs, 1H), 1.37 – 1.32 (bs, 6H), 1.32 – 1.24 (bs, 6H).

^{13}C{^{1}H} NMR (101 MHz, CDCl$_3$) δ 154.1 (d, *J* = 1.5 Hz), 153.1, 127.2 (q, *J* = 32.6 Hz), 126.7 (q, *J* = 3.8 Hz), 124.2 (q, *J* = 271.7 Hz), 122.3, 47.2, 46.4, 21.6, 20.5.

^{19}F NMR (CDCl$_3$): ^{19}F NMR (376 MHz, CDCl$_3$) δ -65.24.

HRMS- ESI (pos): [M+Na]$^+$ calculated for $C_{14}H_{18}F_3NO_2Na$ 312.1182. Found 312.1171.

4-(trifluoromethyl)phenyl 3-oxopiperazine-1-carboxylate (14)

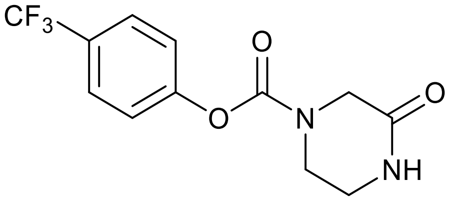

Synthetized using the general procedure at a 1 mmol scale in a Schlenk flask (20 ml, 0.05 M). Isolation with method 2. Reaction time 22 hours. Recrystallized from hot hexane/EtOAc. Product was recovered as white solid. Yield 36 %, 102.3 mg.

^{1}H NMR (400 MHz, CDCl$_3$) δ 7.65 (d, *J* = 8.5 Hz, 2H), 7.26 (d, *J* = 8.4 Hz, 2H), 7.14 – 6.91 (m, 1H), 4.34 (s, 1H), 4.23 (s, 1H), 3.89 (t, *J* = 5.4 Hz, 1H), 3.79 (t, *J* = 5.3 Hz, 1H), 3.50 (q, *J* = 5.7 Hz, 2H).
^{13}C{^{1}H} NMR (101 MHz, CDCl$_3$) δ 167.9, 167.5, 153.5, 153.5, 152.4, 152.4, 128.0 (q, *J* = 32.9 Hz), 126.9 (q, *J* = 3.8 Hz), 123.9 (q, *J* = 271.9 Hz), 122.1, 47.8, 47.5, 41.5, 41.1, 40.8, 40.7.
^{19}F NMR (376 MHz, CDCl$_3$) δ -65.31.
HRMS-ESI (pos): [M+Na]$^+$ calculated for $C_{12}H_{11}F_3N_2O_3Na$ 311.0614. Found 311.0601.

2-methyl 1-(4-(trifluoromethyl)phenyl) pyrrolidine-1,2-dicarboxylate (15)

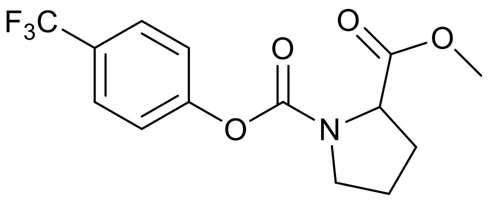

Synthetized using the general procedure at a 1 mmol scale. Isolation with method 2. Reaction time 22 hours. Column run with 1:6 EtOAc:hex. Product was recovered as white solid. Yield 76 %, 227.1 mg.

^{1}H NMR (400 MHz, CDCl$_3$) δ 7.58 (dd, *J* = 8.8, 2.7 Hz, 2H), 7.26 (d, *J* = 8.5 Hz, 1H), 7.19 (d, *J* = 8.5 Hz, 1H), 4.59 – 4.26 (m, 1H), 3.96 – 3.28 (m, 5H), 2.38 – 1.85 (m, 4H).

$^{13}C\{^1H\}$ NMR (101 MHz, $CDCl_3$) δ 172.8, 172.5, 153.8, 153.7, 152.4, 151.9, 127.5 (q, *J* = 32.7 Hz), 127.3 (q, *J* = 32.6 Hz), 126.6 (q, *J* = 3.7 Hz), 126.5 (q, J =3.7 Hz), 124.0 (q, *J* = 271.6 Hz), 122.0, 59.4, 59.3, 52.4, 52.3, 47.2, 47.0, 30.9, 29.9, 24.3, 23.4.

^{13}C-NMR has differentiable rotamer signal for signals all except 124.0 and 122.0 ppm signals, which have almost complete overlap.

^{19}F NMR (376 MHz, $CDCl_3$) δ -65.02, -65.03

HRMS-ESI(pos): $[M+Na]^+$ Calculated for $C_{14}H_{14}F_3N_2O_4Na$: 340.0767. Found: 340.0758

4-(trifluoromethyl)phenyl 4-benzylpiperazine-1-carboxylate (16)

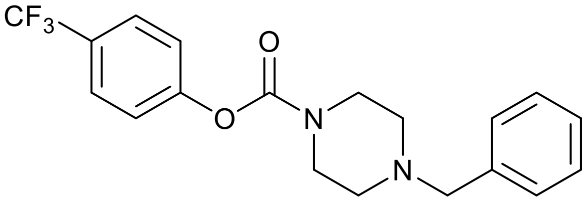

Synthetized using the general procedure at a 1 mmol scale. Isolation with method 2. Reaction time 22 h hours. Column run with 1:6 EtOAc:hex. Product was recovered as white solid. Yield 76.7 %, 279.5 mg.

^1H NMR (400 MHz, $CDCl_3$) δ 7.62 – 7.55 (m, 2H), 7.36 – 7.27 (m, 4H), 7.31 – 7.22 (m, 1H), 7.25 – 7.17 (m, 2H), 3.70 – 3.54 (m, 4H), 3.53 (s, 2H), 2.50 – 2.44 (m, 4H).
$^{13}C\{^1H\}$ NMR (101 MHz, $CDCl_3$) δ 154.0 (d, *J* = 1.5 Hz), 152.8, 137.6, 129.1, 128.4, 127.3 (q, *J* = 32.7 Hz), 127.3, 126.6 (q, *J* = 3.7 Hz), 124.0 (q, *J* = 271.8 Hz), 122.1, 62.9, 52.7, 52.5, 44.6, 44.1.
^{19}F NMR (376 MHz, $CDCl_3$) δ -64.90
HRMS-ESI (pos): $[M+H]^+$ calculated for $C_{19}H_{20}F_3N_2O_2$ 365.1471. Found 365.1473.

4-(trifluoromethyl)phenyl 4-(pyridin-2-yl)piperazine-1-carboxylate (17)

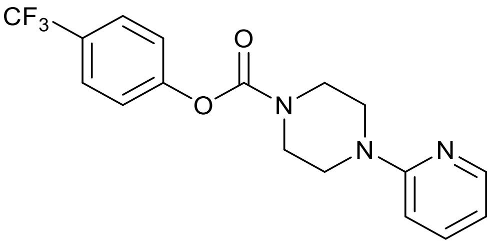

Synthetized using the general procedure at a 1 mmol scale, with using 1.1 mmol of pyridyl piperazine. Isolation with method 2. Reaction time 22 hours. Column run with 1:10 EtOAc:hex for 3 column volumes and then with 1:1 EtOAc:hex + 1 % Et_3N. Product was recovered as white solid. Yield 62.2 %, 217 mg. (When 2 mmol of pyridyl piperazine was used, the yield was 172.7 mg 49.2 %).

^{1}H NMR (400 MHz, $CDCl_3$) δ 8.22 (dd, *J* = 5.4, 2.0 Hz, 1H), 7.67 – 7.60 (m, 2H), 7.56 – 7.46 (m, 1H), 7.26 (d, *J* = 8.5 Hz, 2H), 6.72 – 6.65 (m, 2H), 3.84 – 3.58 (m, 8H).

^{13}C NMR (101 MHz, $CDCl_3$) δ 159.1, 153.9 (d, *J* = 1.8 Hz), 153.0, 148.1, 137.7, 127.6 (q, *J* = 33.0 Hz), 126.7 (q, *J* = 3.8 Hz), 124.1 (q, J = 271.9 Hz), 122.1, 114.1, 107.3, 45.1, 45.0, 44.4, 43.7.

^{19}F NMR (376 MHz, $CDCl_3$) δ -65.22

HRMS-ESI (pos): $[M+H]^+$ calculated for $C_{17}H_{17}F_3N_3O_2$ 352.1267. Found 352.1255.

bis(4-(trifluoromethyl)phenyl) piperazine-1,4-dicarboxylate (18)

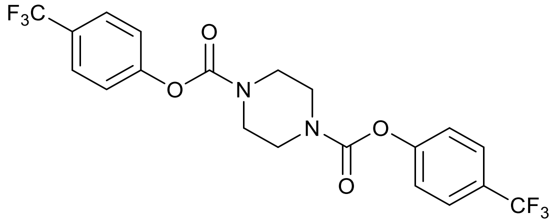

Synthetized using the general procedure with 1 mmol piperazine, 2 mmol TMG, 2 mmol iodobenzotrifluoride. Isolation with method 2. Reaction time 22 hours. Column run with 1:6 EtOAc:hex. Product was recovered as white solid. Yield 27.2 %, 126.0 mg.

^{1}H NMR (400 MHz, $CDCl_3$) δ 7.87 – 7.43 (m, 4H), 7.43 – 6.92 (m, 4H), 4.13 – 3.25 (m, 8H).

^{13}C NMR (101 MHz, $CDCl_3$) δ 153.73 (d, *J* = 1.5 Hz), 152.95, 127.96 (q, *J* = 32.8 Hz), 126.87 (q, *J* = 3.7 Hz), 124.03 (q, *J* = 271.8 Hz), 122.17, 84.43 – 69.82 (m), 44.14 (d, *J* = 62.5 Hz).

^{19}F NMR (376 MHz, $CDCl_3$) δ -65.30

4-(trifluoromethyl)phenyl 4-(8-chloro-5,6-dihydro-11H-benzo[5,6]cyclohepta[1,2-b]pyridin-11-ylidene)piperidine-1-carboxylate (19)

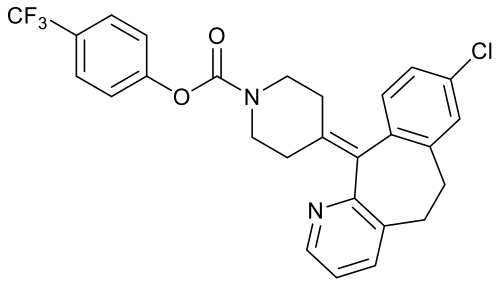

Synthetized using the general procedure at a 1 mmol scale. Isolation with method 2. Reaction time 22 hours. Column run with 1:6 EtOAc:hex. Product was recovered as white solid. Yield 37.0 %, 184.4 mg.

^{1}H NMR (400 MHz, $CDCl_3$) δ 8.41 (dd, *J* = 4.8, 1.7 Hz, 1H), 7.61 (d, *J* = 8.4 Hz, 2H), 7.44 (dd, *J* = 7.7, 1.7 Hz, 1H), 7.24 (d, *J* = 8.4 Hz, 2H), 7.21 – 7.18 (m, 1H), 7.17-7.13 (m, 2H), 7.10 (dd, *J* = 7.7, 4.8 Hz, 1H), 4.04 – 3.83 (m, 2H), 3.47 – 3.23 (m, 4H), 2.93 – 2.75 (m, 2H), 2.71 – 2.37 (m, 4H).

$^{13}C\{^1H\}$ NMR (101 MHz, $CDCl_3$) δ 156.9, 154.1 (d, J = 1.8 Hz), 152.9, 146.8, 139.7, 137.7, 137.6, 136.5, 135.0, 133.4, 133.1, 130.5, 129.1, 127.4 (q, J = 32.7 Hz), 126.7 (q, J = 3.6 Hz), 126.3, 124.1 (q, J = 271.9 Hz), 122.5, 122.2, 45.6, 45.4, 45.3, 31.8, 31.6, 30.9, 30.7, 30.5, 30.3

^{19}F NMR (376 MHz, $CDCl_3$) δ -65.25

HRMS-ESI (pos): $[M+H]^+$ calculated for $C_{27}H_{23}ClF_3N_2O_2$ 499.1395. Found 499.1411.

10.3 Aryl iodide coupling partners

4-cyanophenyl morpholine-4-carboxylate (20)

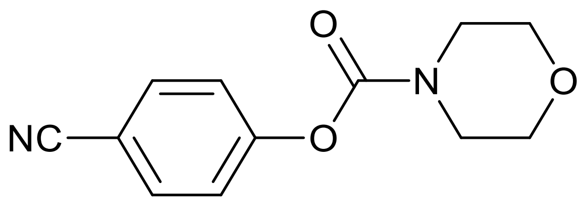

Synthetized using the general procedure at a 2 mmol scale. Reaction time 22 hours. Isolation with method 1. Column run with gradient starting with 1:4 EtOAc:hex with 0.5 V% isopropyl alcohol. Product was recovered as a white solid. Yield 92 %, 431 mg. NMR spectra matches the published spectra.[20]

^1H NMR (400 MHz, $CDCl_3$) δ = 7.66 (d, J = 8.9, 2H), 7.26 (d, J = 8.9 Hz, 2.1, 2H), 3.75 (t, J = 4.6 Hz, 4H), 3.67 (bs, 2H), 3.57 (bs, 2H).

$^{13}C\{^1H\}$ NMR (101 MHz, $CDCl_3$) δ = 154.6, 152.4, 133.6, 122.7, 118.4, 109.2, 66.6, 66.4, 45.0, 44.2.

HRMS-ESI (pos): $[M+Na]^+$ calculated for $C_{12}H_{12}N_2O_3Na$ 255.0740. Found 255.0735.

4-formylphenyl morpholine-4-carboxylate (21)

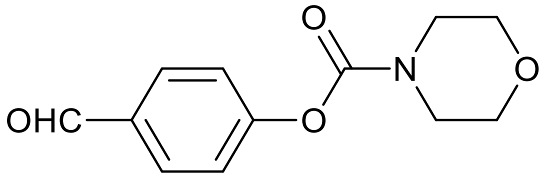

Synthetized using the general procedure at a 2 mmol scale. Reaction time 22 hours. Isolation with method 1. Column run with gradient starting with 1:4 EtOAc:hex with 0.5 V% isopropyl alcohol. Product was recovered as a white solid. Yield 51 %, 240 mg. GC-MS showed that benzaldehyde was a significant side product. NMR spectra matches the published spectra.[20]

^{1}H NMR (400 MHz, $CDCl_3$) δ = 9.97 (s, 1H), 7.89 (m, 2H), 7.29 (m, 2H), 3.74 (bs, 4H), 3.67 (bs, 2H), 3.57 (bs, 2H).

$^{13}C\{^{1}H\}$ NMR (101 MHz, $CDCl_3$) δ = 191.1, 156.0, 152.7, 133.6, 131.2, 122.3, 66.6, 66.5, 45.0, 44.2.

HRMS-ESI (pos): $[M+Na]^+$ calculated for $C_{12}H_{13}NO_4Na$ 258.0737. Found 258.0736.

4-(methoxycarbonyl)phenyl morpholine-4-carboxylate (22)

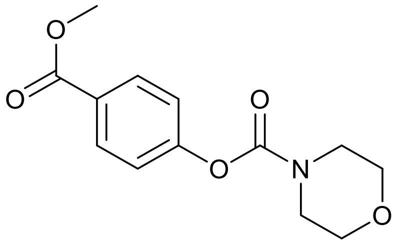

Synthetized using the general procedure at a 1 mmol scale. Isolation with method 2. Reaction time 22 hours. Column run with 1:6 EtOAc:hex. Product was recovered as white solid. Yield 82.4 %, 218.7 mg.

1H NMR (400 MHz, CDCl3) δ 8.07 – 7.99 (m, 2H), 7.21 – 7.14 (m, 2H), 3.86 (s, 3H), 3.73 – 3.67 (m, 4H), 3.66 – 3.59 (m, 2H), 3.56 – 3.49 (m, 2H).

$^{13}C\{^{1}H\}$ NMR (101 MHz, $CDCl_3$) δ 166.2, 154.9, 152.8, 131.0, 127.1, 121.5, 66.5, 66.4, 52.0, 44.9, 44.1.

HRMS-ESI (pos): $[M+Na]^+$ calculated for $C_{13}H_{15}NO_5Na$ 288.0850. Found 288.0842.

4-chlorophenyl morpholine-4-carboxylate (23)

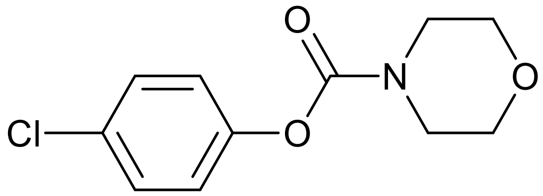

Synthetized using the general procedure at a 2 mmol scale. Reaction time 44 hours. Isolation with method 1. Column run with gradient starting with 1:8 EtOAc:hex with 0.5 V% isopropyl alcohol. Product was recovered as a white solid. Yield 66 %, 329 mg. GC-MS showed that the corresponding directly aminated coupling product was a significant side product.

^{1}H NMR (400 MHz, $CDCl_3$) δ = 7.36 – 7.28 (m, 2H), 7.09 – 7.01 (m, 2H), 3.74 (t, *J*=5.2 Hz, 4H), 3.70 – 3.61 (bs, 2H), 3.59 – 3.52 (bs, 2H).

$^{13}C\{^{1}H\}$ NMR (101 MHz, $CDCl_3$) δ 153.4, 149.8, 130.8, 129.4, 123.1, 66.7, 66.5, 44.9, 44.2.

IR (ATR), cm^{-1}: 3102, 3075, 2996, 2913, 2897, 2853, 1905, 1713, 1590, 1488, 1454, 1412, 1360, 1301, 1278, 1239, 1194, 1157, 1119, 1087, 1058, 1010, 989, 929, 871, 858, 820, 795, 751, 708, 677, 647, 571, 511, 474, 417.

HRMS-ESI (pos): $[M+Na]^+$ calculated for $C_{11}H_{12}ClNO_3Na$ 264.0398. Found 264.0401.

4-(4,4,5,5-tetramethyl-1,3,2-dioxaborolan-2-yl)phenyl morpholine-4-carboxylate (24)

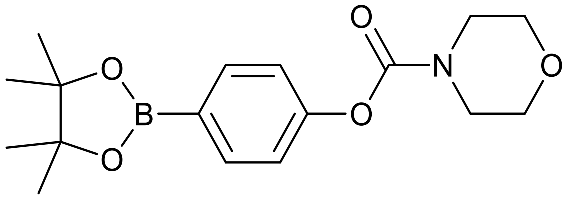

Synthetized using the general procedure at a 2 mmol scale. Reaction time 44 hours. Isolated using method 1. Column run with gradient starting with 1:4 EtOAc:hex with 0.5 V% isopropyl alcohol. Product was recovered as a white solid. Yield 55 %, 372 mg. NMR spectra matches the published spectra.[17]

^{1}H NMR ($CDCl_3$): ^{1}H NMR (400 MHz, $CDCl_3$) δ 7.82 (d, J = 8.5 Hz, 2H), 7.12 (d, J = 8.5 Hz, 2H), 3.74 (t, J = 5.1 Hz, 4H), 3.69 – 3.65 (m, 2H), 3.60 – 3.54 (m, 2H), 1.33 (s, 12H).

^{13}C{^{1}H} NMR (101 MHz, $CDCl_3$) δ = 153.8, 153.4, 136.1, 120.9, 83.8, 66.6, 66.5, 44.9, 44.1, 24.8.

HRMS-ESI (pos): $[M+Na]^+$ calculated for $C_{17}H_{24}BNO_5Na$ 356.1640. Found 356.1627.

4-bromophenyl morpholine-4-carboxylate (25)

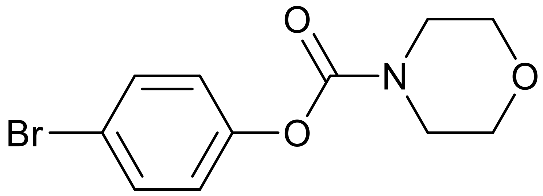

Synthetized using the general procedure at a 2 mmol scale. Reaction time 44 hours. Isolation with method 1. Column run with gradient starting with 1:8 EtOAc:hex with 0.5 V% isopropyl alcohol. Product was recovered as a white solid. Yield 57 %, 326 mg. GC-MS showed that the corresponding directly aminated coupling product was a significant side product.

^{1}H NMR (400 MHz, $CDCl_3$) δ 7.49 (d, *J* = 8.5 Hz, 2H), 7.03 (d, *J* = 8.9 Hz, 2H), 3.76 (t, *J* = 5.1 Hz, 4H), 3.71 – 3.64 (m, 2H), 3.61 – 3.54 (m, 2H).

$^{13}C\{^{1}H\}$ NMR (101 MHz, $CDCl_3$) δ 153.3, 150.3, 132.4, 123.5, 118.5, 66.7, 66.5, 44.9, 44.2.

IR (ART) 3092, 3058, 2969, 2921, 2900, 2854, 1708, 1588, 1488, 1456, 1421, 1366, 1304, 1277, 1242, 1225, 1198, 1169, 1109, 1056, 1023, 1008, 987, 871, 845, 793, 749, 703, 665, 633, 573, 504, 461, 420.

HRMS-ESI (pos): $[M+Na]^+$ calculated for $C_{11}H_{12}BrNO_3$ 307.9893. Found 307.9883.

3-bromophenyl morpholine-4-carboxylate (26)

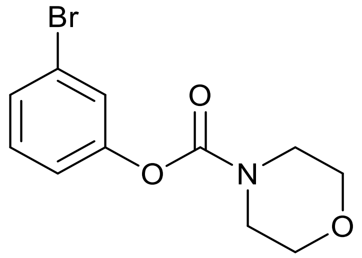

Synthetized using the general procedure at a 1 mmol scale. Isolation with method 2. Reaction time 48 hours. Column run with 1:6 EtOAc:hex. Product was recovered as white solid. Yield 45.4 %, 129.9 mg.

^{1}H NMR (400 MHz, $CDCl_3$) δ 7.37 – 7.29 (m, 2H), 7.22 (t, *J* = 8.0 Hz, 1H), 7.07 (ddd, *J* = 8.2, 2.3, 1.0 Hz, 1H), 3.73 (t, *J* = 4.8 Hz, 4H), 3.67 – 3.48 (m, 4H).

$^{13}C\{^{1}H\}$ NMR (101 MHz, $CDCl_3$) δ 153.2, 151.8, 130.4, 128.7, 125.3, 122.3, 120.6, 66.6, 66.5, 44.9, 44.2.

HRMS-ESI(pos): $[M+Na]^+$ Calculated for $C_{11}H_{12}BrNO_3Na$ 307.9893. Found 307.9899.

2-bromophenyl morpholine-4-carboxylate (27)

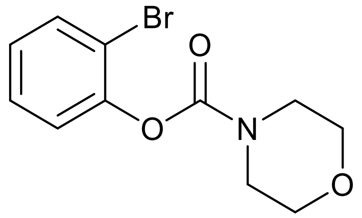

Synthetized using the general procedure at a 1 mmol scale. Isolation with method 2. Reaction time 48 hours. Column run with 1:6 EtOAc:hex. Product was recovered as white solid. Yield 13.8 %, 39.4 mg. NMR spectra matches the published spectra.[21]

^{1}H NMR (400 MHz, $CDCl_3$) δ 7.59 (dd, *J* = 8.0, 1.5 Hz, 1H), 7.33 (ddd, *J* = 8.1, 7.4, 1.5 Hz, 1H), 7.23 (dd, *J* = 8.1, 1.6 Hz, 1H), 7.11 (ddd, *J* = 8.0, 7.4, 1.6 Hz, 1H), 3.84-3.50 (m, 8H).

^{13}C{^{1}H} NMR (101 MHz, $CDCl_3$) δ 152.6, 148.6, 133.3, 128.5, 127.1, 124.2, 116.5, 66.7, 45.3, 44.5.

HRMS-ESI (pos): $[M+Na]^+$ calculated for $C_{11}H_{12}BrNO_3Na$ 307.9893. Found 307.9896.

Phenyl morpholine-4-carboxylate (28)

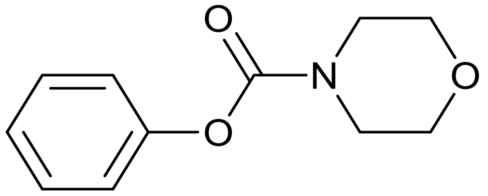

Synthetized using the general procedure at a 2 mmol scale. Reaction time 66 hours. Isolation with method 1. Column run with gradient starting with 1:4 EtOAc:hex with 0.5 V% isopropyl alcohol. Product was recovered as a white solid. Yield 26 %, 107 mg. NMR spectra matches the published spectra.[11]

^{1}H NMR (400 MHz, $CDCl_3$) δ = 7.41 – 7.32 (m, 2H), 7.25 – 7.17 (m, 1H), 7.15 – 7.08 (m, 2H), 3.75 (t, *J*=5.2 Hz, 4H), 3.70 – 3.64 (bs, 2H), 3.60 – 3.54 (bs, 2H).

^{13}C{^{1}H} NMR (101 MHz, $CDCl_3$) δ 153.8, 151.2, 129.4, 125.5, 121.7, 66.7, 66.6, 44.9, 44.1.

HRMS- ESI (pos): $[M+Na]^+$ calculated for $C_{11}H_{13}NO_3$ 230.0788. Found 230.0797.

m-tolyl morpholine-4-carboxylate (29)

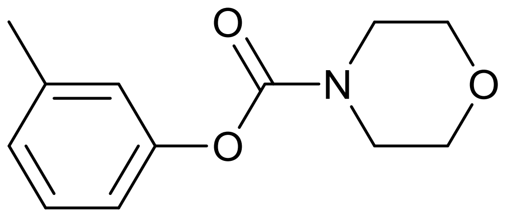

Synthetized using the general procedure at 2 mmol scale. Reaction time 66 hours. Isolation with method 1. Column run with gradient starting with 1:4 EtOAc:hex with 0.5 V% isopropyl alcohol. Product was recovered as a white solid. Yield 19 %, 85.8 mg.

^{1}H NMR (400 MHz, $CDCl_3$) δ 7.24 (t, *J* = 7.8 Hz, 1H), 7.06 – 6.98 (m, 1H), 6.96 – 6.87 (m, 2H), 3.74 (t, *J* = 4.5 Hz, 4H), 3.68 – 3.64 (m, 2H), 3.60 – 3.53 (m, 2H), 2.35 (s, 3H).

^{13}C{^{1}H} NMR (101 MHz, $CDCl_3$) δ 154.0, 151.2, 139.6, 129.1, 126.3, 122.4, 118.7, 66.7, 66.6, 44.9, 44.2, 21.4.

HRMS-ESI (pos): $[M+Na]^+$ calculated for $C_{12}H_{15}NO_3Na$ 244.0944. Found 244.0941.

5-chloropyridin-2-yl morpholine-4-carboxylate (30)

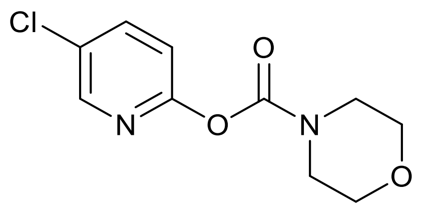

Synthetized using the general procedure at a 1 mmol scale in Schlenk flask (20 ml, 0.05 M) , with 1.1 mmol of morpholine. Isolation with method 2. Reaction time 48 hours. Column run with 1:10 EtOAc:hex for 3 column volumes and then with 1:1 EtOAc:hex + 1 % Et_3N. Product was recovered as white solid. Yield 36.6 %, 88.8 mg.

^{1}H NMR (400 MHz, $CDCl_3$) δ 8.30 (d, *J* = 2.6 Hz, 1H), 7.71 (dd, *J* = 8.6, 2.7 Hz, 1H), 7.07 (d, *J* = 8.6 Hz, 1H), 3.76 – 3.70 (m, 4H), 3.70 – 3.50 (m, 4H).

^{13}C{^{1}H} NMR (101 MHz, $CDCl_3$) δ 156.7, 152.4, 146.9, 139.2, 129.4, 117.2, 66.5, 66.4, 45.1, 44.2.

HRMS-ESI (pos): $[M+Na]^+$ calculated for $C_{10}H_{11}ClN_2O_3$ 265.0350. Found 265.0342.

pyridin-3-yl morpholine-4-carboxylate (31)

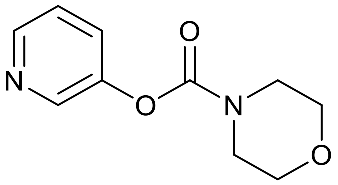

Synthetized using the general procedure at a 1 mmol scale in a Schlenk flask (20 ml, 0.05 M) , with 1.1 mmol of morpholine. Isolation with method 2. Reaction time 66 hours. Column run with 1:10 EtOAc:hex for 3 column volumes and then with 1:1 EtOAc:hex + 1 % Et_3N. Product was recovered as white solid. Yield 36.5 %, 75.9 mg. NMR spectra matches the published spectra.[22]

^{1}H NMR (400 MHz, $CDCl_3$) δ 8.47 – 8.40 (m, 2H), 7.50 (ddd, *J* = 8.3, 2.7, 1.4 Hz, 1H), 7.30 (ddd, *J* = 8.3, 4.7, 0.7 Hz, 1H), 3.74 (dd, *J* = 5.7, 3.9 Hz, 4H), 3.71 – 3.48 (m, 4H).

^{13}C{^{1}H} NMR (101 MHz, $CDCl_3$) δ 153.0, 147.9, 146.6, 143.6, 129.3, 123.8, 66.6, 66.5, 45.0, 44.3.

HRMS-ESI (pos): $[M+Na]^+$ calculated for $C_{10}H_{12}N_2O_3Na$ 231.0740. Found 231.0747.

2-fluoropyridin-4-yl morpholine-4-carboxylate (32)

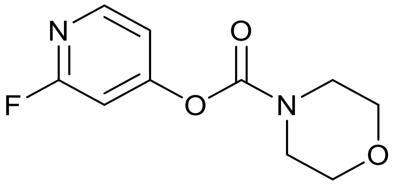

Synthetized using the general procedure at a 1 mmol scale in a Schlenk flask (20 ml, 0.05 M) , with 1.1 mmol of morpholine. Isolation with method 2. Reaction time 48 hours. Column run with 1:10 EtOAc:hex for 3 column volumes and then with 1:1 EtOAc:hex + 1 % Et_3N. Product was recovered as white solid. Yield 33.6 %, 76.1 mg.

^{1}H NMR (400 MHz, $CDCl_3$) δ 8.16 (dd, *J* = 5.8, 1.6 Hz, 1H), 7.05 – 6.99 (m, 1H), 6.81 (t, *J* = 1.6 Hz, 1H), 3.77-3.69 (m, 4H), 3.67 – 3.51 (m, 4H).

^{13}C{^{1}H} NMR (101 MHz, $CDCl_3$) δ 164.6 (d, *J* = 237.5 Hz), 161.6 (d, *J* = 11.7 Hz), 151.4, 148.4 (d, *J* = 17.5 Hz), 114.7 (d, *J* = 4.7 Hz), 102.6 (d, *J* = 41.2 Hz), 66.6, 66.4, 45.1, 44.3.

^{19}F NMR (376 MHz, $CDCl_3$) δ -68.8

HRMS-ESI (pos): $[M+Na]^+$ calculated for $C_{10}H_{11}FN_2O_3Na$ 249.0646. Found 249.0644.

6-methoxypyridin-2-yl morpholine-4-carboxylate (33)

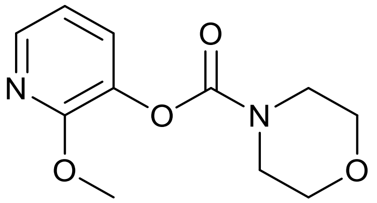

Synthetized using the general procedure at a 1 mmol scale in a Schlenk flask (20 ml, 0.05 M), with 1.1 mmol of morpholine. Isolation with method 2. Reaction time 48 hours. Column run with 1:10 EtOAc:hex for 3 column volumes and then with 1:1 EtOAc:hex + 1 % Et_3N. Product was recovered as colorless oil that slowly solidifies over several days. Yield 59.6 %, 141.9 mg.

^{1}H NMR (400 MHz, $CDCl_3$) δ 7.97 (dd, *J* = 5.0, 1.7 Hz, 1H), 7.34 (dd, *J* = 7.7, 1.7 Hz, 1H), 6.85 (dd, *J* = 7.7, 5.0 Hz, 1H), 3.94 (s, 3H), 3.74 – 3.68 (m, 4H), 3.68 – 3.44 (m, 4H).

^{13}C{^{1}H} NMR (101 MHz, $CDCl_3$) δ 156.7, 152.9, 143.3, 135.4, 130.7, 116.8, 66.6, 66.5, 53.7, 45.1, 44.3.

HRMS-ESI (pos): $[M+Na]^+$ calculated for $C_{11}H_{14}N_2O_4$ 261.0846. Found 261.0833.

11 OTHER SYNTHESIS

Pinzer ligand (N2,N6-bis(2,6-diisopropylphenyl)pyridine-2,6-dicarboxamide) and formation of its nickel(II) complex

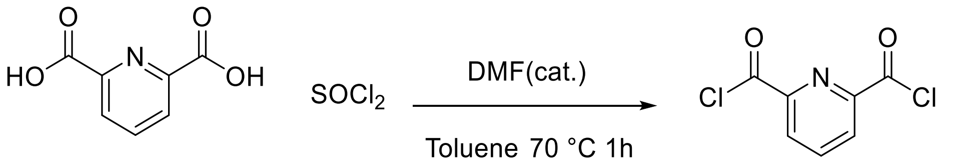

Into a 100 ml 2-neck flask was added 7,06 g (42,2 mmol) of pyridine dicarboxylic acid and dissolved in 30 ml of dry toluene. 1 ml of dry DMF was added as a catalyst. Rubber septum was added to one of the necks and a condenser with tubing connected to saturated Na_2CO_3 was connected to the other. 15,3 ml (211 mmol) of $SOCl_2$ was added dropwise through the septum in to the mixture. After addition, the mixture was heated to 70 °C for 1 h. Afterwards, volatiles were removed and the crude product was used directly in the following reaction.

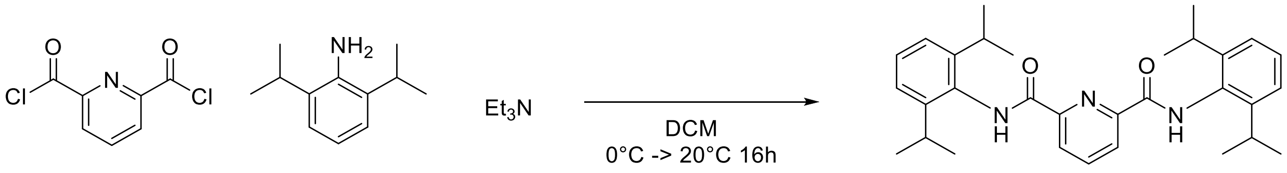

Into a 250 ml round-bottom flask was added 50 ml of dichloromethane, 15,95 ml (84,5 mmol) of 2,6-diisopropyl aniline, and 12,3 ml (89 mmol). The solution was cooled with water-ice bath. Crude diacylchloride pyridine was dissolved in 50 ml of dichloromethane and was slowly added to the amine solution. After addition the mixture was stirred for 15 minutes, then cooling was removed and the mixture was left to room temperature for 16 hours. Afterwards, the mixture was washed with water and brine, dried with Na_2SO_4, filtered and evaporated. Recrystallized from toluene : hexane to give 16,7 g (81 %) of product . NMR matches the reported literature values.[23]

^{1}H NMR (400 MHz, $CDCl_3$) δ 9.03 (s, 2H), 8.59 (d, J = 7.8 Hz, 2H), 8.21 (t, J = 7.8 Hz, 1H), 7.39 (dd, J = 8.4, 7.1 Hz, 2H), 7.27 (d, J = 7.5 Hz, 5H), 3.17 (h, J = 6.9 Hz, 4H), 1.26 (d, J = 6.9 Hz, 23H).

^{13}C{^{1}H} NMR (101 MHz, $CDCl_3$) δ 162.5, 148.9, 146.0, 139.6, 130.6, 128.6, 125.9, 123.7, 29.1, 23.6.

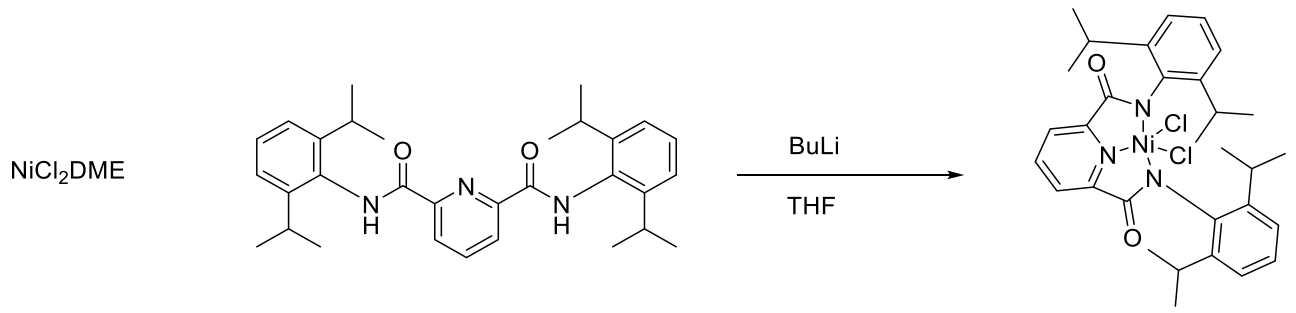

300 mg of the ligand was dissolved in 10 ml of dry THF under argon. The solution was cooled to 0°C. 0,8 ml of butyl lithium was added and the mixture was stirred for 30 minutes. 132 mg of $NiCl_2$DME was dissolved in 5 ml of DMF and then added to the ligand mixture at 0°C. The mixture was stirred at room temperature overnight. Volatiles were then removed and the product was recrystallized from Et_2O to give 174 mg (46 %) of product. NMR matches the reported values in literature.

^{1}H NMR (400 MHz, $CDCl_3$) δ 8.04 (t, J = 7.8 Hz, 1H), 7.76 (d, J = 7.7 Hz, 2H), 7.10 (dd, J = 8.5, 6.7 Hz, 2H), 7.01 (d, J = 7.0 Hz, 4H), 3.92 (h, J = 6.9 Hz, 4H), 1.39 (d, J = 6.9 Hz, 12H), 1.28 (d, J = 6.9 Hz, 12H).

Ligand L1 (N,N,N,N-tetramethyl-[2,2'-bipyridine]-4,4'-diamine)

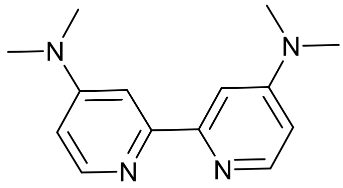

Synthesized from bipyridine with the method described by Ladoucer et al.[24]

^{1}H NMR (400 MHz, $CDCl_3$) δ 8.29 (d, *J* = 5.9 Hz, 2H), 7.68 (d, *J* = 2.7 Hz, 2H), 6.51 (dd, *J* = 5.9, 2.7 Hz, 2H), 3.09 (s, 12H).

Ligand L2 (4,4'-di(pyrrolidin-1-yl)-2,2'-bipyridine)

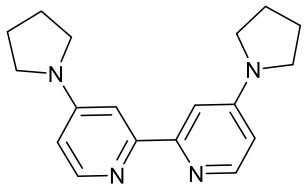

Synthesized from 4,4'-dichloro-2,2'-bipyridine with the method described by De Bon et al.[25]

^{1}H NMR (400 MHz, $CDCl_3$) δ 8.26 (d, *J* = 5.8 Hz, 2H), 7.52 (d, *J* = 2.5 Hz, 2H), 6.38 (dd, *J* = 5.8, 2.5 Hz, 2H), 3.46 – 3.39 (m, 8H), 2.08 – 1.98 (m, 8H).

4-(trifluoromethyl)phenyl butylcarbamate (34)

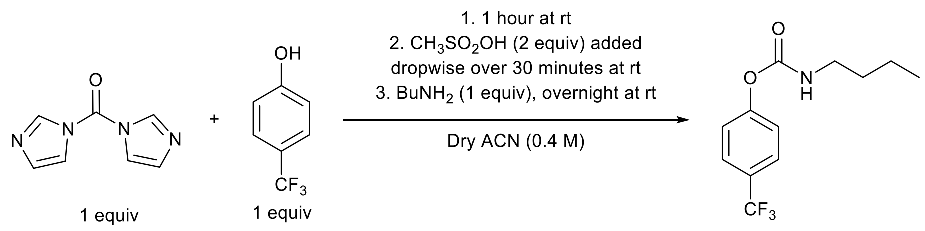

Prepared according to method described by Watson et al.[26] Carbonyldiimidazole (6 mmol, 973 mg) was dissolved in 15 ml of dry acetonitrile in 50 ml flask. The flask was placed in a room temperature water bath to stabilize temperature changes. 4-hydroxybenzotrifluoride (6 mmol, 973 mg) was added and the mixture was stirred for 1 hour at room temperature. Methanesulfonic acid (12 mmol, 780 µl) was added to the mixture dropwise over 30 minutes, while taking care that the internal temperature stayed below 30 °C. White precipitate formed. Butyl amine (6 mmol, 593 µl) was added and the mixture was stirred overnight.

The mixture was transferred to a separatory funnel with 50 ml of EtOAc and the successively washed with 2x50ml H_2O, 50 ml 2M Na_2CO_3, 50 ml H_2O, 50 ml brine. Organic phase was dried with anhydrous Na_2SO_4, filtered and evaporated. The crude product was purified with column

chromatography using 6:1 hexane:EtOAc to yield 1.14 g (73 %) of 4-(trifluoromethyl)phenyl butylcarbamate.

^{1}H NMR (400 MHz, $CDCl_3$) δ 7.67 – 7.61 (m, 2H), 7.31 – 7.25 (m, 2H), 5.06 (s, 1H), 3.31 (td, *J* = 7.1, 7.1, 5.9 Hz, 2H), 1.60 (ddd, *J* = 13.4, 9.2, 6.4 Hz, 2H), 1.44 (dq, *J* = 9.8, 7.3, 7.3, 7.3 Hz, 2H), 0.99 (t, *J* = 7.3, 7.3 Hz, 3H).

1-benzylpiperazine

To a 250 ml Erlenmeyer flask was added 5.43 g of anhydrous piperazine (63 mmol) and 50 ml of absolute ethanol was added to dissolve it. While stirring 5.25 ml of 12 M HCl (63 mmol) was added dropwise and the mixture was heated to 65°C and stirred for 10 minutes. Benzyl chloride was added dropwise, which reacts instantly and causes precipitation of piperazine dihydrochloride hydrate. The mixture is allowed to cool to room temperature.

The precipitated piperazine dihydrochloride hydrate was filtered out dried to give crude hydrochloride salt of the product. The crude product was made basic by adding 50 ml of 2M NaOH solution. The aqueous solution was then extracted with 5x10ml of chloroform. Combined extracts were dried over sodium sulfate. After evaporation of the solvent, the resulting oil was distilled (122-124/2.5 mmHg) to yield 4.03 g (76 %).

^{1}H NMR (400 MHz, $CDCl_3$) δ 7.36 – 7.20 (m, 6H), 3.49 (s, 2H), 2.91 – 2.85 (m, 4H), 2.41 (d, *J* = 5.3 Hz, 4H).

$^{13}C\{^1H\}$ NMR (101 MHz, $CDCl_3$) δ 138.2, 129.3, 128.3, 127.1, 63.8, 54.7, 46.3.

$(PPh_3)_2$Ni-o-tolylBr

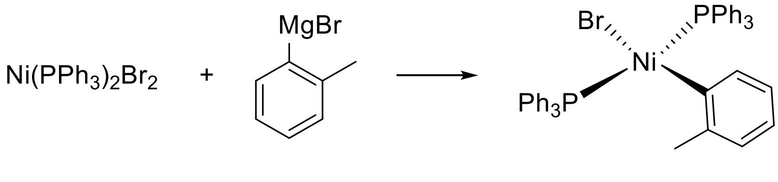

Preparation of o-tolylMgBr: Magnesium turnings (487 mg, 20 mmol) was added to a dried 25 ml two-neck flask. The flask was purged with argon and the necks were connected to reflux condenser and a septum. Anhydrous, degassed THF (5 ml) was added to the flask followed by a small drop of dibromoethane. 2-bromotoluene (1.7 g, 10 mmol) in THF (5 ml) was added dropwise. The mixture was furthers stirred for 1 hour at room temperature. The product solution was titrated two times against iodine (96 and 124 mg) in THF saturated with LiCl, which gave concentration of 0.67 M, 75 % yield.

$Ni(PPh_3)_2Br_2$ (5.15 g, 6.9 mmol) was dissolved in 60 ml of CH_2Cl_2 and cooled to 0°C during which the solution turned from green to red. o-tolylMgBr solution (10.5 ml, 0.67 M, 7.0 mmol) prepared above was added dropwise. The mixture was stirred at 0°C for 30 minutes. Solvent was evaporated in rotary evaporator to give a brown solid. Methanol (50 ml) was added and the mixture was sonicated and then filtered. The resulting yellow solid was washed with cold Et_2O to yield the product $(PPh_3)_2$Ni-o-tolylBr (4.56 g, 87.6 %). NMR matches the reported spectra.[27]

^{1}H NMR (400 MHz, C_6D_6) δ 7.78 (d, *J* = 7.2 Hz, 12H), 7.39 (d, *J* = 7.5 Hz, 1H), 6.99 (s, 18H), 6.43 (t, *J* = 7.5 Hz, 1H), 6.26 (t, *J* = 7.4 Hz, 1H), 6.16 (d, *J* = 7.4 Hz, 1H), 2.44 (s, 3H).

^{31}P NMR (162 MHz, C_6D_6) δ 23.02

^{1}H NMR (400 MHz, $CDCl_3$) δ 7.70 – 7.48 (m, 12H), 7.37 (t, *J* = 7.4 Hz, 6H), 7.29 (d, *J* = 7.6 Hz, 12H), 7.14 (d, *J* = 7.5 Hz, 1H), 6.43 – 6.23 (m, 2H), 5.98 (d, *J* = 7.3 Hz, 1H), 2.21 (s, 3H).

^{13}C{^{1}H} NMR (101 MHz, $CDCl_3$) δ 151.7 (t, *J* = 32.7 Hz), 143.5, 135.9, 134.8 (t, *J* = 5.5 Hz), 132.1 (t, *J* = 21.5 Hz), 129.5, 129.4, 127.7 (t, *J* = 4.9 Hz), 123.0, 122.1, 26.0.

(dtbbpy)Ni-o-tolylBr

$(PPh_3)_2$Ni-o-tolylBr (1.45 g 2 mmol) and 4,4'-di-tert-butyl-2,2'-bipyridine (560 mg, 2.1 mmol) were weighed in a dry 250 ml round-bottom flask. Et_2O (100 ml) was added and the mixture was stirred in room temperature for 3 days. The mixture was filtered and then washed several times with Et_2O and then with pentane to yield an orange product (dtbbpy)Ni-o-tolylBr (0.903 g, 94 %)

^{1}H NMR (500 MHz, CD_2Cl_2) δ 9.25 (d, *J* = 5.9 Hz, 1H), 7.93 – 7.75 (m, 2H), 7.58-7.46 (m, 2H), 7.13 (d, *J* = 6.2 Hz, 1H), 7.03 (d, *J* = 6.2 Hz, 1H), 6.89 – 6.68 (m, 3H), 3.03 (s, 3H), 1.42 (s, 9H), 1.34 (s, 9H).

^{13}C{^{1}H} NMR (126 MHz, CD_2Cl_2) δ 163.8, 162.9, 156.2, 153.1, 150.9, 150.7, 149.6, 142.8, 136.4, 127.5, 123.9, 123.7, 123.2, 122.6, 117.8, 117.1, 35.6, 30.3, 30.1, 25.6.

TMGH$^+$ morpholine-N-carboxylate

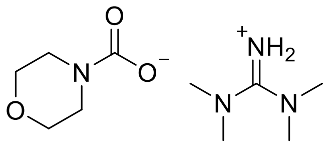

Morpholine (1.7 g, 20 mmol) and tetramethylguanadine (2.4g, 21 mmol) was added to a 50 ml round bottom flask and diluted with 20 ml of diethyl ether. The solution was flushed with carbon dioxide and white crystalline precipitate formed. The mixture was stirred under carbon dioxide for further 30 minutes and then it was filtered. The filtrate was washed with diethyl ether to yield 4.3 g (88 % yield)

of white crystalline solid, which was stored in inert gas glovebox. Slow decomposition was observed over several days, when the product was stored in a vial open to air, likely due to hydrolysis.

^{1}H NMR (400 MHz, $CDCl_3$) δ 8.74 (s, 1H), 3.52 (s, 4H), 3.33 (s, 4H), 2.84 (s, 12H).

^{13}C{^{1}H} NMR (101 MHz, $CDCl_3$) δ 163.6, 162.3, 67.5, 45.0, 39.5.

o-tolyl morpholine-4-carboxylate

Prepared according to method described by Watson et al.[26] Carbonyldiimidazole (10 mmol, 1.6 g) was dissolved in 20 ml of dry acetonitrile in 100 ml flask. The flask was placed in a room temperature water bath to stabilize temperature changes. o-cresol (10 mmol, 1.07 g) was added and the mixture was stirred for 1 hour at room temperature. Methanesulfonic acid (20 mmol, 1.3 ml) was added to the mixture dropwise over 30 minutes. White precipitate formed. Morpholine (10 mmol, 870 μl) was added and the mixture was stirred overnight.

The mixture was transferred to a separatory funnel with 50 ml of EtOAc and the successively washed with 2x50ml H_2O, 50 ml 2M Na_2CO_3, 50 ml H_2O, 50 ml brine. Organic phase was dried with anhydrous Na_2SO_4, filtered and evaporated. The crude product was purified with column chromatography using 4:1 hexane:EtOAc to yield 1.73g (78 %) of o-tolyl morpholine-4-carboxylate.

^{1}H NMR (400 MHz, $CDCl_3$) δ 7.27 – 7.19 (m, 2H), 7.15 (td, J = 7.3, 1.3 Hz, 1H), 7.09 (dd, J = 7.8, 1.4 Hz, 1H), 3.81 – 3.75 (m, 4H), 3.73 (s, 2H), 3.61 (s, 2H), 2.24 (s, 3H).

^{13}C{^{1}H} NMR (101 MHz, $CDCl_3$) δ 153.4, 149.7, 131.0, 130.2, 126.8, 125.7, 122.1, 66.7, 44.9, 44.2, 16.1.

12 CALCULATION OF THE REDUCTION POTENTIALS OF THE PHOTOCATALYSTS

The excited state reduction potential, E(PC*/PC^{-}), can be calculated with equation 1.

$$E(PC^{*}/PC^{-}) = E(PC/PC^{-}) + E_{00}^{s} \tag{1}$$

Where E^{s}_{00} is the energy of the vibrationally relaxed first excited singlet state in electron volts. This energy can be estimated from the absorption and fluorescence maxima of the first excited singlet state.[28] The absorption and fluorescence maxima that correspond to the S_1 should be adjacent and have the highest wavelengths (lowest energy). Equation 2 was used for the calculations of redox potentials.

$$E(PC^*/PC^-) = E(PC/PC^-) + (\frac{hc}{\frac{\lambda_{A,max} + \lambda_{F,max}}{2}}) \cdot (\frac{1\,eV}{1.602177 \cdot 10^{-19} J}) \quad 2$$

Where h is the Planck's constant, c is the speed of light and $\lambda_{A,max}$ and $\lambda_{F,max}$ are the absorbance and fluorescence maxima corresponding to the S_1 state respectively.

In Table S18 is collected the values determined from absorption and fluorescence spectra as well as cyclic voltammetry experiments shown in sections 13.

Table S18. Tabulated values for the synthetized photocatalysts.

Photocatalyst	Measured in[a]	Absorption maximum (nm)	Fluorescence maximum (nm)	$E(PC/PC^-)$ (V vs SCE)	$E(PC^*/PC^-)$[c] (V vs SCE)
4DPAPN	ACN	442	572	-1.54	0.90
4DPAIPN	ACN	470	522	-1.66	0.84
4DPATPN	DMF	519	573	- 1.45	0.82
4DPAPN-OMe	DMF	504	543	-1.64	0.73
4DPAIPN-OMe	ACN	497	577	-1.80	0.51
4DPATPN-OMe	DMF	566	628	-1.54	0.54
4DPAPN-^{t}Bu	ACN	463	595	-1.60	0.74
4DPAIPN-^{t}Bu	ACN	486	555	-1.67	0.71
4DPATPN-^{t}Bu	DMF	543	604	-1.36	0.80
4DPAPN-Ph	ACN	474	602	-1.44	0.87
4DPAIPN-Ph	ACN	489	548	-1.56	0.83
4DPATPN-Ph	DMF	539	603	-1.49[b]	0.69[b]

Both the absorption and fluorescence maxima are the ones corresponding to the S_1 state. In the fluorescence studies, excitation wavelength of 455 nm, corresponding to blue light, was used. All potentials are measured in acetonitrile solution and are referenced against SCE by setting Fc^+/Fc at 0.38 V.[a] The PC:s are less soluble in ACN than DMF but ACN is more stable so wider voltammograms (PC:s donor ability) could be recorded and therefore ACN was preferred. [b] Very rough estimate because 4DPATPN-Ph is too insoluble even in DMF to give a sufficiently concentrated solution to yield a good voltammogram. [c]Calculated with equation 2.

13 ABSORPTION- AND FLUORESCENCE SPECTRA AND VOLTAMMOGRAMS

Cyclic voltammetry (CV) measurements were performed as follows: Photocatalyst and ferrocene (Fc, sublimed under atmospheric pressure) were dissolved (both 1 mM) using an electrolyte solution (0.2 M Bu_4NPF_6 in acetonitrile or DMF pre-dried with 3Å molecular sieves and distilled over CaH_2). Autolab PGSTAT 20 potentiostat along with ALS SVC-3 voltammetry cell with glassy carbon working electrode (WE) with 3 mm diameter, platinum wire counter electrode (CE) and Ag/Ag^+ (0.01 M $AgNO_3$ in electrolyte solution) reference electrode (RE) were used. The electrochemical cell was degassed by bubbling nitrogen for about 10 minutes. A pre-bubbler was used to avoid concentration and possible precipitation of the photocatalyst.[1] The scan rate was set to 50 mV/s with a 10 mV step potential. In most scans, the scan area vs SCE (saturated calomel electrode) was –2.6 - 2.9 V with ACN or -2.1 – 1.4 V with DMF (with ACN, some solvent reactions was observed at around +2 V). Only one scan per photocatalyst was run. Between runs, the system was thoroughly rinsed with distilled ACN or DMF, the WE was re-polished using 0.05 µm polishing alumina if necessary and a background run (2 cycles at 100 mV/s) was run. The voltammograms were rescaled vs SCE by setting Fc^+/Fc at 0.38 V.[2] The reversible reaction at around -1.5 V vs SCE visible for all photocatalysts was taken to be the PC/PC^- conversion.

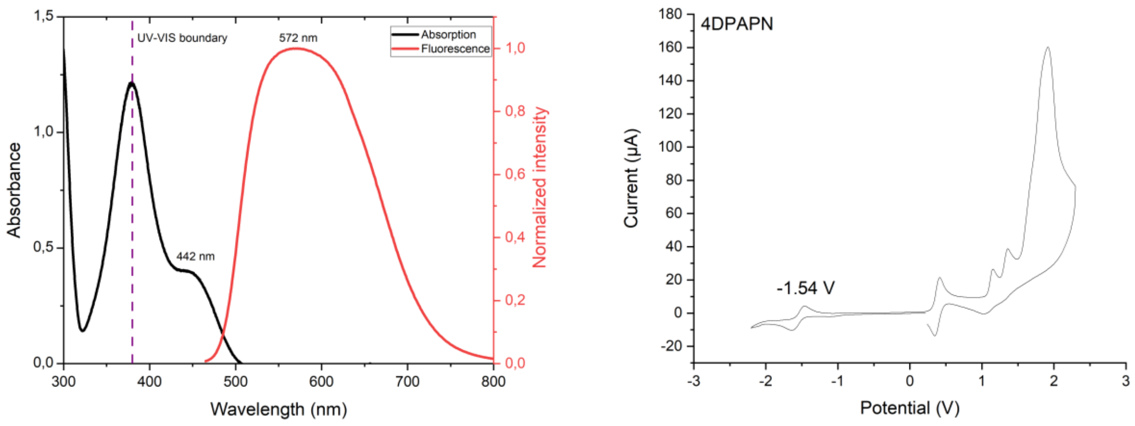

Figure S10. Absorption- and fluorescence spectra and voltammogram of 4DPAPN. Excitation with 450 nm light. Voltammogram recorded in ACN. The voltammogram is given in IUPAC convention. Measurement was performed with glassy carbon working electrode, platinum wire counter electrode and Ag/Ag^+ (0.01 M $AgNO_3$ in electrolyte solution) reference electrode. The measurement was carried out at room temperature at 50 mV/s with 10 mV step potential in degassed 1 mM photocatalyst solution using 0.2 M Bu_4NPF_6 as electrolyte and ferrocene (1 mM) as internal standard. The measurement was started at open potential (0.217 V) into the direction of negative potentials. Only one cycle was recorded. The voltammogram was rescaled vs SCE by setting Fc/Fc^+ at 0.38 V.

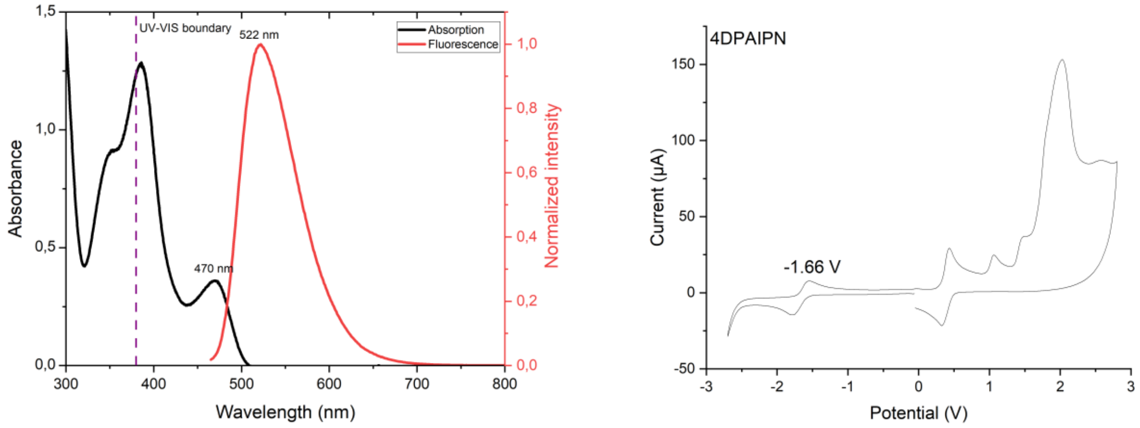

Figure S11. Absorption- and fluorescence spectra and voltammogram of 4DPAIPN. Excitation with 450 nm light. Voltammogram recorded in ACN. The voltammogram is given in IUPAC convention. Measurement was performed with glassy carbon working electrode (internal diameter 3 mm), platinum wire counter electrode and Ag/Ag^+ (0.01 M $AgNO_3$ in electrolyte solution) reference electrode. The measurement was carried out at room temperature at 50 mV/s with 10 mV step potential in degassed 1 mM photocatalyst solution using 0.2 M Bu_4NPF_6 as electrolyte and ferrocene (1 mM) as internal standard. The measurement was started at open circuit potential (0.06 V) into the direction of negative potentials. Only one cycle was recorded. The voltammogram was rescaled vs SCE by setting Fc/Fc^+ at 0.38 V.

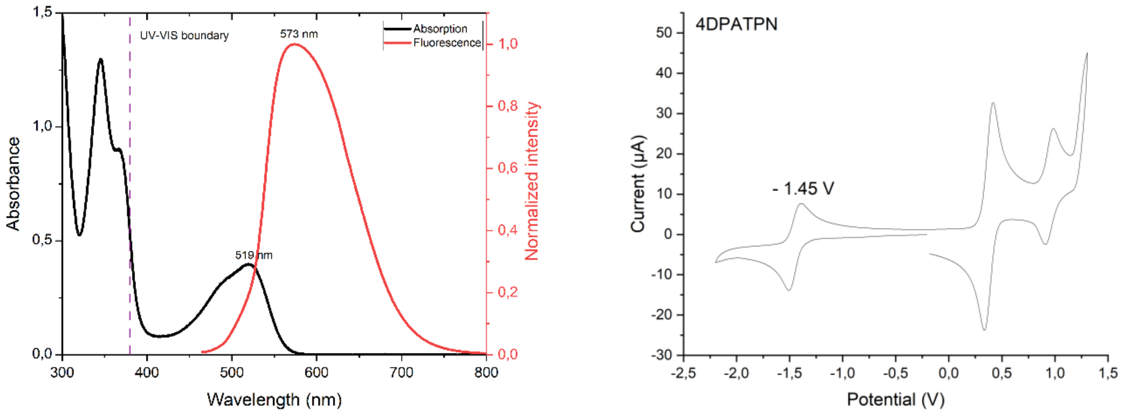

Figure S12. Absorption- and fluorescence spectra and voltammogram of 4DPATPN. Excitation with 450 nm light. Voltammogram recorded in DMF. The voltammogram is given in IUPAC convention. Measurement was performed with glassy carbon working electrode (internal diameter 3 mm), platinum wire counter electrode and Ag/Ag^+ (0.01 M $AgNO_3$ in electrolyte solution) reference electrode. The measurement was carried out at room temperature at 50 mV/s with 10 mV step potential in degassed 1 mM photocatalyst solution using 0.2 M Bu_4NPF_6 as electrolyte and ferrocene (1 mM) as internal standard. The measurement was started at open circuit potential (0.209 V) into the direction of negative potentials. Only one cycle was recorded. The voltammogram was rescaled vs SCE by setting Fc/Fc^+ at 0.38 V.

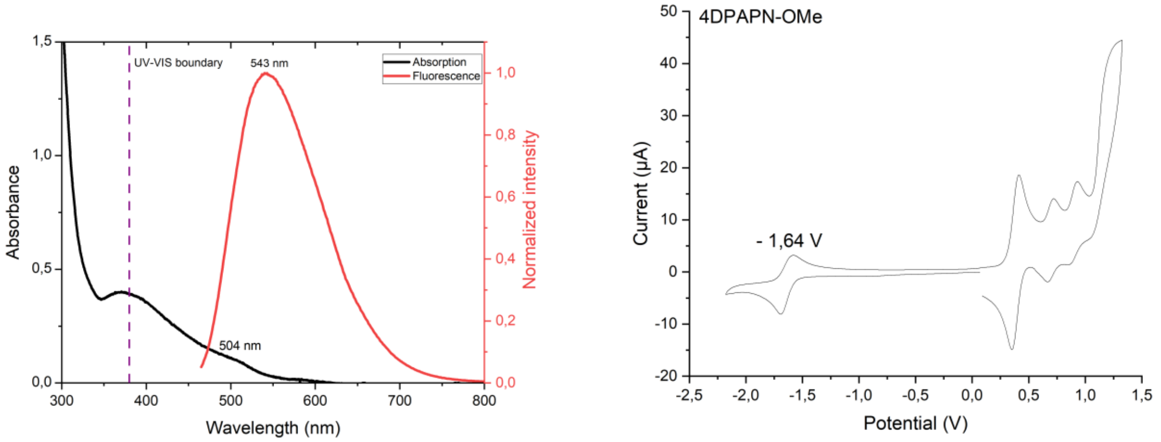

Figure S13. Absorption- and fluorescence spectra and voltammogram of 4DPAPN-OMe. Excitation with 450 nm light. Voltammogram recorded in DMF. The voltammogram is given in IUPAC convention. Measurement was performed with glassy carbon working electrode (internal diameter 3 mm), platinum wire counter electrode and Ag/Ag^+ (0.01 M $AgNO_3$ in electrolyte solution) reference electrode. The measurement was carried out at room temperature at 50 mV/s with 10 mV step potential in degassed 1 mM photocatalyst solution using 0.2 M Bu_4NPF_6 as electrolyte and ferrocene (1 mM) as internal standard. The measurement was started at open circuit potential (-0.09 V) into the direction of negative potentials. Only one cycle was recorded. The voltammogram was rescaled vs SCE by setting Fc/Fc^+ at 0.38 V.

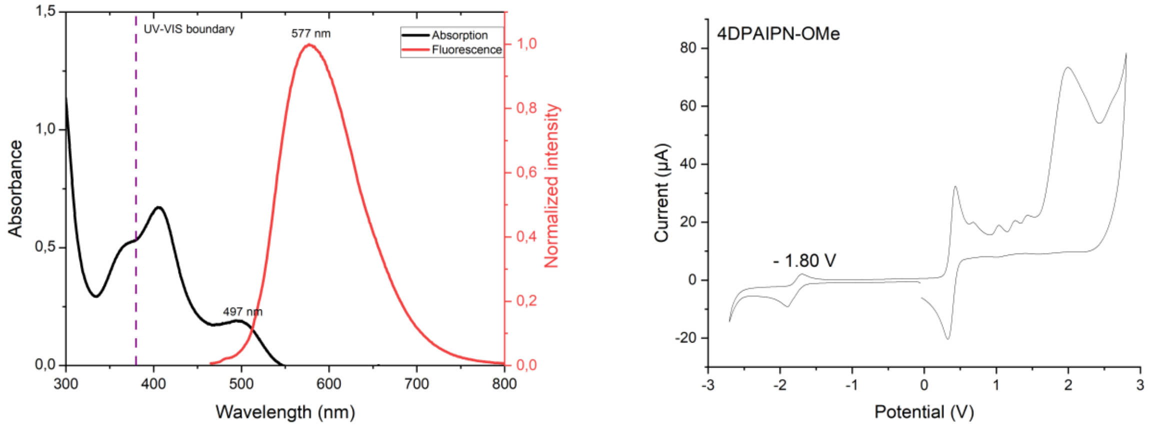

Figure S14. Absorption- and fluorescence spectra and voltammogram of 4DPAIPN-OMe. Excitation with 450 nm light. Voltammogram recorded in ACN. The voltammogram is given in IUPAC convention. Measurement was performed with glassy carbon working electrode (internal diameter 3 mm), platinum wire counter electrode and Ag/Ag^+ (0.01 M $AgNO_3$ in electrolyte solution) reference electrode. The measurement was carried out at room temperature at 50 mV/s with 10 mV step potential in degassed 1 mM photocatalyst solution using 0.2 M Bu_4NPF_6 as electrolyte and ferrocene (1 mM) as internal standard. The measurement was started at open circuit potential (-0.05 V) into the direction of negative potentials. Only one cycle was recorded. The voltammogram was rescaled vs SCE by setting Fc/Fc^+ at 0.38 V.

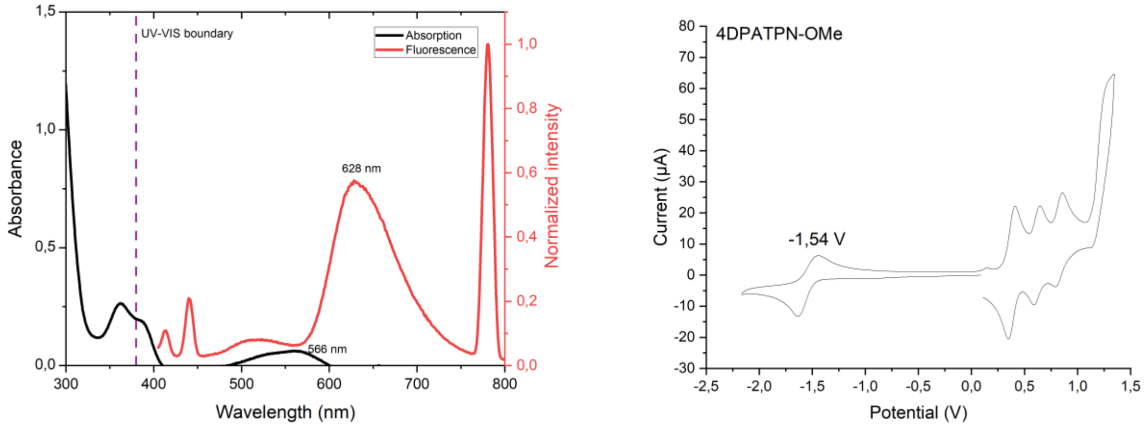

Figure S15. Absorption- and fluorescence spectra and voltammogram of 4DPATPN-OMe. Excitation with 390 nm light. Voltammogram recorded in DMF. The voltammogram is given in IUPAC convention. Measurement was performed with glassy carbon working electrode (internal diameter 3 mm), platinum wire counter electrode and Ag/Ag^{+} (0.01 M $AgNO_3$ in electrolyte solution) reference electrode. The measurement was carried out at room temperature at 50 mV/s with 10 mV step potential in degassed 1 mM photocatalyst solution using 0.2 M Bu_4NPF_6 as electrolyte and ferrocene (1 mM) as internal standard. The measurement was started at open circuit potential (-0.04 V) into the direction of negative potentials. Only one cycle was recorded. The voltammogram was rescaled vs SCE by setting Fc/Fc^{+} at 0.38 V.

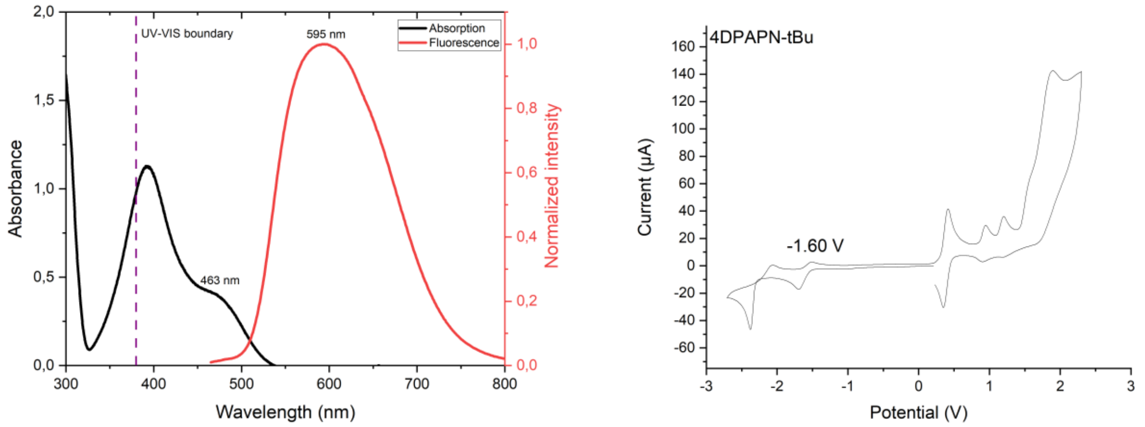

Figure S16. Absorption- and fluorescence spectra and voltammogram of 4DPAPN-tBu. Excitation with 450 nm light. Voltammogram recorded in ACN. The voltammogram is given in IUPAC convention. Measurement was performed with glassy carbon working electrode (internal diameter 3 mm), platinum wire counter electrode and Ag/Ag^{+} (0.01 M $AgNO_3$ in electrolyte solution) reference electrode. The measurement was carried out at room temperature at 50 mV/s with 10 mV step potential in degassed 1 mM photocatalyst solution using 0.2 M Bu_4NPF_6 as electrolyte and ferrocene (1 mM) as internal standard. The measurement was started at open circuit potential (0.209 V) into the direction of negative potentials. Only one cycle was recorded. The voltammogram was rescaled vs SCE by setting Fc/Fc^{+} at 0.38 V.

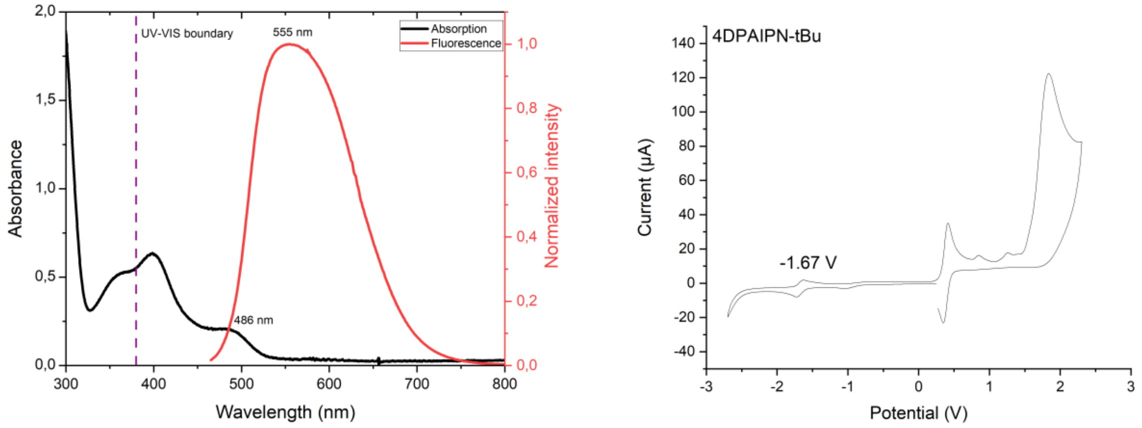

Figure S17. Absorption- and fluorescence spectra and voltammogram of 4DPAIPN-tBu. Excitation with 450 nm light. Voltammogram recorded in ACN. The voltammogram is given in IUPAC convention. Measurement was performed with glassy carbon working electrode (internal diameter 3 mm), platinum wire counter electrode and Ag/Ag^+ (0.01 M $AgNO_3$ in electrolyte solution) reference electrode. The measurement was carried out at room temperature at 50 mV/s with 10 mV step potential in degassed 1 mM photocatalyst solution using 0.2 M Bu_4NPF_6 as electrolyte and ferrocene (1 mM) as internal standard. The measurement was started at open circuit potential (0.252 V) into the direction of negative potentials. Only one cycle was recorded. The voltammogram was rescaled vs SCE by setting Fc/Fc^+ at 0.38 V.

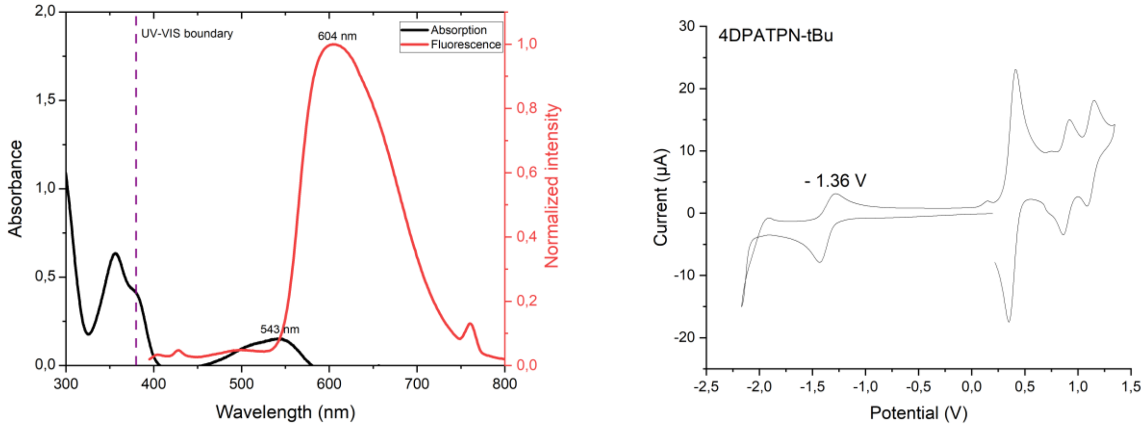

Figure S18. Absorption- and fluorescence spectra and voltammogram of 4DPATPN-tBu. Excitation with 380 nm light. Voltammogram recorded in DMF. The voltammogram is given in IUPAC convention. Measurement was performed with glassy carbon working electrode (internal diameter 3 mm), platinum wire counter electrode and Ag/Ag^+ (0.01 M $AgNO_3$ in electrolyte solution) reference electrode. The measurement was carried out at room temperature at 50 mV/s with 10 mV step potential in degassed 1 mM photocatalyst solution using 0.2 M Bu_4NPF_6 as electrolyte and ferrocene (1 mM) as internal standard. The measurement was started at open circuit potential (0.178 V) into the direction of negative potentials. Only one cycle was recorded. The voltammogram was rescaled vs SCE by setting Fc/Fc^+ at 0.38 V.

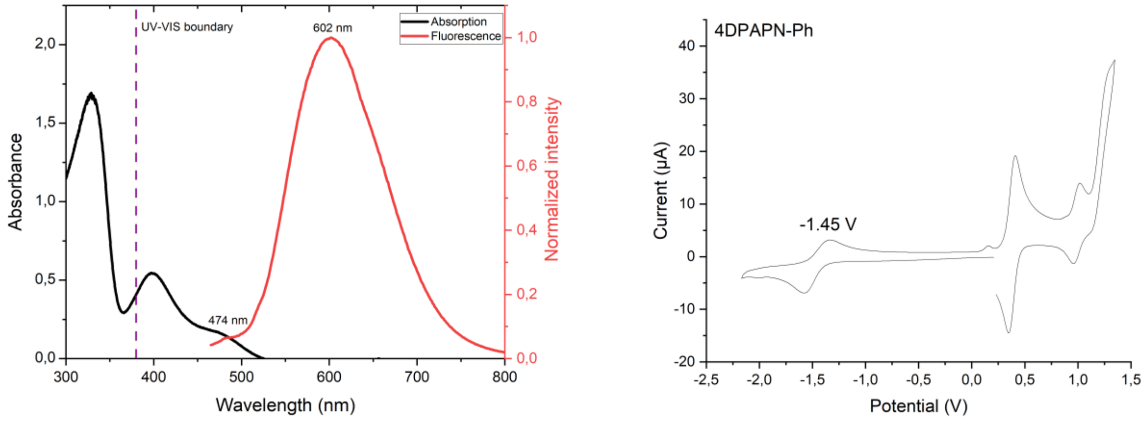

Figure S19. Absorption- and fluorescence spectra and voltammogram of 4DPAPN-Ph. Excitation with 450 nm light. Voltammogram recorded in DMF. The voltammogram is given in IUPAC convention. Measurement was performed with glassy carbon working electrode (internal diameter 3 mm), platinum wire counter electrode and Ag/Ag^+ (0.01 M $AgNO_3$ in electrolyte solution) reference electrode. The measurement was carried out at room temperature at 50 mV/s with 10 mV step potential in degassed 1 mM photocatalyst solution using 0.2 M Bu_4NPF_6 as electrolyte and ferrocene (1 mM) as internal standard. The measurement was started at open circuit potential (0.017 V) into the direction of negative potentials. Only one cycle was recorded. The voltammogram was rescaled vs SCE by setting Fc/Fc^+ at 0.38 V.

.

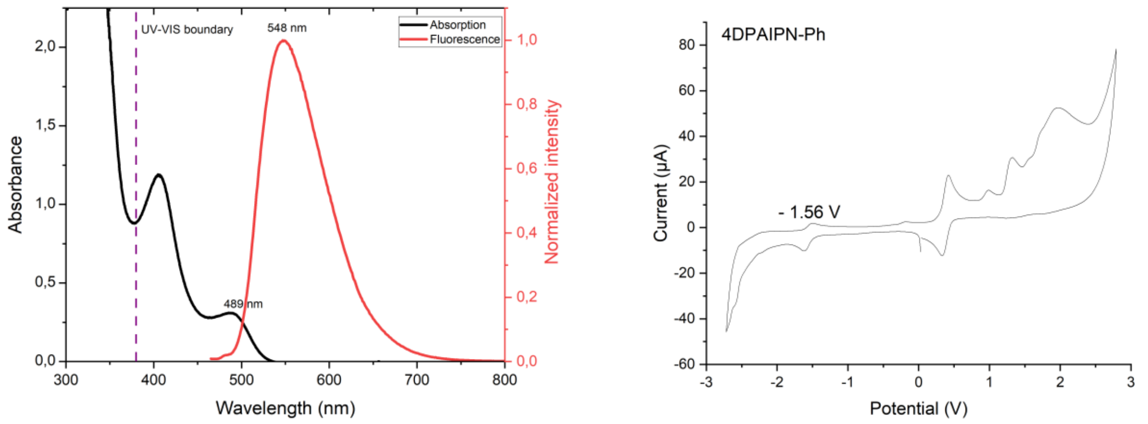

Figure S20. Absorption- and fluorescence spectra and voltammogram of 4DPAIPN-Ph. Excitation with 450 nm light. Voltammogram recorded in ACN. The voltammogram is given in IUPAC convention. Measurement was performed with glassy carbon working electrode (internal diameter 3 mm), platinum wire counter electrode and Ag/Ag^+ (0.01 M $AgNO_3$ in electrolyte solution) reference electrode. The measurement was carried out at room temperature at 50 mV/s with 10 mV step potential in degassed 1 mM photocatalyst solution using 0.2 M Bu_4NPF_6 as electrolyte and ferrocene (1 mM) as internal standard. The measurement was started at open circuit potential (0.028 V) into the direction of negative potentials. Only one cycle was recorded. The voltammogram was rescaled vs SCE by setting Fc/Fc^+ at 0.38 V.

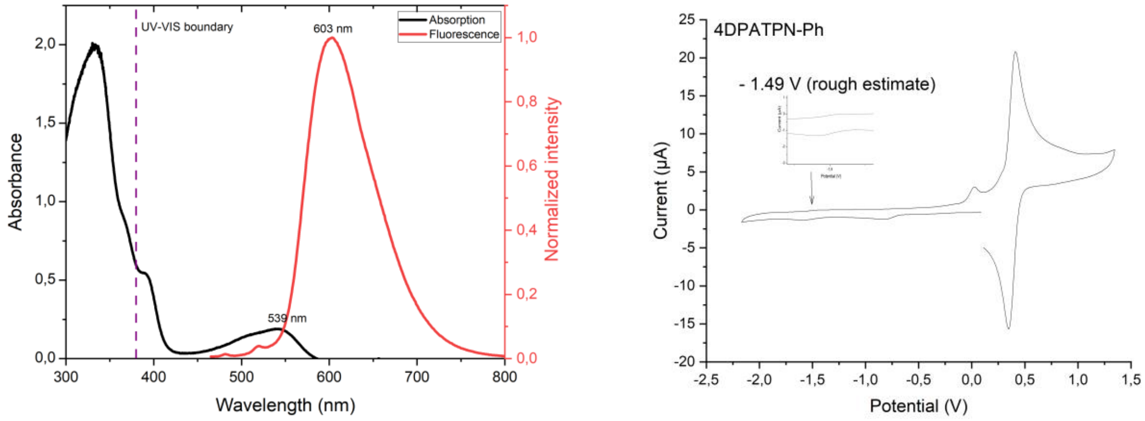

Figure S21. Absorption- and fluorescence spectra and voltammogram of 4DPATPN-Ph. Excitation with 450 nm light. Voltammogram recorded in DMF. The voltammogram is given in IUPAC convention. Measurement was performed with glassy carbon working electrode (internal diameter 3 mm), platinum wire counter electrode and Ag/Ag^+ (0.01 M $AgNO_3$ in electrolyte solution) reference electrode. The measurement was carried out at room temperature at 50 mV/s with 10 mV step potential in degassed 1 mM photocatalyst solution using 0.2 M Bu_4NPF_6 as electrolyte and ferrocene (1 mM) as internal standard. The measurement was started at open circuit potential (0.083 V) into the direction of negative potentials. Only one cycle was recorded. The voltammogram was rescaled vs SCE by setting Fc/Fc^+ at 0.38 V.

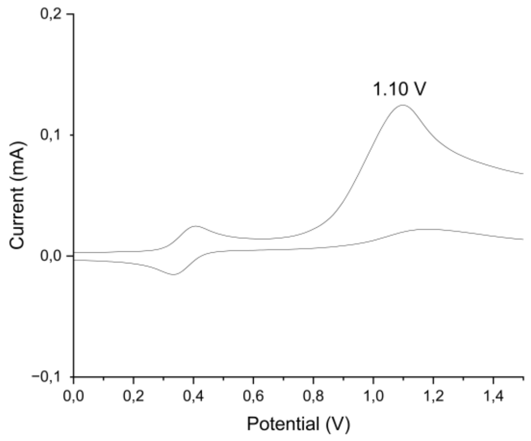

Figure S22. Voltammogram of 0.01 M morpholine in DMF. The voltammogram is given in IUPAC convention. Measurement was performed with glassy carbon working electrode (internal diameter 3 mm), platinum wire counter electrode and Ag/Ag^+ (0.01 M $AgNO_3$ in electrolyte solution) reference electrode. The measurement was carried out at room temperature at 50 mV/s with 10 mV step potential in degassed 0.01 M morpholine solution using 0.1 M Bu_4NPF_6 as electrolyte and ferrocene (1 mM) as internal standard. The measurement was started at +1.98 V into the direction of negative potentials. Only one cycle was recorded. The voltammogram was rescaled vs SCE by setting Fc/Fc^+ at 0.38 V.

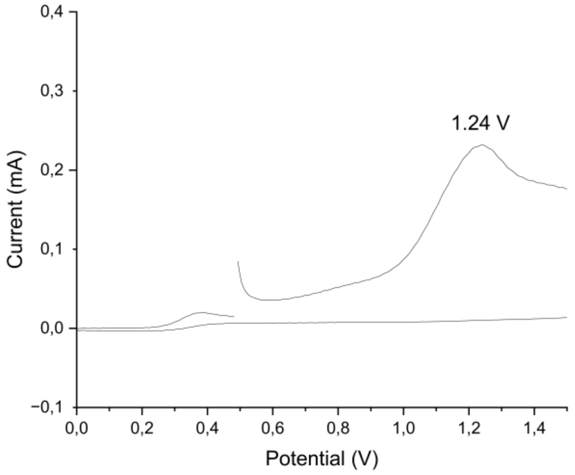

Figure S23: Voltammogram of 0.1 M TMG in DMF. The voltammogram is given in IUPAC convention. Measurement was performed with glassy carbon working electrode (internal diameter 3 mm), platinum wire counter electrode and Ag/Ag^+ (0.01 M $AgNO_3$ in electrolyte solution) reference electrode. The measurement was carried out at room temperature at 50 mV/s with 10 mV step potential in degassed 0.1 M TMG solution using 0.1 M Bu_4NPF_6 as electrolyte and ferrocene (1 mM) as internal standard. The measurement was started at open circuit potential (0.492 V) into the direction of negative potentials. Only one cycle was recorded. The voltammogram was rescaled vs SCE by setting Fc/Fc^+ at 0.38 V.

14 Reaction kinetics

14.1 Procedure

Into an 8 ml vial was added 100ul of each stock solutions of 0.5 M morpholine + 0,5 M TMG (0.2 mmol), 0.5 M p-iodobenzotrifluoride (0.2 mmol), 0.01 M $NiBr_2$dtbbpy (0.01 mmol), 0.5 mM DPAPN-tbu (0.2 umol) and 0.25 M hexafluorobenzene (0.1 mmol) that were dissolved in DMF. The solution was diluted to 7 ml with dry DMF and the vial was sealed with a septum. The solution was bubbled with carbon dioxide for 10 seconds, and then the vial was irradiated as described previously. 0.4 ml samples were taken periodically to study the reaction profile and the reaction progress was measured by ^{19}F-NMR. The concentrations of reagents were then changed to study the rate orders of the reaction.

For $Ni(COD)_2$ experiments: In argon filled glovebox, 2.8±0.1 mg of $Ni(COD)_2$ (0.01 mmol) and 2.7±0.1 mg of dtbbpy (0.01 mmol) was weighed in to a 8 ml vial. 2 ml of DMF and stirrer bar was added and the mixture was stirred for a minute. 29.4 ul of p-iodobenzotrifluoride (0.2 mmol) was added and the mixture was stirred for a minute. Degassed stock solutions of the morpholine + TMG, DPAPN-tBu (only for Figure S 54) and hexafluorobenzene were added as above. Then the solution was diluted to 7 ml and flushed with CO_2.

For a reaction of type

$$aA + bB => P$$

the reaction rate for the formation of P is

$$rate_p = k[A]^n\,[B]^m$$

where [A] is the concentration of reagent A, n is the reaction order of A, and k is the reaction rate coefficient. After taking a logarithm we can determine reaction order n from linear fit from measurements of the reaction rate in different concentrations of A, when all the other variables are kept constant.

$$\log rate_P = n\log[A] + \log k + m\log[B]$$

$$\log rate_P = n\log[A] + b$$

Usually, the initial rate is measured because at that point the concentrations of all the reagents are known. However, this reaction has an activation period that causes a sigmoidal reaction profile. We wanted to study the carbamate forming reaction instead of the activation reaction. Therefore, the fastest rate of the reaction (steepest part of the curve) was chosen as the reaction rate for the rate order analysis. At this point some of the reagents have been consumed and therefore the concentrations are not strictly known. However, since the reaction profile and the selectivities are very similar in all experiments and the maximum rate is generally achieved after 10% conversion into product, the reaction order can still be determined, but the error margin is larger.

14.2 Linear fit of product formation to reagent concentration

The logarithm of the highest reaction rate was plotted against the logarithm of the concentration of the reagent for each of the reagents. The result was that the carbamate has zero order relationship to product formation or any of the side reactions. The iodobenzotrifluoride is first order. The nickel seems to have non-linear relation. This is likely because the nickel needs to be initially activated by the photocatalyst and then subsequently excited by the photocatalyst in each step of the cycle. The unactive nickel can likely quench the photocatalyst and therefore competes with the active nickel, which results a hill like rate curve. The photocatalyst has 0.4-0.6 order relation to the product and side product formation. This is likely because the photocatalyst is not needed to all of the product forming catalytic cycles, but there are some important cycles where it catalyses the reaction. With increased amount of photocatalyst the time required for nickel activation becomes shorter. This activation period becomes shorter even after 1% of photocatalyst, even though the maximum rate is already achieved with 0.5% of photocatalyst.

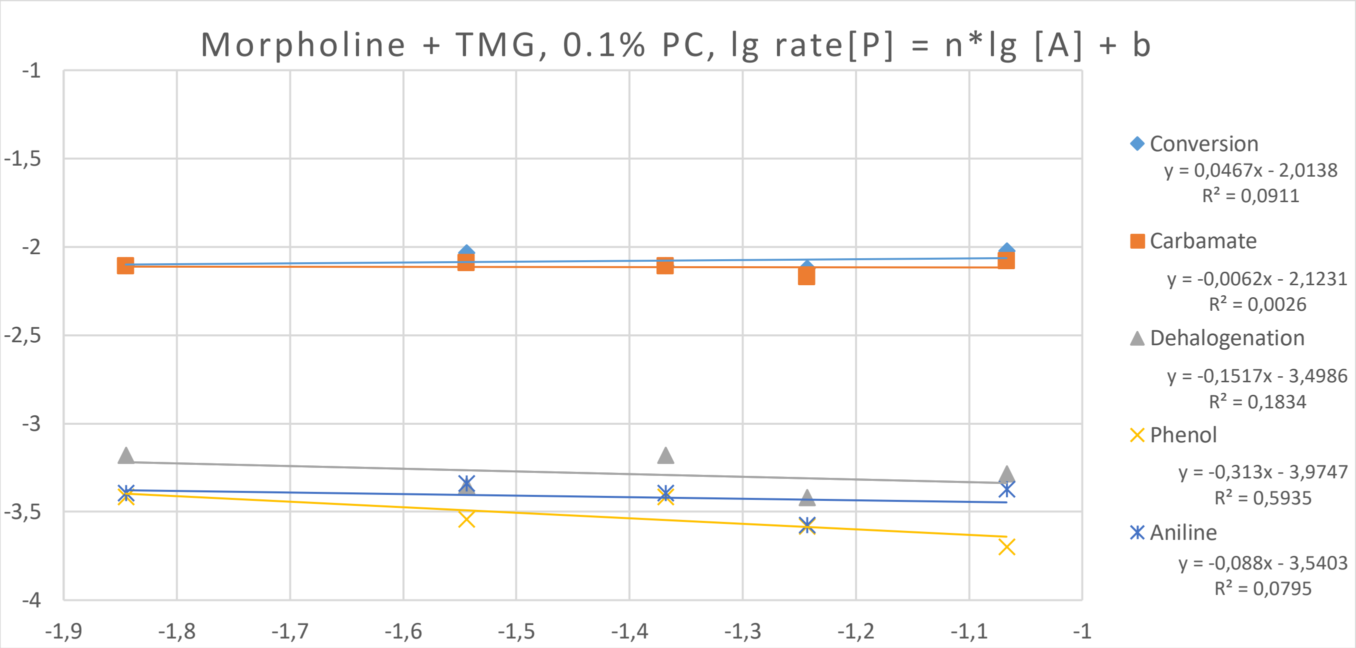

Figure S 24: Effect of amine and TMG concentration to the rate order of the reactions. 0.2 mmol iodobenzotrifluoride, 0.01 mmol $NiBr_2$dtbbpy, 0.2 µmol DPAPN-tbu, 0.1, 0.2, 0.3, 0.4 or 0.6 mmol morpholine and TMG, diluted to 7 ml in DMF. The rate is in lg mmol/h and the concentration is in lg mol/l

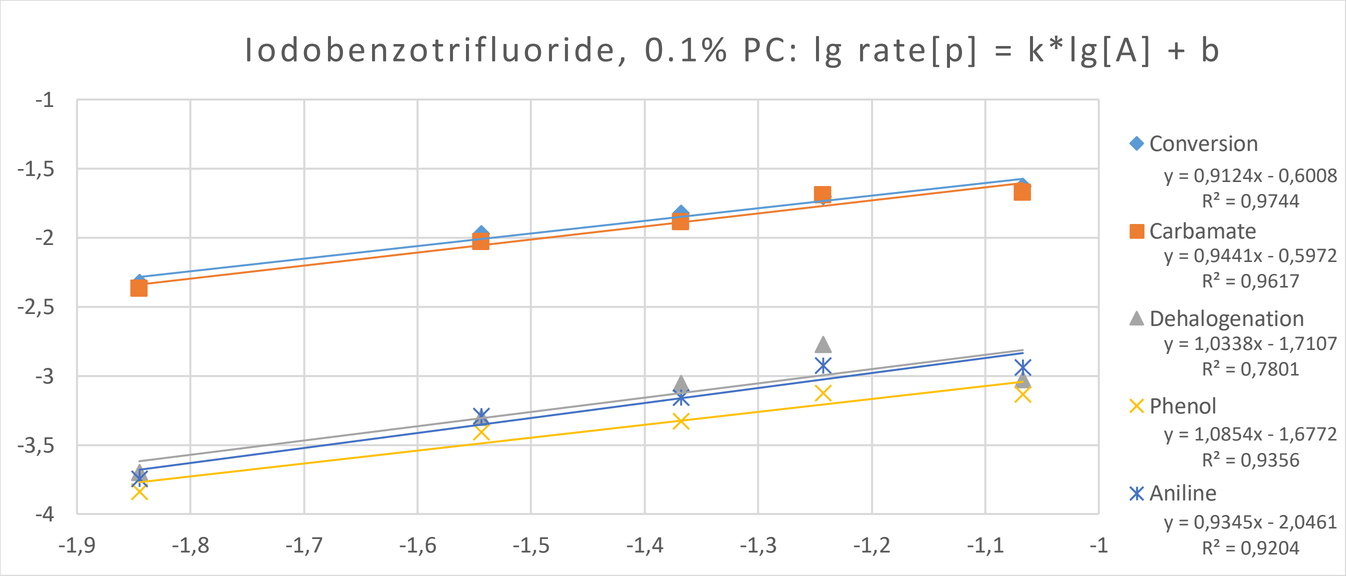

Figure S 25: Effect of iodobenzotrifluoride concentration to the rate order of the reactions. 0.2 mmol morpholine, 0.2 mmol TMG, 0.01 mmol $NiBr_2$dtbbpy, 0.2 µmol DPAPN-tbu, 0.1, 0.2, 0.3, 0.4 or 0.6 mmol iodobenzotrifluoride, diluted to 7 ml in DMF. The rate is in lg mmol/h and the concentration is in lg mol/l

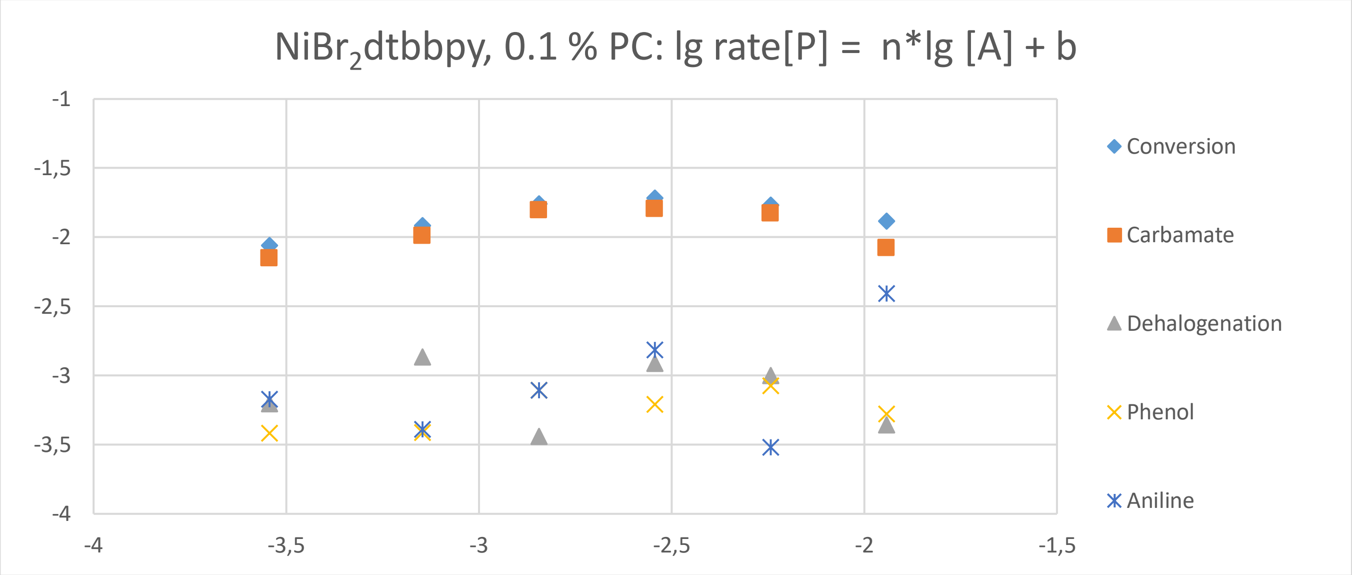

Figure S 26: Effect of $NiBr_2$dtbbpy concentration to the rate order of the reactions. 0.2 mmol morpholine, 0.2 mmol TMG, 0.2mmol iodobenzotrifluoride 0.2 µmol DPAPN-tbu, 0.002, 0.005, 0.01, 0.02, 0.04 or 0.08 mmol $NiBr_2$dtbbpy, diluted to 7 ml in DMF. The rate is in lg mmol/h and the concentration is in lg mol/l. Non- linear correlation is likely caused by photocatalyst becoming the limiting reagent

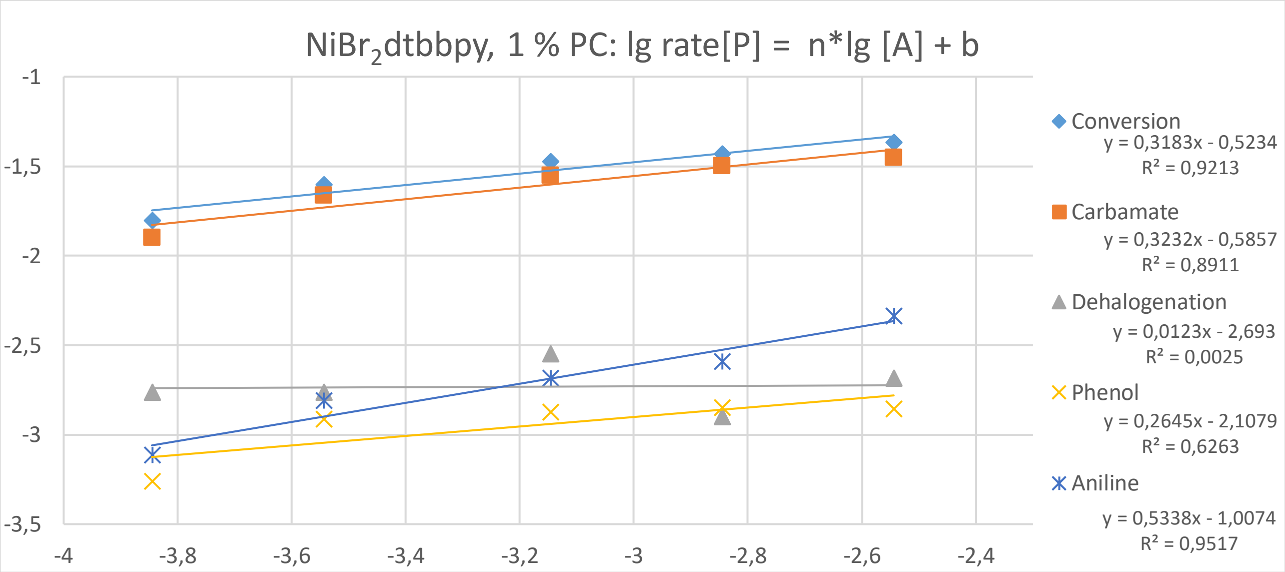

Figure S 27: Effect of $NiBr_2$dtbbpy concentration to the rate order of the reactions, with 1% photocatalyst to avoid saturation. 0.2 mmol morpholine, 0.2 mmol TMG, 0.2mmol iodobenzotrifluoride 2 µmol DPAPN-tbu, 0.002, 0.005, 0.01, 0.02, 0.04 or 0.08 mmol $NiBr_2$dtbbpy, diluted to 7 ml in DMF. The rate is in lg mmol/h and the concentration is in lg mol/l.

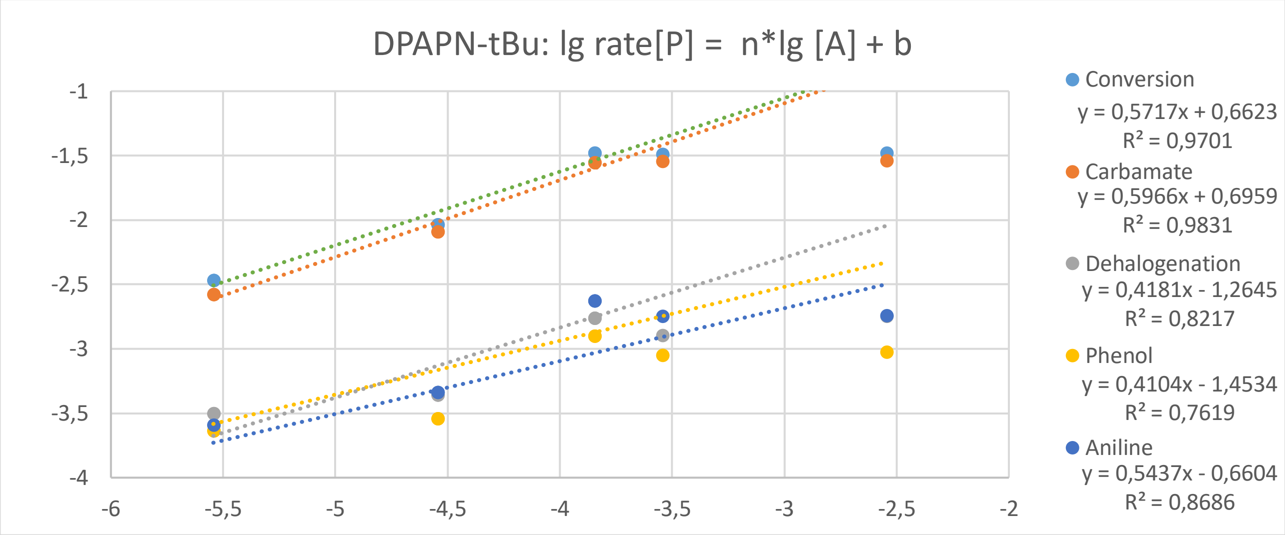

Figure S 28: Effect of DPAPN-tBu concentration to the rate order of the reactions. 0.2 mmol morpholine, 0.2 mmol TMG, 0.2mmol iodobenzotrifluoride 0.01 mmol $NiBr_2$dtbbpy, 0.02, 0.2, 1, 2 or 20 µmol DPAPN-tbu, diluted to 7 ml in DMF. The rate is in lg mmol/h and the concentration is in lg mol/l. Only the first three points were selected for the linear fit, as the rate becomes saturated at higher concentrations

14.3 Reaction profiles

The title of each figure shows the equivalents of each reagent morpholine + tetramehylguanidine : p-iodobenzotrifluoride : $NiBr_2$dtbbpy : DPAPN-tBu. 1 equivalent equals 0.2 mmol, 28.6 mM. The y-axis is the ^{19}F NMR yield and the x-axis is time in minutes.

To an 8ml vial was added

14.3.1 Morpholine and TMG

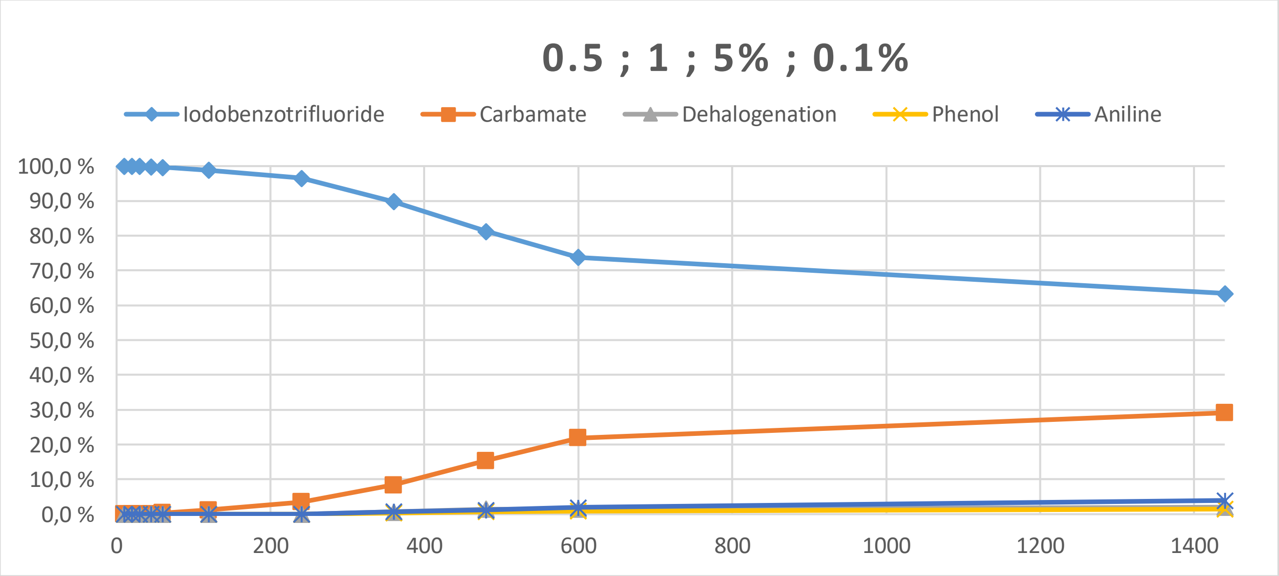

Figure S 29: Conversion of starting material and formation of different products over time (in minutes). The ratio of amine + TMG : p-iodobenzotrifluoride : $NiBr_2$dtbbpy : DPAPN-tBu is shown in the title.

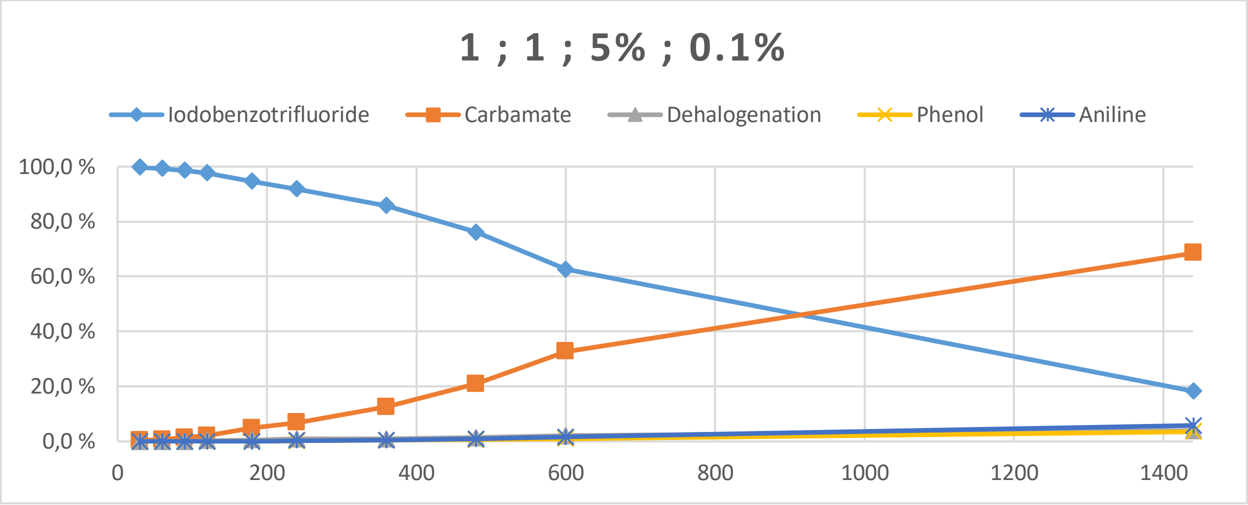

Figure S 30: Conversion of starting material and formation of different products over time (in minutes). The ratio of amine + TMG : p-iodobenzotrifluoride : $NiBr_2dtbbpy$: DPAPN-tBu is shown in the title.

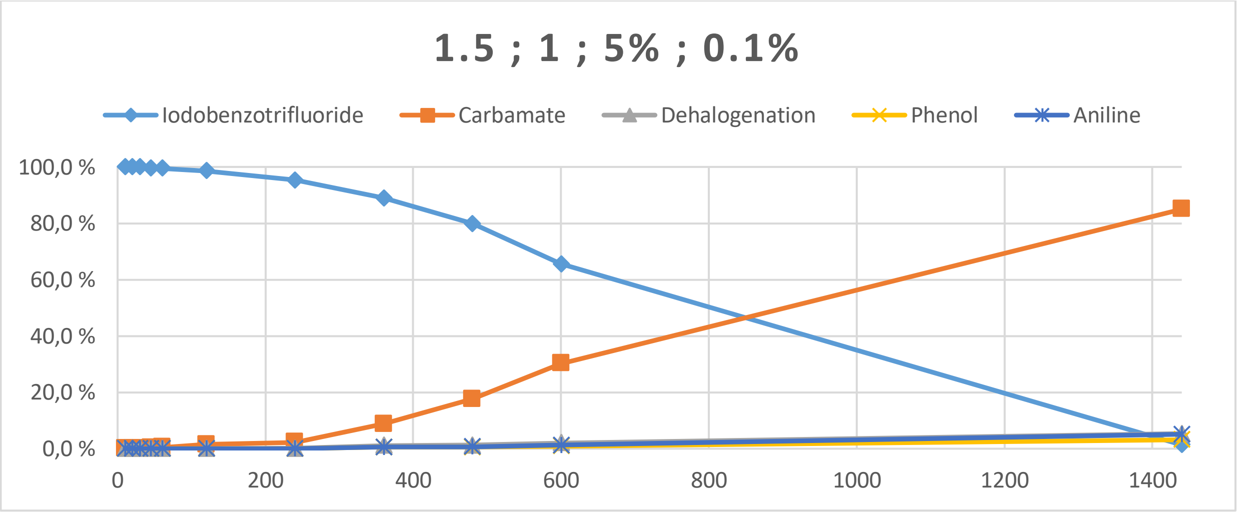

Figure S 31: Conversion of starting material and formation of different products over time (in minutes). The ratio of amine + TMG : p-iodobenzotrifluoride : $NiBr_2dtbbpy$: DPAPN-tBu is shown in the title.

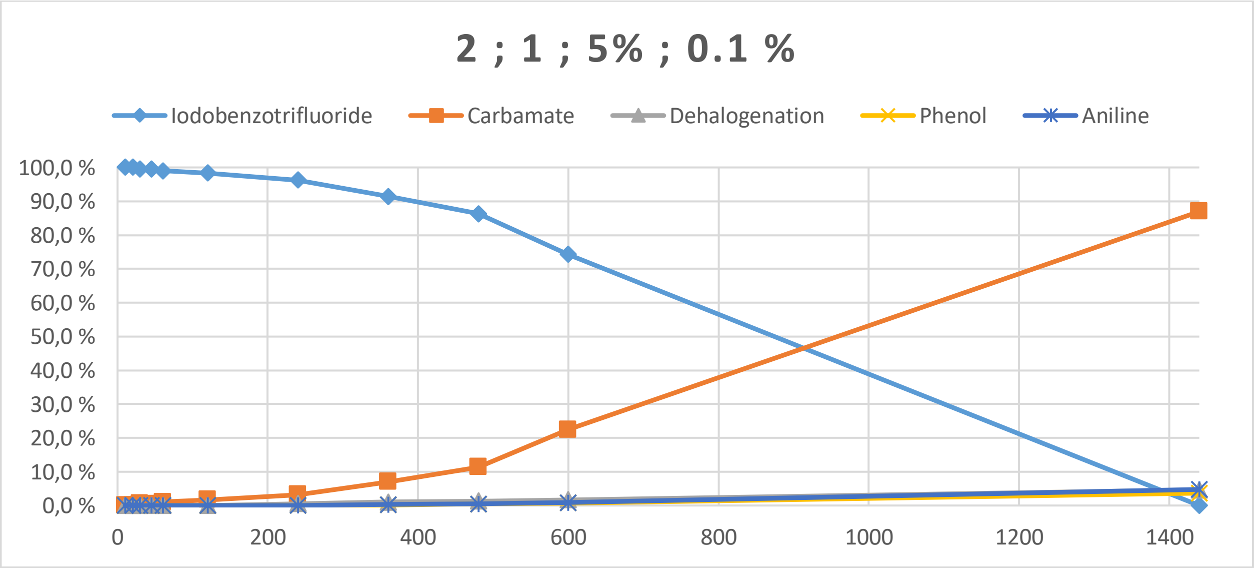

Figure S 32: Conversion of starting material and formation of different products over time (in minutes). The ratio of amine + TMG : p-iodobenzotrifluoride : $NiBr_2dtbbpy$: DPAPN-tBu is shown in the title.

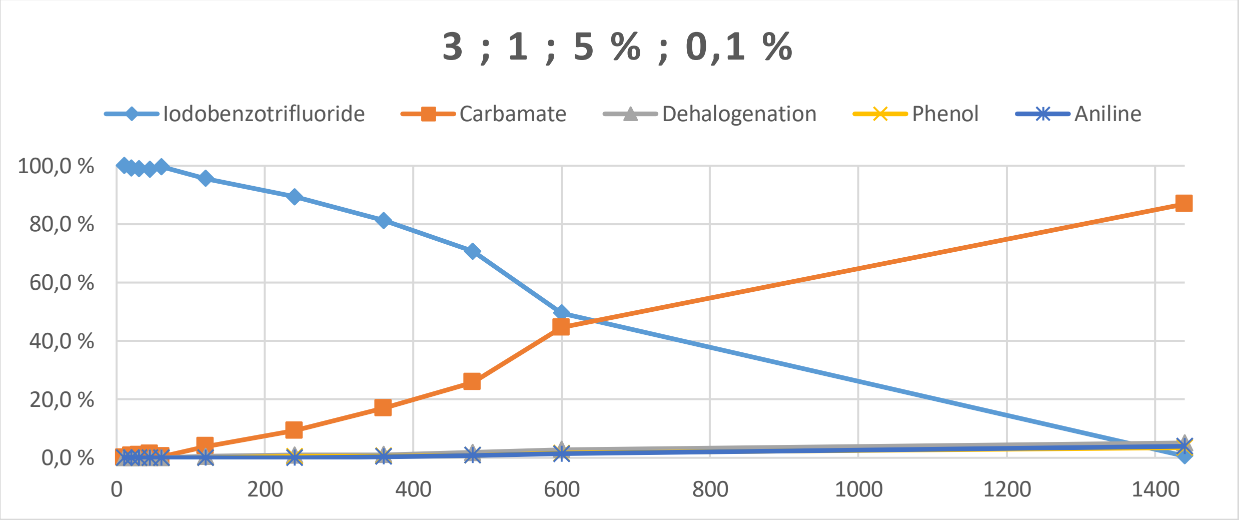

Figure S 33: : Conversion of starting material and formation of different products over time (in minutes). The ratio of amine + TMG : p-iodobenzotrifluoride : $NiBr_2$dtbbpy : DPAPN-tBu is shown in the title.

14.3.2 Iodobenzotrifluoride

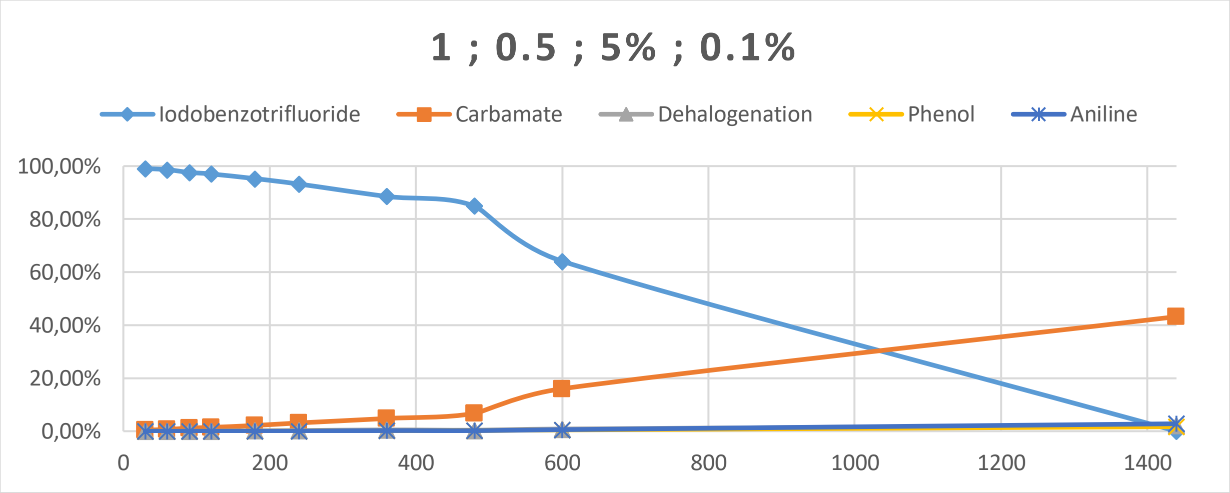

Figure S 34: Conversion of starting material and formation of different products over time (in minutes). The ratio of amine + TMG : p-iodobenzotrifluoride : $NiBr_2$dtbbpy : DPAPN-tBu is shown in the title.

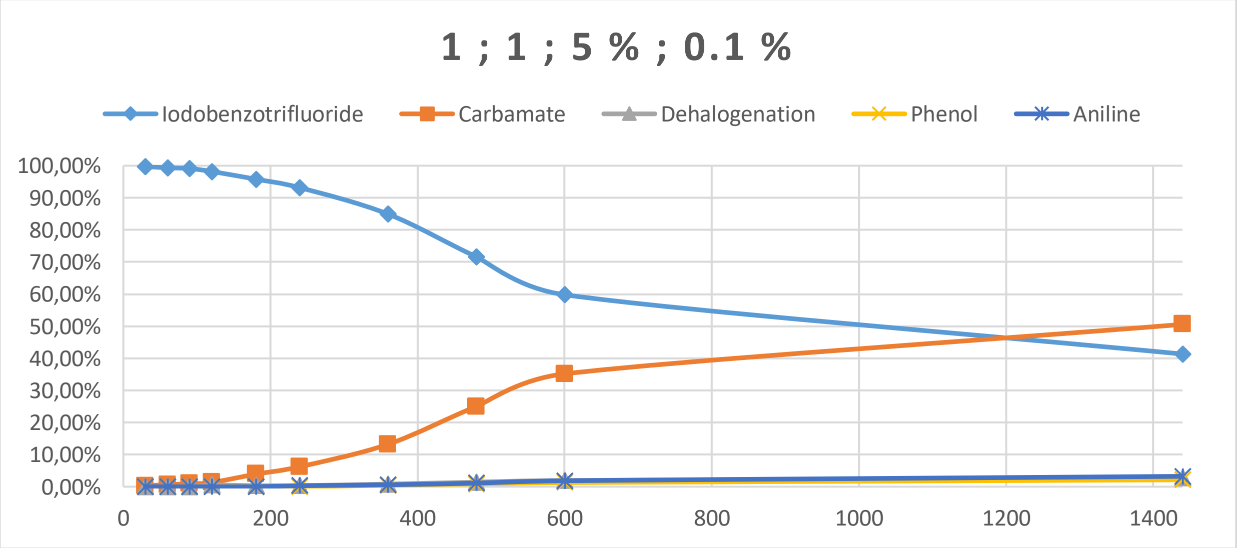

Figure S 35: Conversion of starting material and formation of different products over time (in minutes). The ratio of amine + TMG : p-iodobenzotrifluoride : $NiBr_2$dtbbpy : DPAPN-tBu is shown in the title.

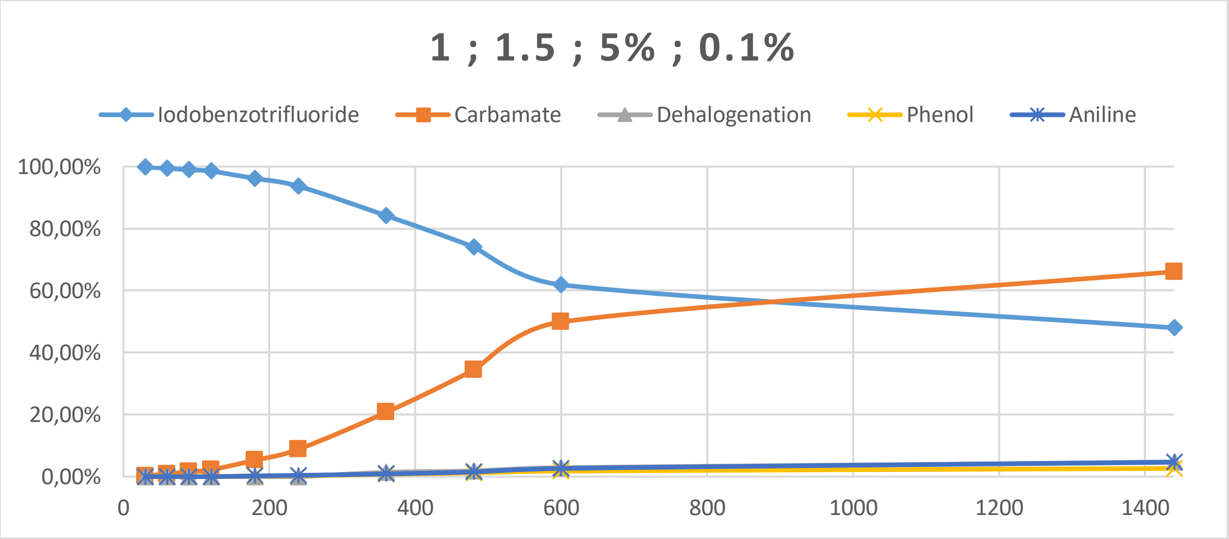

Figure S 36: Conversion of starting material and formation of different products over time (in minutes). The ratio of amine + TMG : p-iodobenzotrifluoride : $NiBr_2$dtbbpy : DPAPN-tBu is shown in the title.

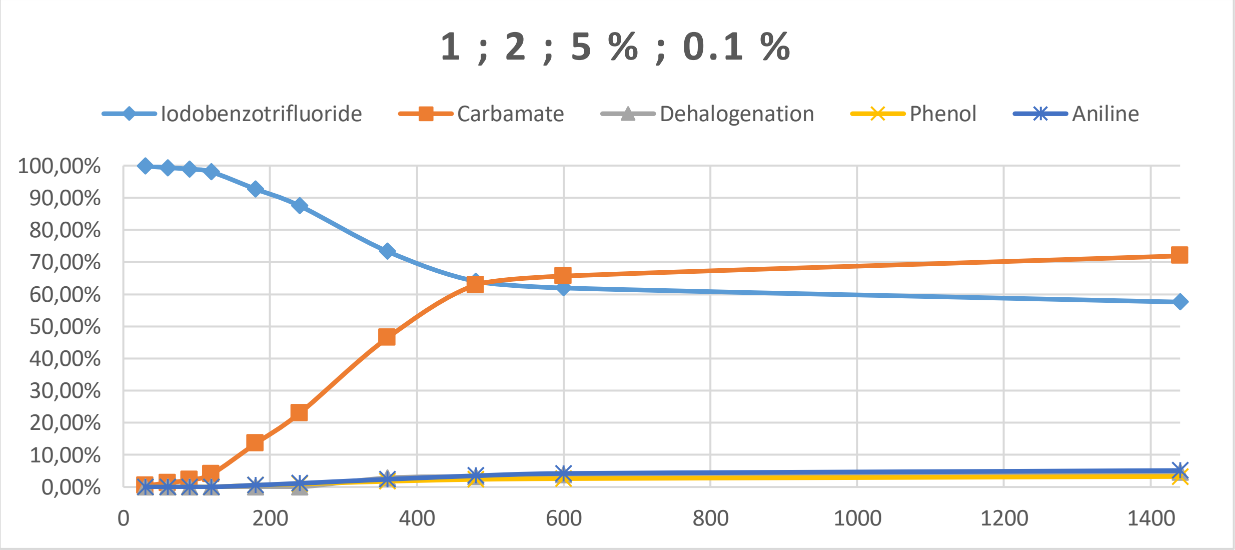

Figure S 37: Conversion of starting material and formation of different products over time (in minutes). The ratio of amine + TMG : p-iodobenzotrifluoride : $NiBr_2$dtbbpy : DPAPN-tBu is shown in the title.

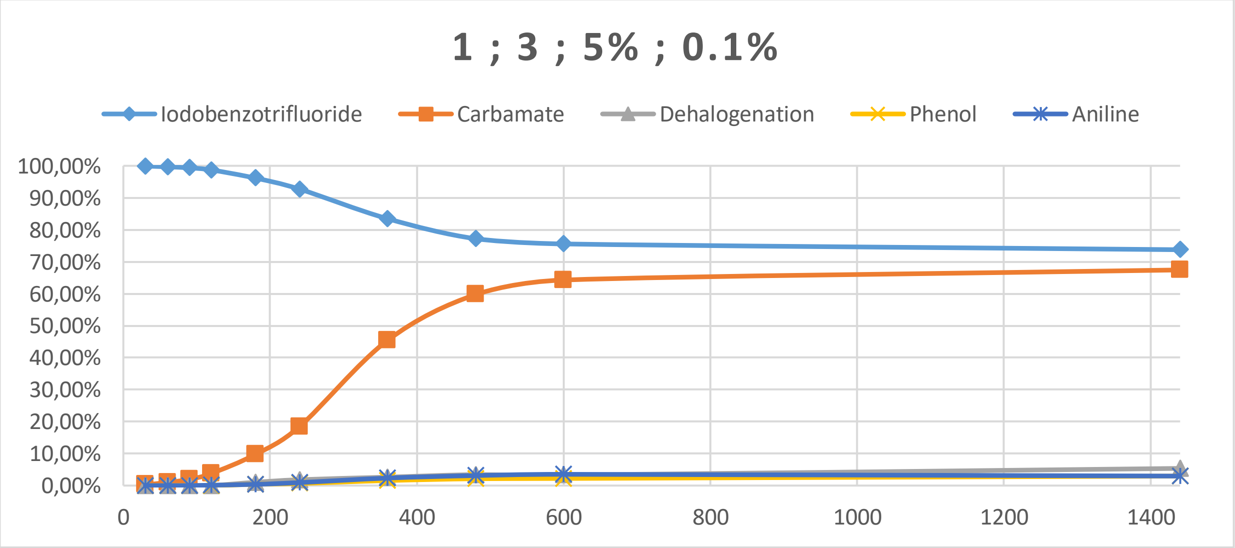

Figure S 38: Conversion of starting material and formation of different products over time (in minutes). The ratio of amine + TMG : p-iodobenzotrifluoride : $NiBr_2$dtbbpy : DPAPN-tBu is shown in the title.

14.3.3 $NiBr_2dtbbpy$

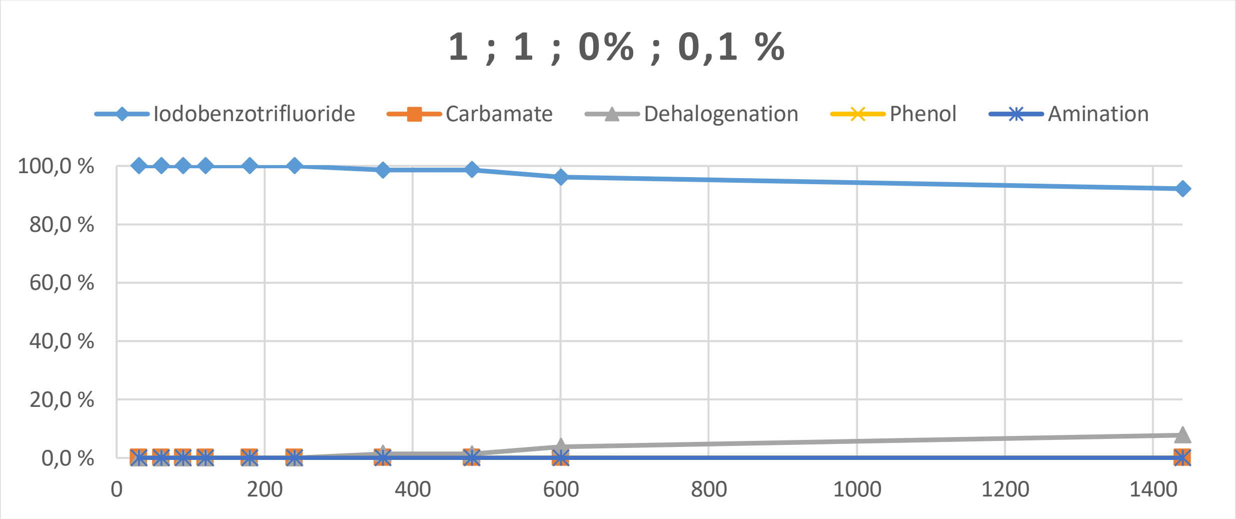

Figure S 39: Conversion of starting material and formation of different products over time (in minutes). The ratio of amine + TMG : p-iodobenzotrifluoride : $NiBr_2dtbbpy$: DPAPN-tBu is shown in the title.

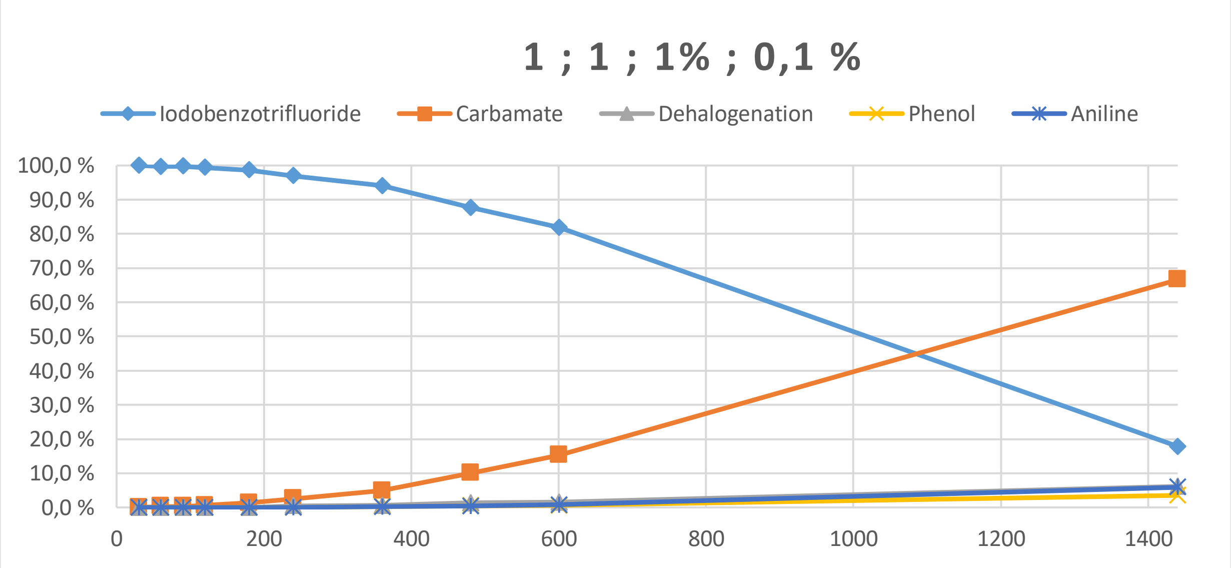

Figure S 40: Conversion of starting material and formation of different products over time (in minutes). The ratio of amine + TMG : p-iodobenzotrifluoride : $NiBr_2dtbbpy$: DPAPN-tBu is shown in the title.

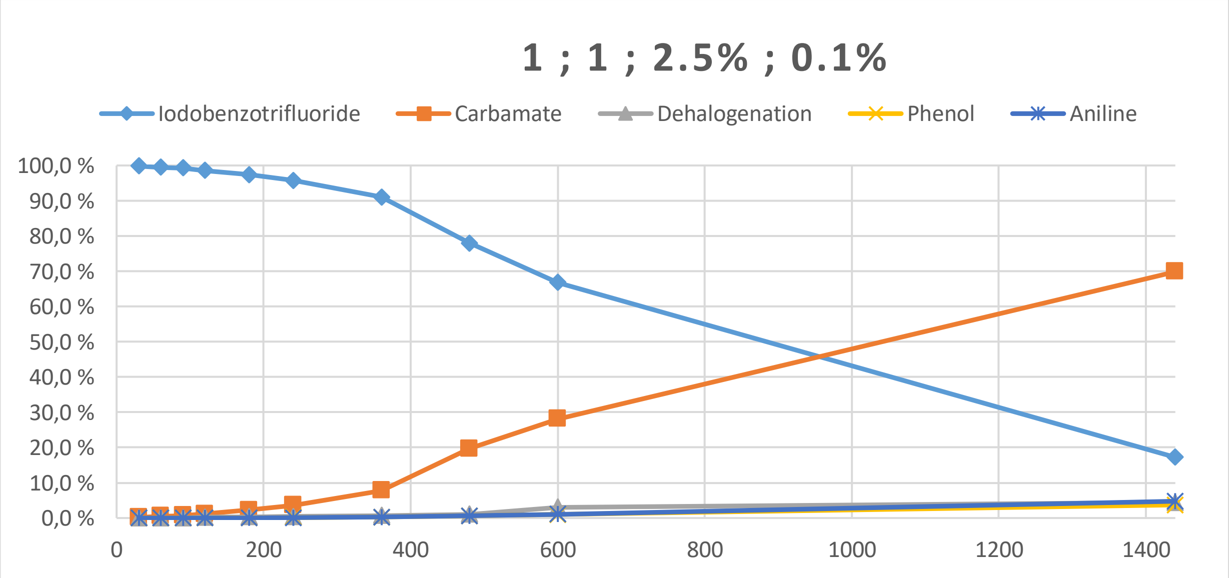

Figure S 41: Conversion of starting material and formation of different products over time (in minutes). The ratio of amine + TMG : p-iodobenzotrifluoride : $NiBr_2dtbbpy$: DPAPN-tBu is shown in the title.

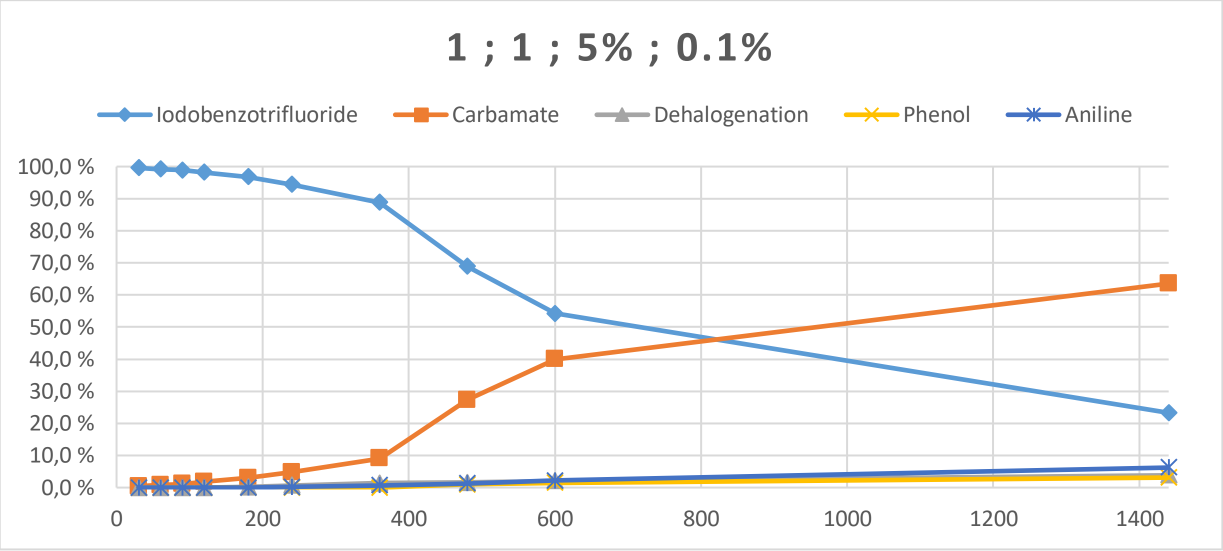

Figure S 42: Conversion of starting material and formation of different products over time (in minutes). The ratio of amine + TMG : p-iodobenzotrifluoride : $NiBr_2$dtbbpy : DPAPN-tBu is shown in the title.

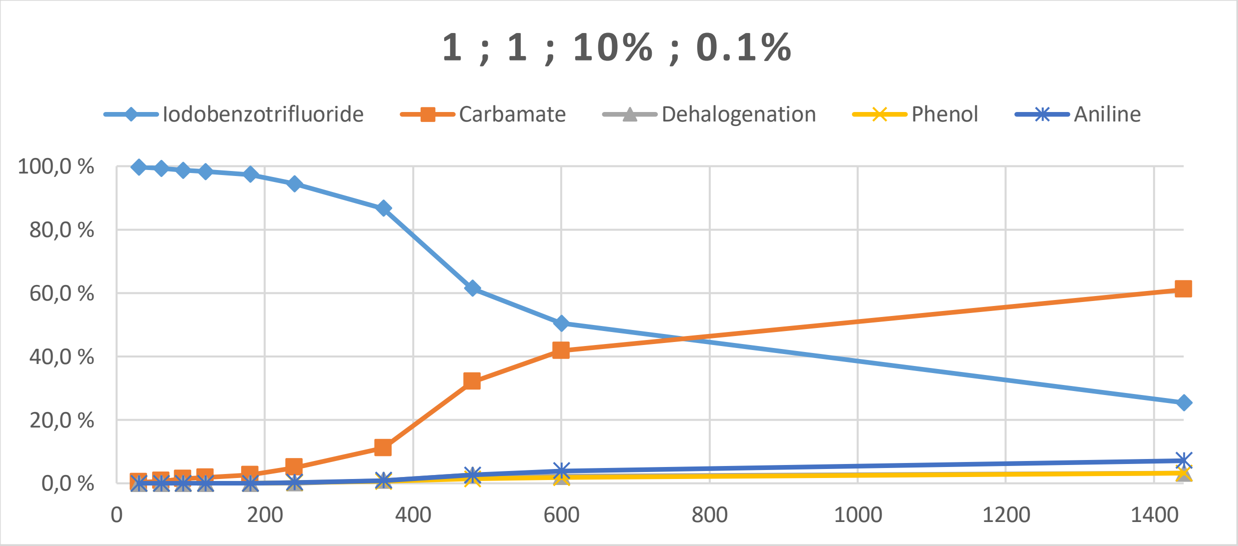

Figure S 43: Conversion of starting material and formation of different products over time (in minutes). The ratio of amine + TMG : p-iodobenzotrifluoride : $NiBr_2$dtbbpy : DPAPN-tBu is shown in the title.

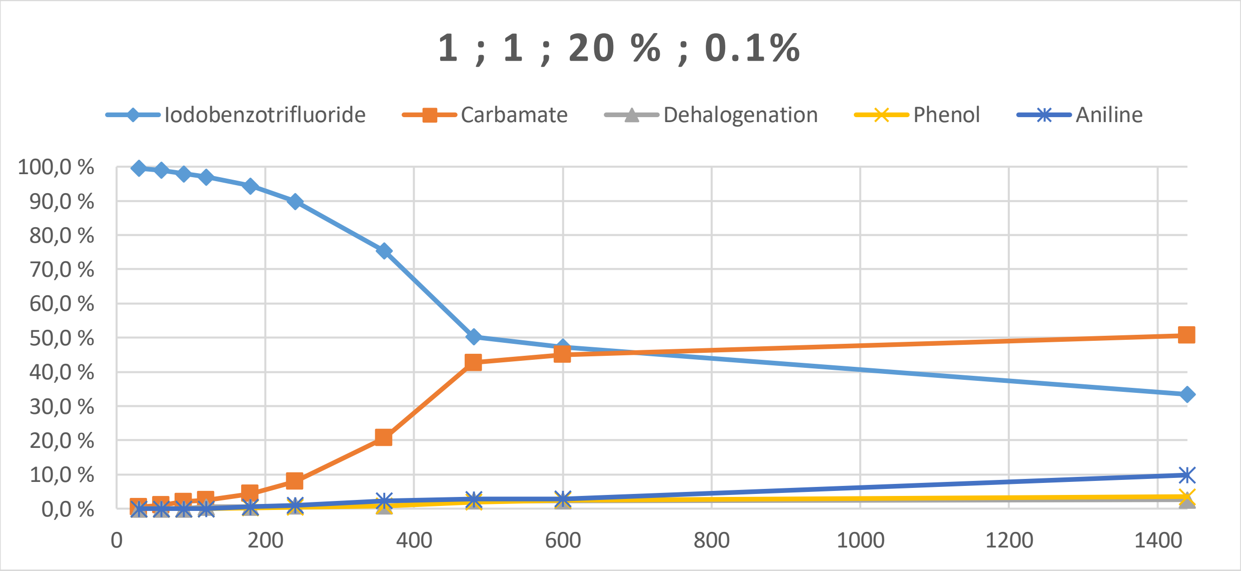

Figure S 44: Conversion of starting material and formation of different products over time (in minutes). The ratio of amine + TMG : p-iodobenzotrifluoride : $NiBr_2$dtbbpy : DPAPN-tBu is shown in the title.

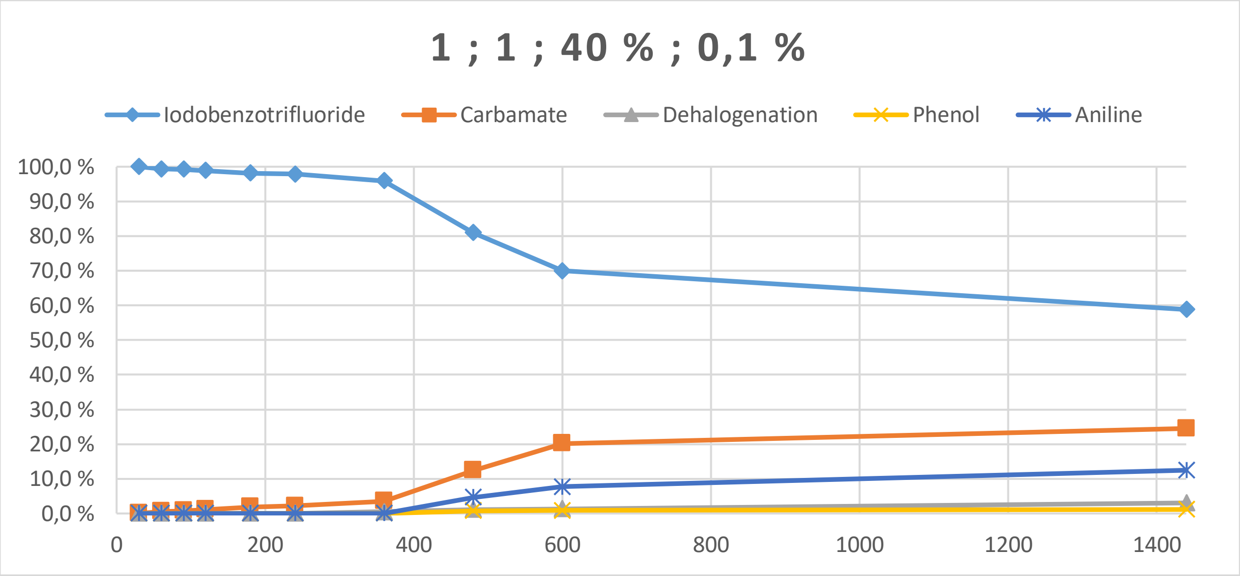

Figure S 45: Conversion of starting material and formation of different products over time (in minutes). The ratio of amine + TMG : p-iodobenzotrifluoride : $NiBr_2$dtbbpy : DPAPN-tBu is shown in the title.

14.3.4 DPAPN-tBu

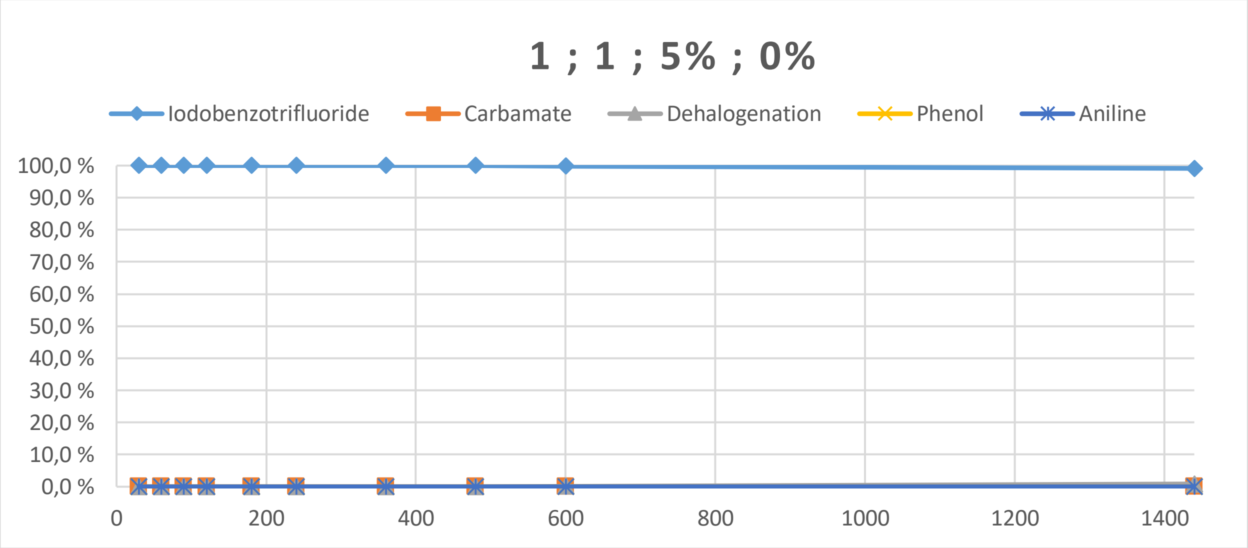

Figure S 46: Conversion of starting material and formation of different products over time (in minutes). The ratio of amine + TMG : p-iodobenzotrifluoride : $NiBr_2$dtbbpy : DPAPN-tBu is shown in the title.

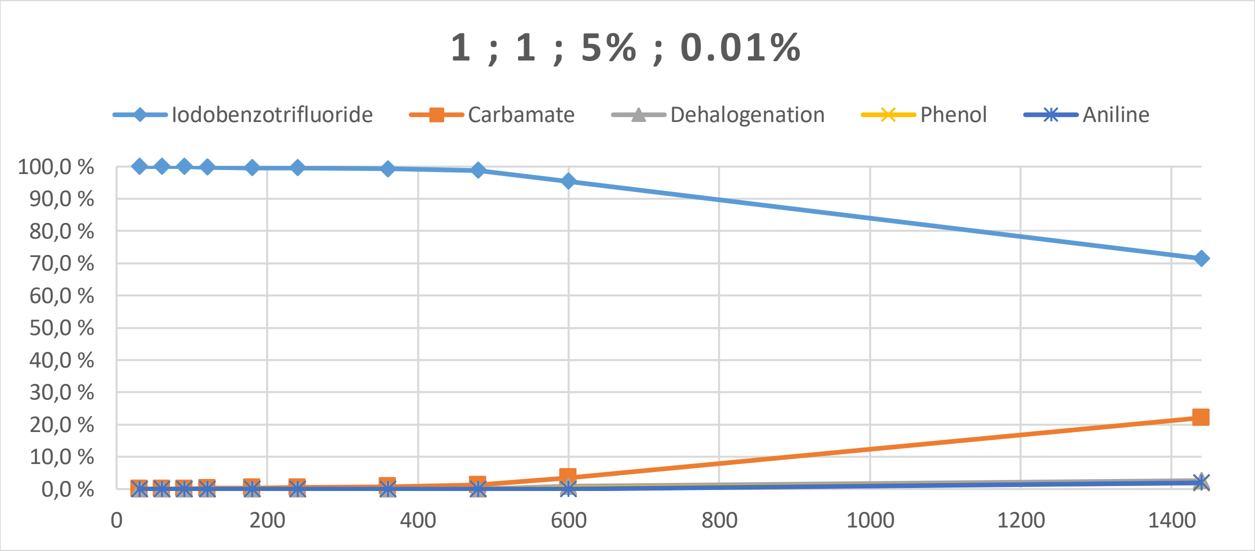

Figure S 47: Conversion of starting material and formation of different products over time (in minutes). The ratio of amine + TMG : p-iodobenzotrifluoride : $NiBr_2$dtbbpy : DPAPN-tBu is shown in the title.

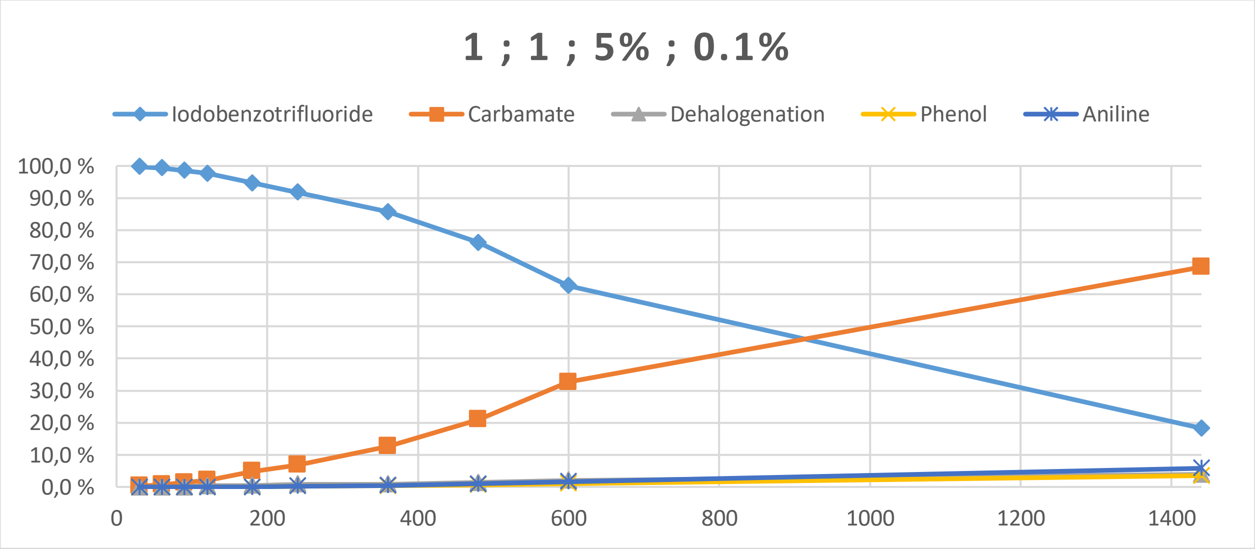

Figure S 48: Conversion of starting material and formation of different products over time (in minutes). The ratio of amine + TMG : p-iodobenzotrifluoride : $NiBr_2$dtbbpy : DPAPN-tBu is shown in the title.

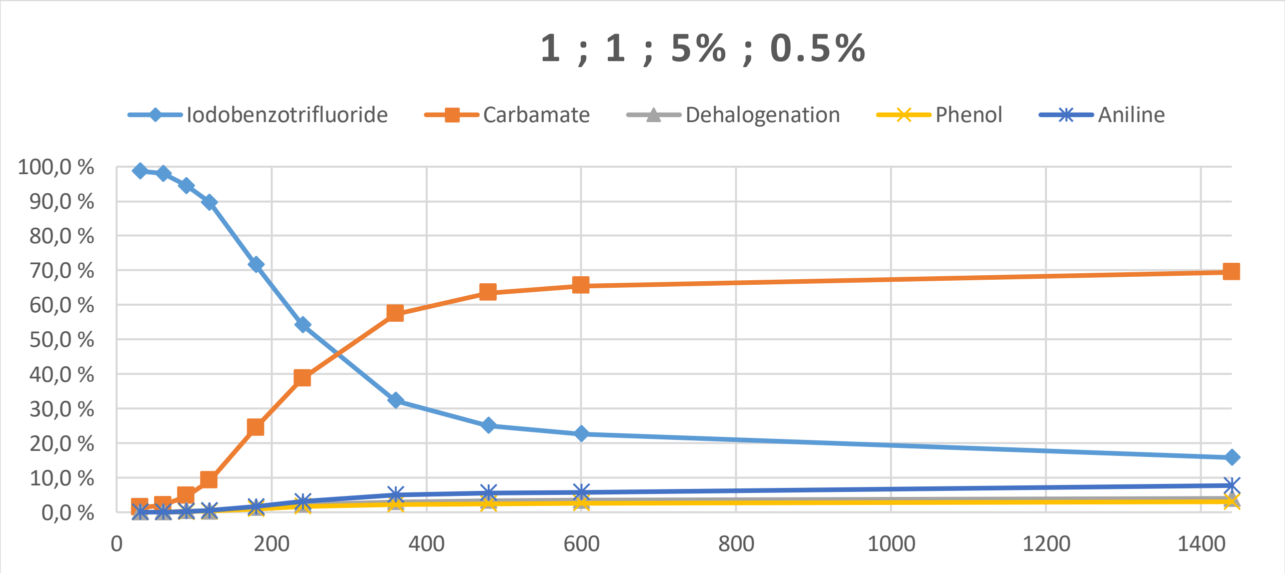

Figure S 49: Conversion of starting material and formation of different products over time (in minutes). The ratio of amine + TMG : p-iodobenzotrifluoride : $NiBr_2$dtbbpy : DPAPN-tBu is shown in the title.

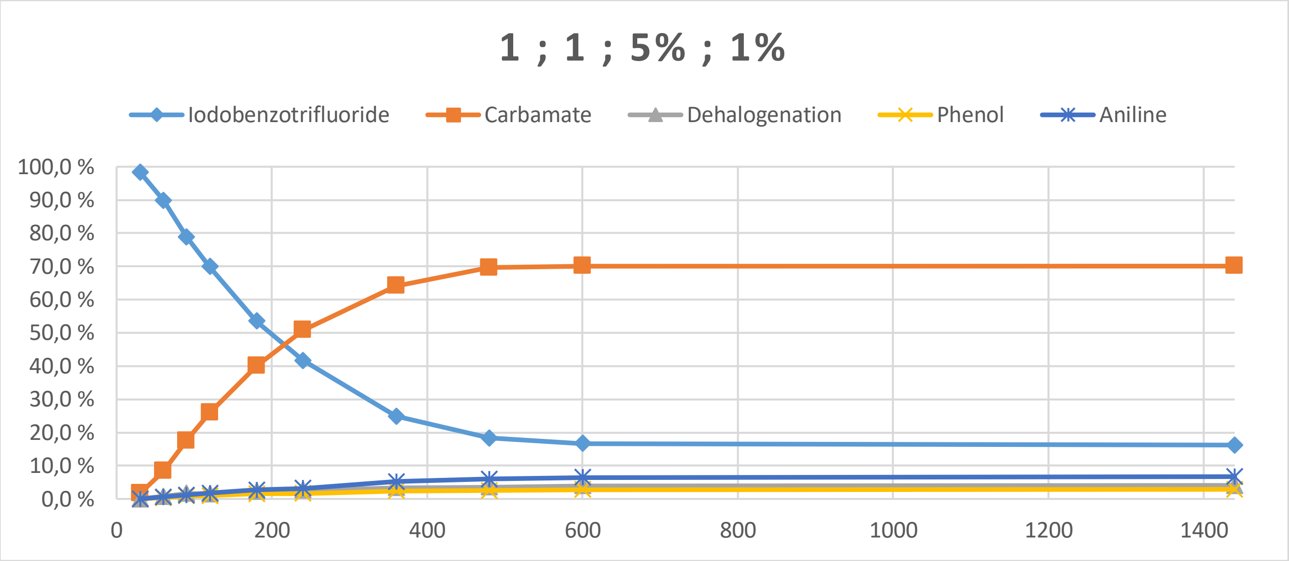

Figure S 50: Conversion of starting material and formation of different products over time (in minutes). The ratio of amine + TMG : p-iodobenzotrifluoride : $NiBr_2$dtbbpy : DPAPN-tBu is shown in the title.

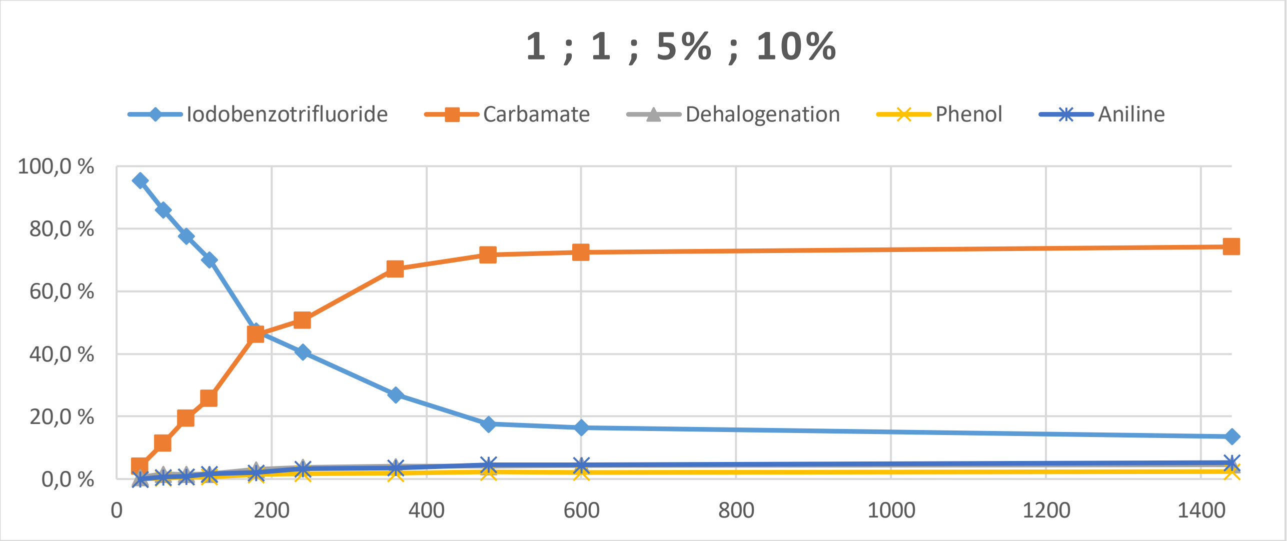

Figure S 51: Conversion of starting material and formation of different products over time (in minutes). The ratio of amine + TMG : p-iodobenzotrifluoride : $NiBr_2$dtbbpy : DPAPN-tBu is shown in the title.

14.3.5 $Ni(COD)_2$

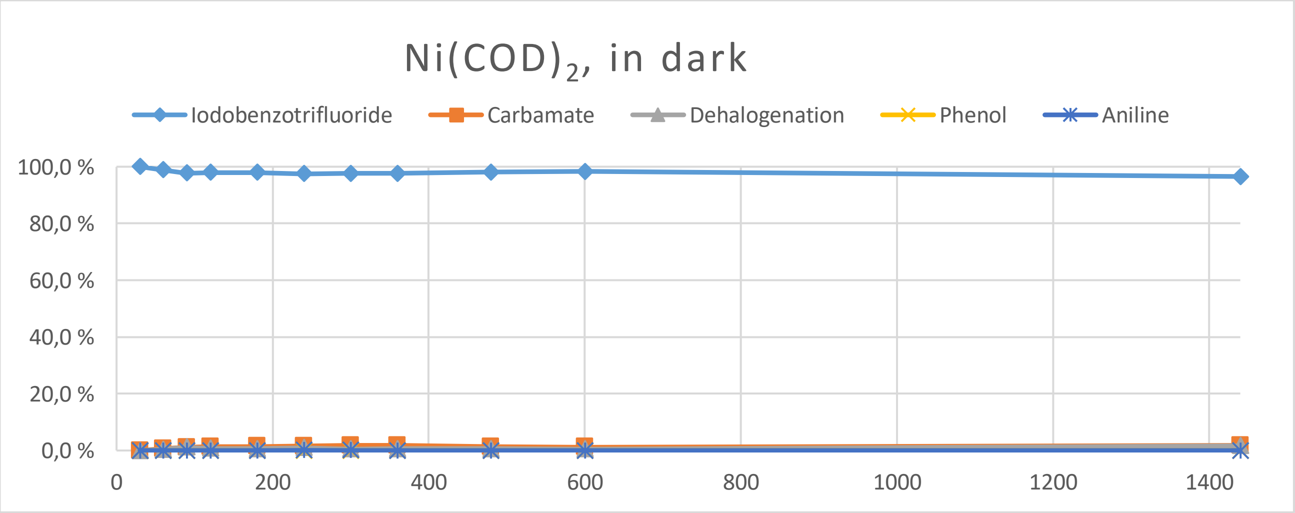

Figure S 52: Conversion of starting material and formation of different products over time (in minutes). Reaction stops after around 2 hours, with 2% conversion and 1.3% carbamate.

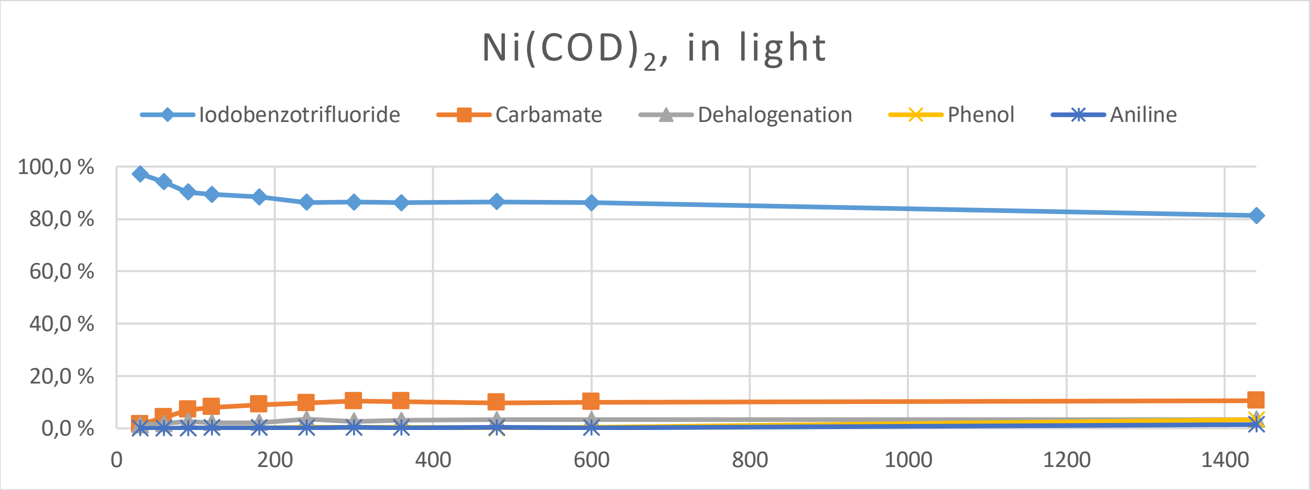

Figure S 53: Conversion of starting material and formation of different products over time (in minutes). Reaction stops after around 4 hours, with 14 % conversion and 10% carbamate

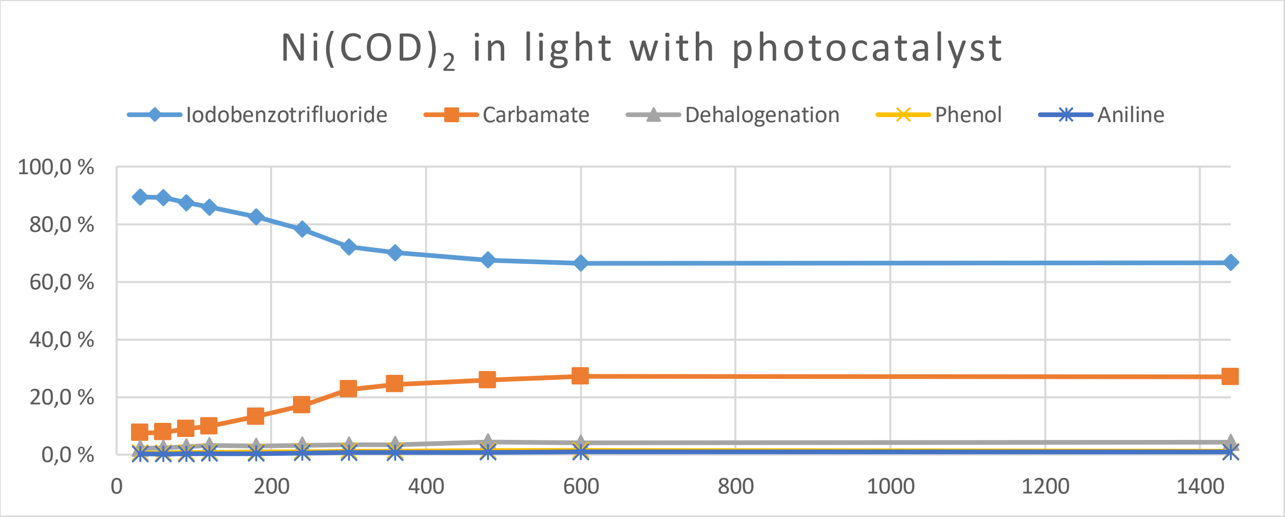

Figure S 54: Conversion of starting material and formation of different products over time (in minutes). First reaction cycle happens very quickly as the first measurement after 30 minutes already has 10% conversion with 7.5% carbamate. After 6 hours, the reaction slows significantly and finally stops after 10 hours, reaching final conversion of 33% and 27% carbamate.

14.3.6 Miscellaneous

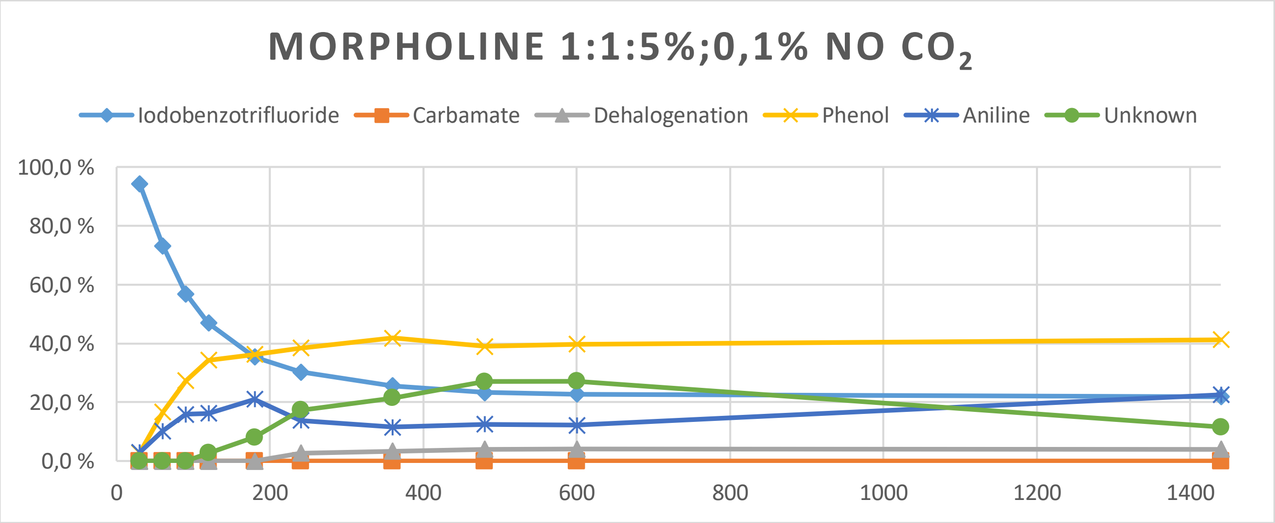

Figure S 55: Conversion of starting material and formation of different products over time (in minutes). The reaction was flushed with argon instead of CO_2. The unknown compound has a broad ^{19}F signal in DMF at -61.54, can't be detected

in GC/MS and can't be easily isolated. Large amount of phenol formation is explained by urea formation, which was confirmed with GC/MS.

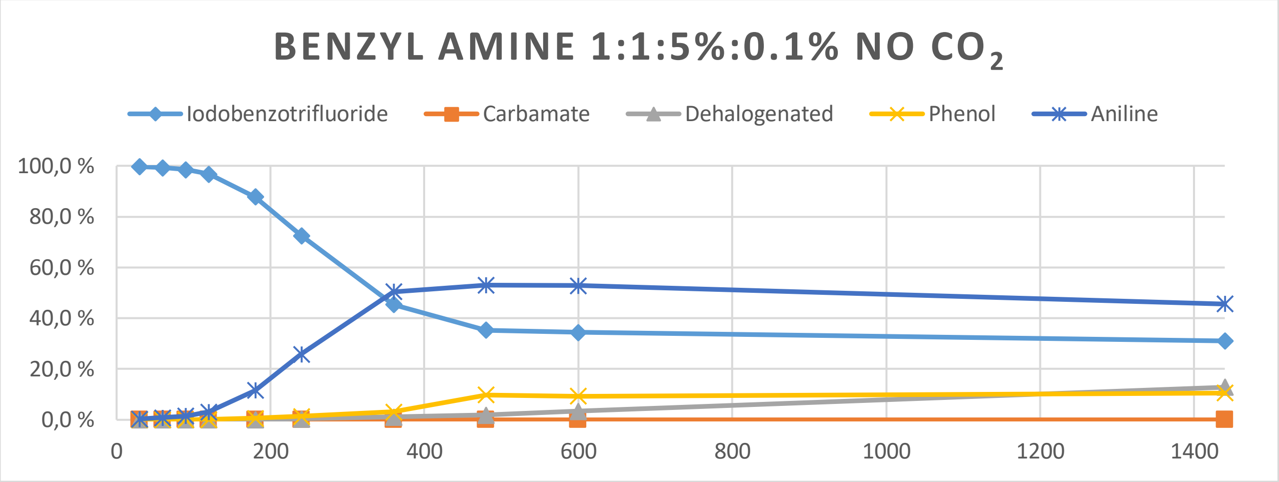

Figure S 56: Conversion of starting material and formation of different products over time (in minutes). The reaction was flushed with argon instead of CO_2.

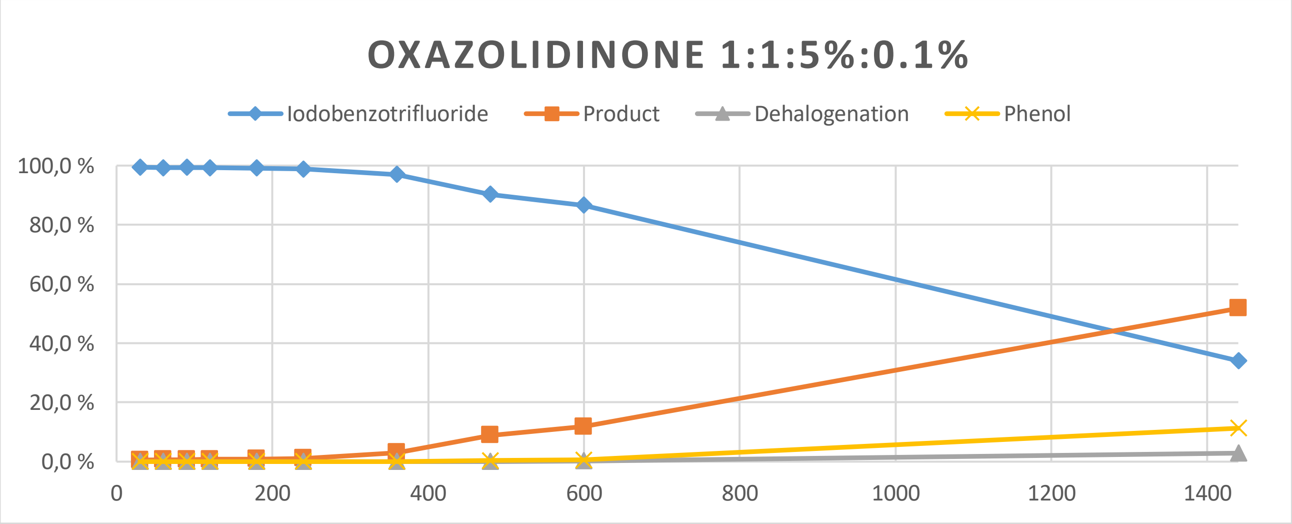

Figure S 57: Conversion of starting material and formation of different products over time (in minutes).

15 (DTBBPY)NI(O-TOLYL)BR

The potential intermediate (dtbbpy)Ni(o-tolyl)Br was tested for catalytic activity. (dtbbpy)Ni(o-tolyl)Br (10.0 mg, 0.02 mmol, 5%) was weighed to a dried 8 ml vial in argon filled glove box and sealed with a septum cap. 4-iodobenzotrifluoride (0.4 mmol, 1 eq), morpholine (0.8 mmol, 2 eq), tetramethylguanadine (0.8 mmol, 2 eq), DPAPN-tbu (0.4 µmol 0.1%) were added as degassed stock solutions in dimethylformamide. Total volume of the solution was 4 ml. The solution was irradiated with 2x32W CFL for 22 hours. 0.2 mmol of hexafluorobenzene was added as internal standard and 19F NMR was recorded.

Table S19: (dtbbpy)Ni(o-tolyl)Br catalytic activity

	CF_3PhI	CF_3Ph	Carbamate	Aniline	Phenol
As above	7,3 %	7,0 %	77,7 %	3,9 %	4,1 %
No DPAPN-tbu	84,0 %	2,1 %	12,4 %	1,0 %	0,5 %
Dark	100,0 %	0,0 %	0,0 %	0,0 %	0,0 %

The result are very close to the optimized conditions with (dtbbpy)$NiBr_2$, except full conversion wasn't quite reached.

(dtbbpy)Ni(o-tolyl)Br was also studied stoichimetrically. In argon filled glovebox, 2.7 mg (5.4 µmol) of (dtbbpy)Ni(o-tolyl)Br and 4.0 mg (16.3 µmol) of tetramethylguanadinium morpholine carboxylate was added to a 2 ml vial followed by 1.5 ml of dry degassed acetonitrile. Either 1.5 mg (5.4 µmol) of ferrocinium tetrafluoroborate or 0.1 mg of DPAPN-tbu was added. The vials were sealed and then stirred in with or without irradiation in 40-45 °C for 3 hours. 5.4 µmol of mesitylene was added as internal standard. The reaction mixture was poured in 5 ml of 3 M HCl, 2 ml of EtOAc was added and the mixture was vigorously stirred. NaCl was added to saturate the aquous phase. The organic layer was taken as a gas chromatography sample to determinate the yield.

Table S 20: Stoichiometric reaction with (dtbbpy)Ni(o-tolyl)Br and 3 equivalents of tetramethyl guanidium morpholine-N-carboxylate. Carbamate was only formed, when ferrocenium tetrafluoroborate was added.

	Toluene (%)	Carbamate (%)
Dark	67.2	0
With light	63.5	0
With photocatalyst	54.0	0
With $FeCp_2BF_4$	30.6	30.5

No carbamate formation was detected when only 1 equivalent of tetramethylguanadinium morpholine carboxylate was used. With irradiation only toluene was formed. In dark or with ferrocenium tetrafluoroborate, NMR shows that the o-tolyl is still connected to the nickel.

The stoichiometric reaction was also studied by NMR, but the signals were too broad in CD_3CN, CD_2Cl_2 and C_6D_6 for accurate measurements.

16 REFERENCES

1. Elgrishi, N.; Rountree, K. J.; McCarthy, B. D.; Rountree, E. S.; Eisenhart, T. T.; Dempsey, J. L., A Practical Beginner's Guide to Cyclic Voltammetry. *Journal of chemical education* **2017,** *95* (2), 197-206.
2. Pavlishchuk, V. V.; Addison, A. W., Conversion constants for redox potentials measured versus different reference electrodes in acetonitrile solutions at 25°C. *Inorganica Chimica Acta* **2000,** *298* (1), 97-102.
3. Kortunov, P. V.; Baugh, L. S.; Siskin, M.; Calabro, D. C., In Situ Nuclear Magnetic Resonance Mechanistic Studies of Carbon Dioxide Reactions with Liquid Amines in Mixed Base Systems: The Interplay of Lewis and Brønsted Basicities. *Energy Fuels* **2015,** *29* (9), 5967-5989.
4. Mannisto, J. K.; Pavlovic, L.; Tiainen, T.; Nieger, M.; Sahari, A.; Hopmann, K. H.; Repo, T., Mechanistic insights into carbamate formation from CO2 and amines: the role of guanidine–CO2 adducts. *Catalysis Science & Technology* **2021**.
5. Escobar, R. A.; Johannes, J. W., A Unified and Practical Method for Carbon–Heteroatom Cross-Coupling using Nickel/Photo Dual Catalysis. *Chem. Eur. J.* **2020,** *26* (23), 5168-5173.
6. Welin, E. R.; Le, C.; Arias-Rotondo, D. M.; McCusker, J. K.; MacMillan, D. W. C., Photosensitized, energy transfer-mediated organometallic catalysis through electronically excited nickel(II). *Science* **2017,** *355* (6323), 380-385.
7. Lu, J.; Pattengale, B.; Liu, Q.; Yang, S.; Shi, W.; Li, S.; Huang, J.; Zhang, J., Donor–Acceptor Fluorophores for Energy-Transfer-Mediated Photocatalysis. *J. Am. Chem. Soc.* **2018,** *140* (42), 13719-13725.
8. Ou, W.; Zou, R.; Han, M.; Yu, L.; Su, C., Tailorable carbazolyl cyanobenzene-based photocatalysts for visible light-induced reduction of aryl halides. *Chin. Chem. Lett.* **2020,** *31* (7), 1899-1902.
9. Corcoran, E. B.; Pirnot, M. T.; Lin, S.; Dreher, S. D.; DiRocco, D. A.; Davies, I. W.; Buchwald, S. L.; MacMillan, D. W. C., Aryl amination using ligand-free Ni(II) salts and photoredox catalysis. *Science* **2016,** *353* (6296), 279-283.
10. Liu, Y.; Chen, X.-L.; Li, X.-Y.; Zhu, S.-S.; Li, S.-J.; Song, Y.; Qu, L.-B.; Yu, B., 4CzIPN-tBu-Catalyzed Proton-Coupled Electron Transfer for Photosynthesis of Phosphorylated N-Heteroaromatics. *J. Am. Chem. Soc.* **2020,** *143* (2).
11. Xiong, W.; Qi, C.; Guo, T.; Zhang, M.; Chen, K.; Jiang, H., A copper-catalyzed oxidative coupling reaction of arylboronic acids, amines and carbon dioxide using molecular oxygen as the oxidant. *Green Chem.* **2017,** *19* (7), 1642-1646.
12. Sun, R.; Qin, Y.; Nocera, D. G., General Paradigm in Photoredox Nickel-Catalyzed Cross-Coupling Allows for Light-Free Access to Reactivity. *Angew. Chem. Int. Ed.* **2020,** *59* (24), 9527-9533.
13. Pearson, R. M.; Lim, C.-H.; McCarthy, B. G.; Musgrave, C. B.; Miyake, G. M., Organocatalyzed Atom Transfer Radical Polymerization Using N-Aryl Phenoxazines as Photoredox Catalysts. *J. Am. Chem. Soc.* **2016,** *138* (35), 11399-11407.
14. Mannisto, J. K.; Sahari, A.; Lagerblom, K.; Niemi, T.; Nieger, M.; Sztano, G.; Repo, T., One-Step Synthesis of 3,4-Disubstituted 2-Oxazolidinones by Base-Catalyzed CO(2) Fixation and Aza-Michael Addition. *Chemistry* **2019,** *25* (44), 10284-10289.
15. Bragg, R. A.; Clayden, J.; Morris, G. A.; Pink, J. H., Stereodynamics of Bond Rotation in Tertiary Aromatic Amides. *Chemistry - A European Journal* **2002,** *8* (6), 1279-1289.
16. Mandel, J.; Pan, X.; Hay, E. B.; Geib, S. J.; Wilcox, C. S.; Curran, D. P., Rotational isomers of N-methyl-N-arylacetamides and their derived enolates: implications for asymmetric Hartwig oxindole cyclizations. *J. Org. Chem.* **2013,** *78* (8), 4083-9.
17. Nishizawa, A.; Takahira, T.; Yasui, K.; Fujimoto, H.; Iwai, T.; Sawamura, M.; Chatani, N.; Tobisu, M., Nickel-Catalyzed Decarboxylation of Aryl Carbamates for Converting Phenols into Aromatic Amines. *J. Am. Chem. Soc.* **2019,** *141* (18), 7261-7265.
18. Watson, R. B.; Butler, T. W.; DeForest, J. C., Preparation of Carbamates, Esters, Amides, and Unsymmetrical Ureas via Brønsted Acid-Activated N-Acyl Imidazoliums. *Org. Process Res. Dev.* **2021,** *25* (3), 500-506.
19. Nakamura, K.; Yasui, K.; Tobisu, M.; Chatani, N., Rhodium-catalyzed cross-coupling of aryl carbamates with arylboron reagents. *Tetrahedron; Tetrahedron Young Investigator Award 2015 Cooperative Catalysis for Inert Bond Functionalization Yoshiaki Nakao* **2015,** *71* (26), 4484-4489.
20. Varjosaari, S. E.; Suating, P.; Adler, M. J., One-Pot Synthesis of O-Aryl Carbamates. *Synthesis* **2016,** *48* (1), 43-47.
21. Grzyb, J. A.; Shen, M.; Yoshina-Ishii, C.; Chi, W.; Brown, R. S.; Batey, R. A., Carbamoylimidazolium and thiocarbamoylimidazolium salts: novel reagents for the synthesis of ureas, thioureas, carbamates, thiocarbamates and amides. *Tetrahedron* **2005,** *61* (30), 7153-7175.
22. Nishizawa, A.; Takahira, T.; Yasui, K.; Fujimoto, H.; Iwai, T.; Sawamura, M.; Chatani, N.; Tobisu, M., Nickel-Catalyzed Decarboxylation of Aryl Carbamates for Converting Phenols into Aromatic Amines. *J. Am. Chem. Soc.* **2019,** *141* (18), 7261-7265.

23. Kleigrewe, N.; Steffen, W.; Blomker, T.; Kehr, G.; Frohlich, R.; Wibbeling, B.; Erker, G.; Wasilke, J. C.; Wu, G.; Bazan, G. C., Chelate bis(imino)pyridine cobalt complexes: synthesis, reduction, and evidence for the generation of ethene polymerization catalysts by Li+ cation activation. *J. Am. Chem. Soc.* **2005,** *127* (40), 13955-68.
24. Ladouceur, S.; Swanick, K. N.; Gallagher-Duval, S.; Ding, Z.; Zysman-Colman, E., Strongly Blue Luminescent Cationic Iridium(III) Complexes with an Electron-Rich Ancillary Ligand: Evaluation of Their Optoelectronic and Electrochemiluminescence Properties. *Eur. J. Inorg. Chem.* **2013,** *2013* (30), 5329-5343.
25. De Bon, F.; Abreu, C. M. R.; Serra, A. C.; Gennaro, A.; Coelho, J. F. J.; Isse, A. A., Catalytic Halogen Exchange in Supplementary Activator and Reducing Agent Atom Transfer Radical Polymerization for the Synthesis of Block Copolymers. *Macromol. Rapid Commun.* **2021,** *42* (4), 2000532-2000532.
26. Watson, R. B.; Butler, T. W.; DeForest, J. C., Preparation of Carbamates, Esters, Amides, and Unsymmetrical Ureas via Brønsted Acid-Activated N-Acyl Imidazoliums. *Org. Process Res. Dev.* **2020,** *25* (3), 500-506.
27. Nakamura, Y.; Maruya, K.-i.; Mizoroki, T., A Study of the Ligand Exchange of Bromo(o-tolyl)bis(triphenylphosphine)nickel(II) with Amine by Means of31P-and13C-NMR Spectroscopy. *Bull. Chem. Soc. Jpn.* **1980,** *53* (11), 3089-3092.
28. McQuarrie, D. A.; Simon, J. D., *Physical Chemistry: A Molecular Approach*. 1 ed.; University Science Books: California, 1997; p 592-595.

17 NMR AND IR SPECTRA

17.1 NMR AND IR SPECTRA OF PHOTOCATALYSTS

^{1}H and ^{13}C NMR and IR spectra of 4DPAPN (7a)

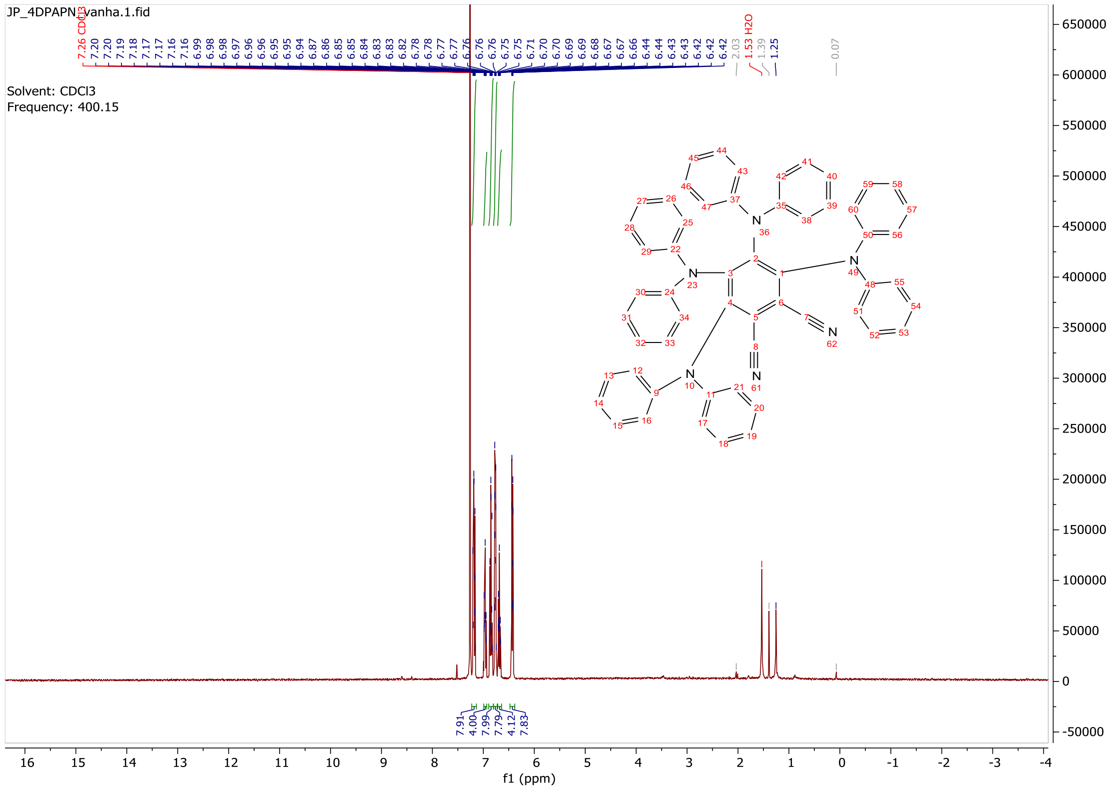

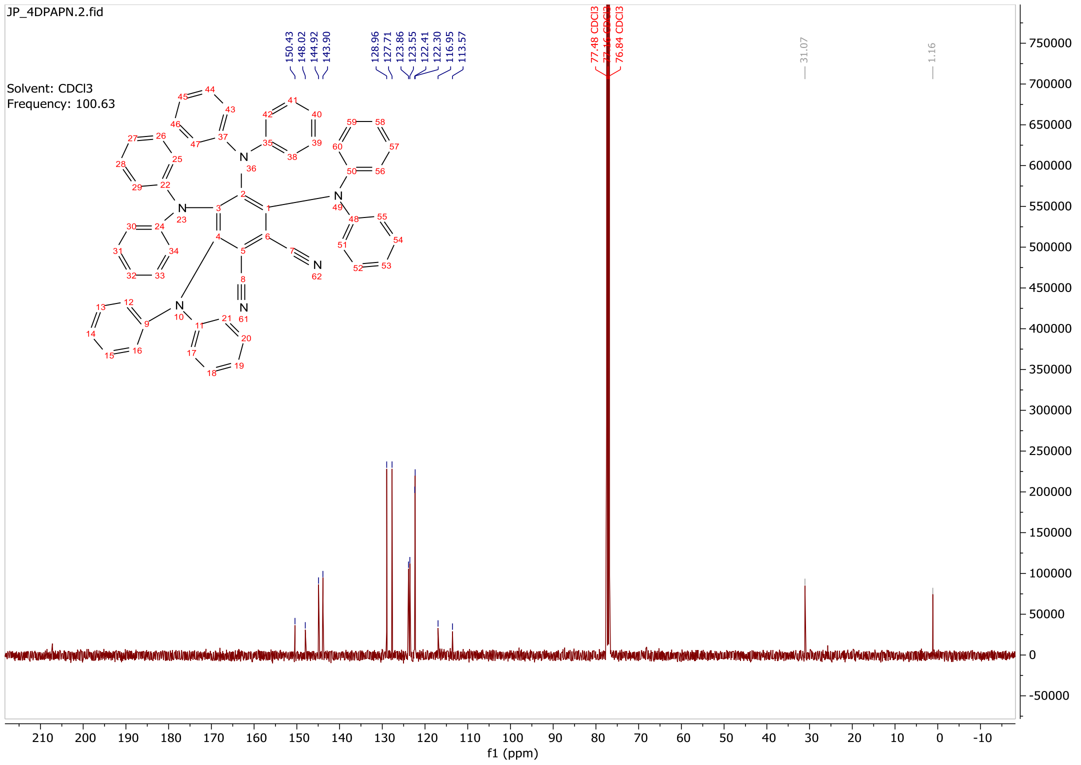

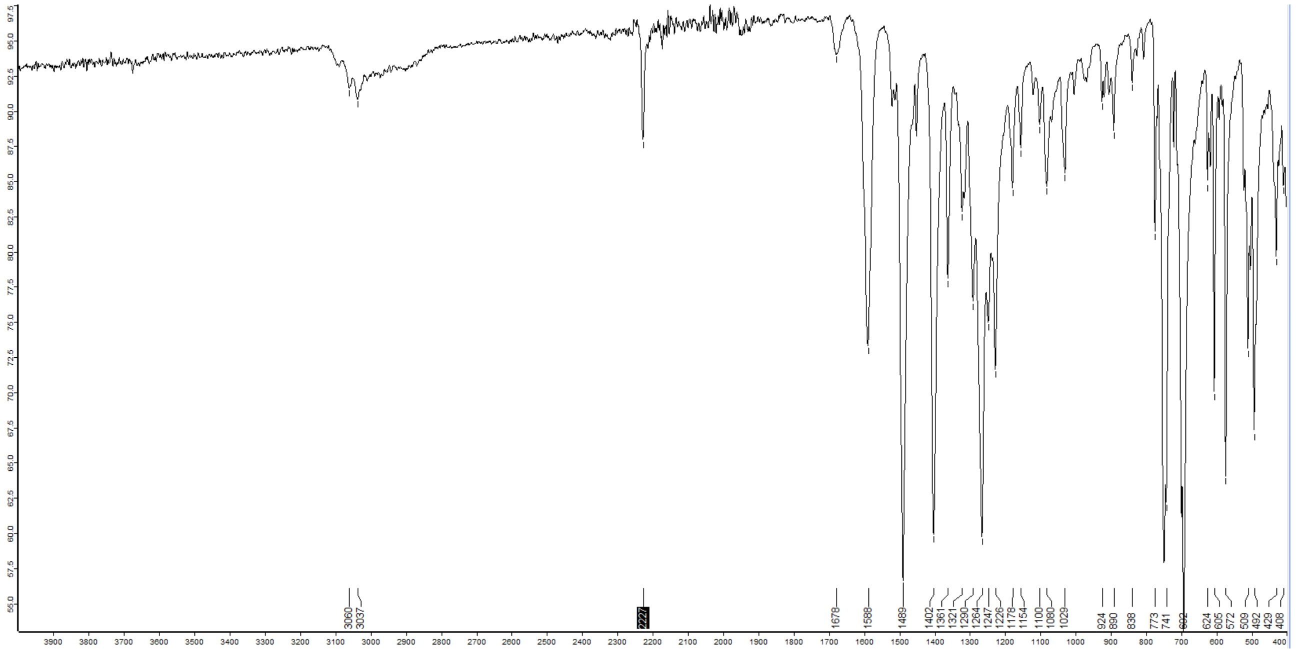

^{1}H and ^{13}C NMR and IR spectra of 4DPAIPN (8a)

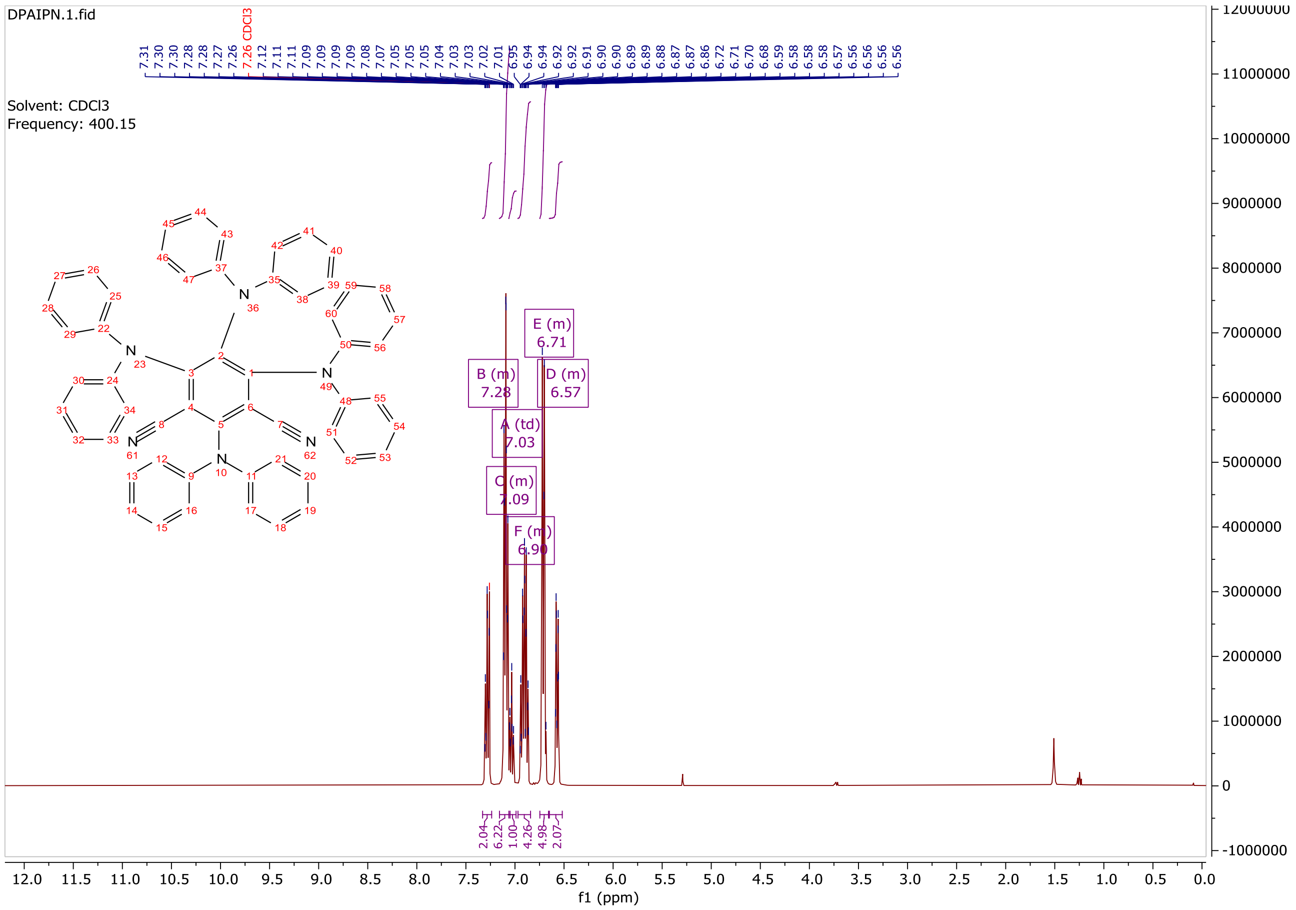

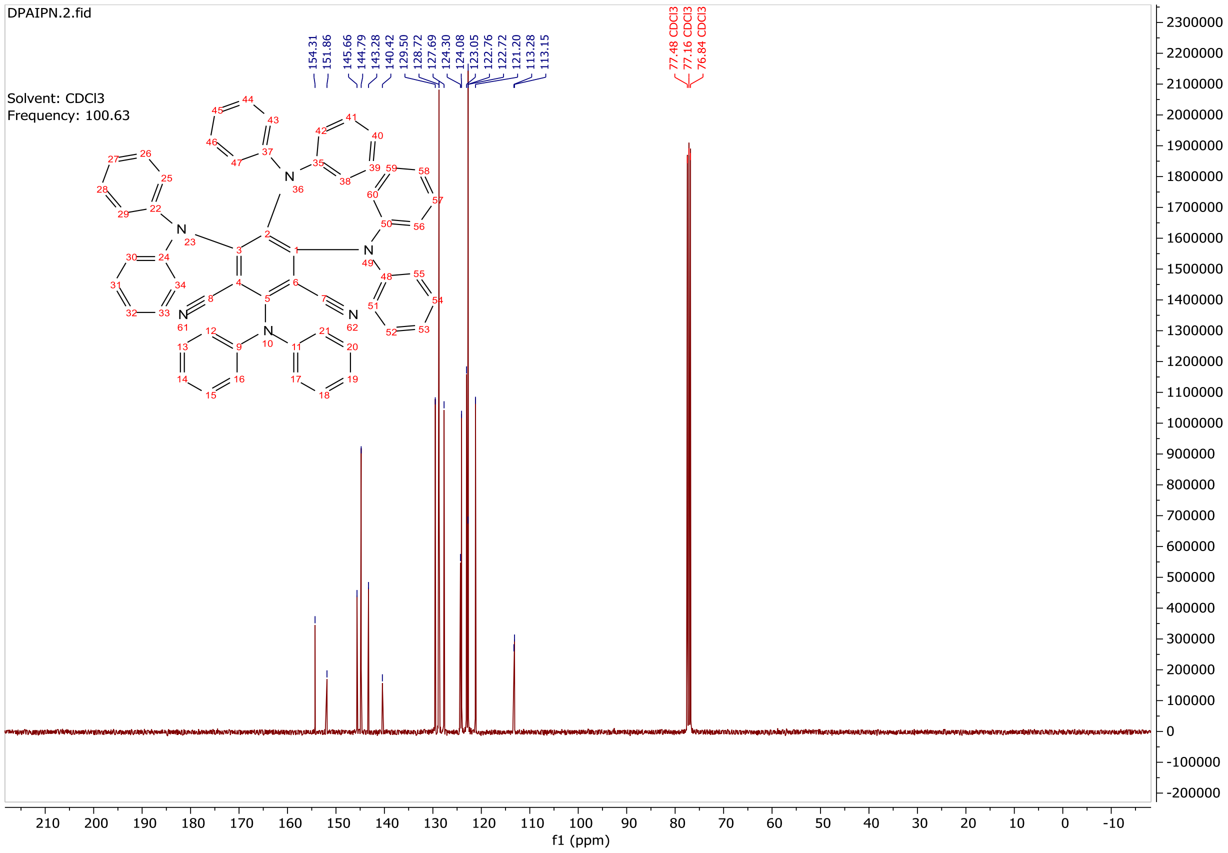
DPAIPN.2.fid
Solvent: CDCl3
Frequency: 100.63
154.31
151.86
145.66
144.79
143.28
140.42
129.50
128.72
127.69
124.30
124.08
123.05
122.76
122.72
121.20
113.28
113.15
77.48 CDCl3
77.16 CDCl3
76.84 CDCl3
f1 (ppm)

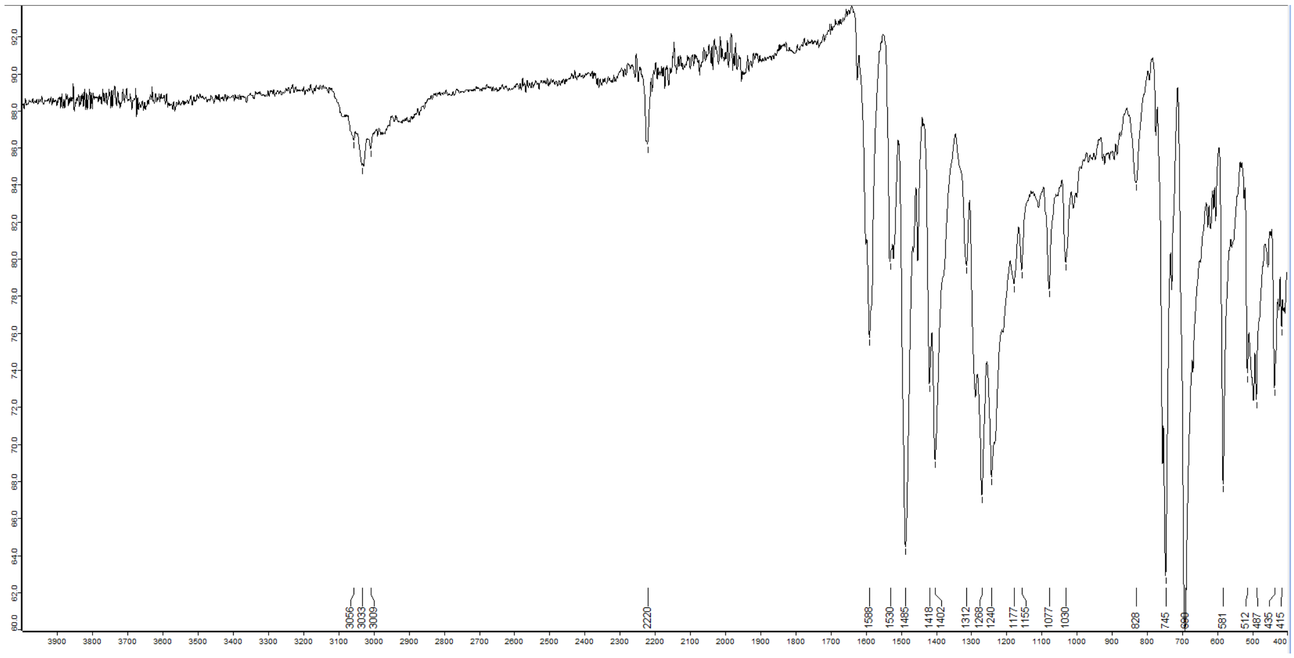
3056
3033
3009
2220
1588
1530
1485
1418
1402
1312
1268
1240
1177
1155
1077
1030
828
745
581
512
487
435
415

^{1}H and ^{13}C NMR and IR spectra of 4DPATPN (9a)

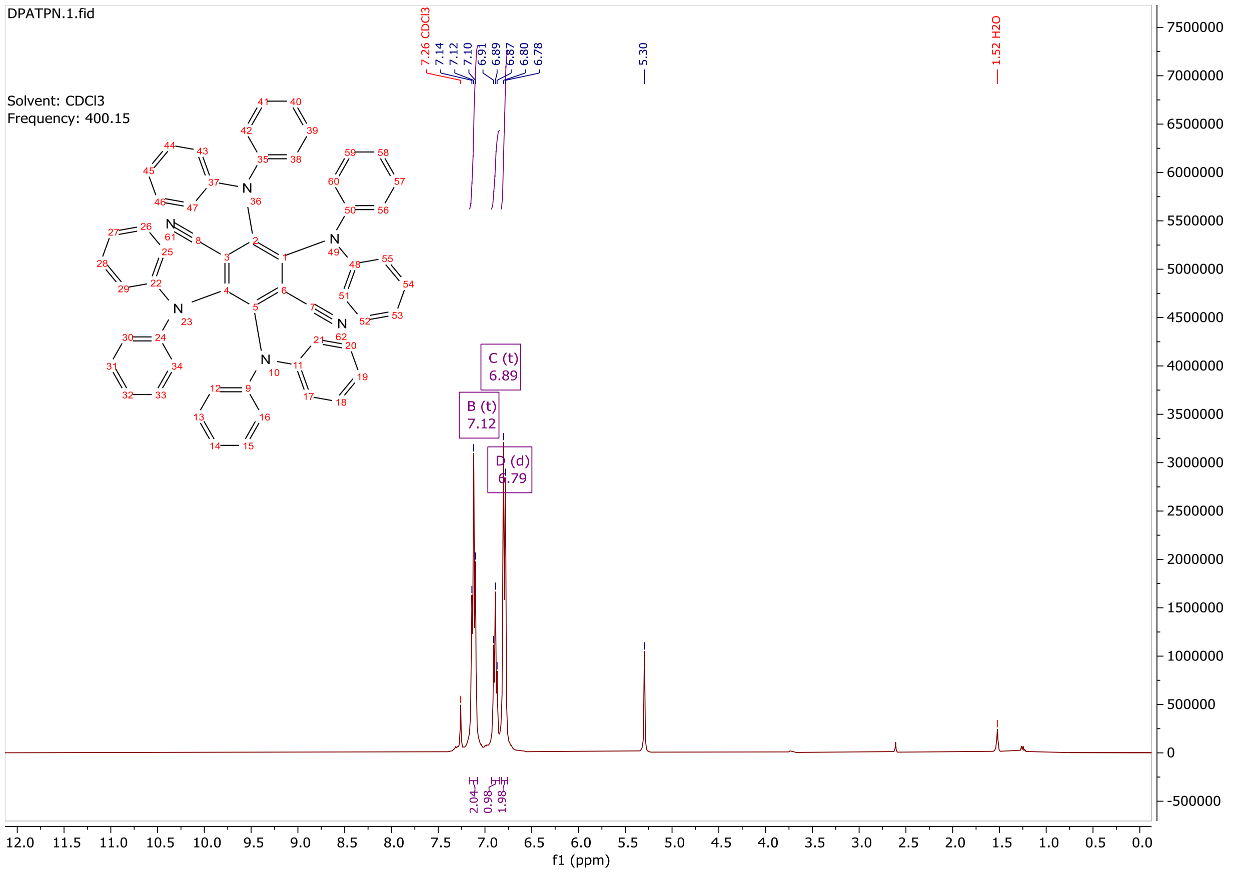

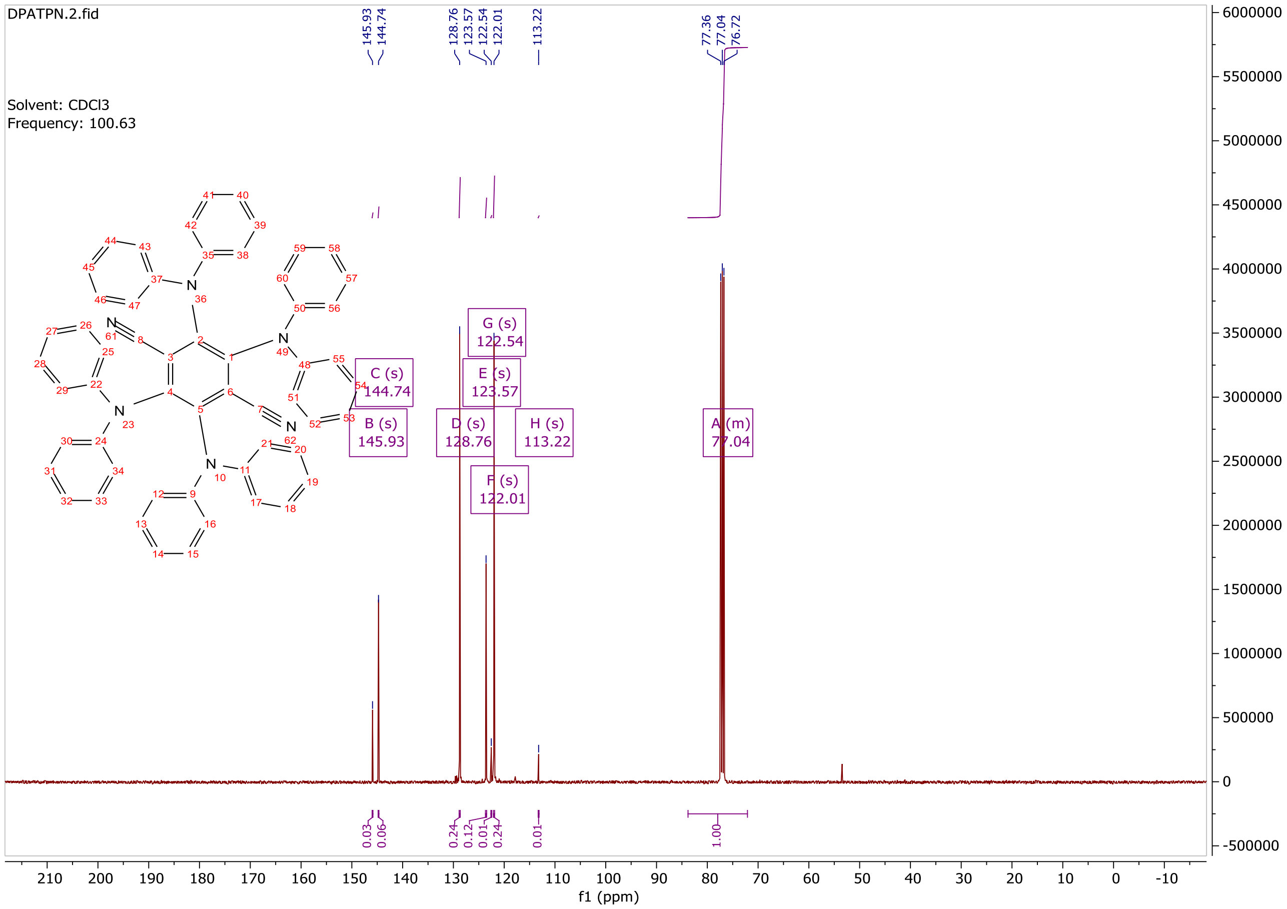

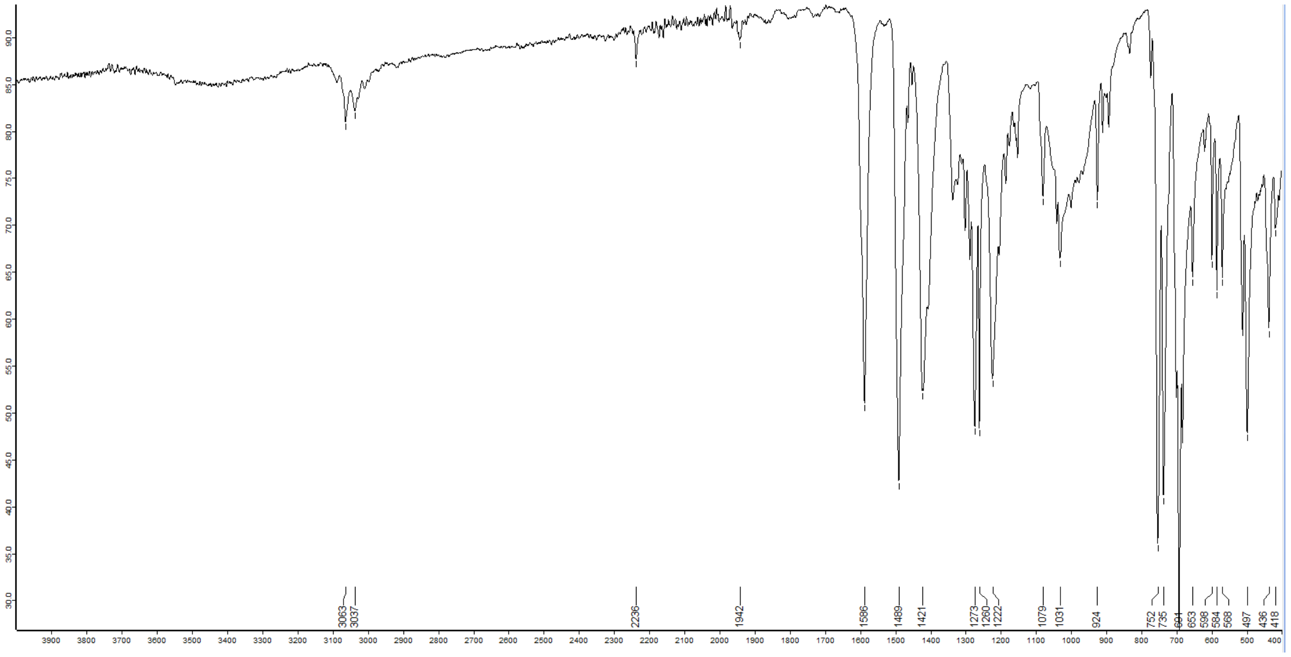
3063
3037
2236
1942
1586
1489
1421
1273
1260
1222
1079
1031
924
752
735
691
653
598
584
568
497
436
418

^{1}H and ^{13}C NMR and IR spectra of 4DPAPN-OMe (7b)

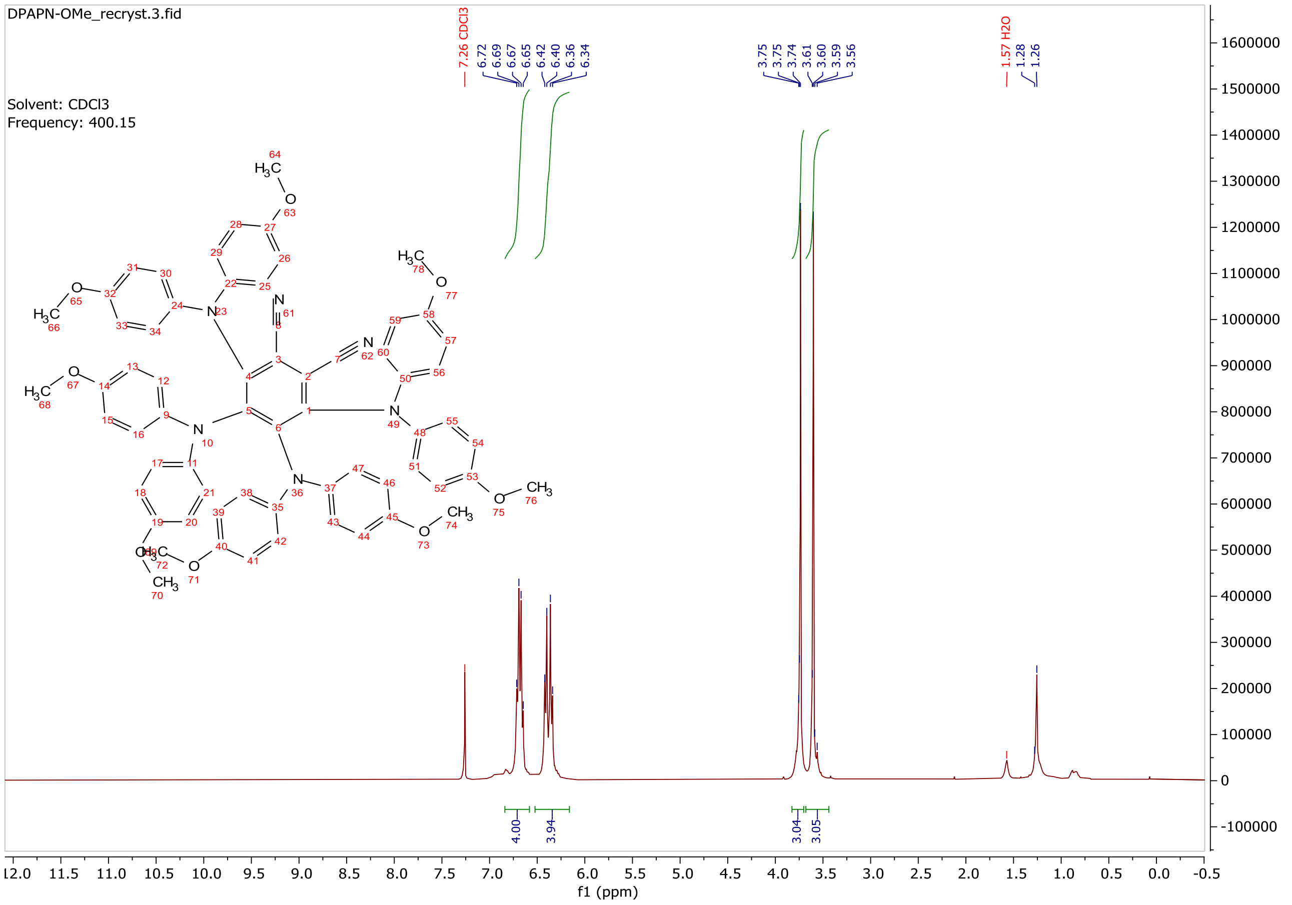

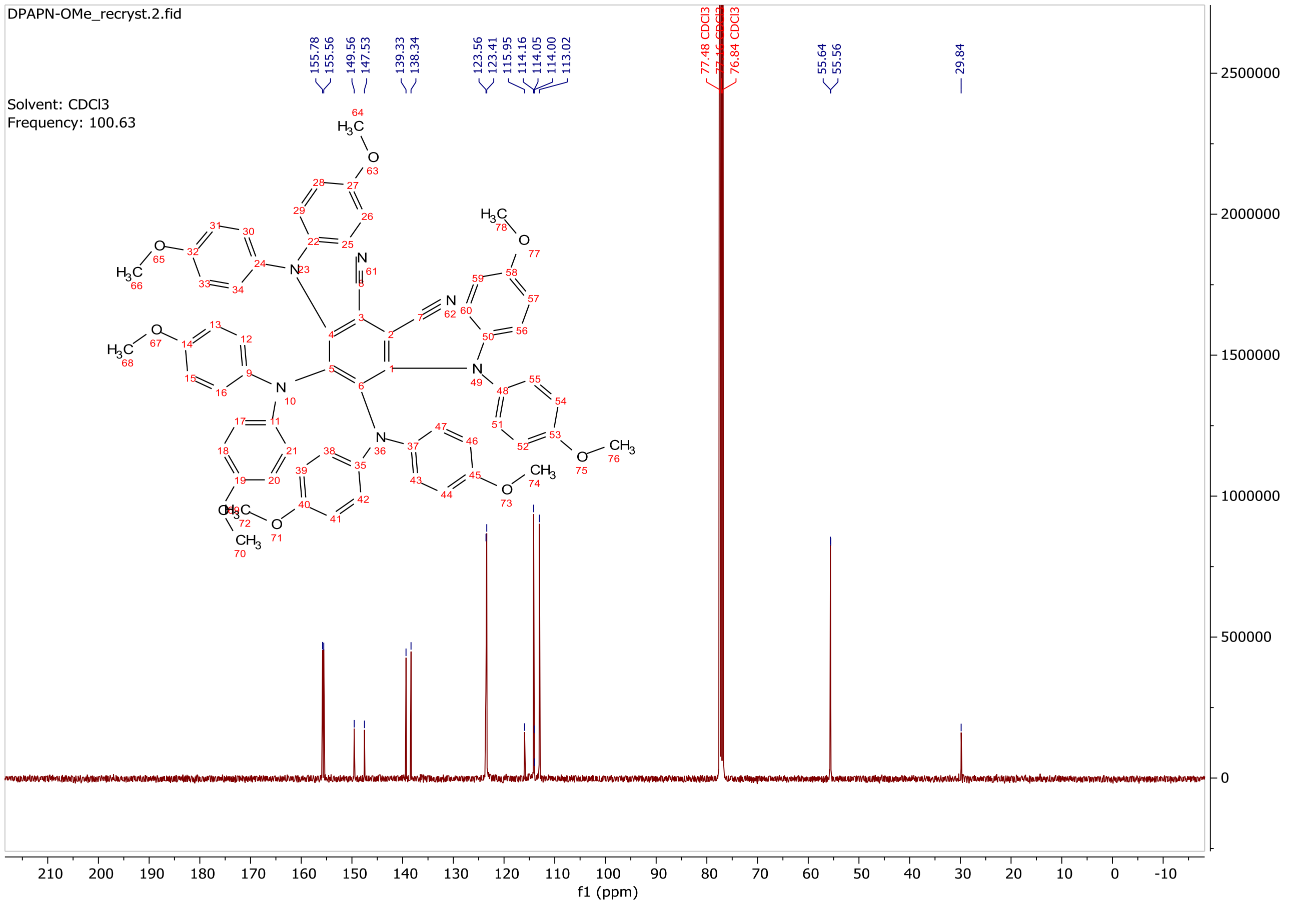

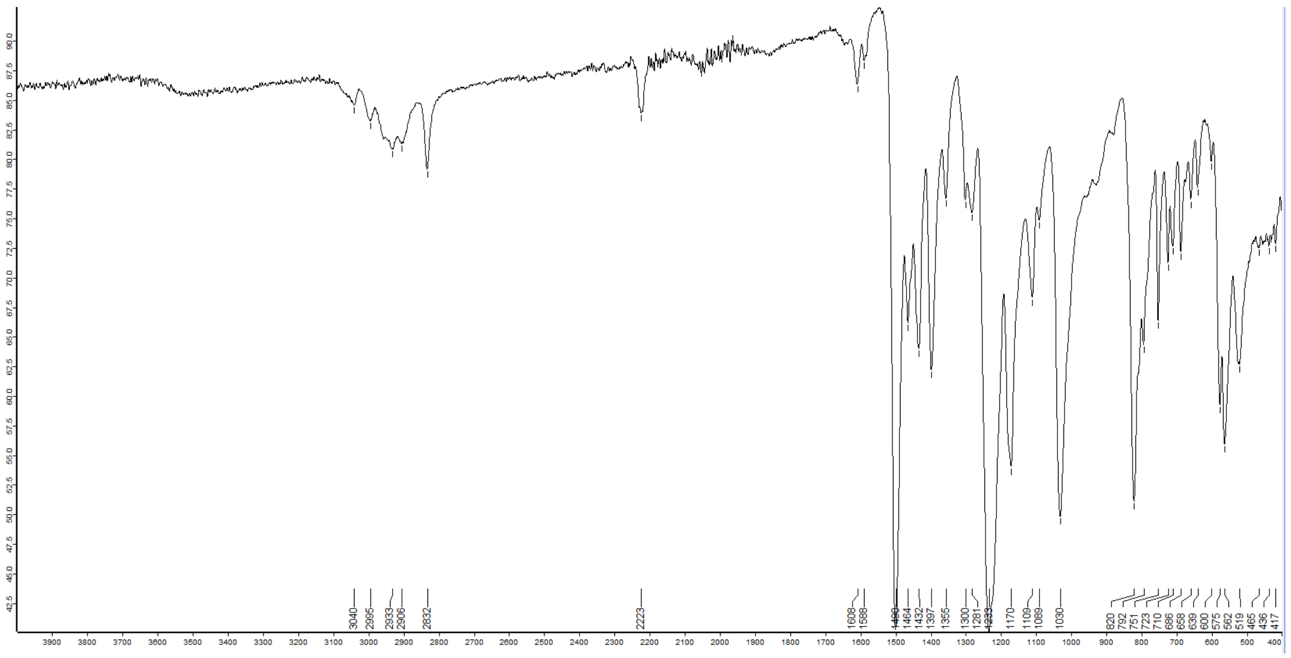

^{1}H and ^{13}C NMR and IR spectra of 4DPAIPN-OMe (8b)

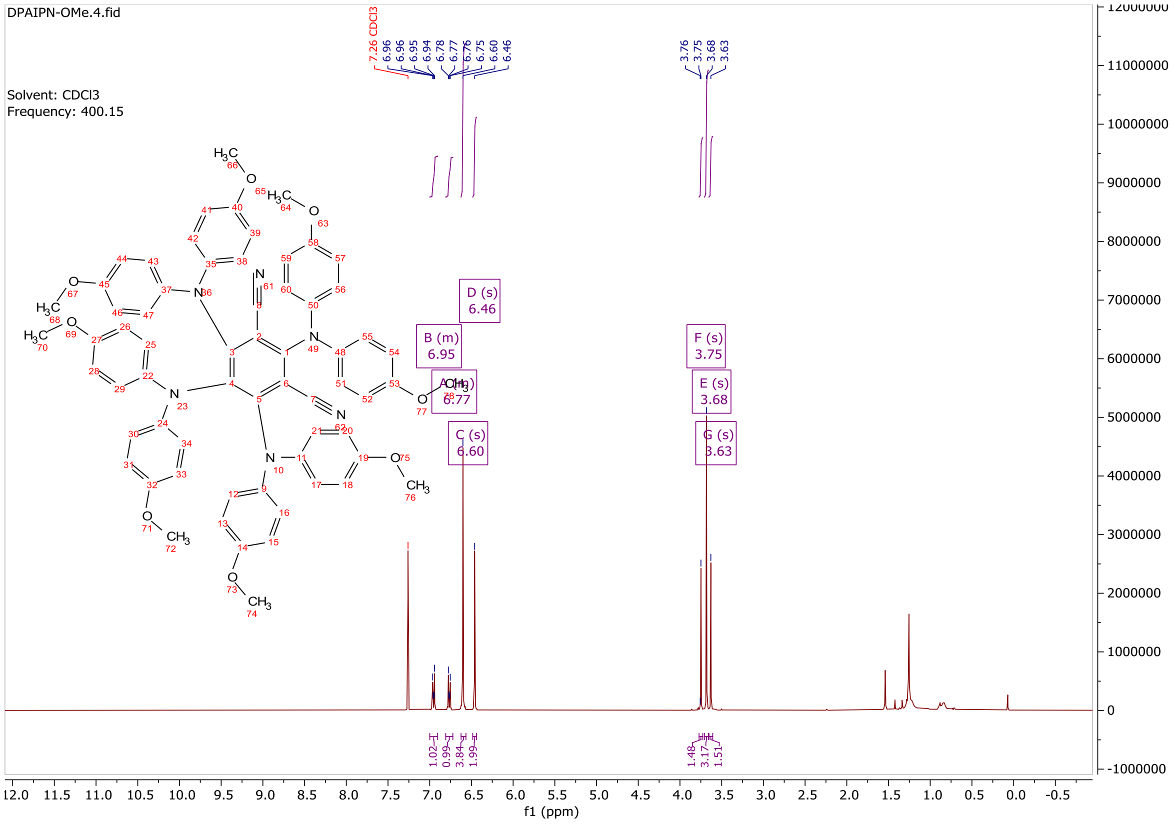

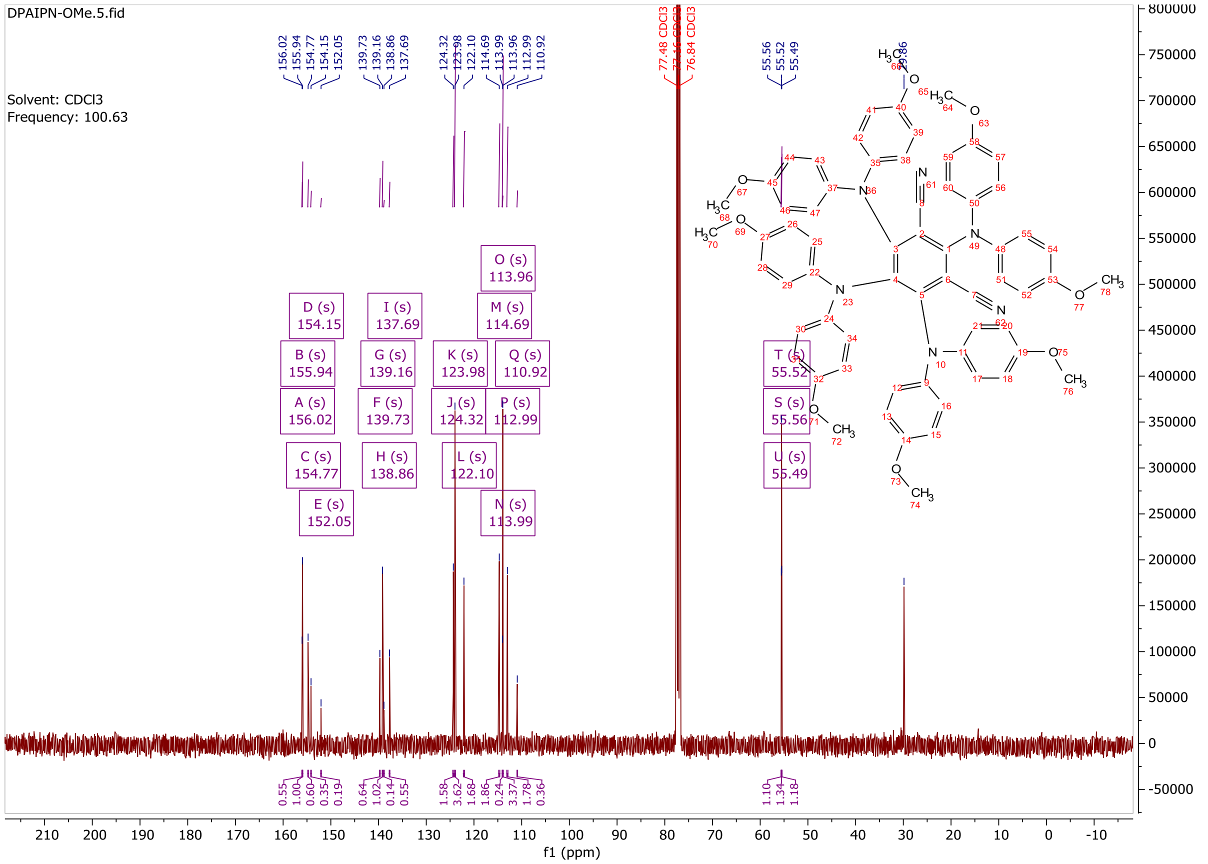
DPAIPN-OMe.5.fid
Solvent: CDCl3
Frequency: 100.63
f1 (ppm)

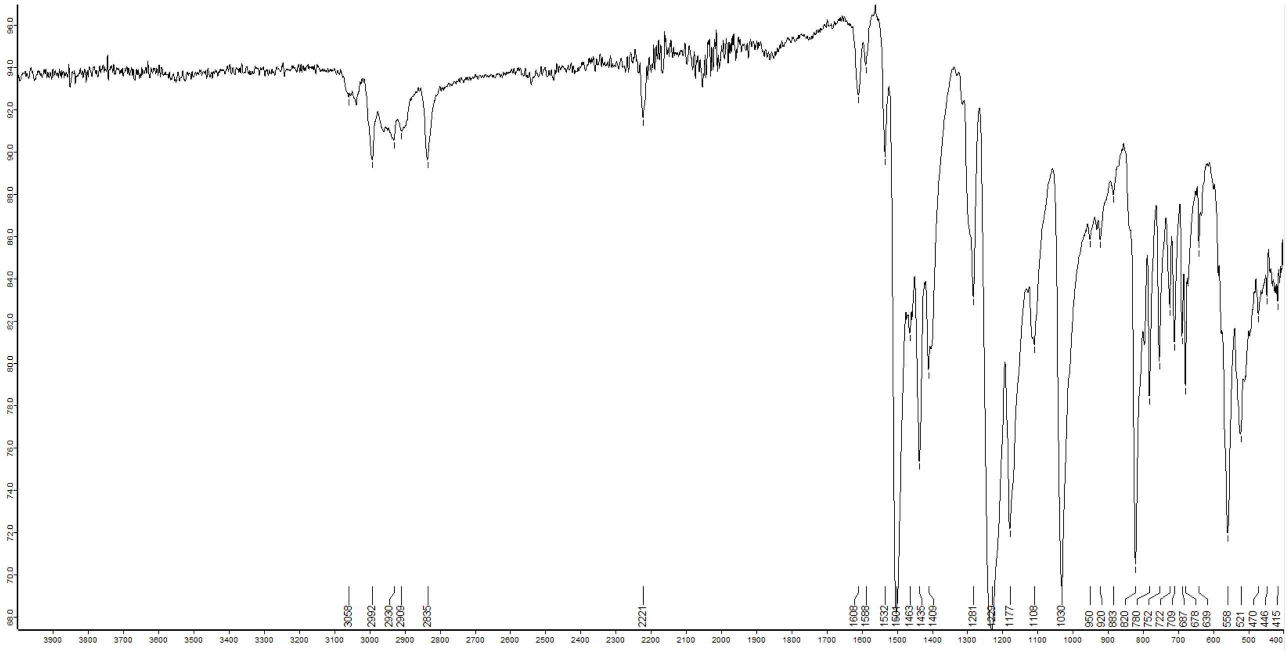

^{1}H and ^{13}C NMR and IR spectra of 4DPATPN-OMe (9b)

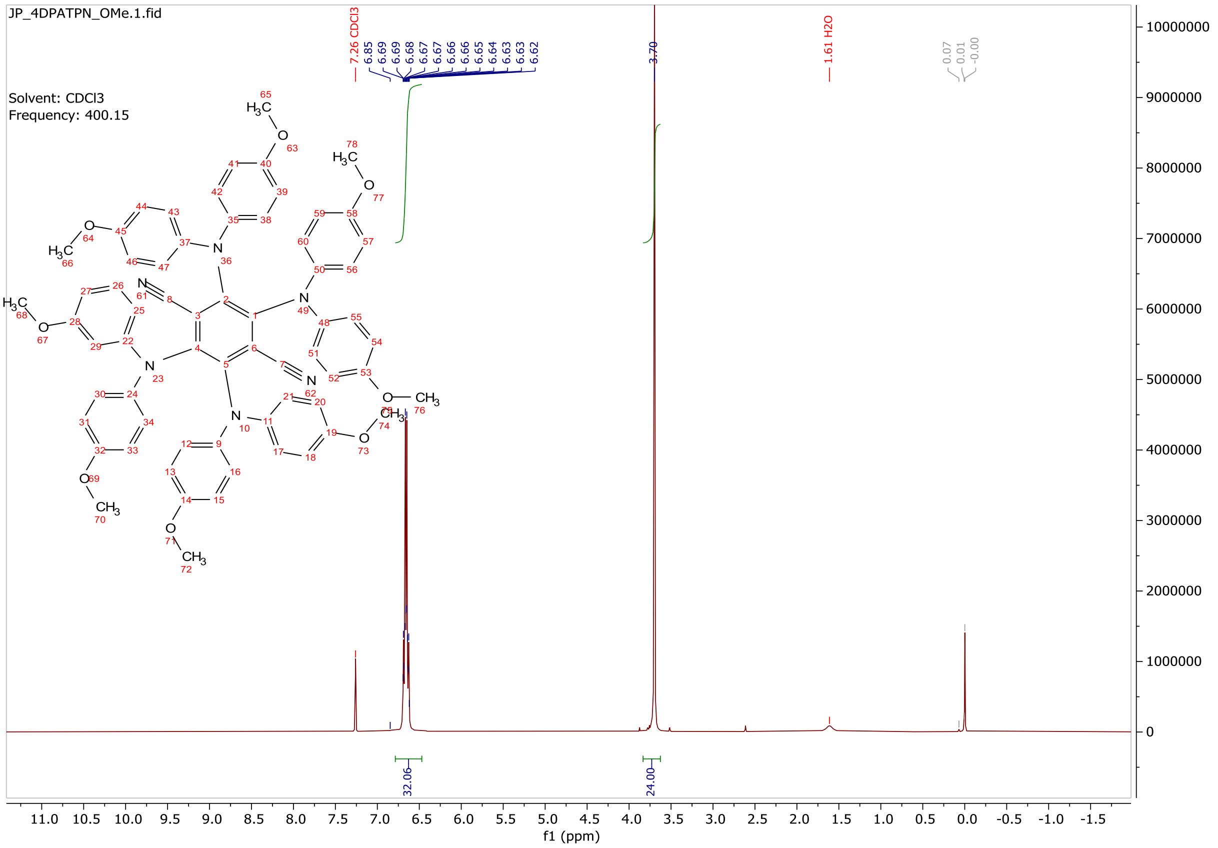

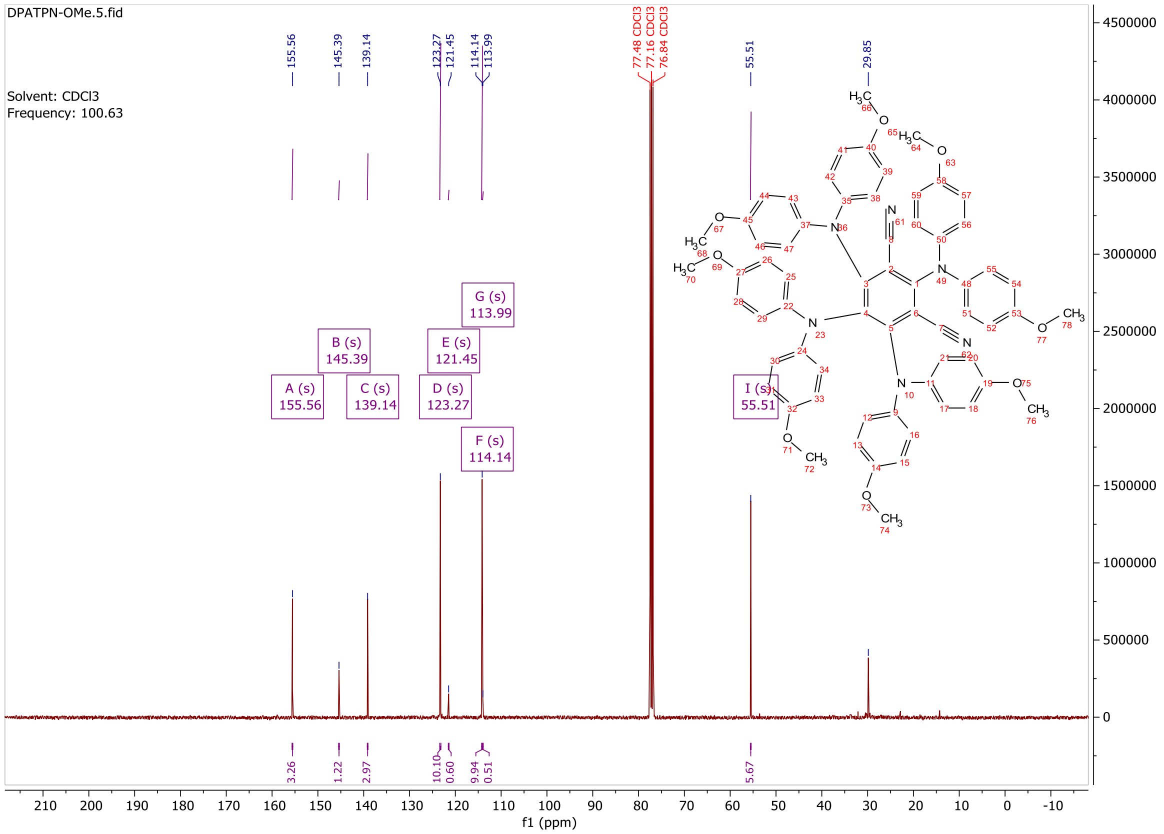

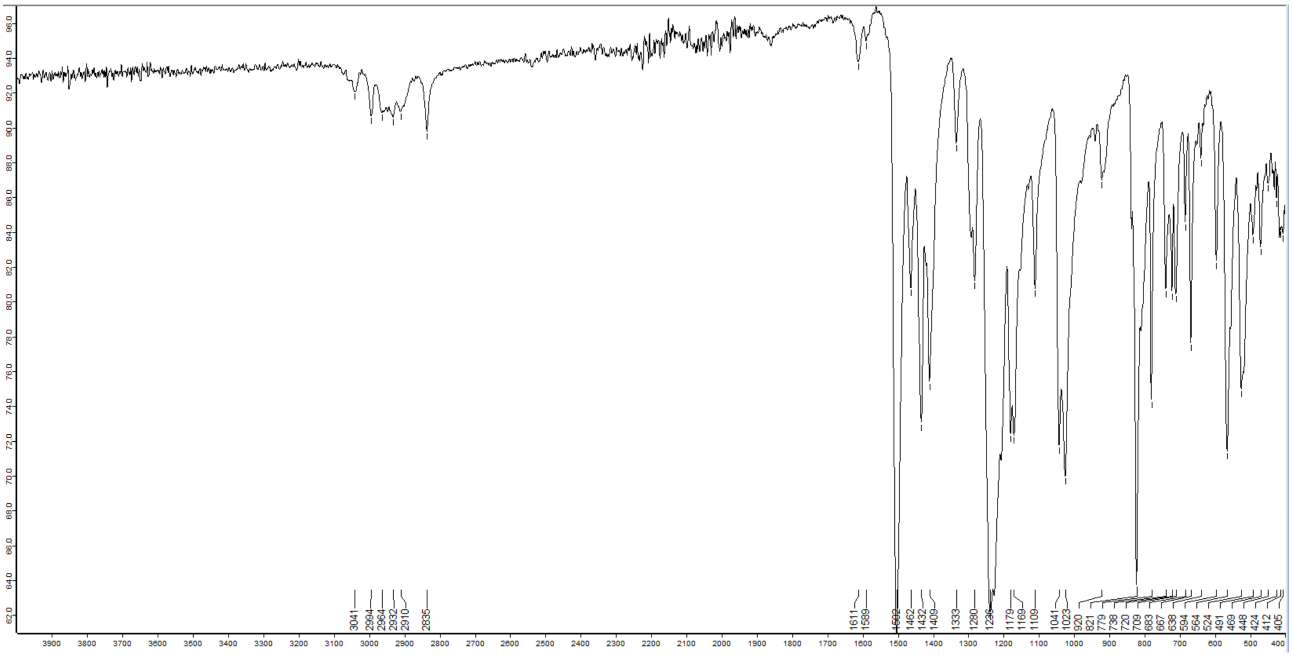

^{1}H and ^{13}C NMR and IR spectra of 4DPAPN-[t]Bu (7c)

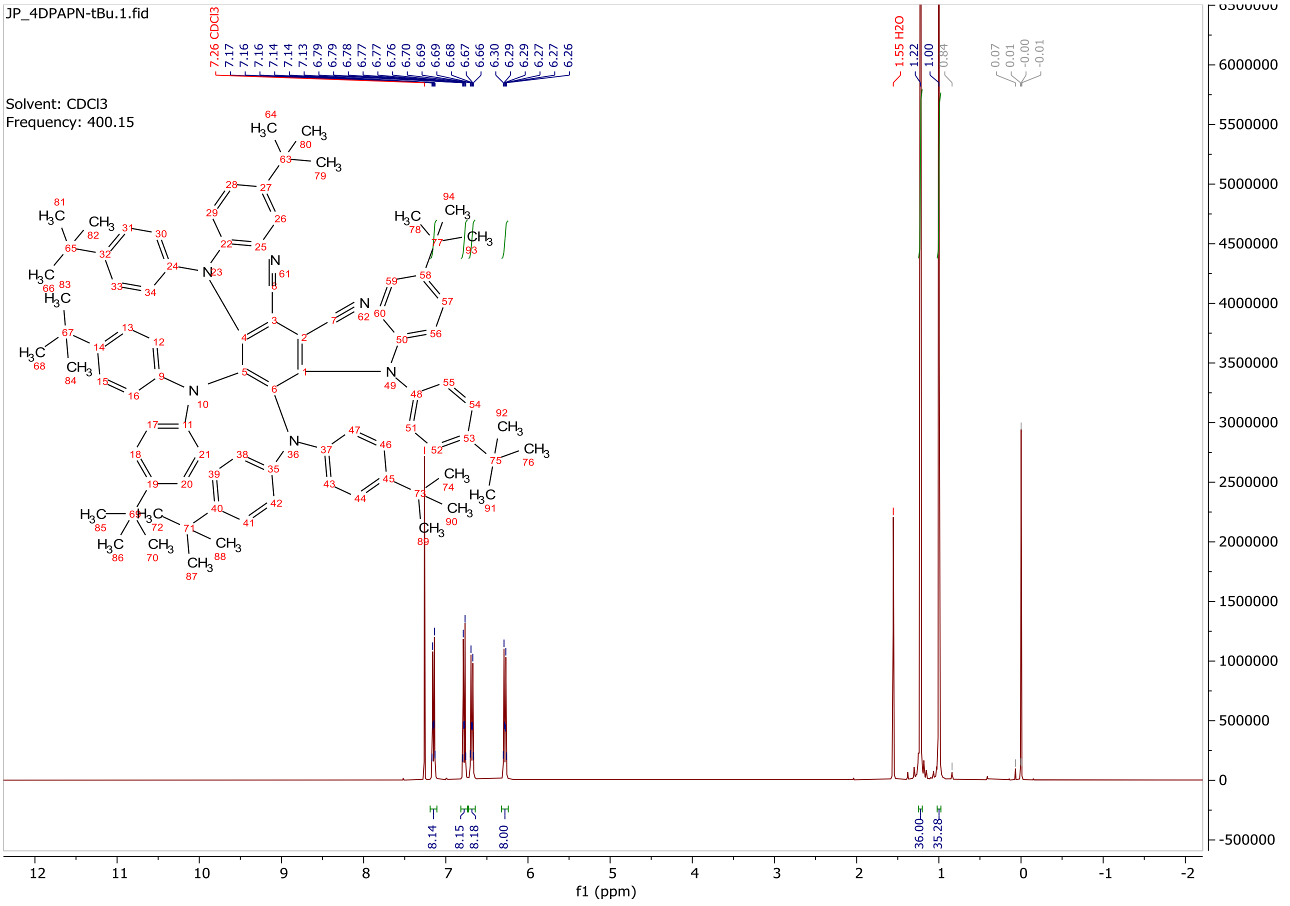

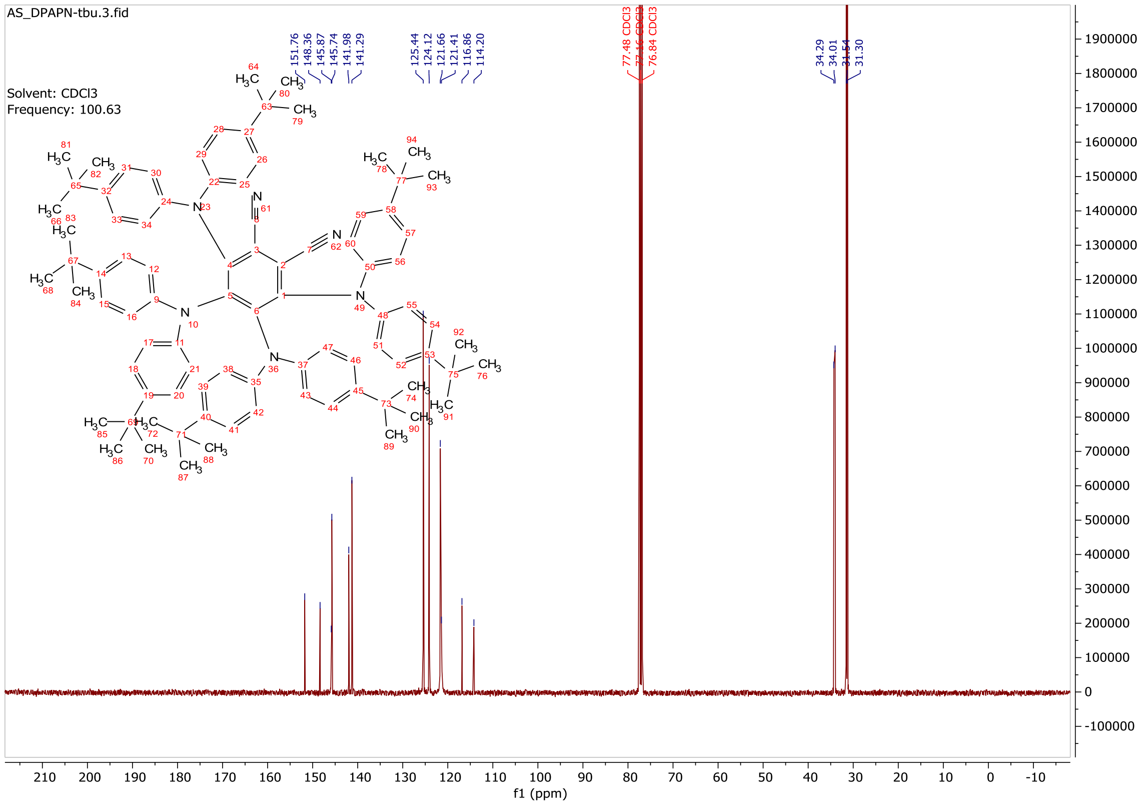
AS_DPAPN-tbu.3.fid
Solvent: CDCl3
Frequency: 100.63
151.76
148.36
145.87
145.74
141.98
141.29
125.44
124.12
121.66
121.41
116.86
114.20
77.48 CDCl3
77.16 CDCl3
76.84 CDCl3
34.29
34.01
31.54
31.30
f1 (ppm)

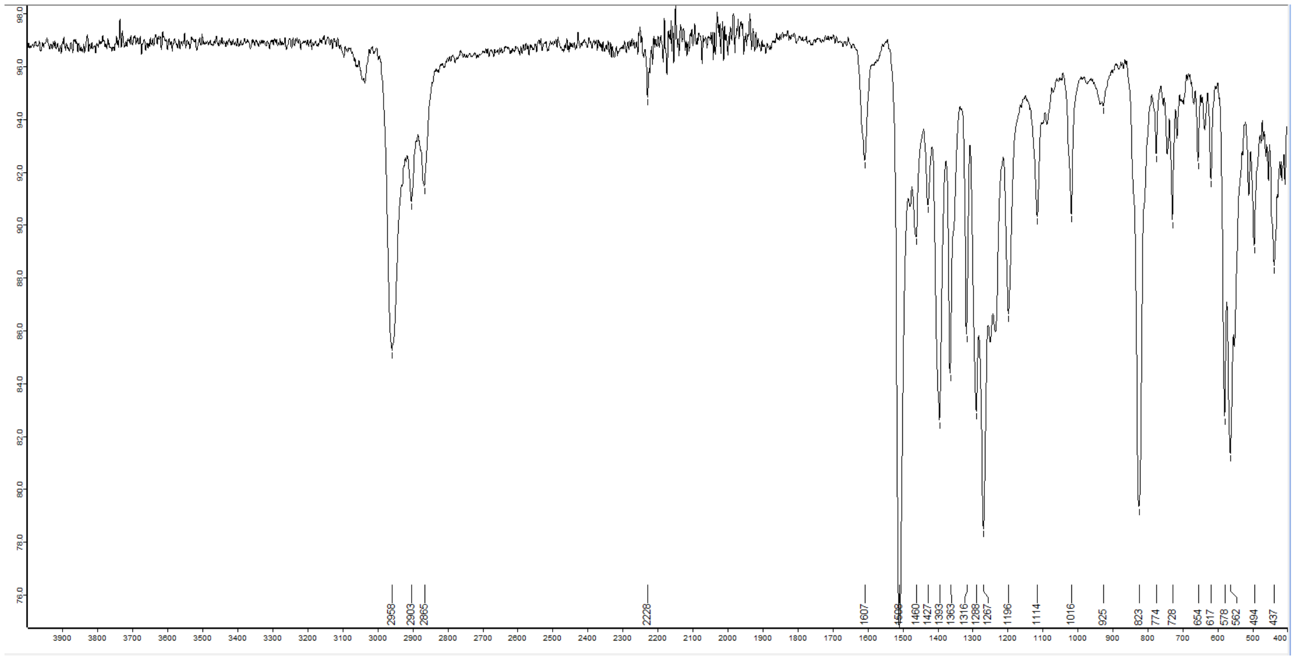
2958
2903
2865
2228
1607
1508
1460
1427
1393
1363
1316
1288
1267
1196
1114
1016
925
823
774
728
654
617
578
562
494
437

^{1}H and ^{13}C NMR and IR spectra of 4DPAIPN-^{t}Bu (7d)

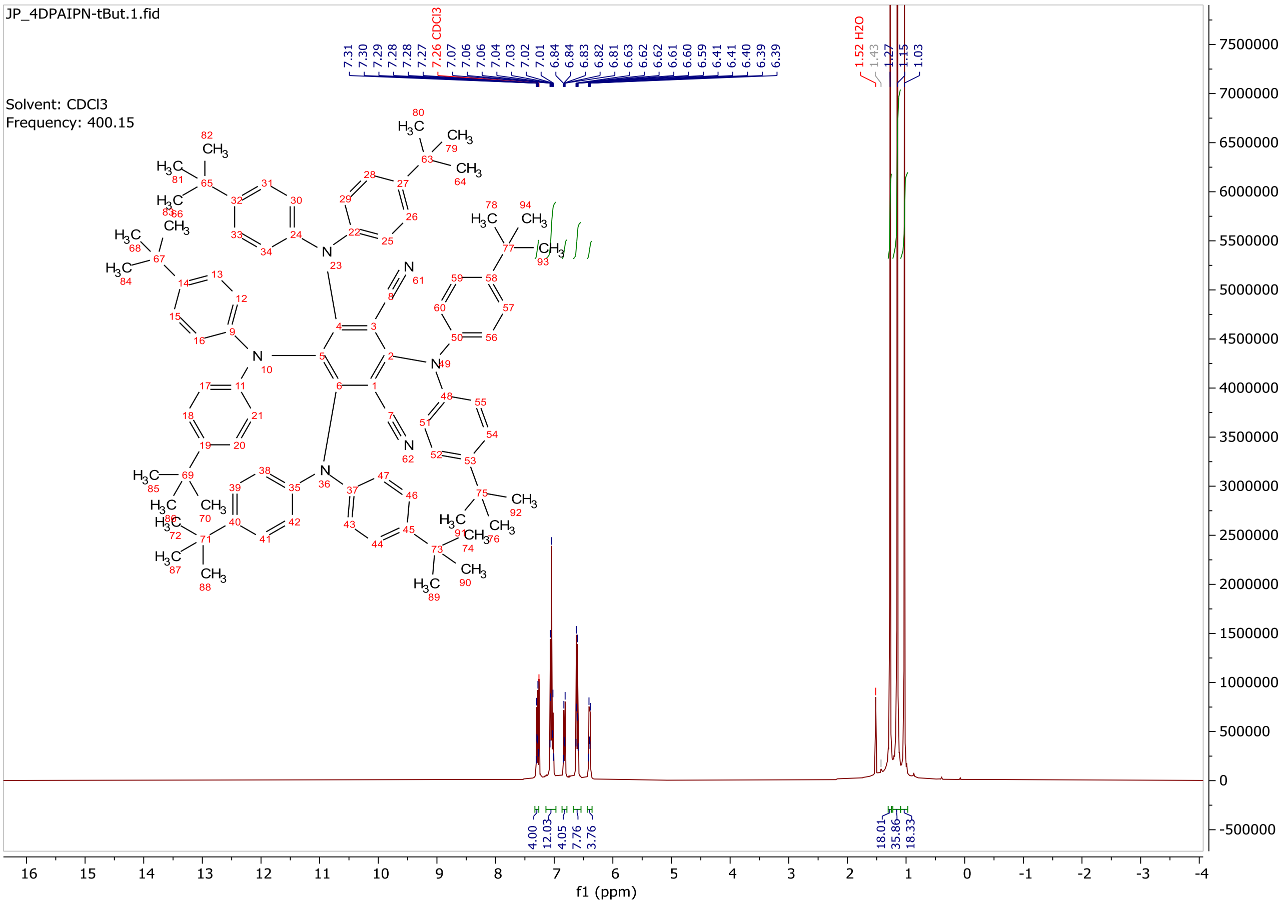

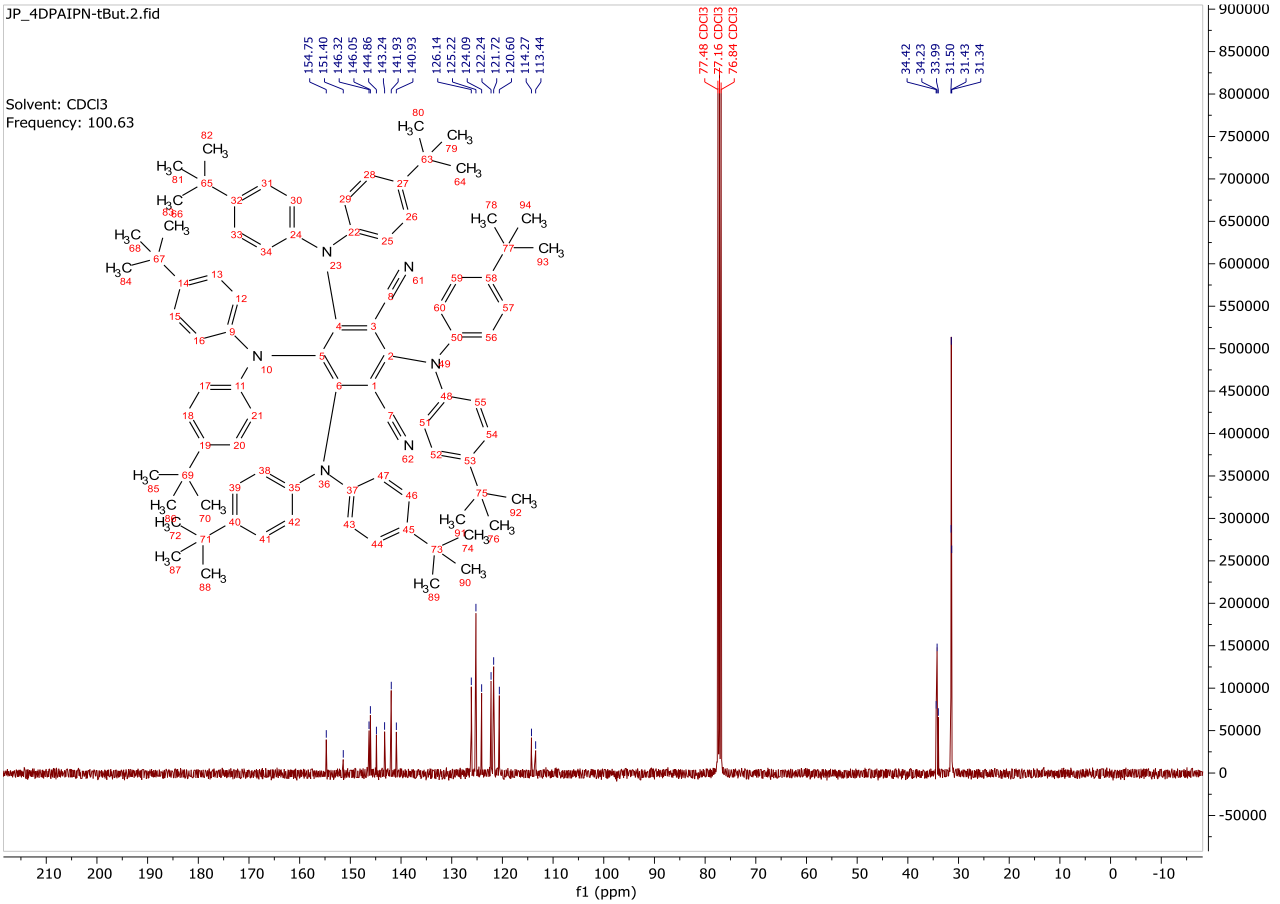

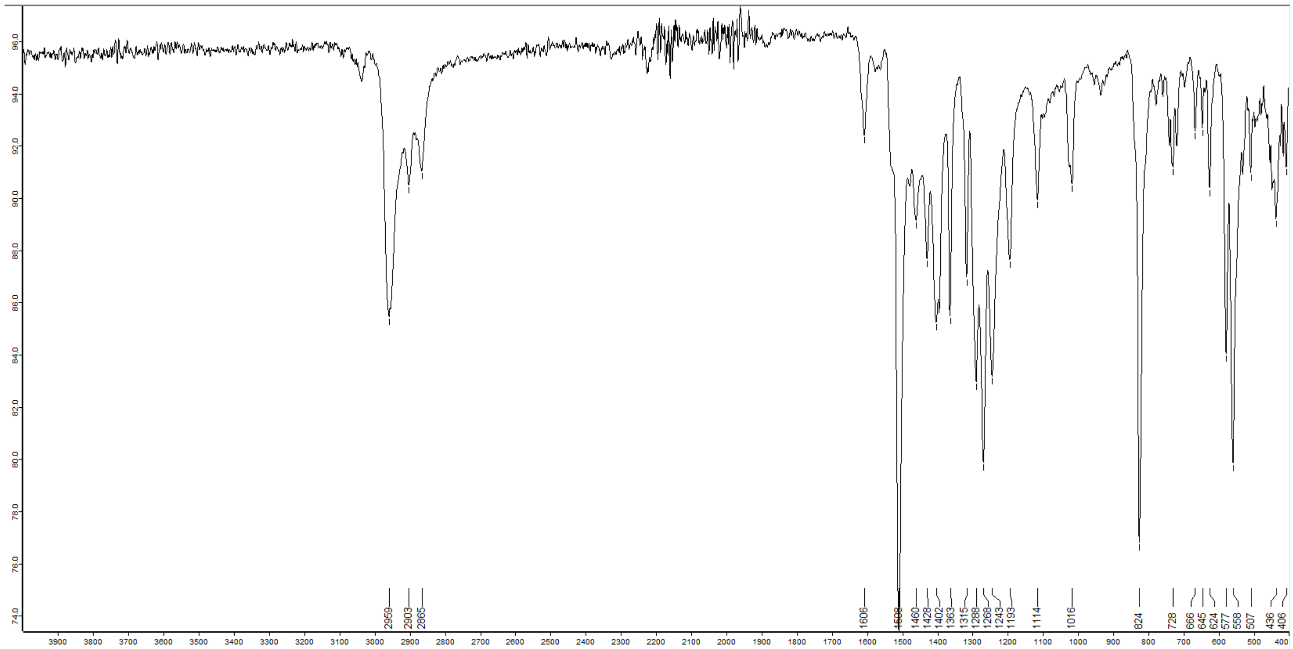

^{1}H and ^{13}C NMR and IR spectra of 4DPATPN-^{t}Bu (9c)

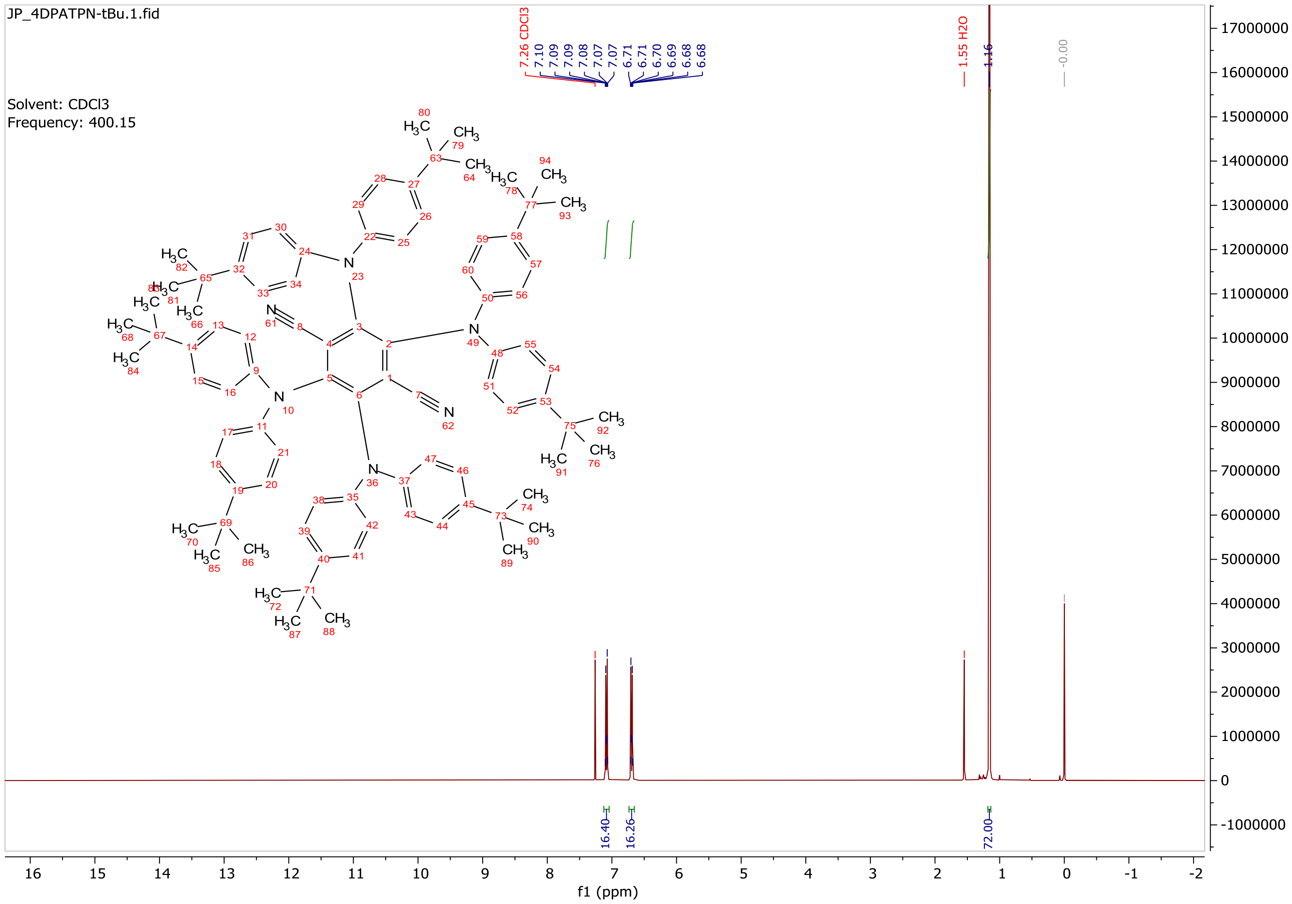

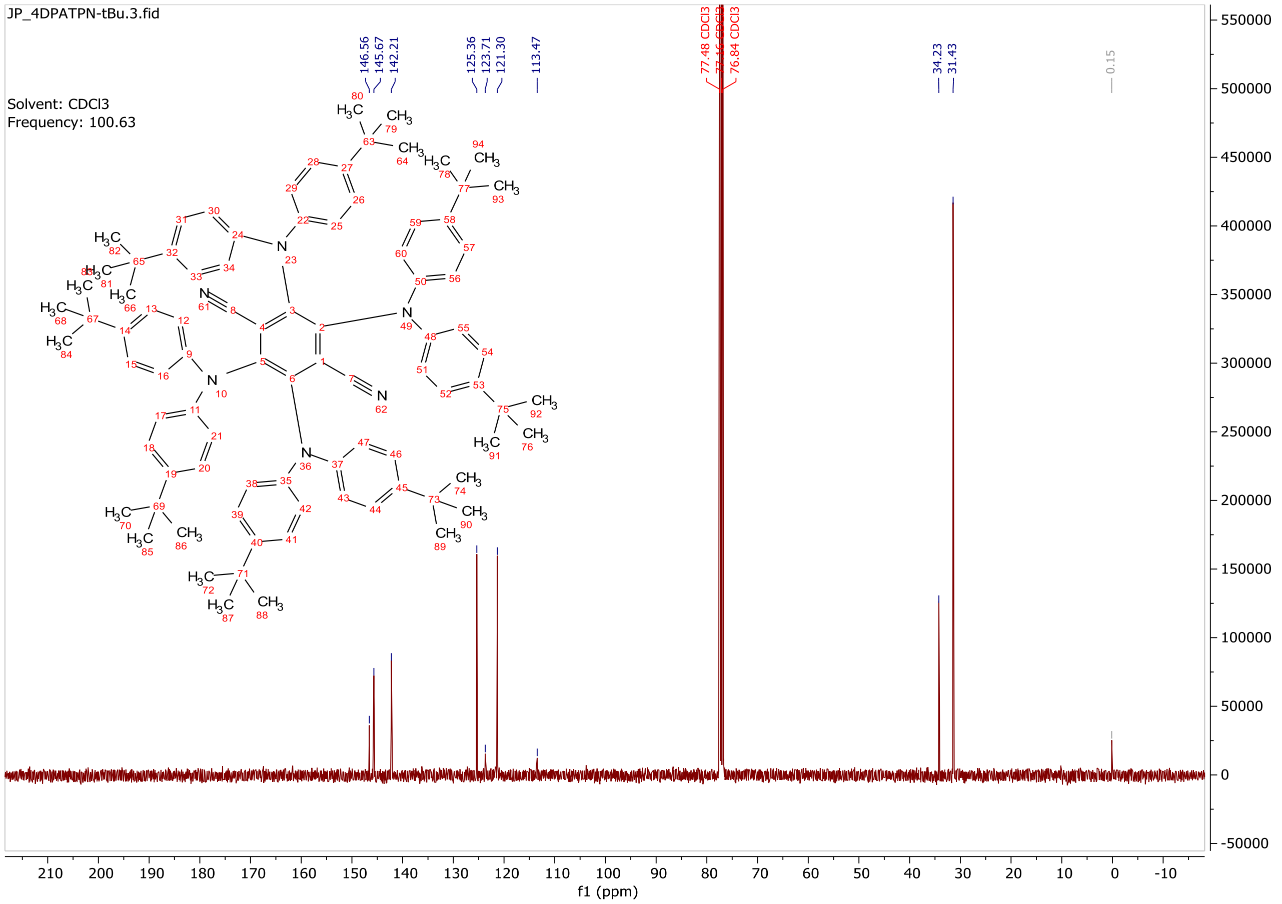

JP_4DPATPN-tBu.3.fid
Solvent: CDCl3
Frequency: 100.63
146.56
145.67
142.21
125.36
123.71
121.30
113.47
77.48 CDCl3
76.84 CDCl3
34.23
31.43
0.15
f1 (ppm)

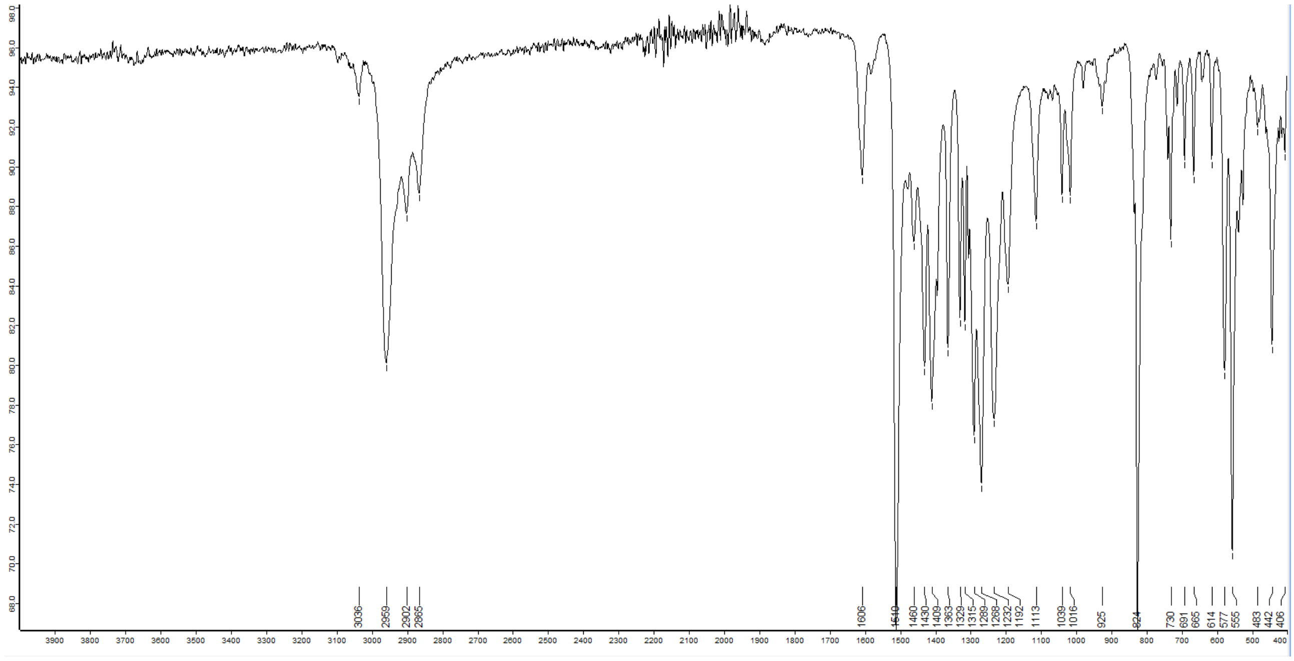

3036
2959
2902
2865
1606
1510
1460
1430
1409
1363
1329
1315
1289
1268
1232
1192
1113
1039
1016
925
824
730
691
665
614
577
555
483
442
406

^{1}H and ^{13}C NMR and IR spectra of 4DPAPN-Ph (7d)

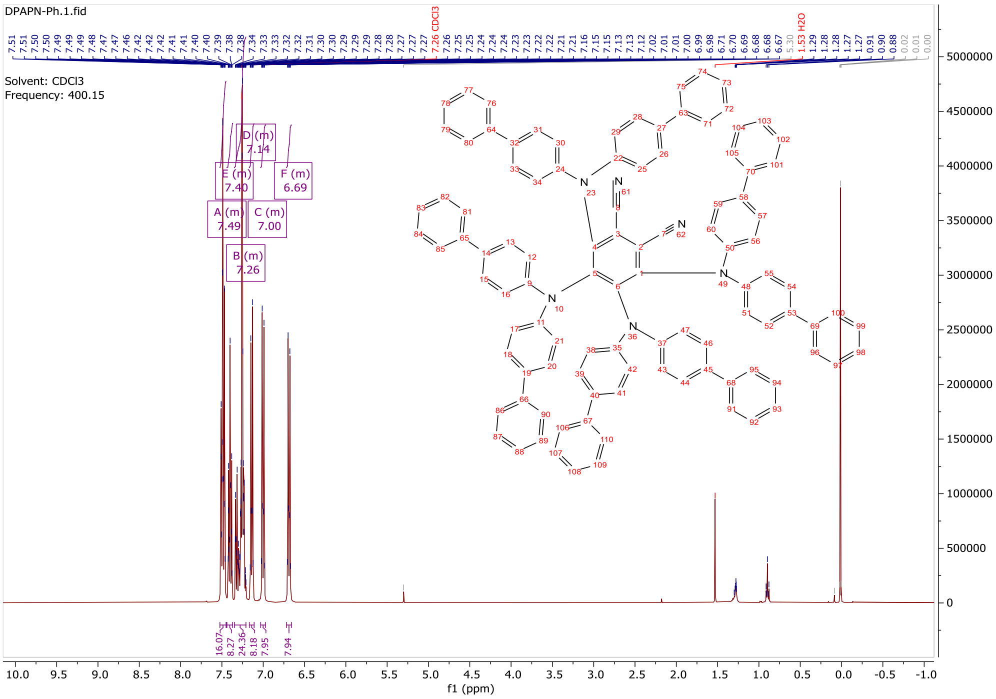

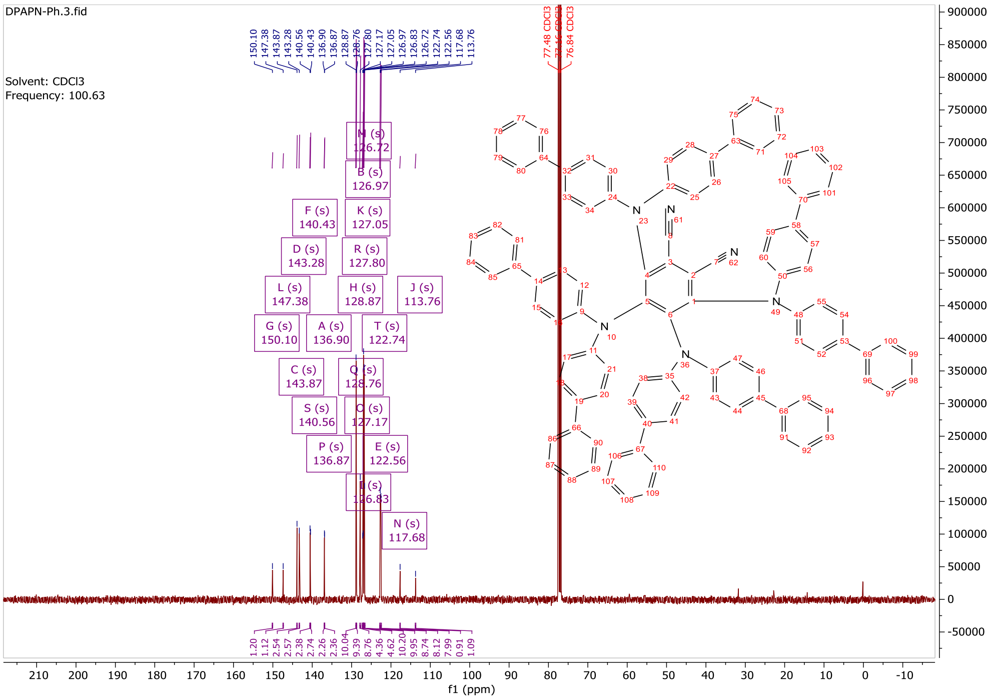

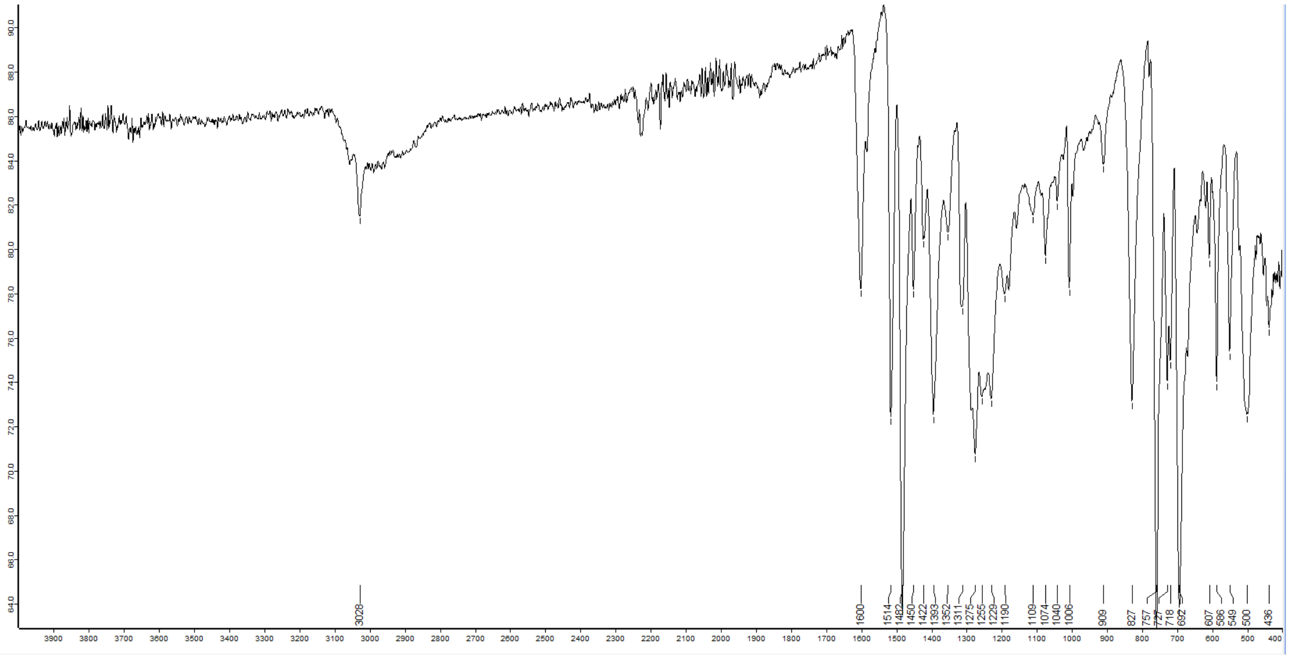

^{1}H and ^{13}C NMR and IR spectra of 4DPAIPN-Ph (8d)

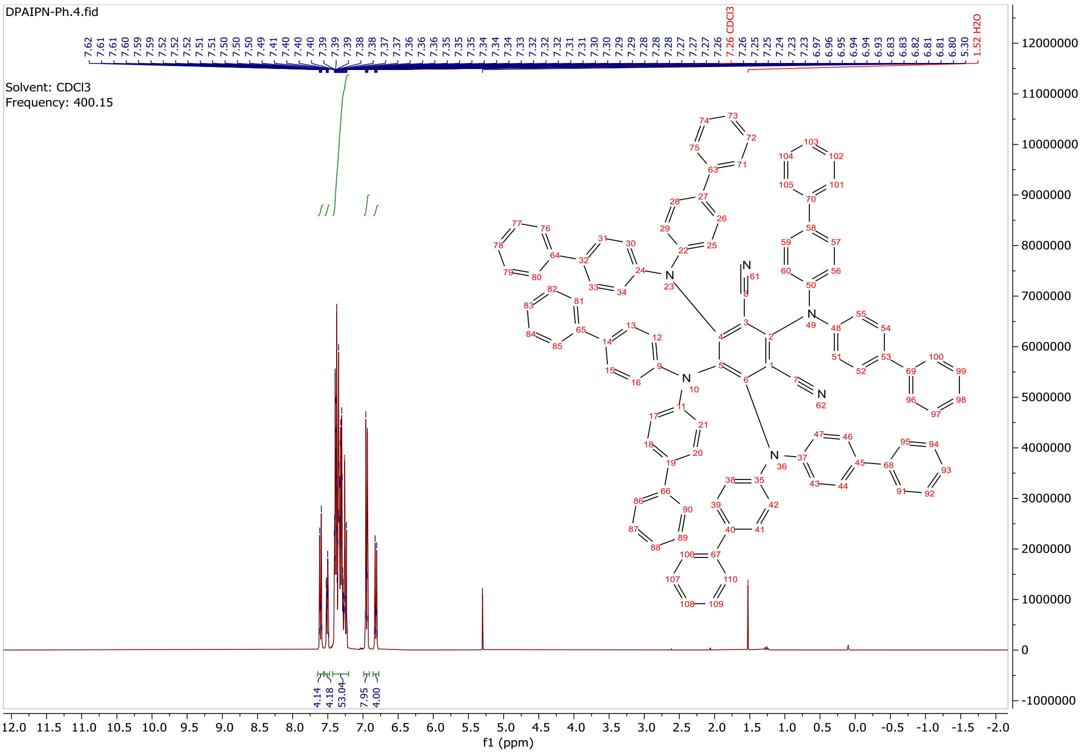

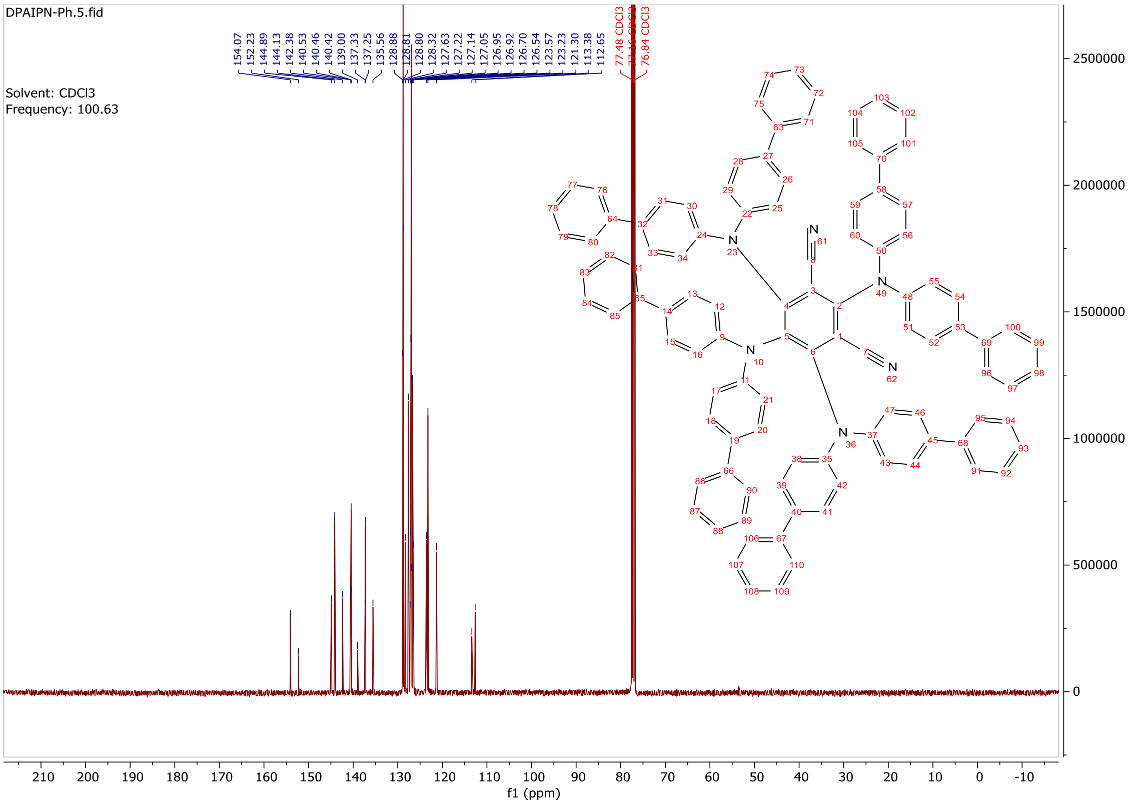
DPAIPN-Ph.5.fid
Solvent: CDCl3
Frequency: 100.63
154.07
152.23
144.89
144.13
142.38
140.53
140.46
140.42
139.00
137.33
137.25
135.56
128.88
128.81
128.80
128.32
127.63
127.22
127.14
127.05
126.95
126.92
126.70
126.54
123.57
123.23
121.30
113.38
112.65
77.48 CDCl3
76.84 CDCl3
f1 (ppm)

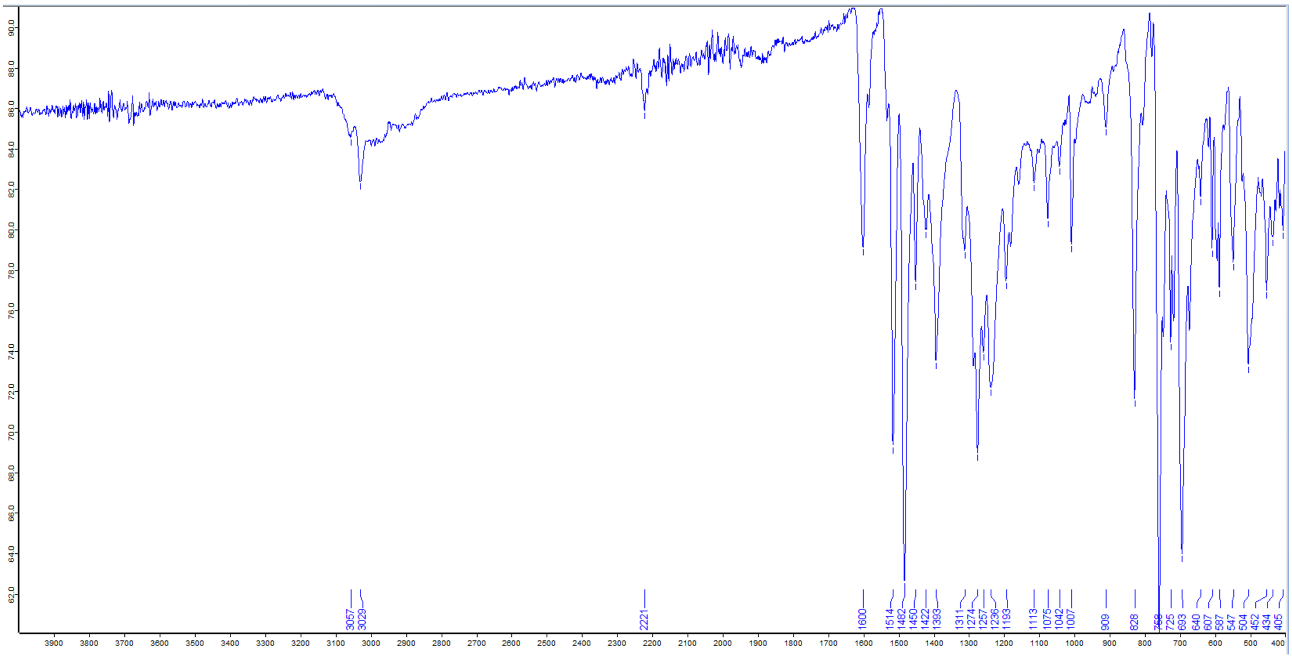
3057
3029
2221
1600
1514
1482
1450
1422
1393
1311
1274
1257
1236
1193
1113
1075
1042
1007
909
828
725
693
640
607
587
547
504
452
434
405

^{1}H and IR spectra of 4DPATPN-Ph (9d)

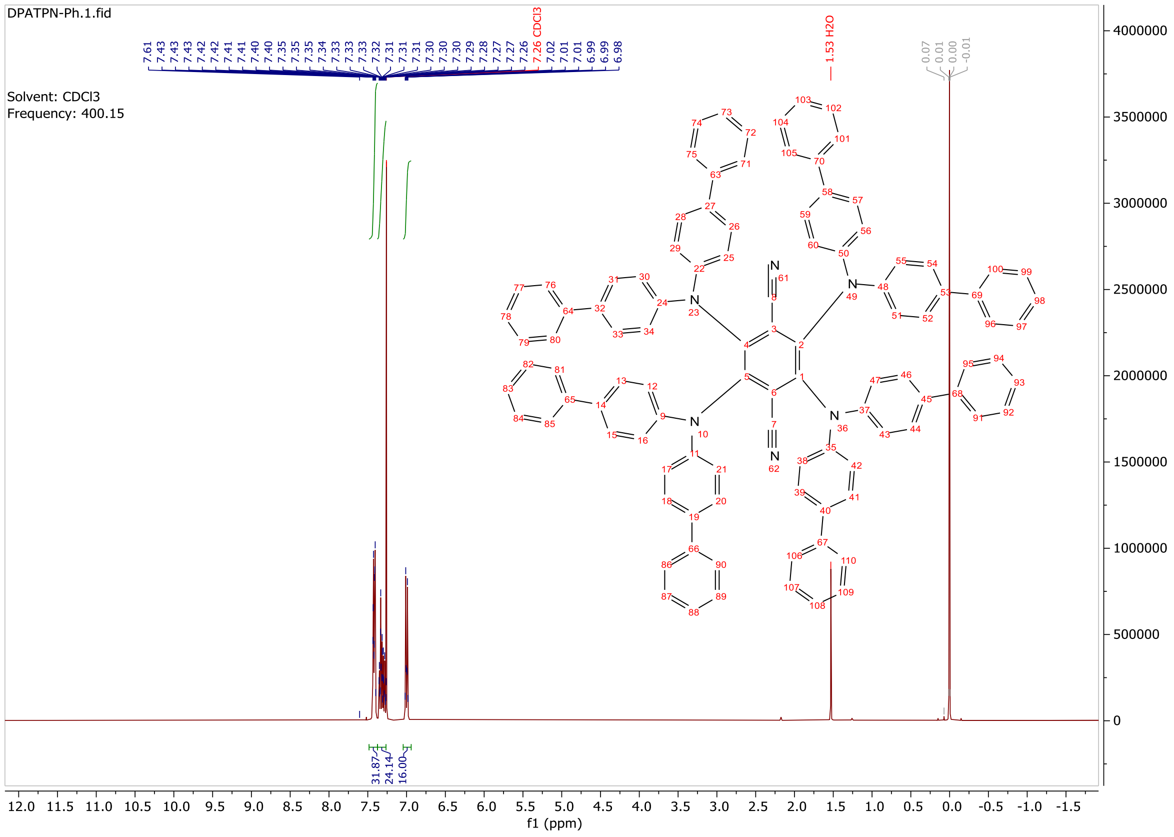

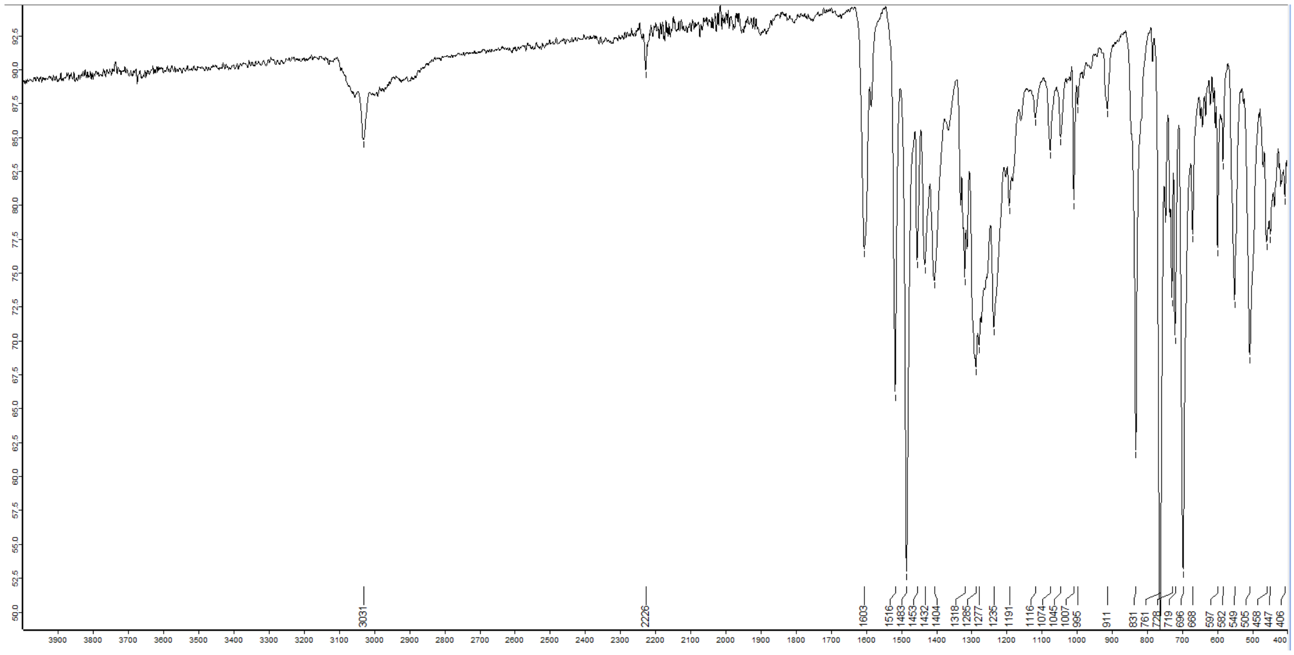

^{1}H NMR spectrum of 10-(naphthalen-1-yl)-10H-phenoxazine

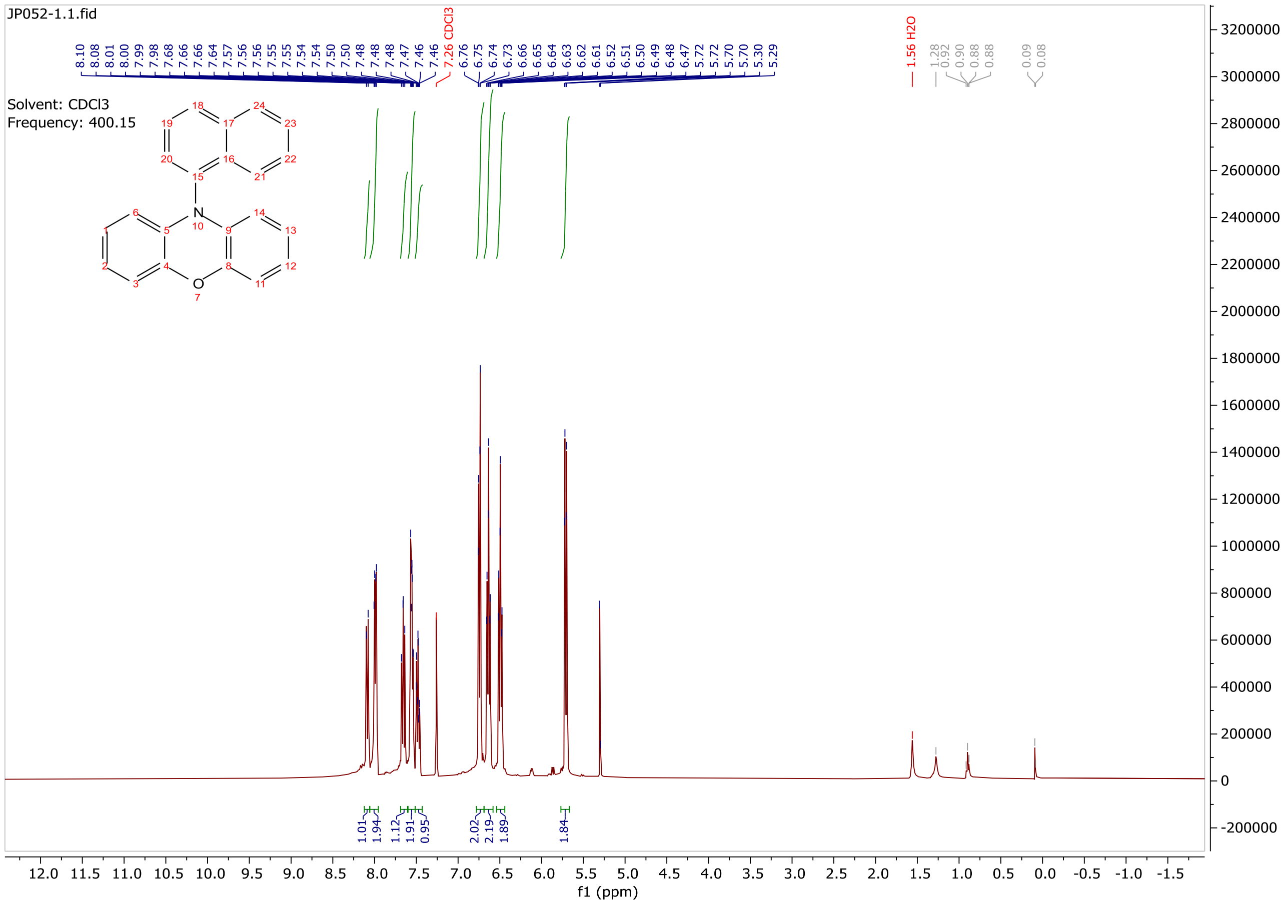

17.2 NMR AND IR SPECTRA OF O-ARYL CARBAMATES

^{1}H, ^{13}C and ^{19}F NMR spectra of 4-(trifluoromethyl)phenyl morpholine-4-carboxylate (3)

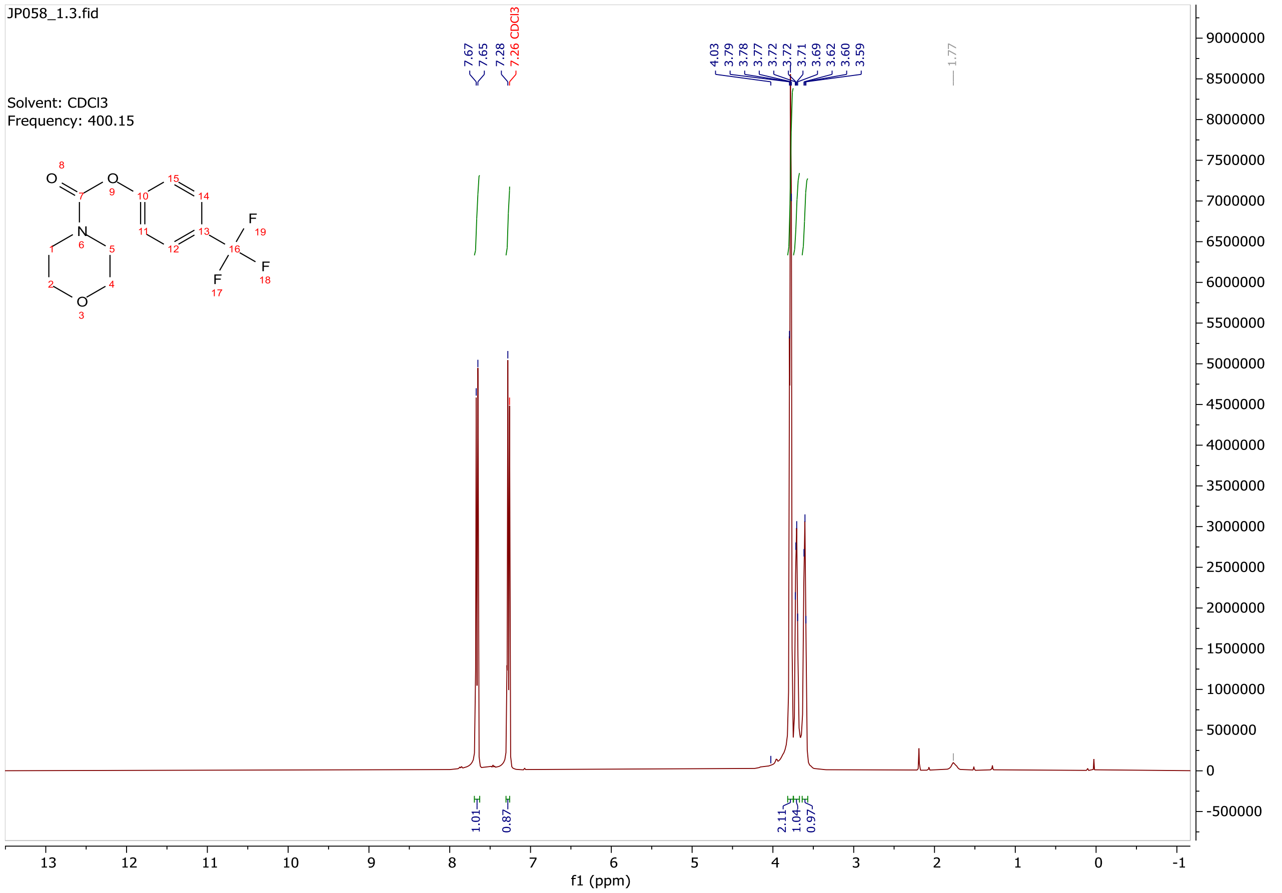

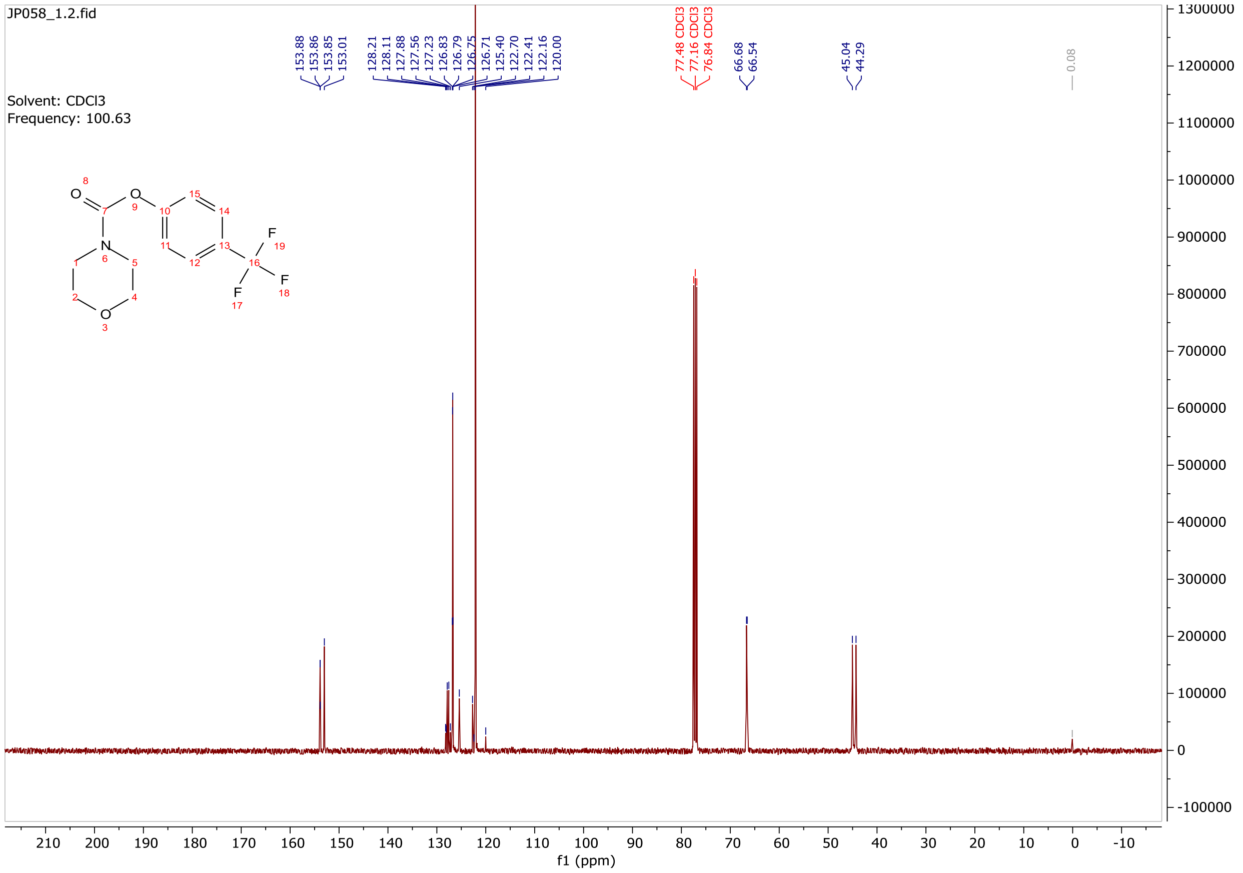

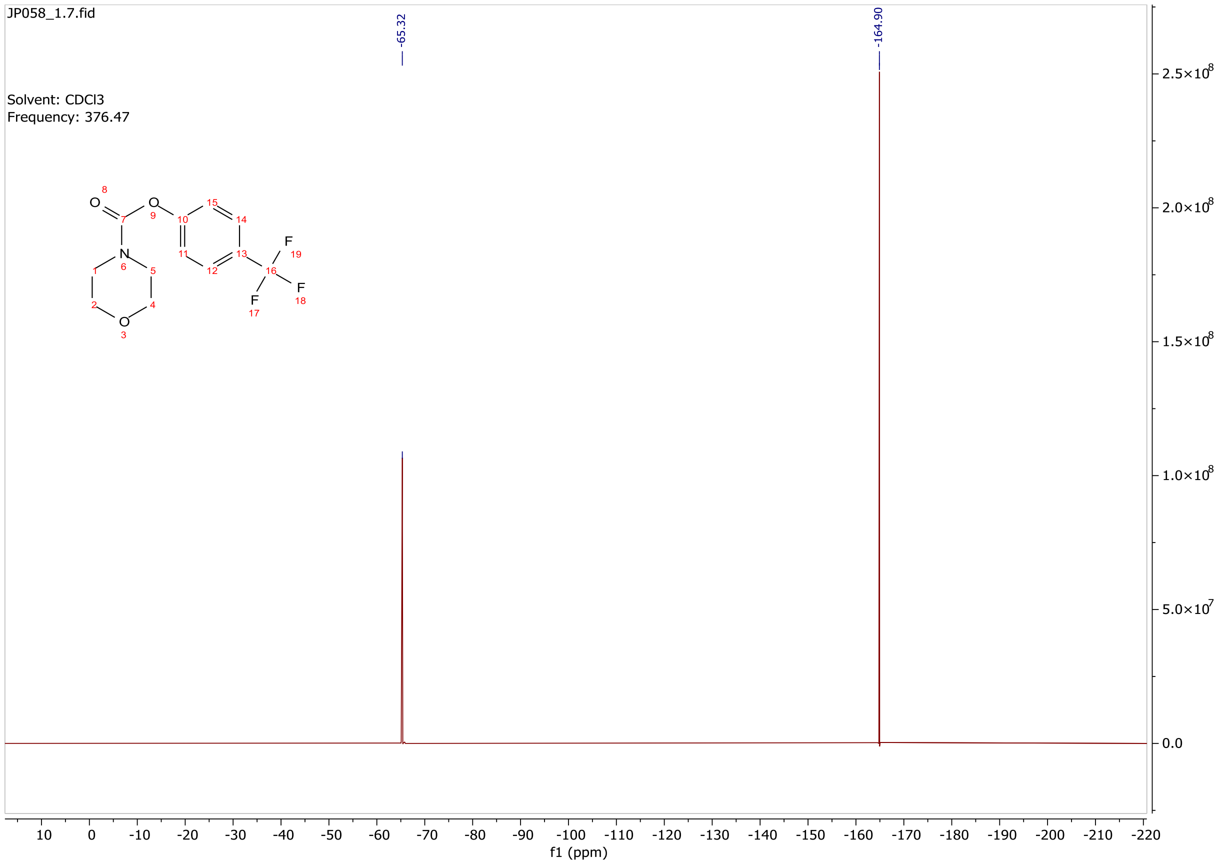

^{1}H, ^{13}C and ^{19}F NMR spectra of 4-(trifluoromethyl)phenyl piperidine-1-carboxylate (10)

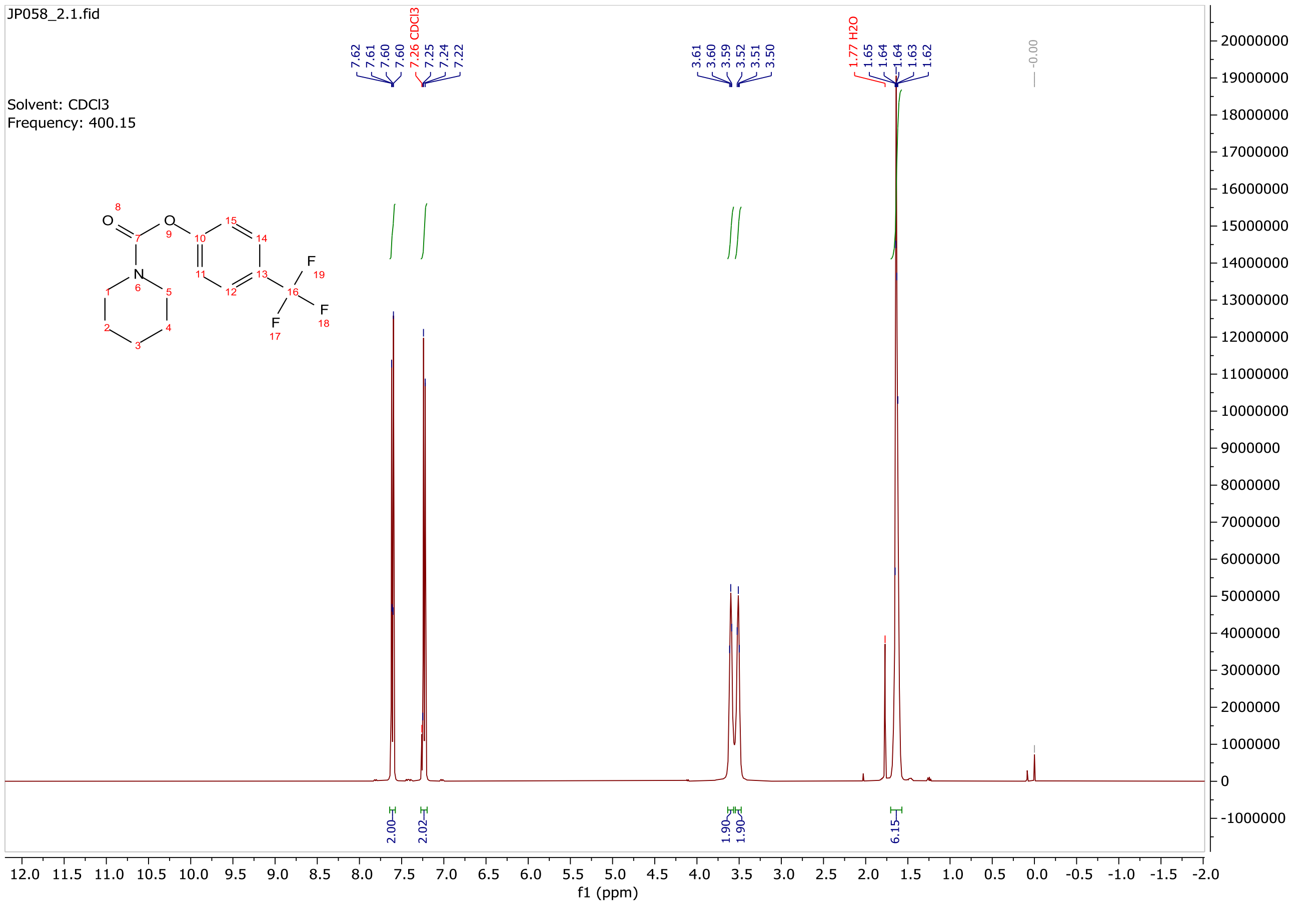

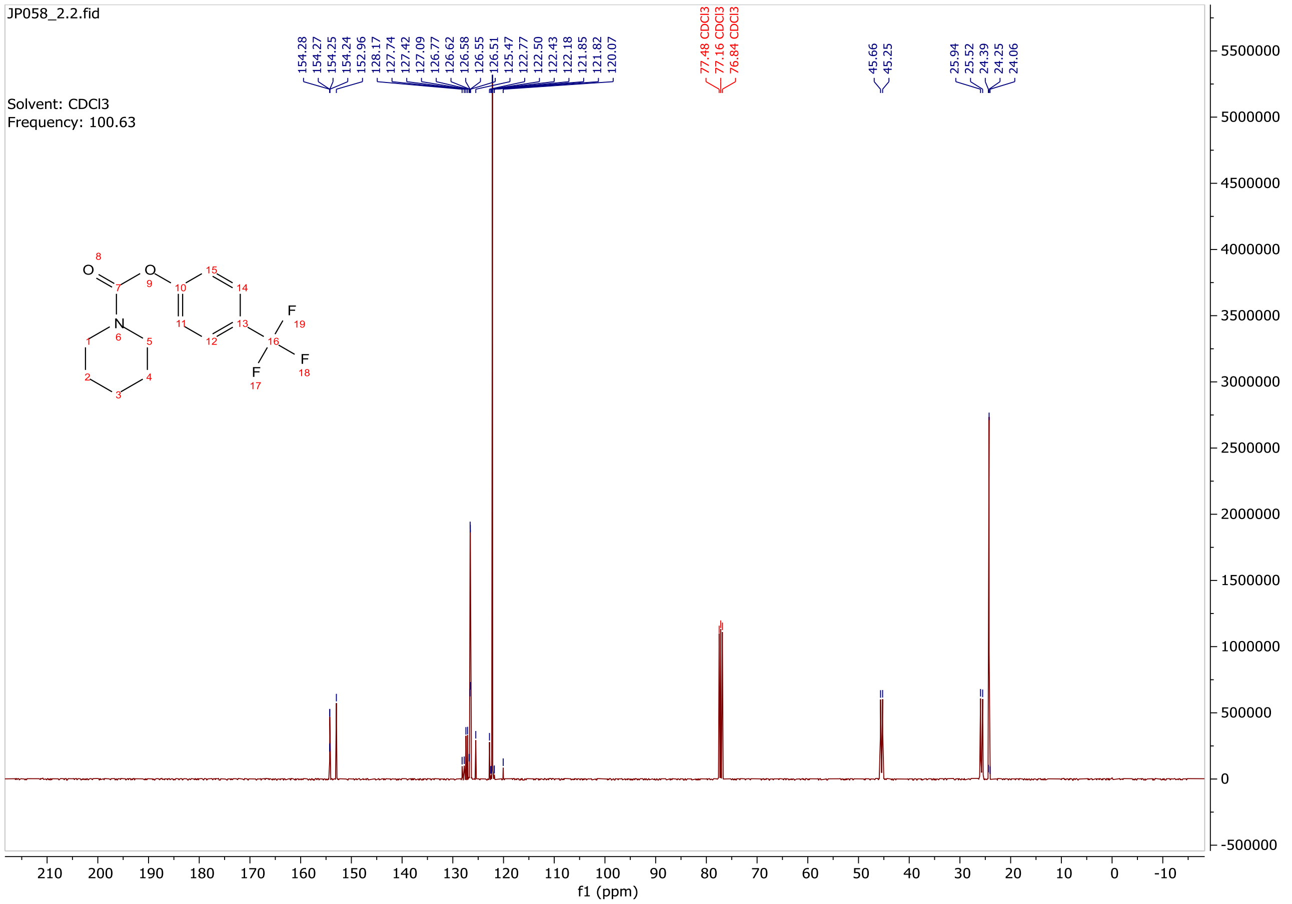
JP058_2.2.fid
Solvent: CDCl3
Frequency: 100.63
154.28
154.27
154.25
154.24
152.96
128.17
127.74
127.42
127.09
126.77
126.62
126.58
126.55
126.51
125.47
122.77
122.50
122.43
122.18
121.85
121.82
120.07
77.48 CDCl3
77.16 CDCl3
76.84 CDCl3
45.66
45.25
25.94
25.52
24.39
24.25
24.06
f1 (ppm)

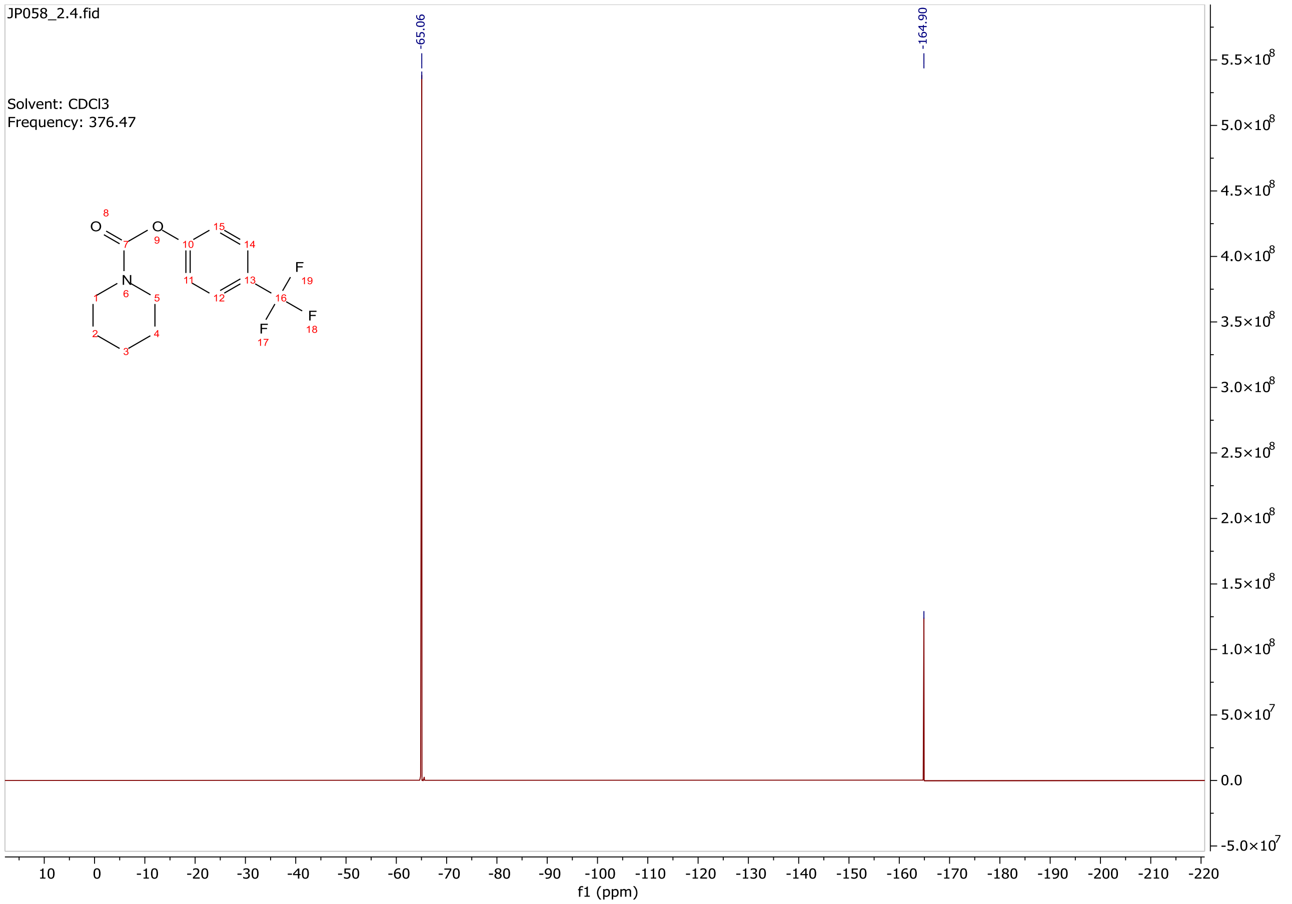
JP058_2.4.fid
Solvent: CDCl3
Frequency: 376.47
-65.06
-164.90
f1 (ppm)

^{1}H, ^{13}C and ^{19}F NMR and IR spectra of 4-(trifluoromethyl)phenyl ethyl(methyl)carbamate (11)

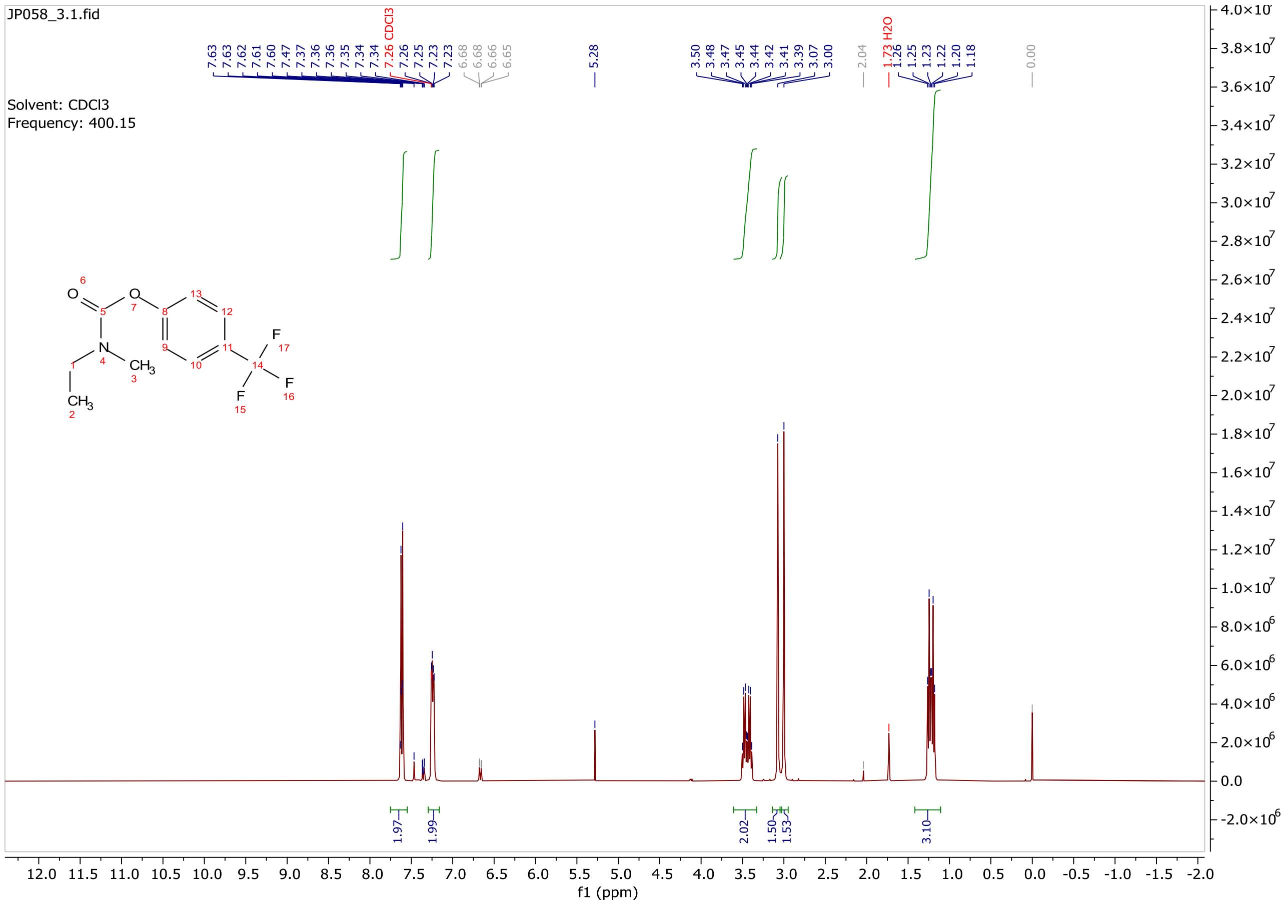

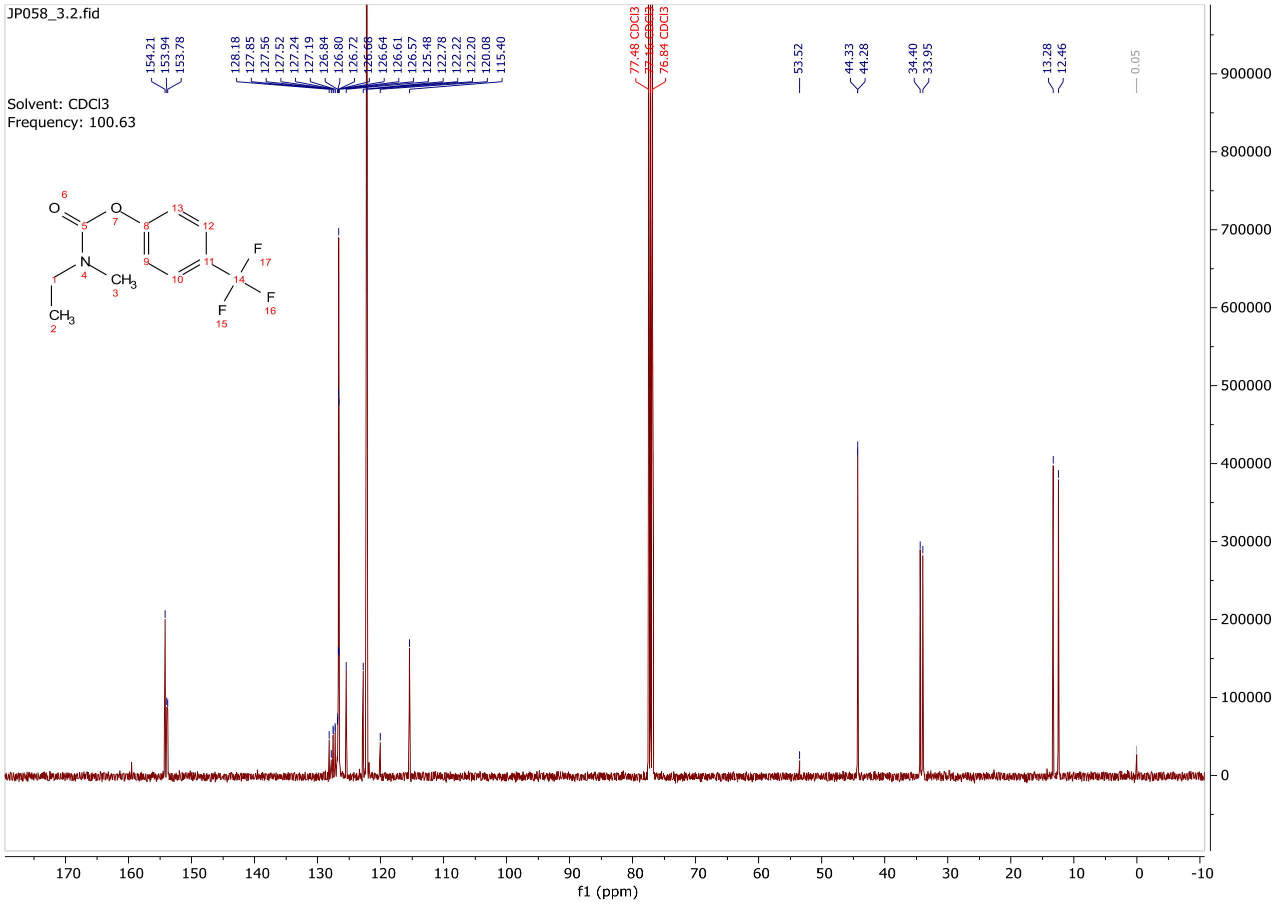

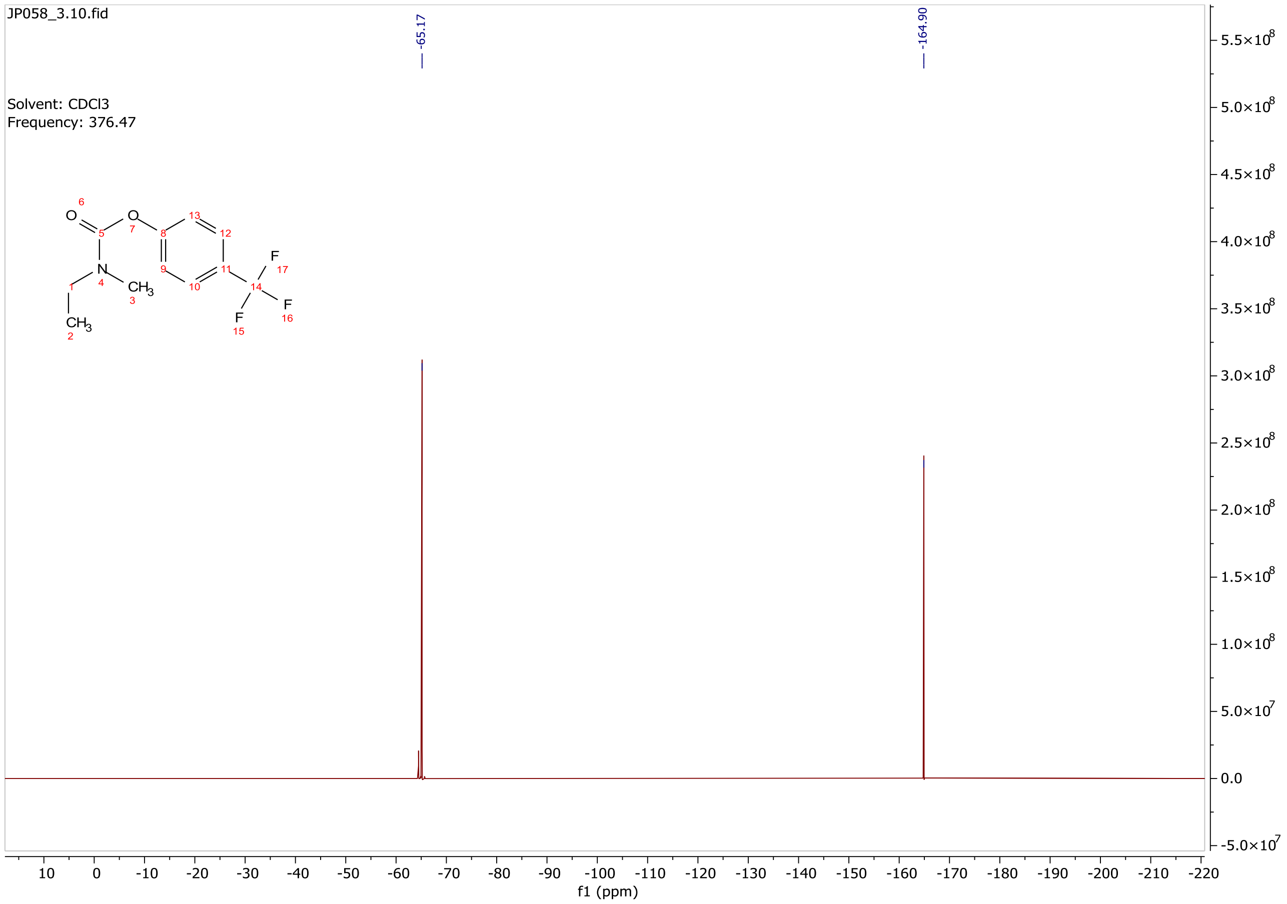
JP058_3.10.fid
Solvent: CDCl3
Frequency: 376.47
-65.17
-164.90
f1 (ppm)

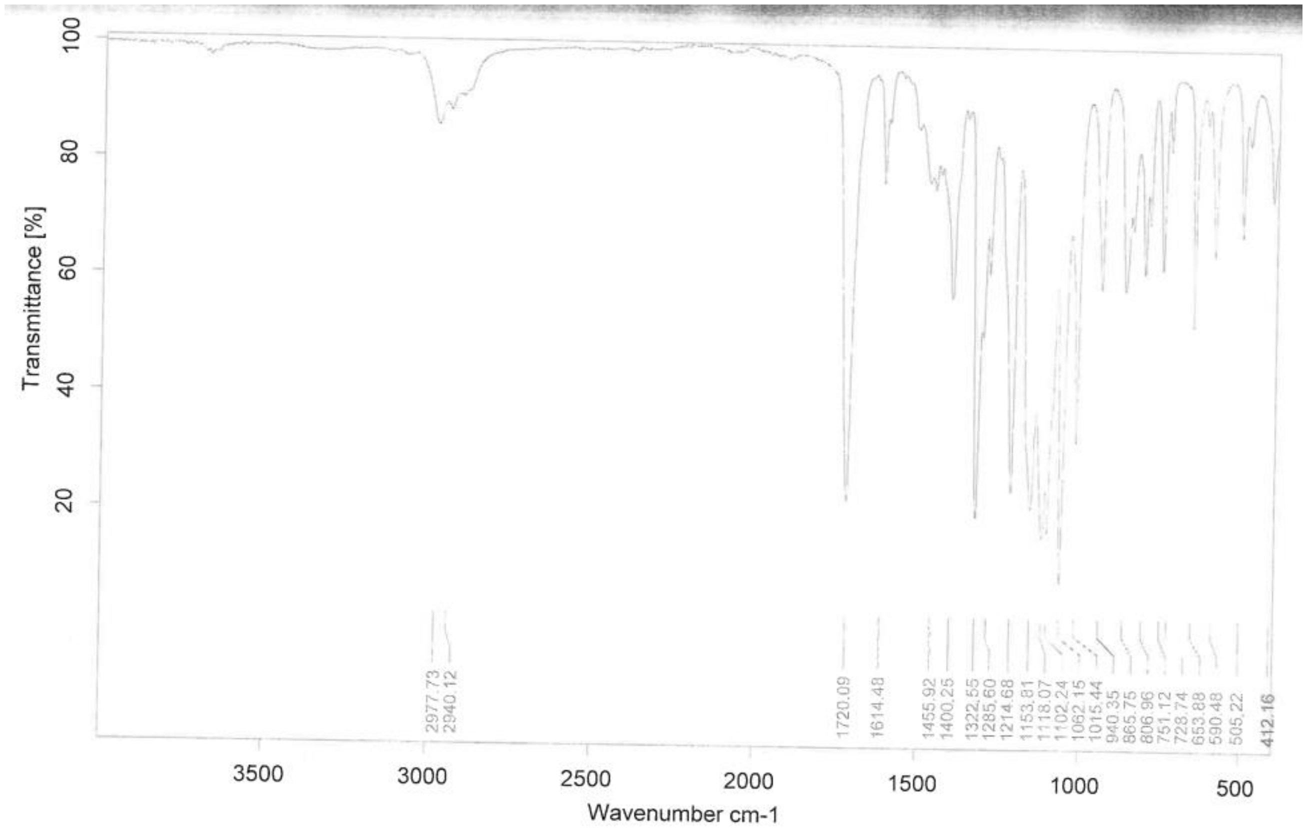
Transmittance [%]
Wavenumber cm-1
2977.73
2940.12
1720.09
1614.48
1455.92
1400.25
1322.55
1285.60
1214.68
1153.81
1118.07
1102.24
1062.15
1015.44
940.35
865.75
806.96
751.12
728.74
653.88
590.48
505.22
412.16

^{1}H, ^{13}C and ^{19}F NMR and IR spectra of 4-(trifluoromethyl)phenyl dibenzylcarbamate (12)

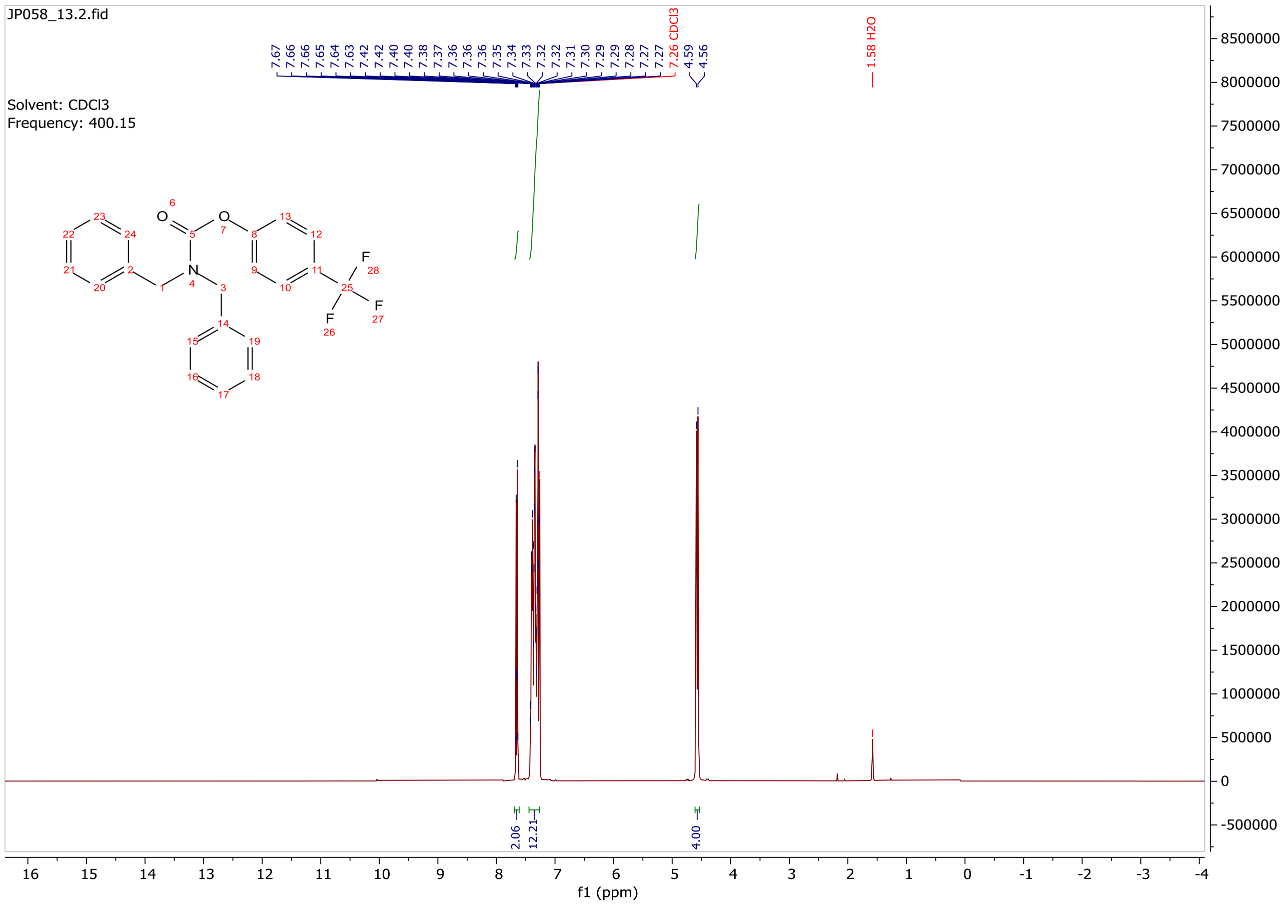

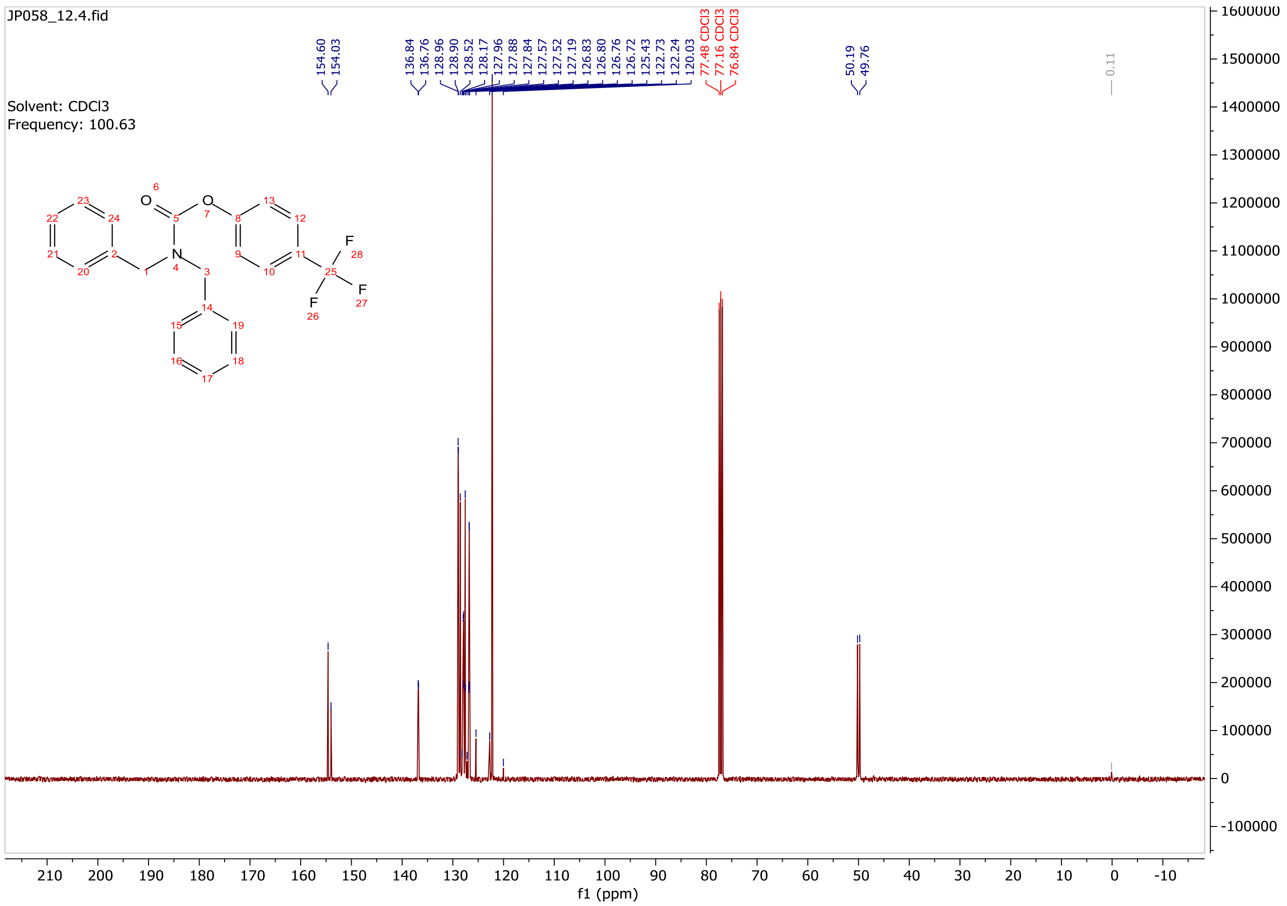

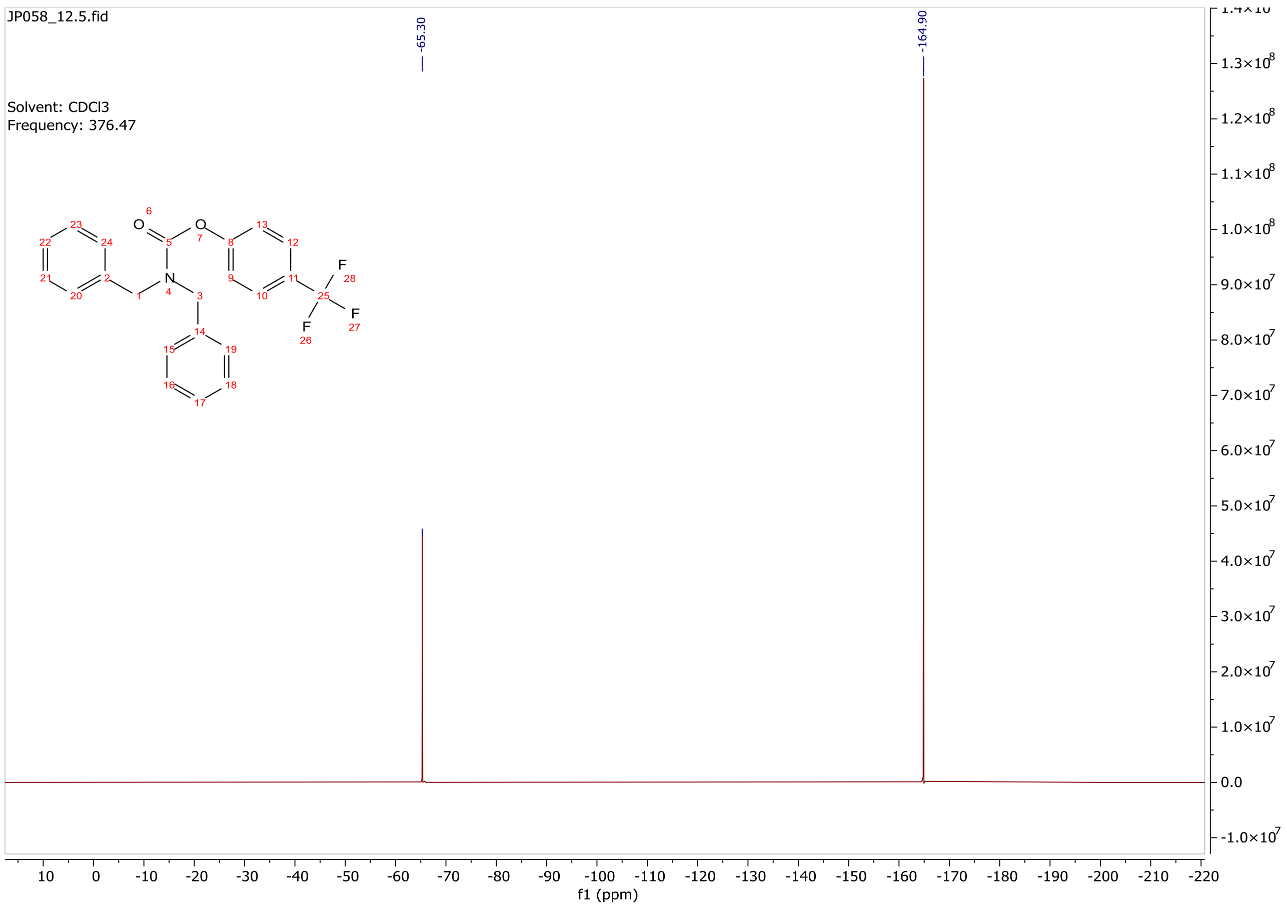
JP058_12.5.fid
Solvent: CDCl3
Frequency: 376.47
-65.30
-164.90
f1 (ppm)

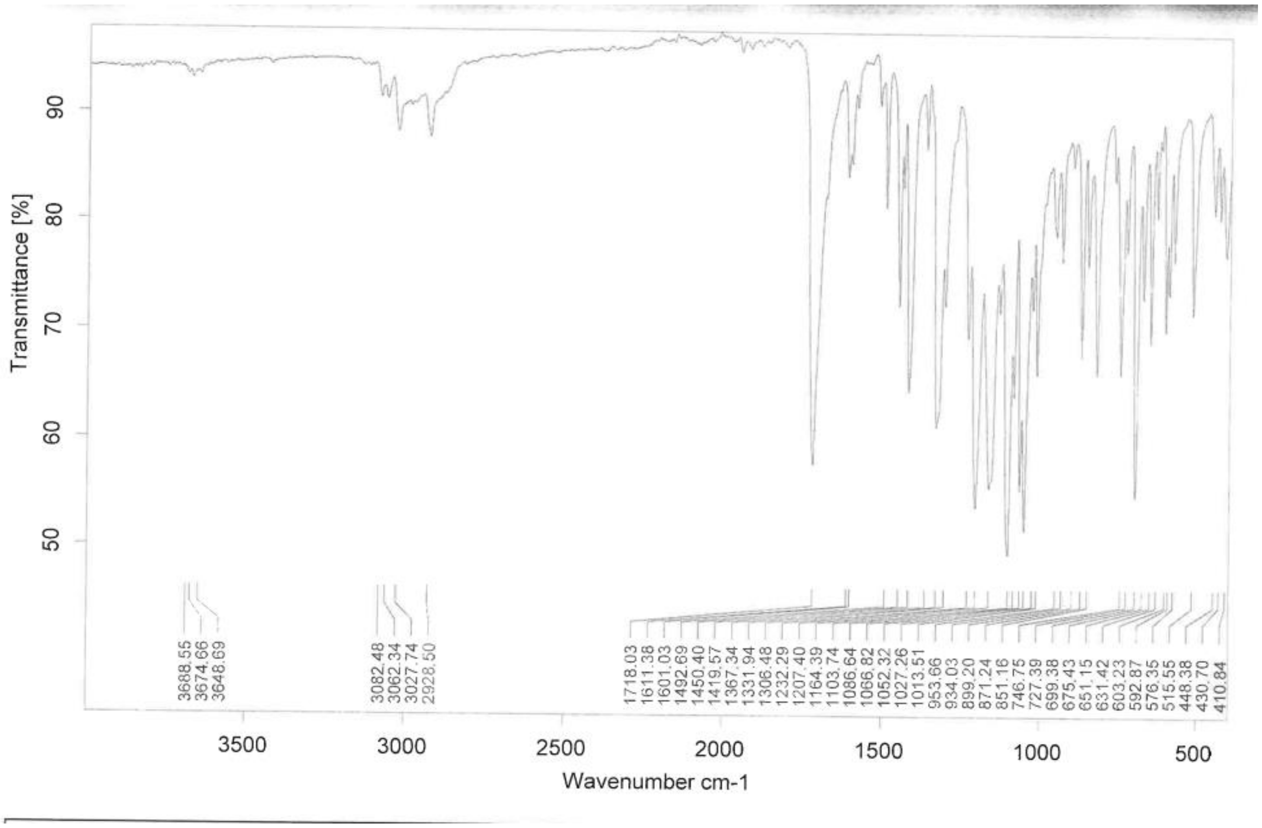
Transmittance [%]
Wavenumber cm-1
3688.55
3674.66
3648.69
3082.48
3062.34
3027.74
2928.50
1718.03
1611.38
1601.03
1492.69
1450.40
1419.57
1367.34
1331.94
1306.48
1232.29
1207.40
1164.39
1103.74
1086.64
1066.82
1052.32
1027.26
1013.51
953.66
934.03
899.20
871.24
851.16
746.75
727.39
699.38
675.43
651.15
631.42
603.23
592.87
576.35
515.55
448.38
430.70
410.84

^{1}H, ^{13}C and ^{19}F NMR spectra of 4-(trifluoromethyl)phenyl diisopropylcarbamate (13)

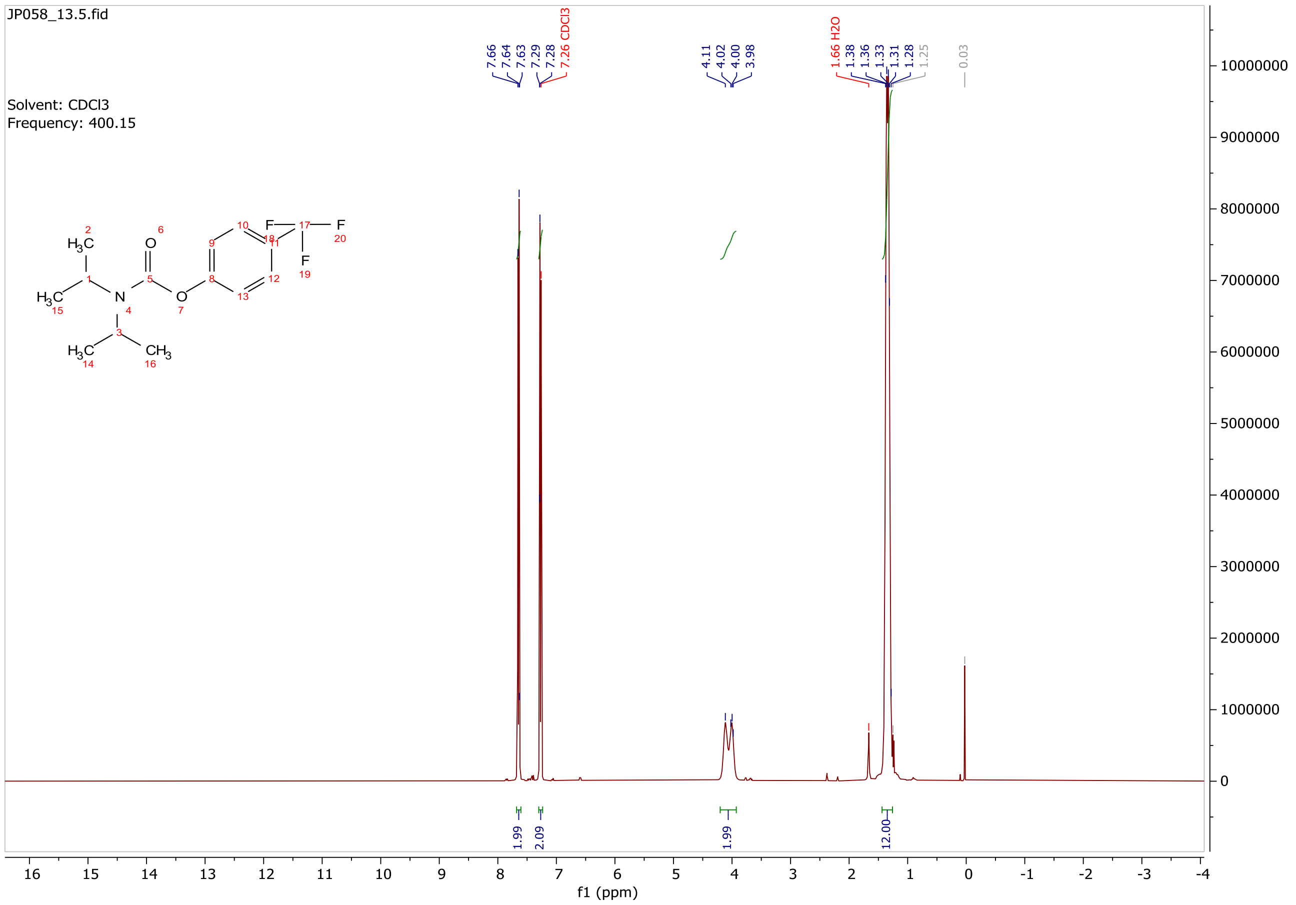

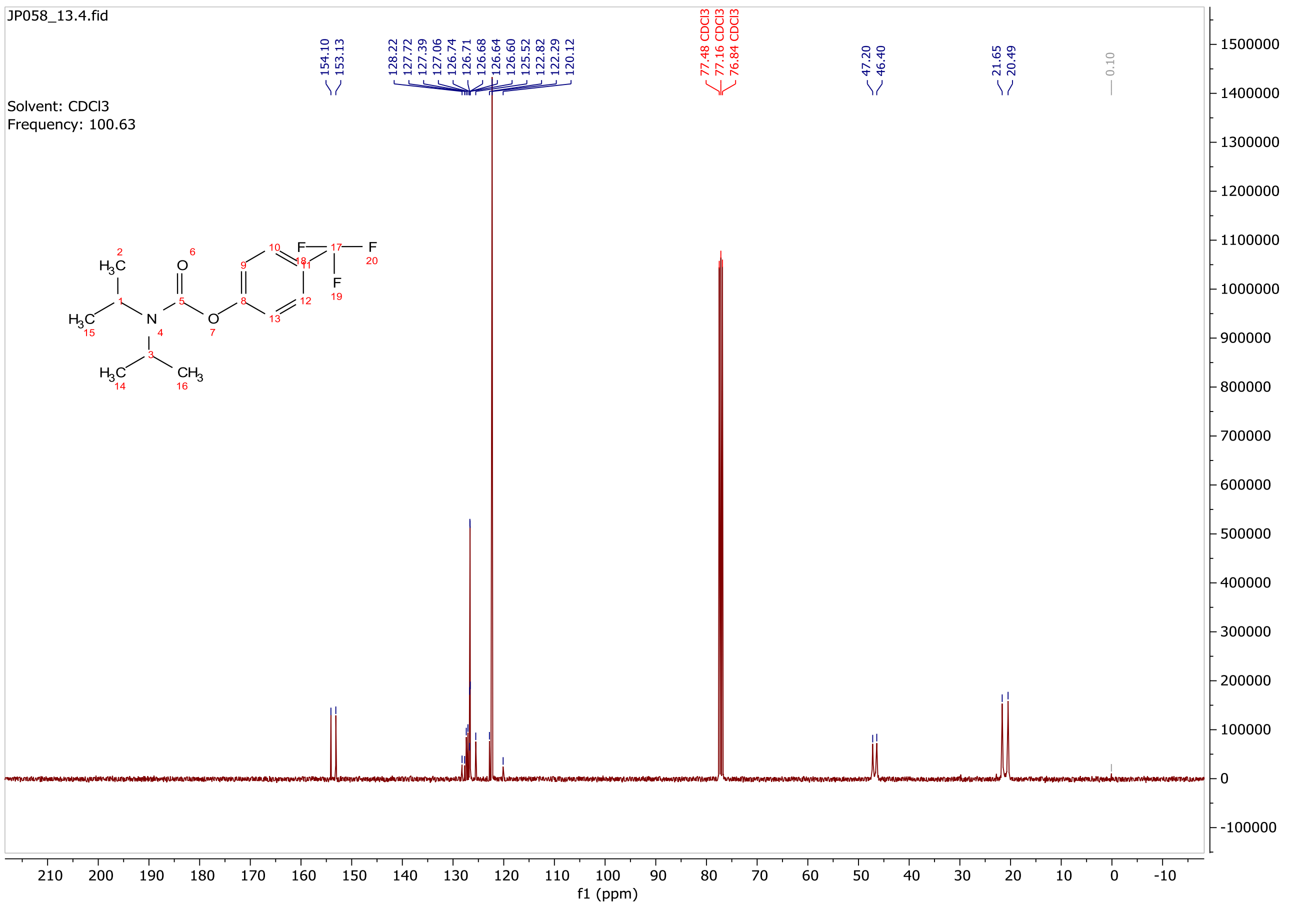

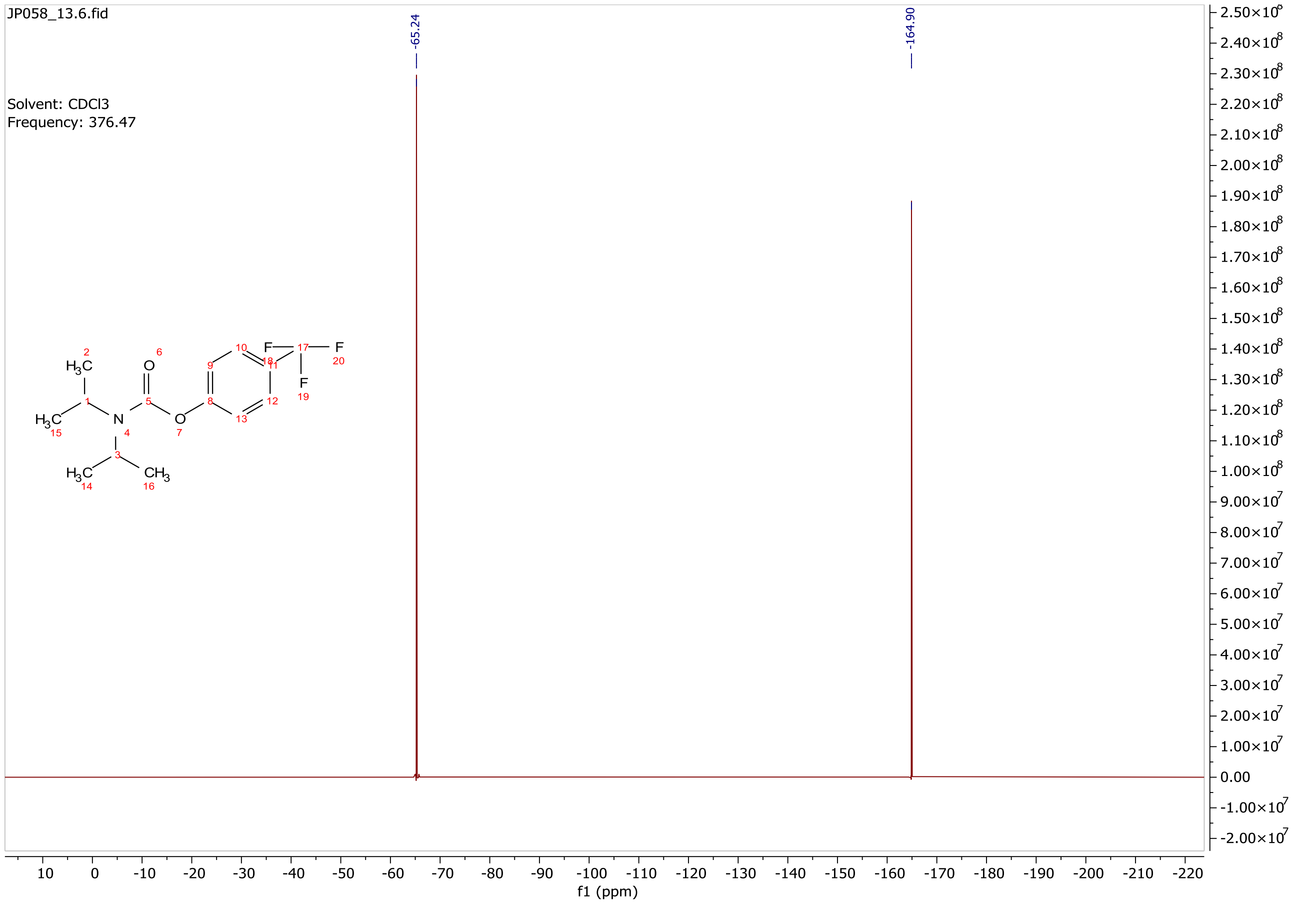

^{1}H, ^{13}C and ^{19}F NMR spectra of 4-(trifluoromethyl)phenyl 3-oxopiperazine-1-carboxylate (14)

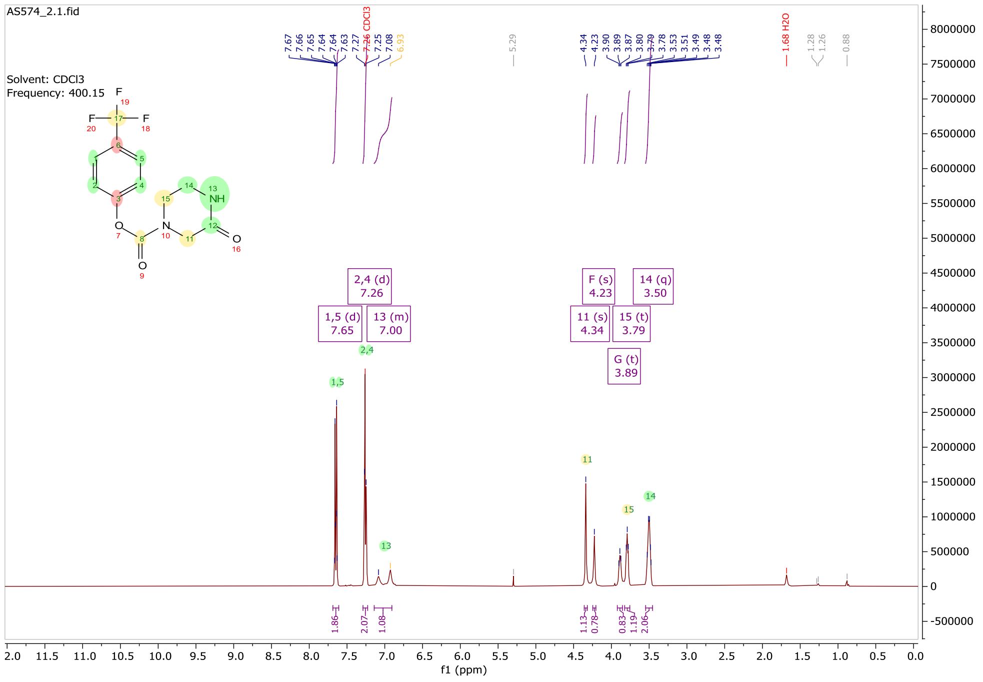

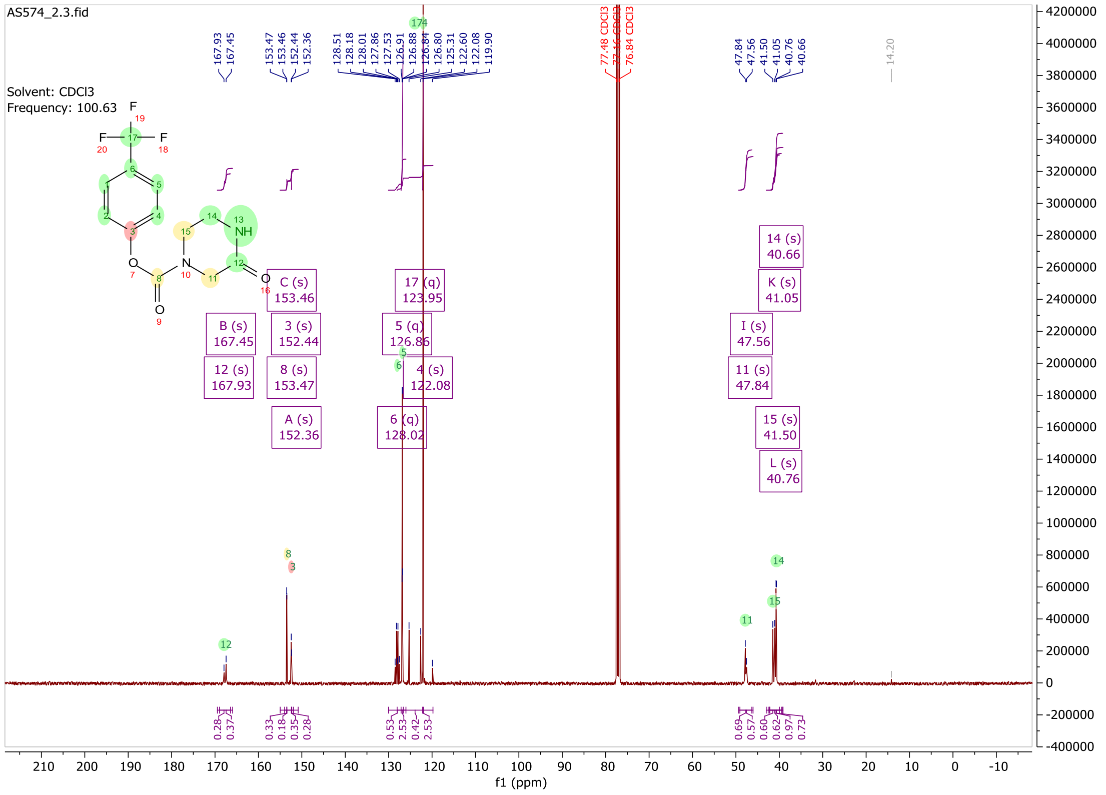
AS574_2.3.fid
Solvent: CDCl3
Frequency: 100.63
f1 (ppm)

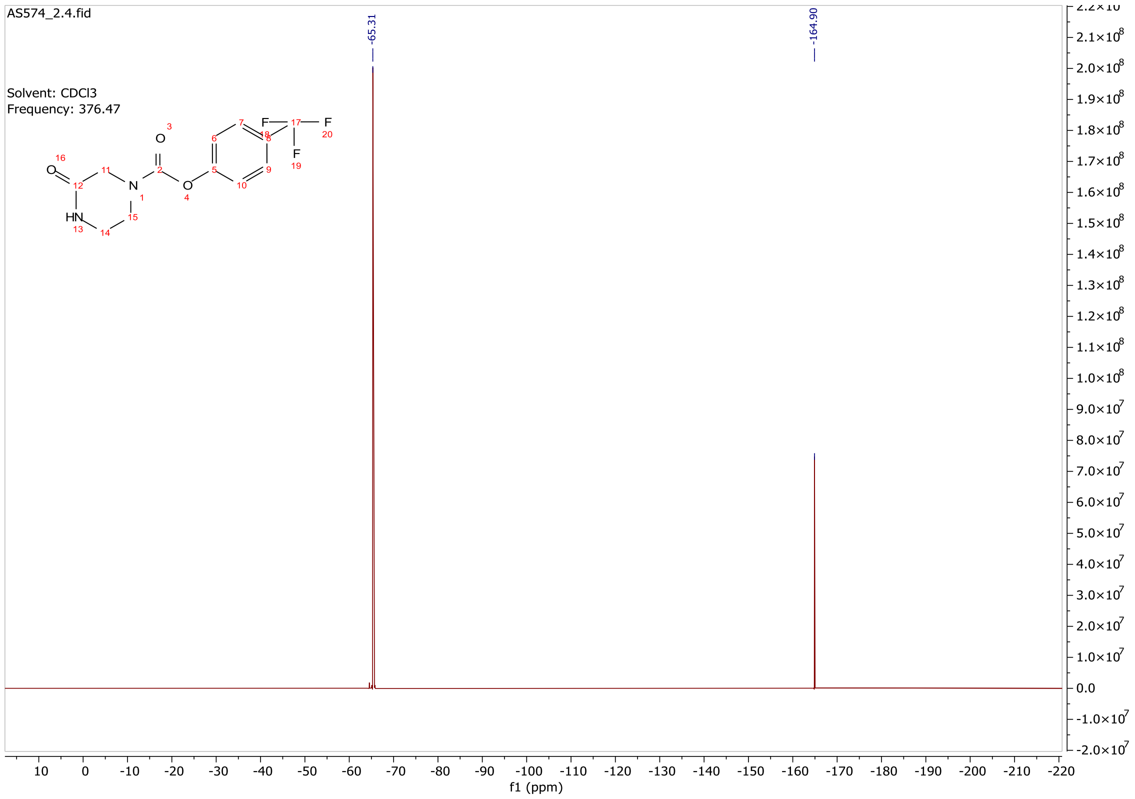
AS574_2.4.fid
Solvent: CDCl3
Frequency: 376.47
-65.31
-164.90
f1 (ppm)

2-methyl 1-(4-(trifluoromethyl)phenyl) pyrrolidine-1,2-dicarboxylate (15)

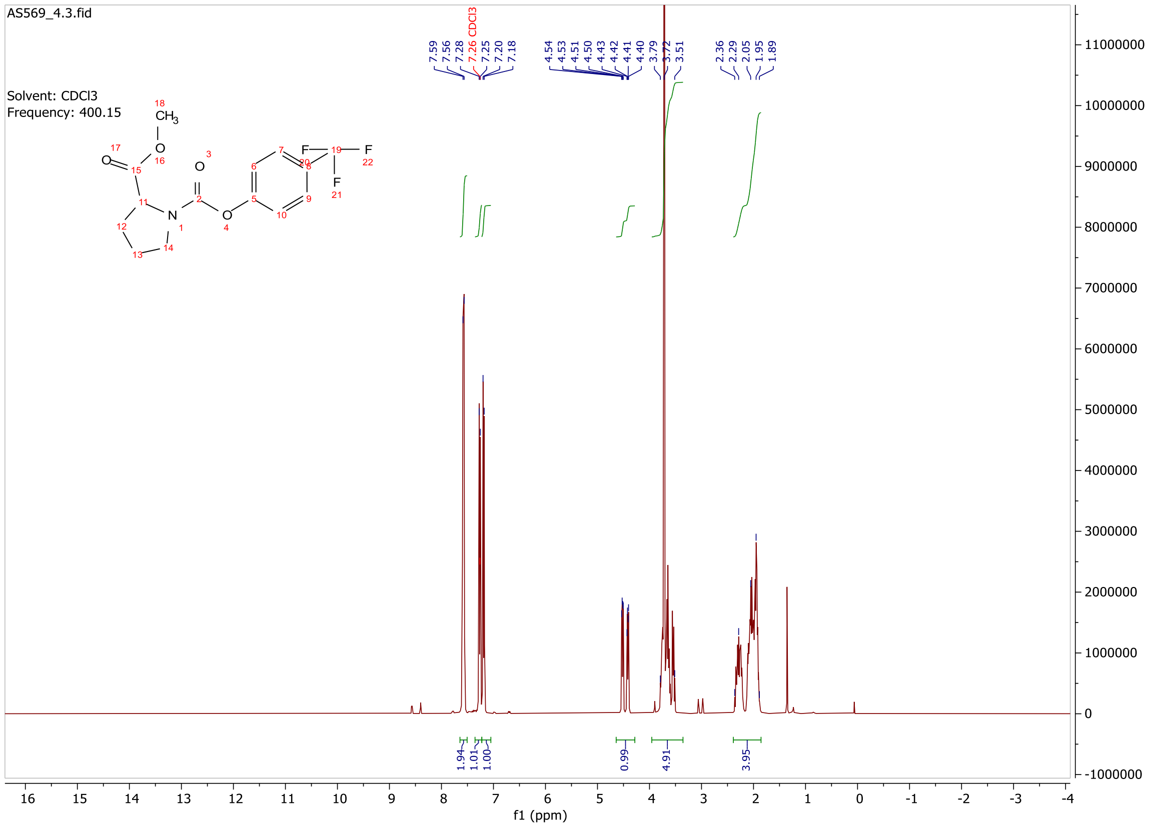

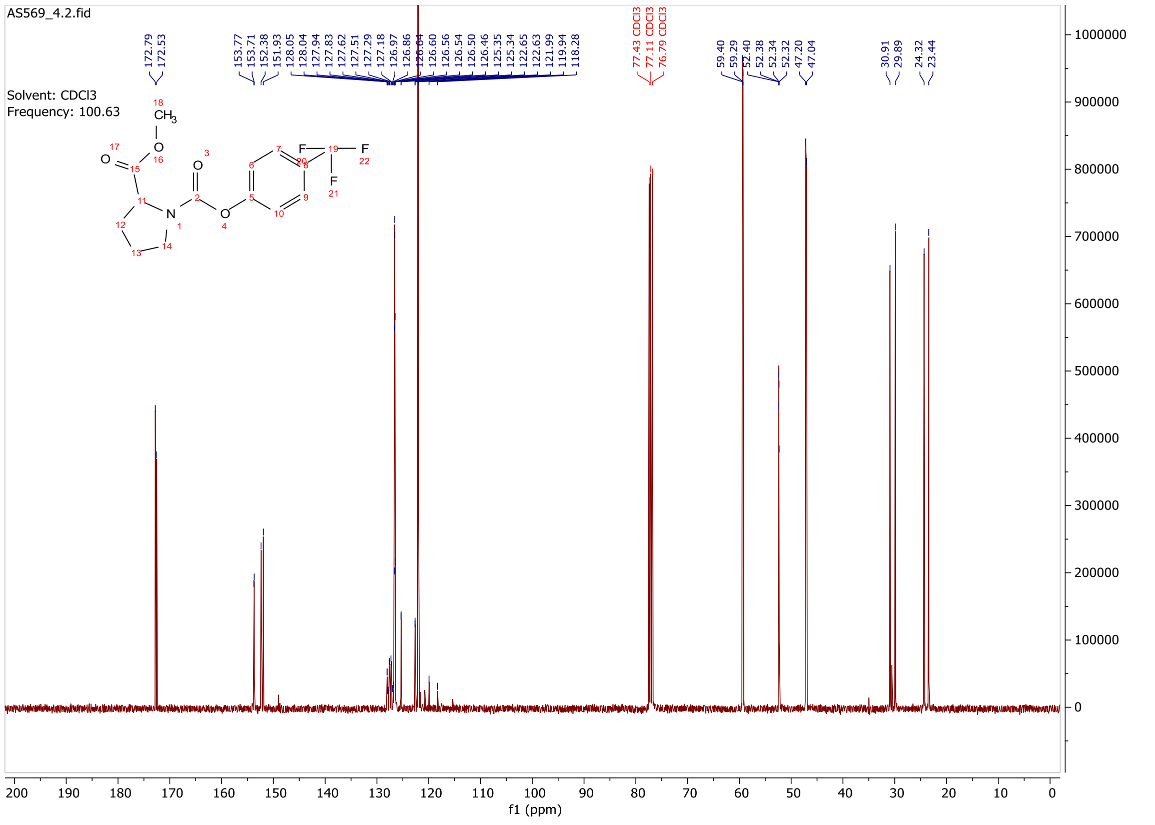

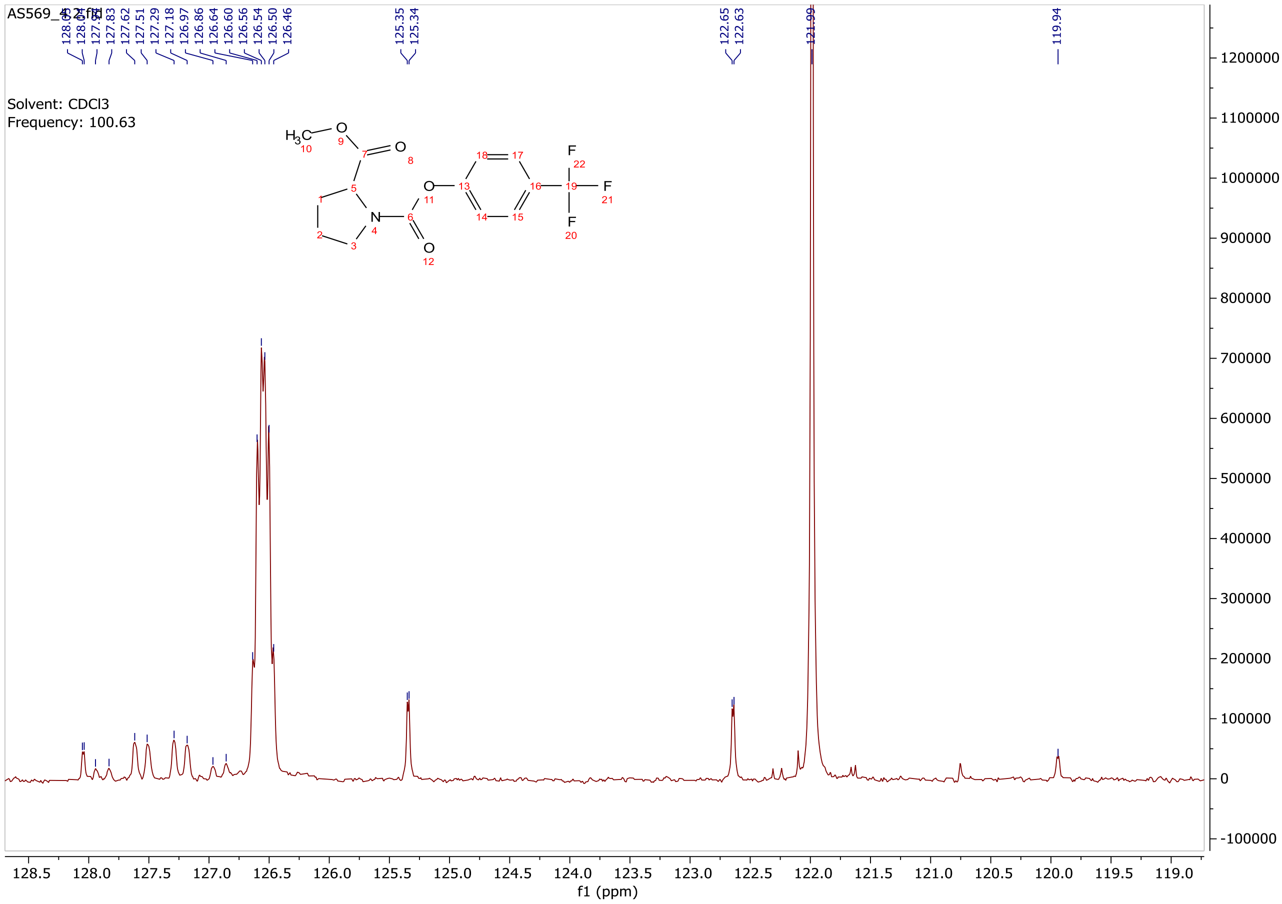

AS569_4.2.fid
127.83
127.62
127.51
127.29
127.18
126.97
126.86
126.64
126.60
126.56
126.54
126.50
126.46
125.35
125.34
122.65
122.63
119.94
Solvent: CDCl3
Frequency: 100.63
f1 (ppm)

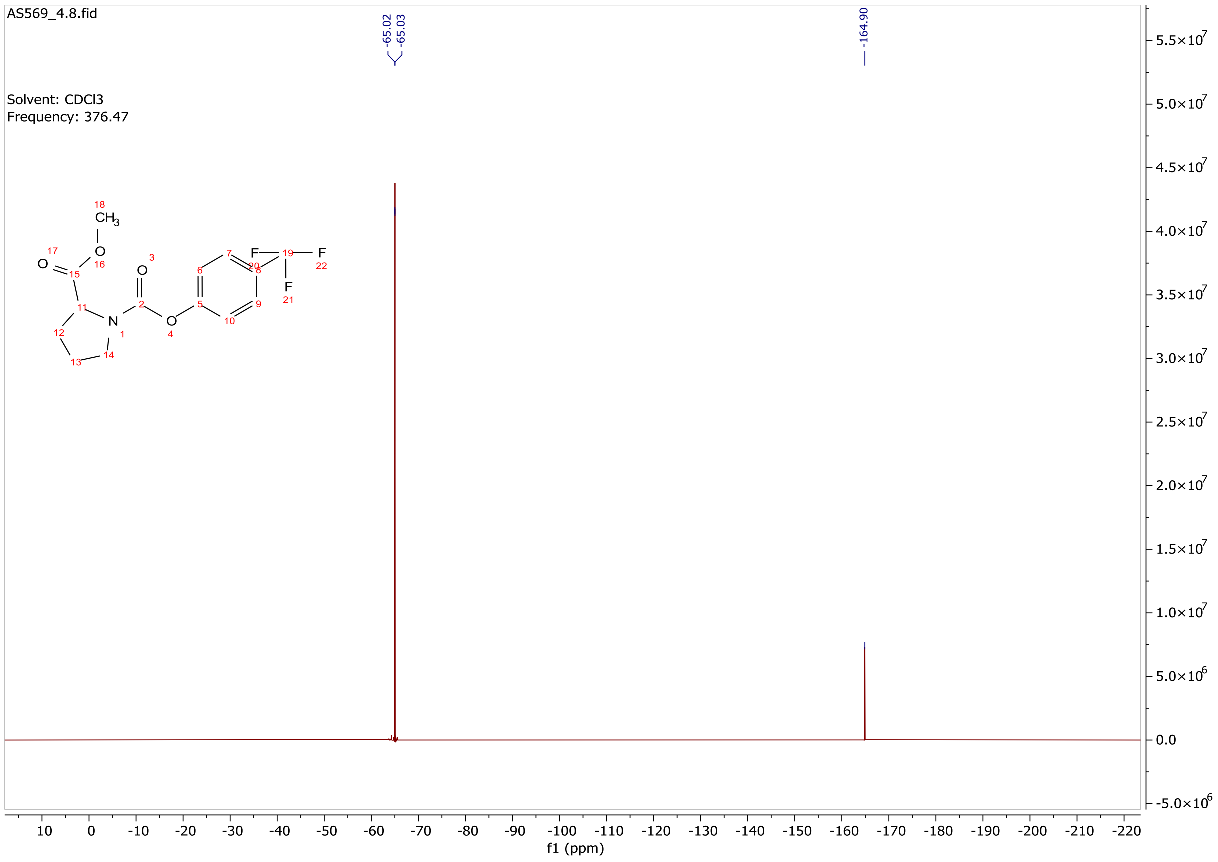

AS569_4.8.fid
-65.02
-65.03
-164.90
Solvent: CDCl3
Frequency: 376.47
f1 (ppm)

^{1}H, ^{13}C and ^{19}F NMR spectra of 4-(trifluoromethyl)phenyl 4-benzylpiperazine-1-carboxylate (16)

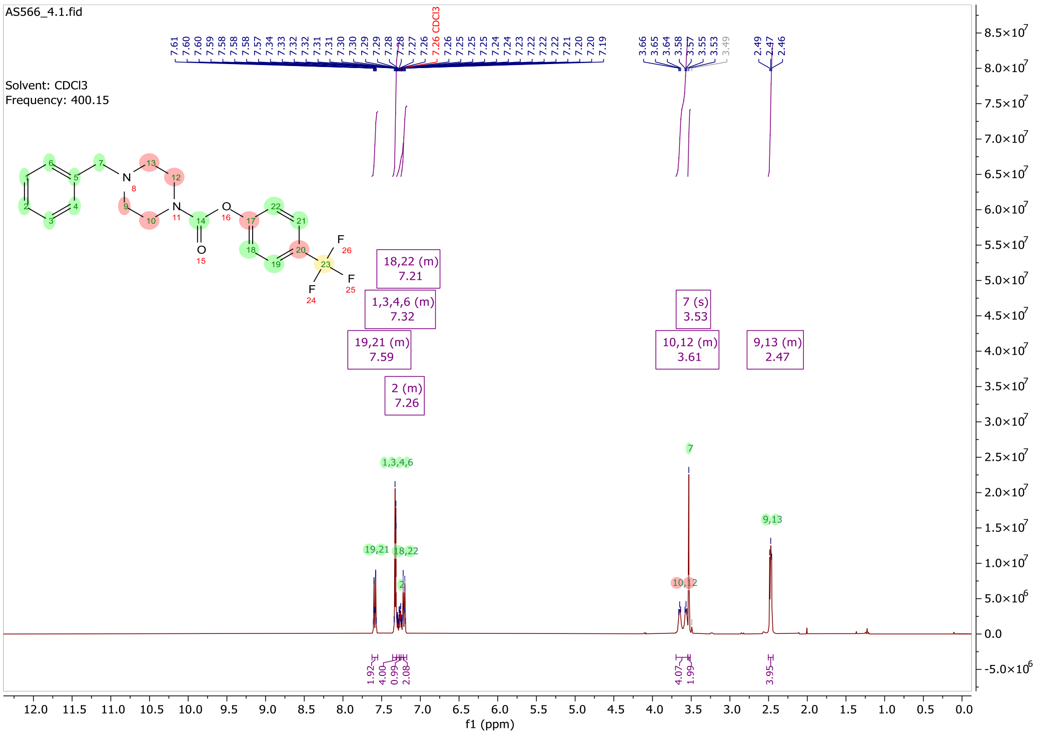

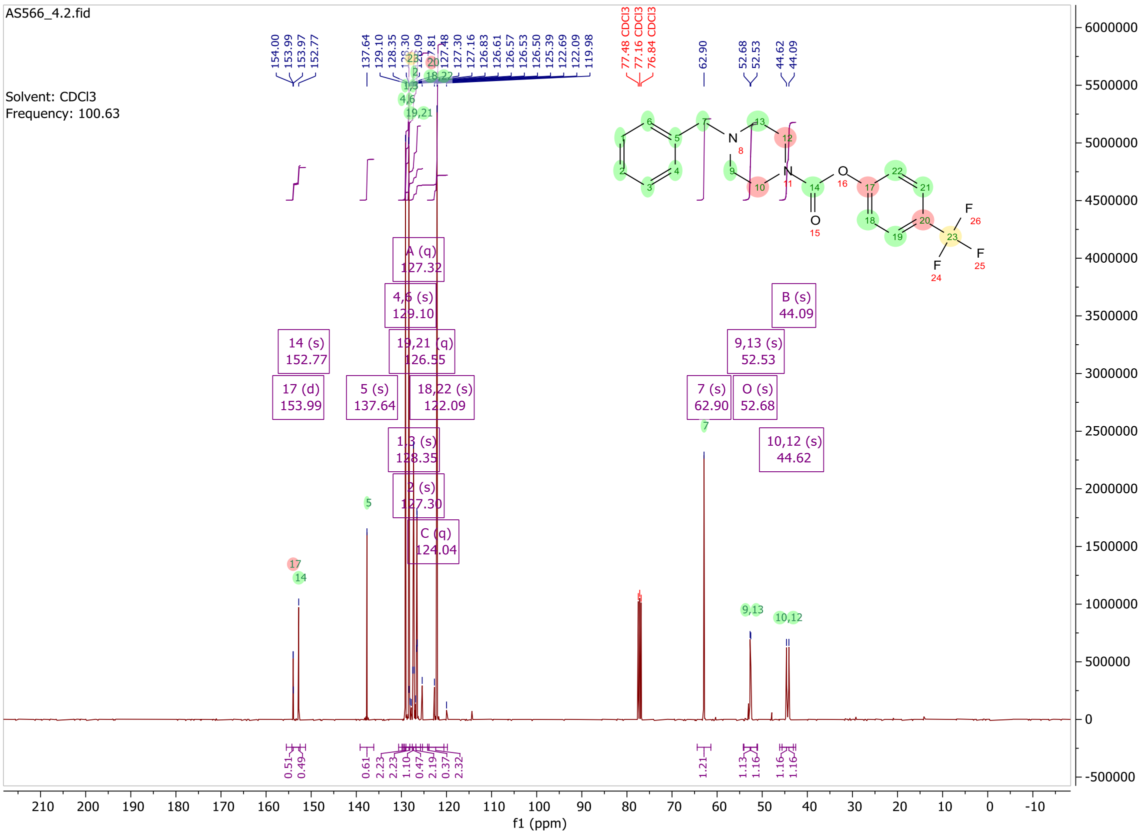

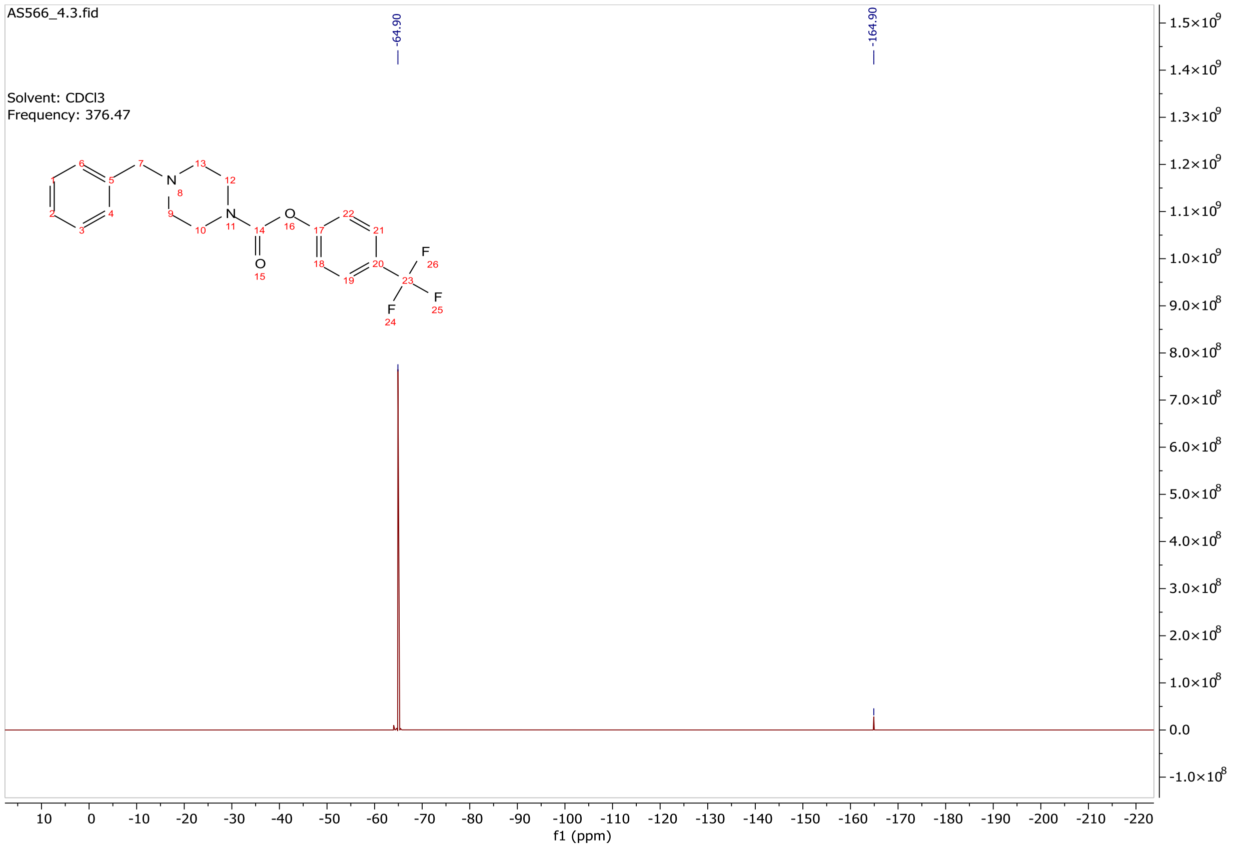
AS566_4.3.fid
Solvent: CDCl3
Frequency: 376.47
-64.90
-164.90
f1 (ppm)

4-(trifluoromethyl)phenyl 4-(pyridin-2-yl)piperazine-1-carboxylate (17)

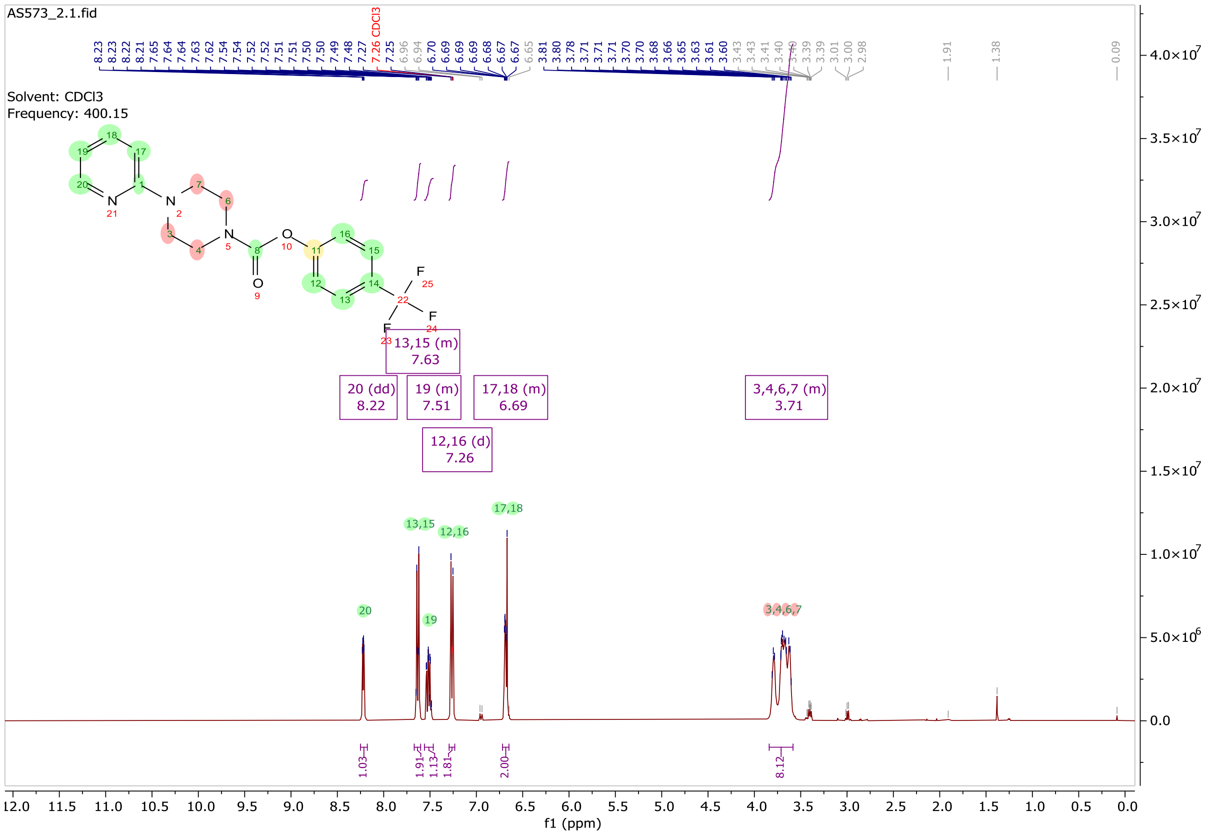

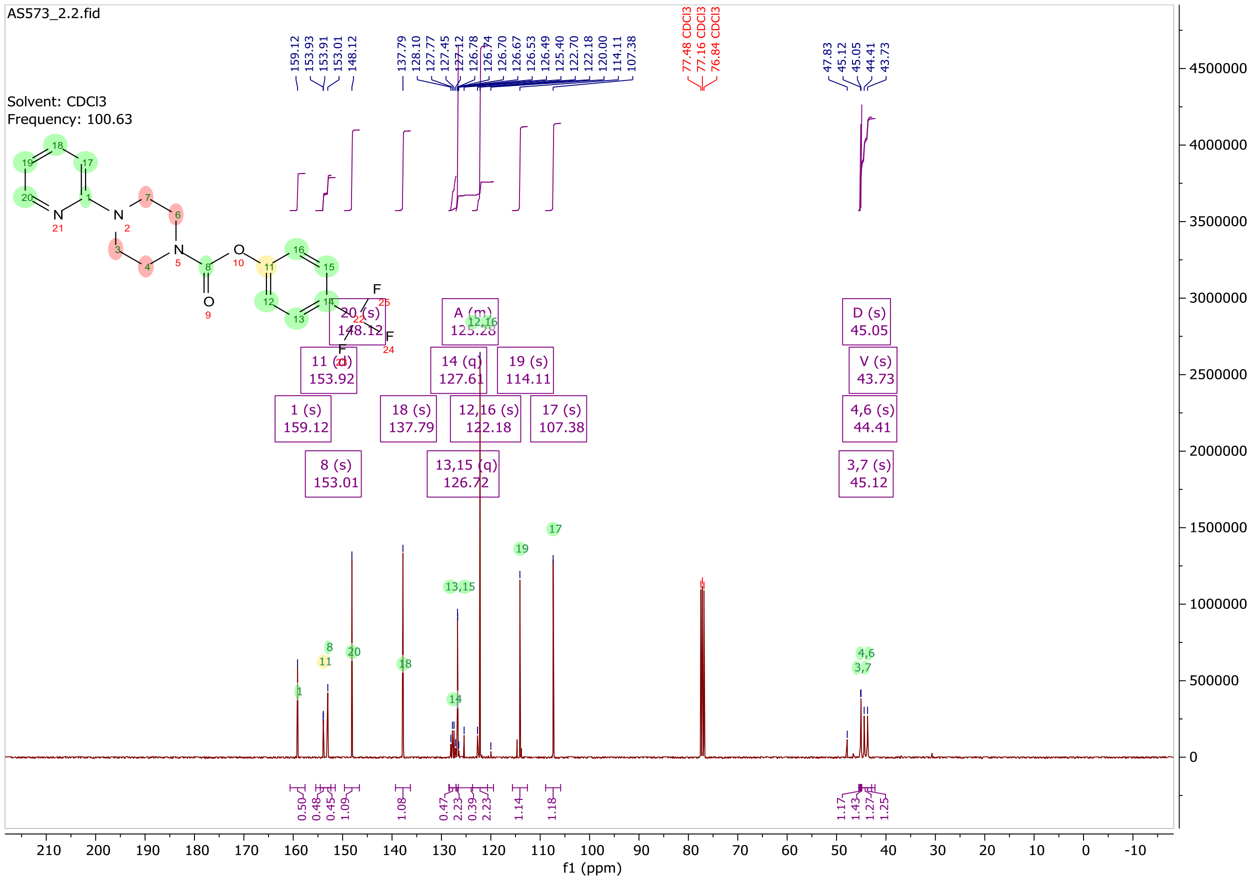

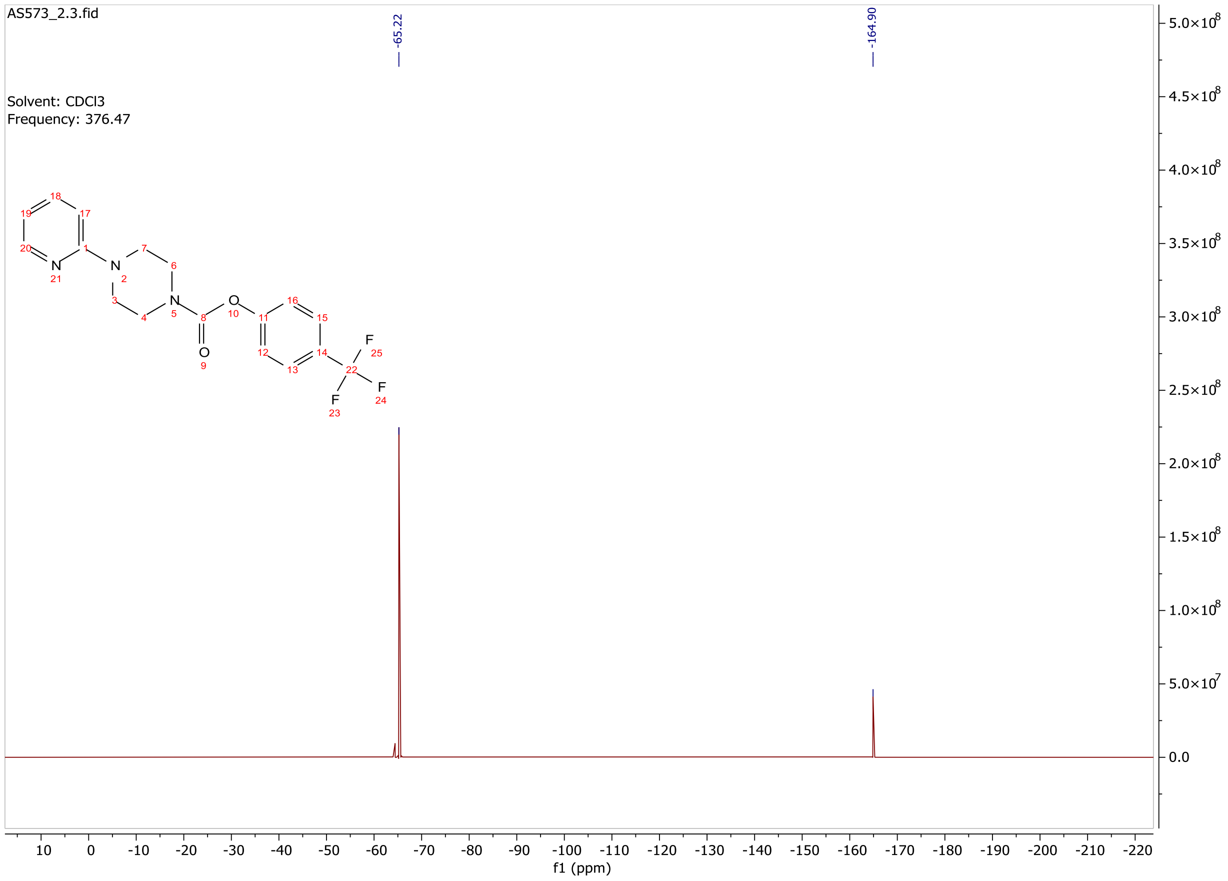
AS573_2.3.fid
Solvent: CDCl3
Frequency: 376.47
-65.22
-164.90
f1 (ppm)

^{1}H, ^{13}C and ^{19}F NMR spectra of bis(4-(trifluoromethyl)phenyl) piperazine-1,4-dicarboxylate (18)

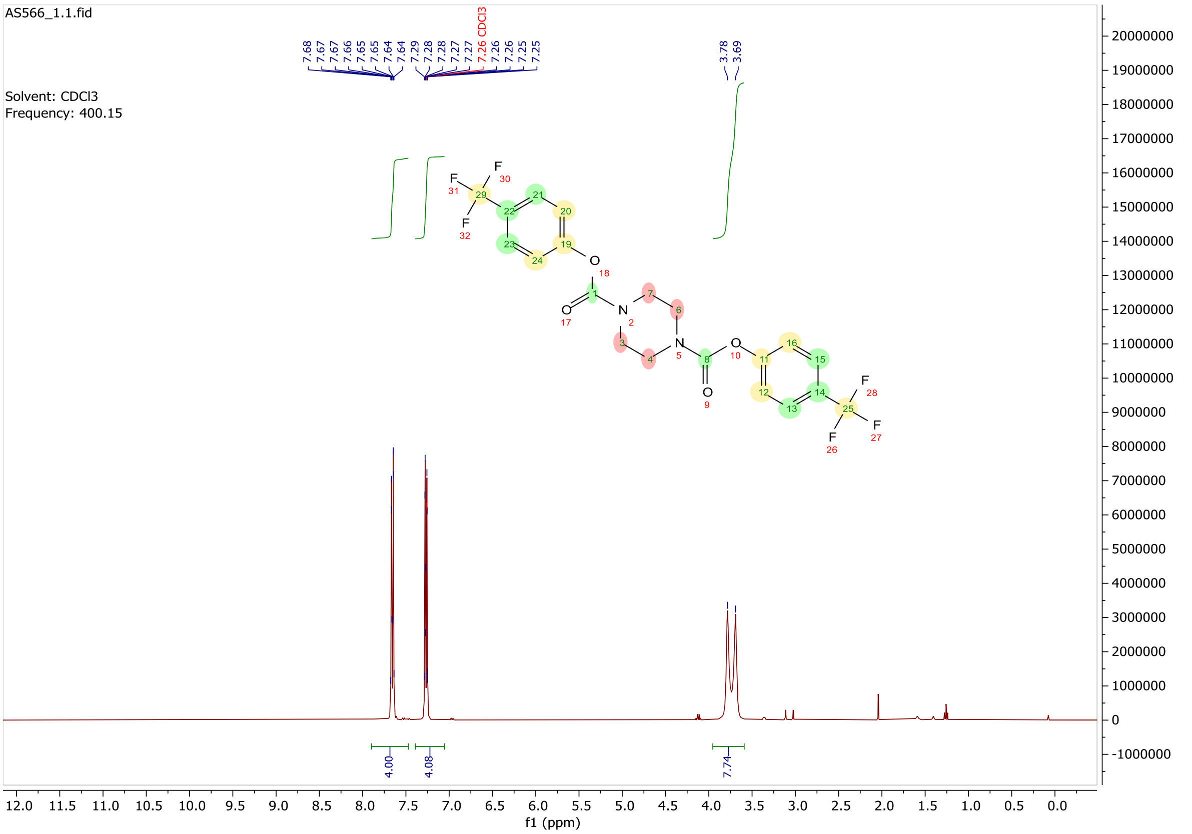

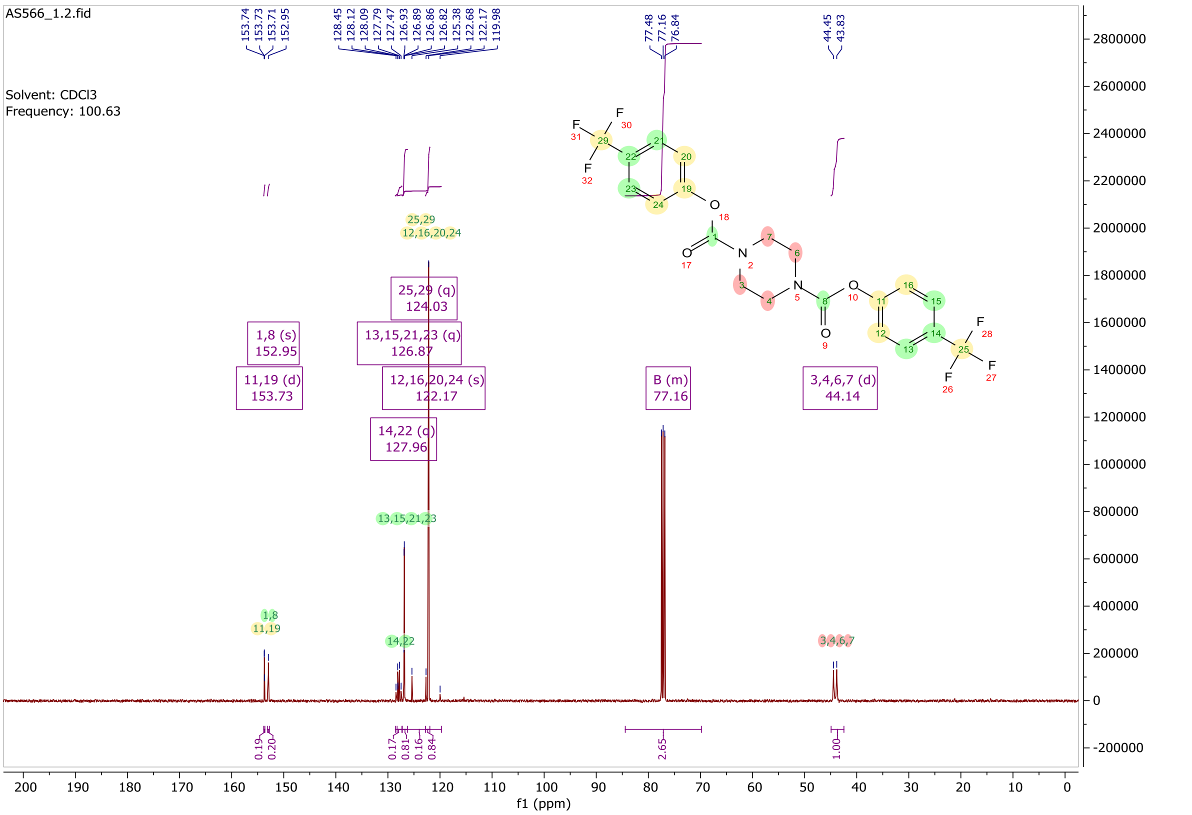

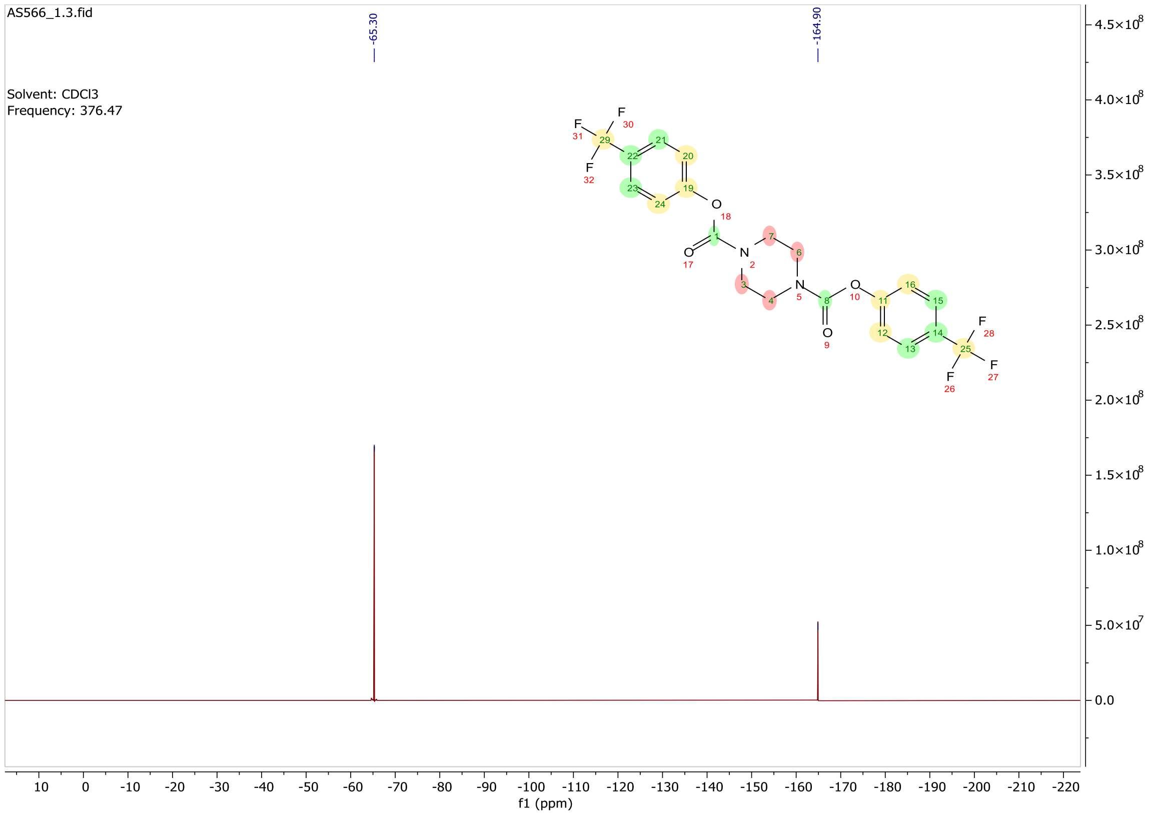
AS566_1.3.fid
-65.30
-164.90
Solvent: CDCl3
Frequency: 376.47
f1 (ppm)

^{1}H, ^{13}C and ^{19}F NMR spectra of 4-(trifluoromethyl)phenyl 4-(8-chloro-5,6-dihydro-11H-benzo[5,6]cyclohepta[1,2-b]pyridin-11-ylidene)piperidine-1-carboxylate (19)

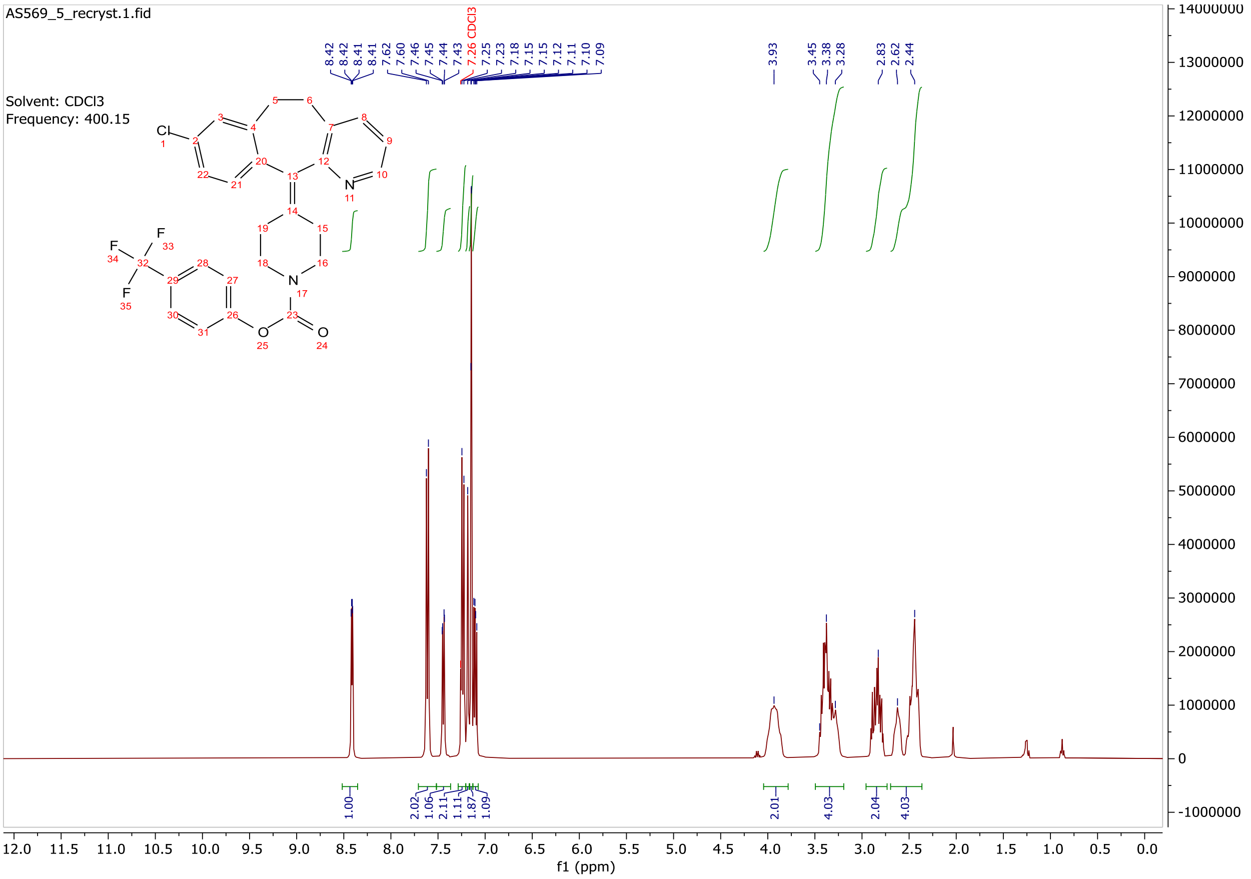

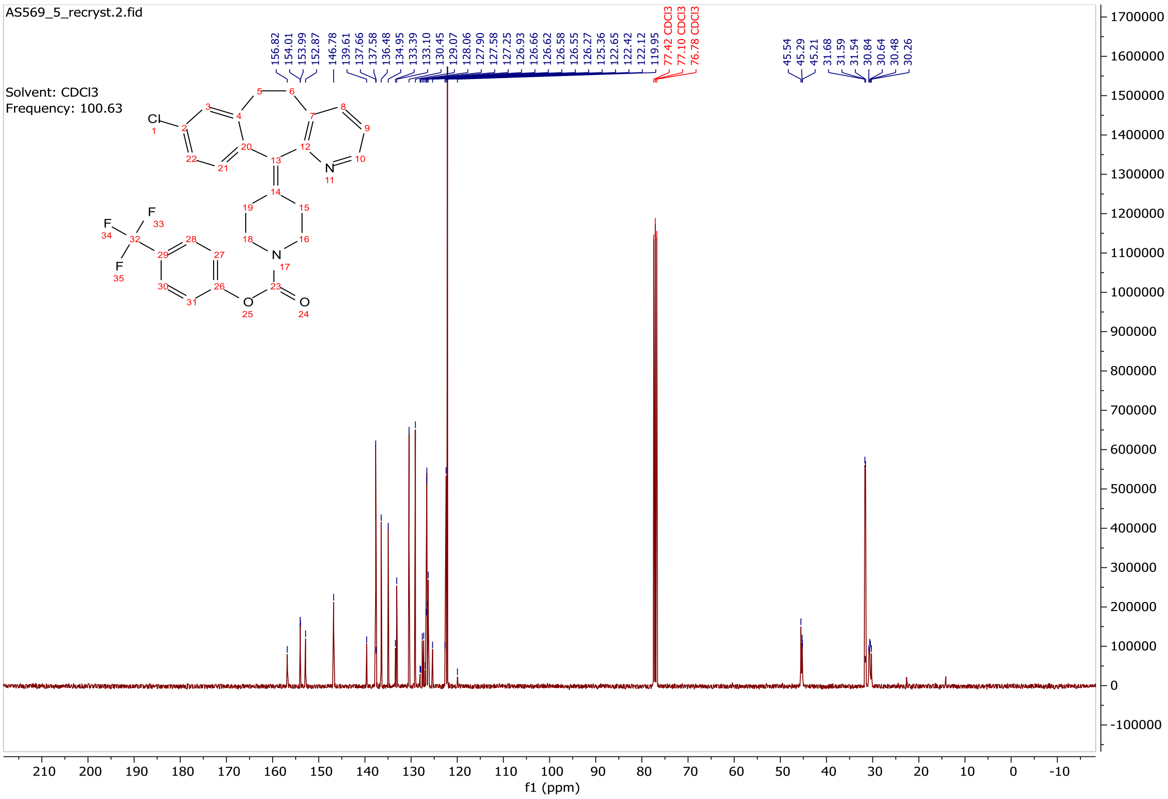

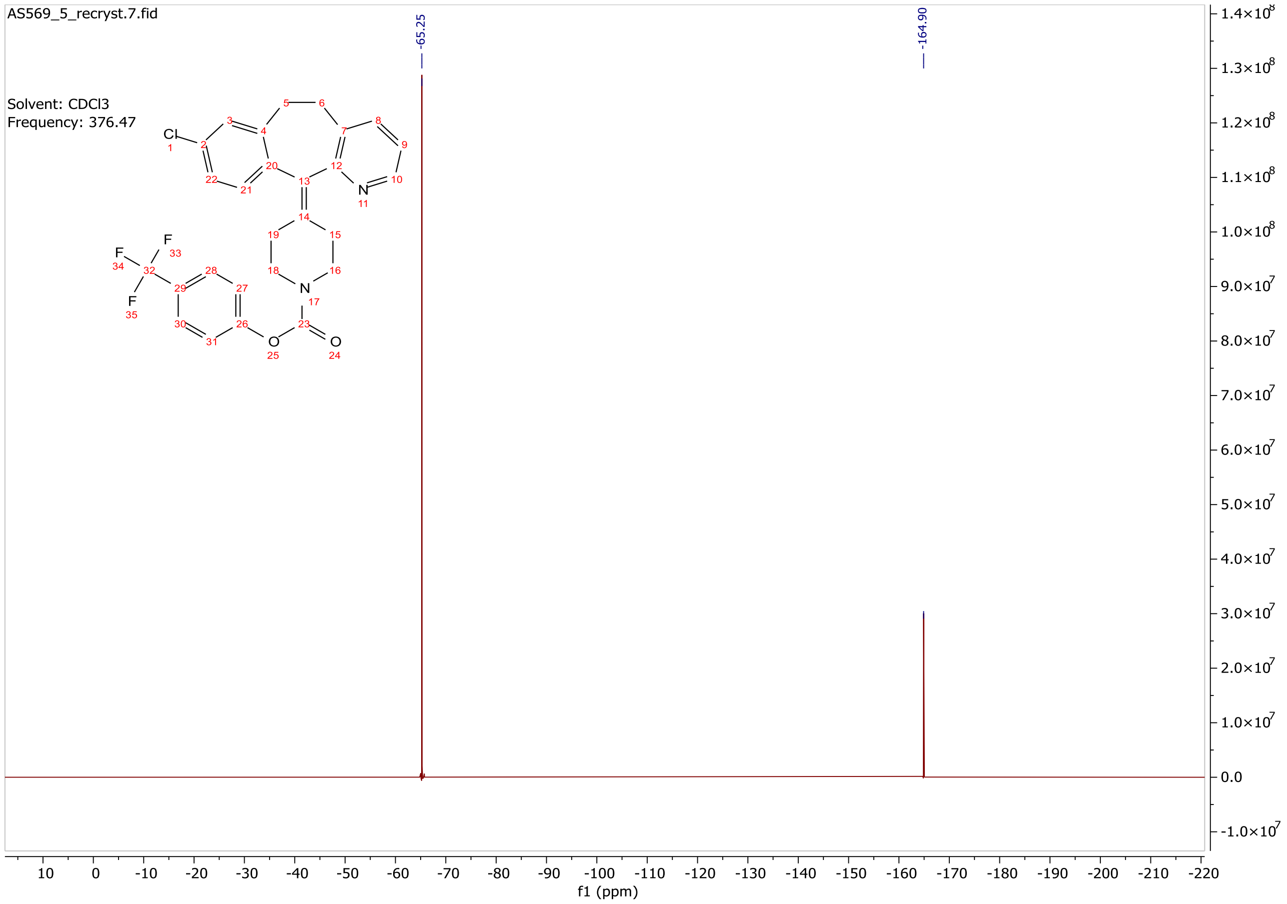
AS569_5_recryst.7.fid
Solvent: CDCl3
Frequency: 376.47
-65.25
-164.90
f1 (ppm)

^{1}H and ^{13}C NMR spectra of 4-cyanophenyl morpholine-4-carboxylate (20)

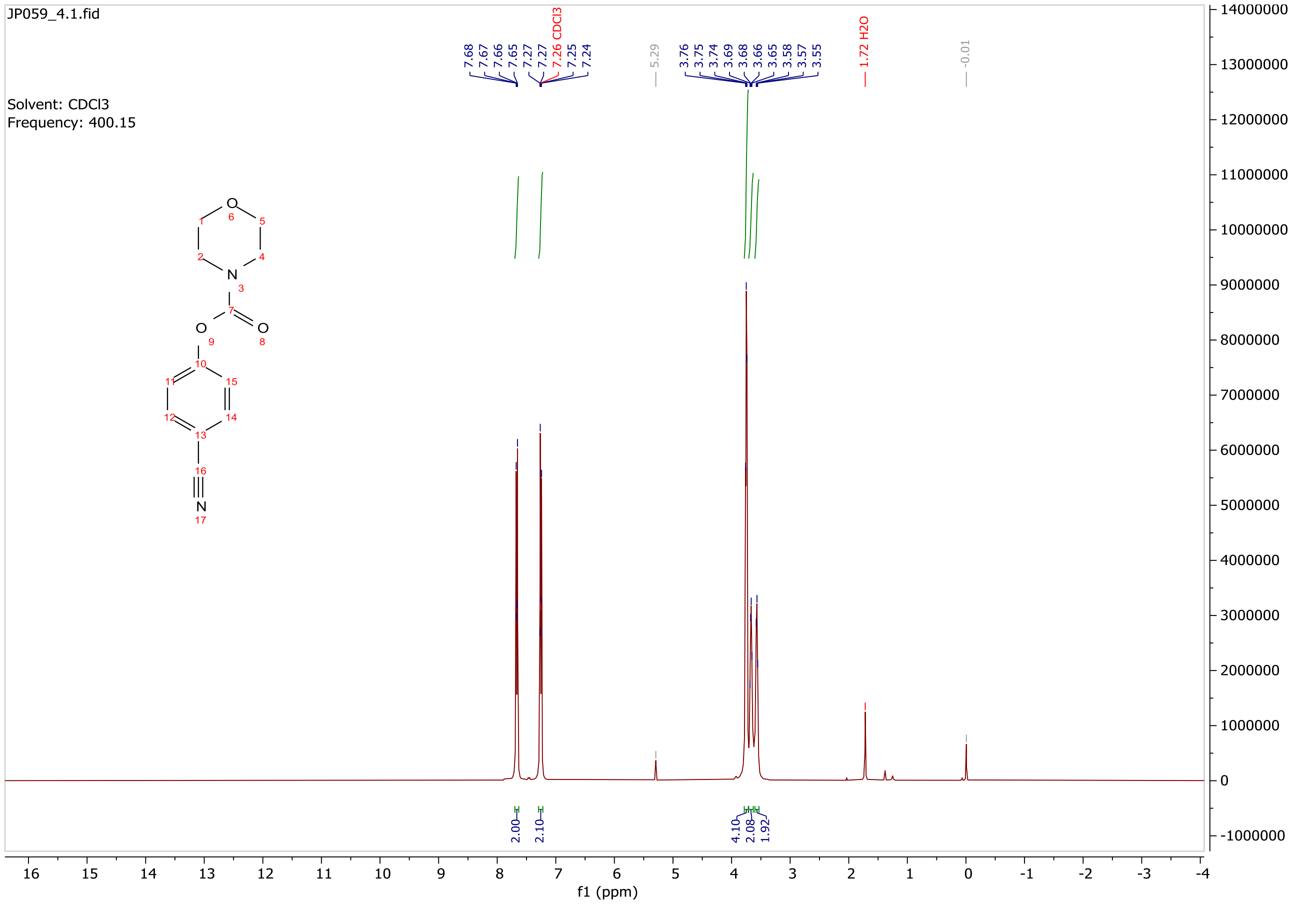

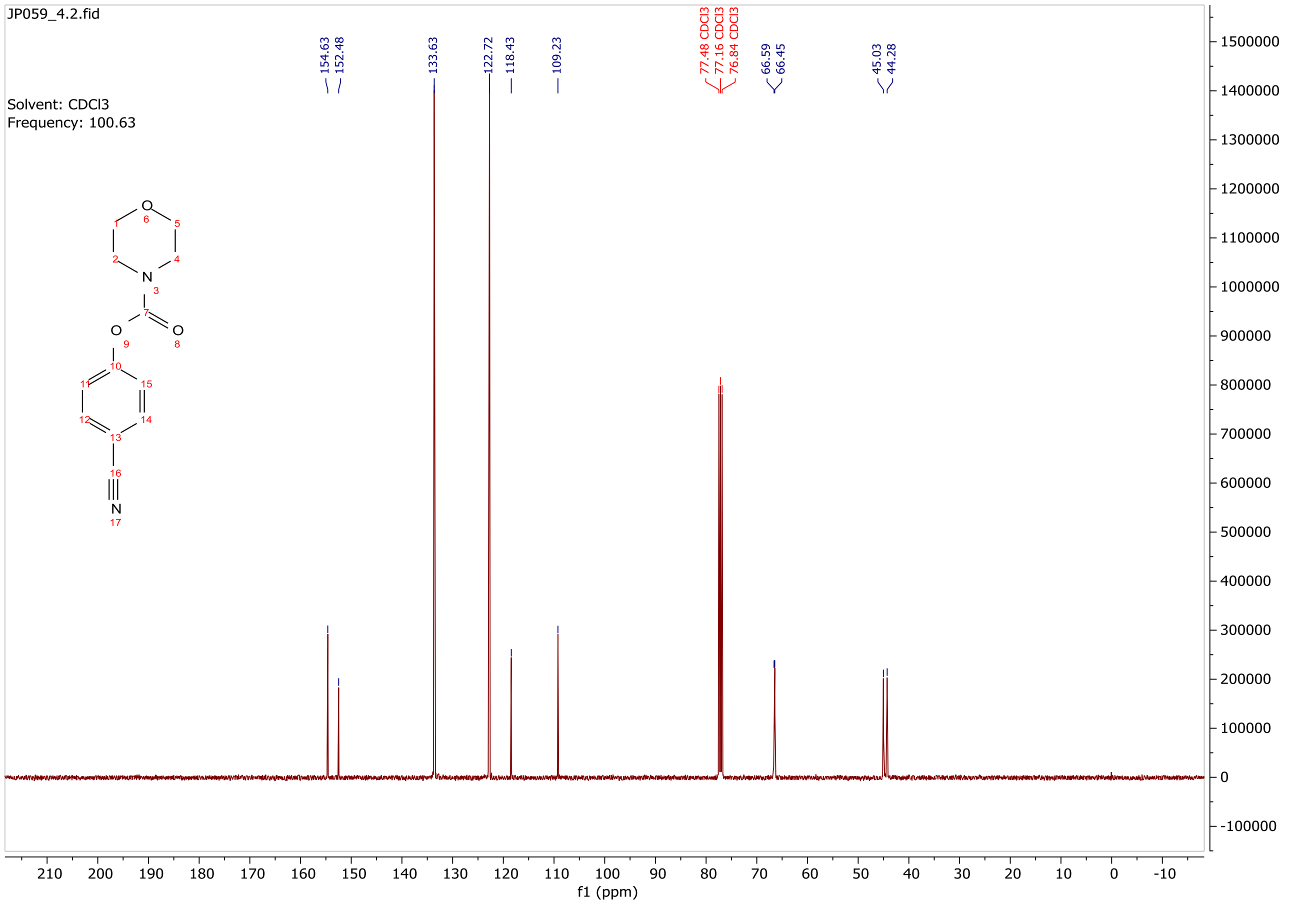

^{1}H and ^{13}C NMR spectra of 4-formylphenyl morpholine-4-carboxylate (21)

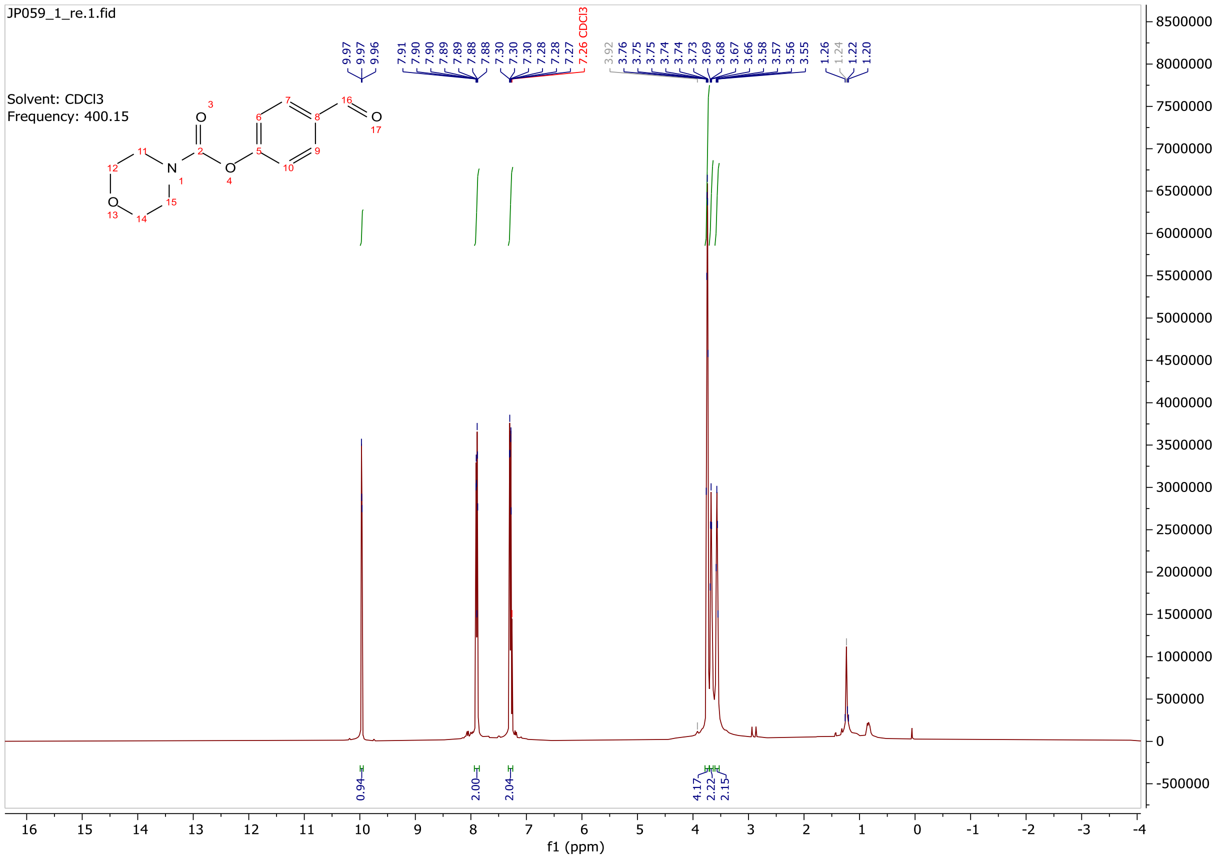

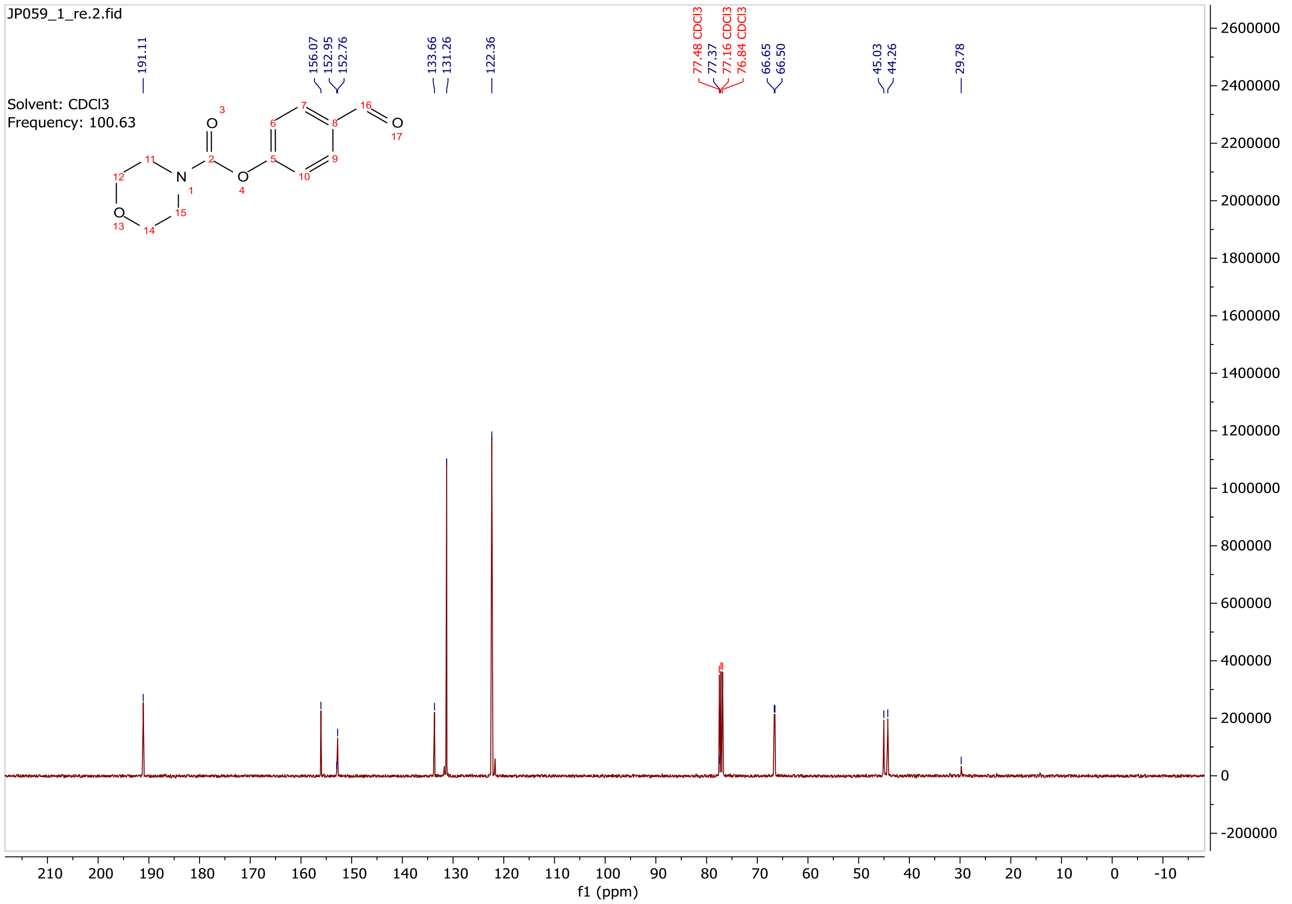

^{1}H and ^{13}C NMR spectra of 4-(methoxycarbonyl)phenyl morpholine-4-carboxylate (22)

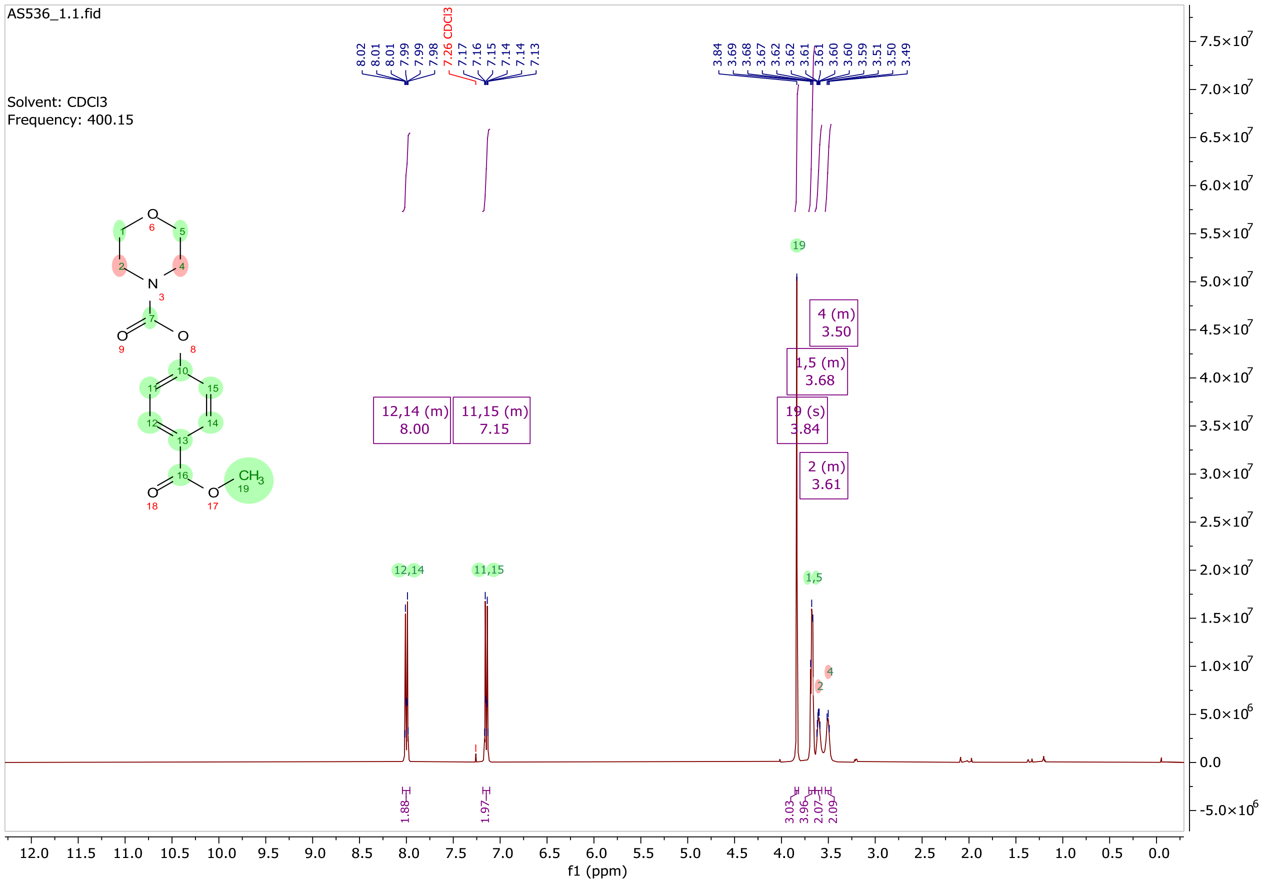

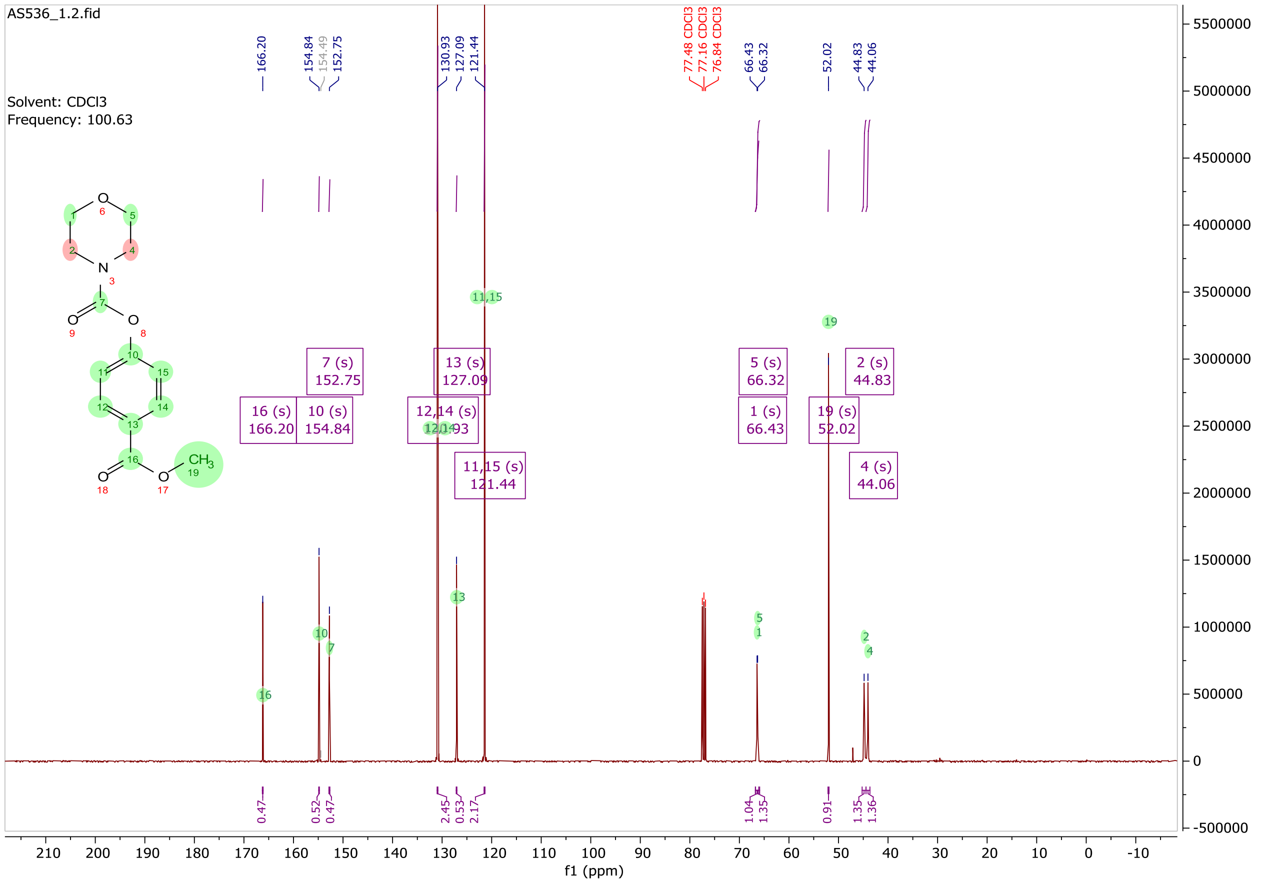

^{1}H and ^{13}C NMR and IR spectra of 4-chlorophenyl morpholine-4-carboxylate (23)

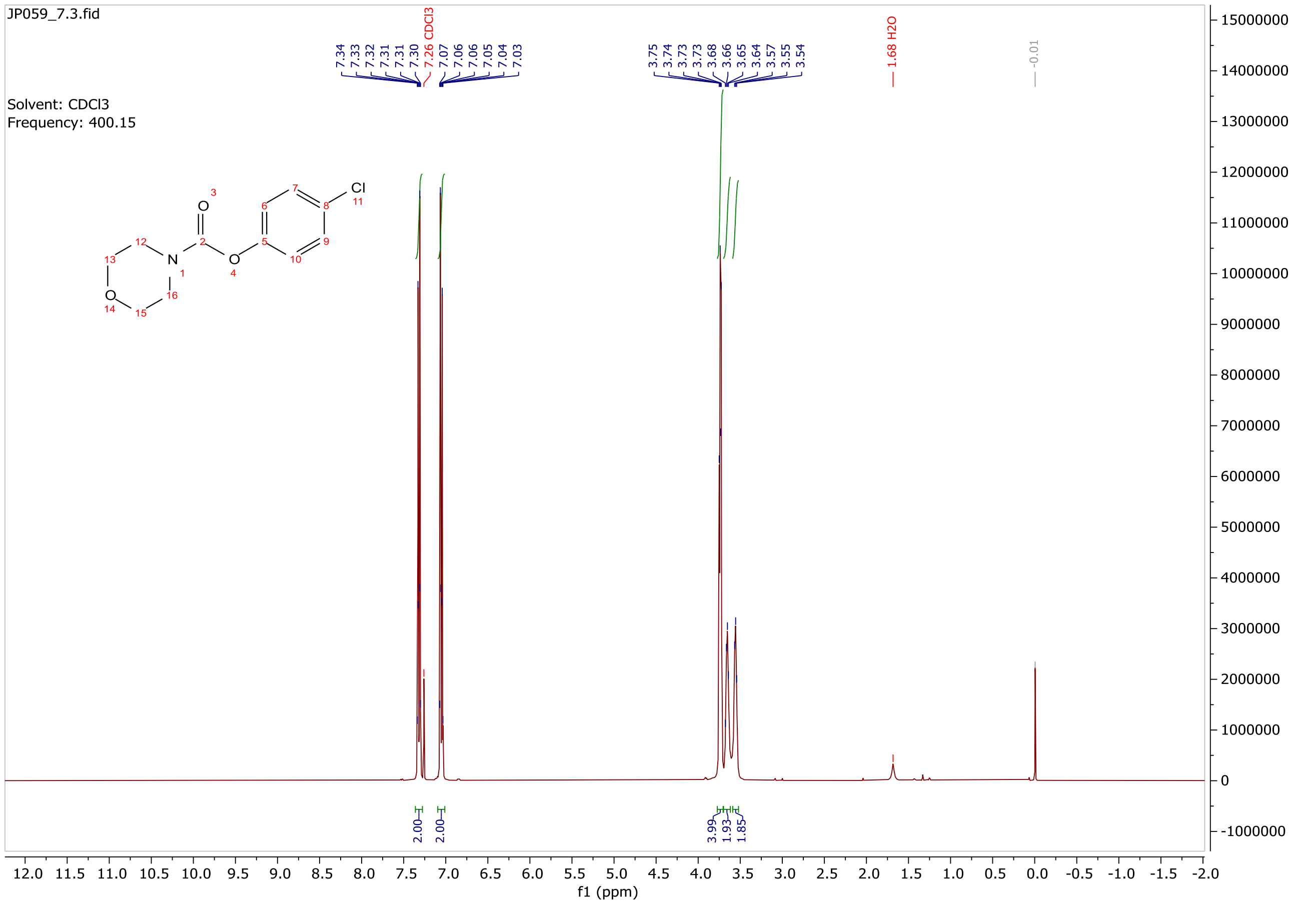

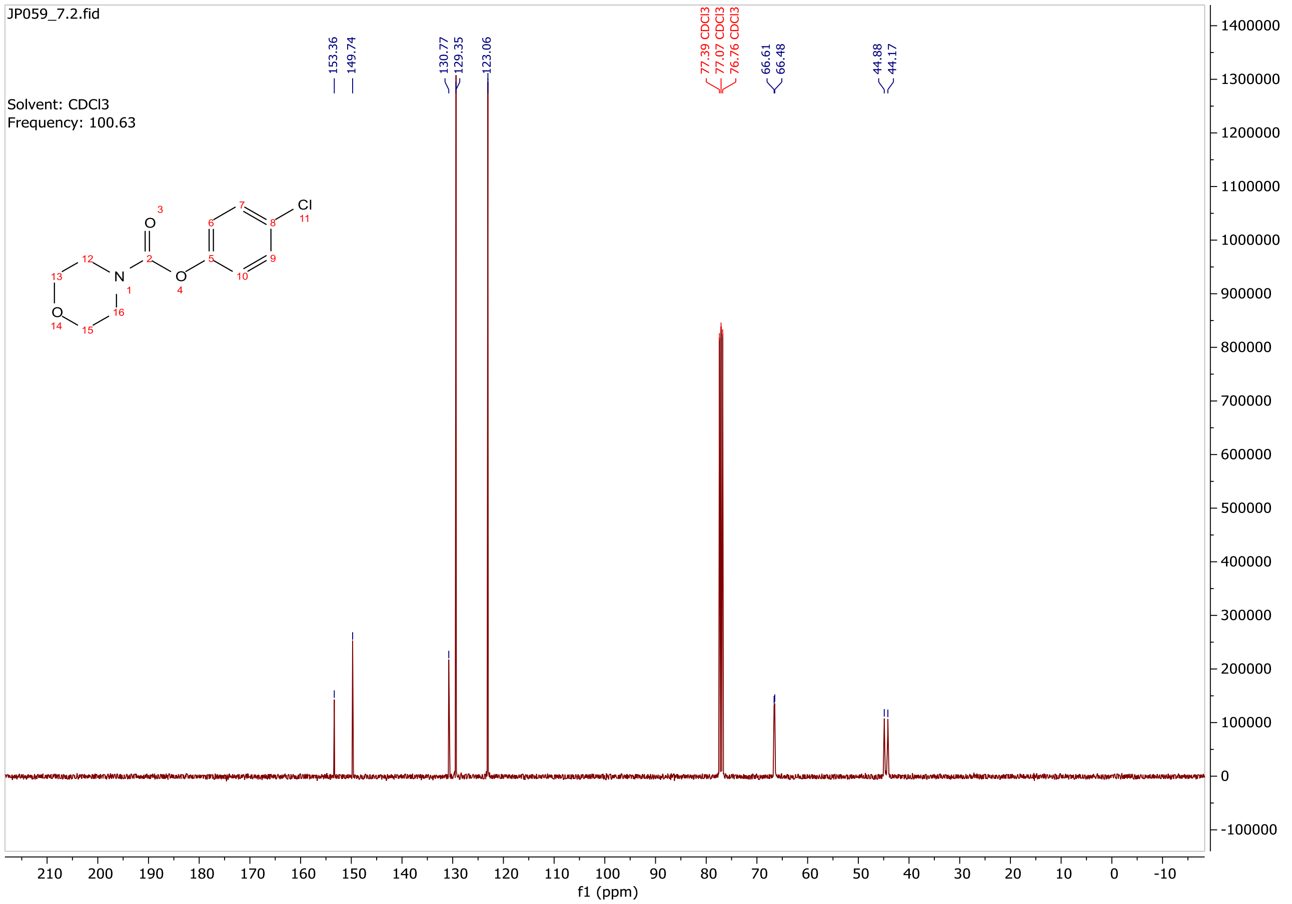

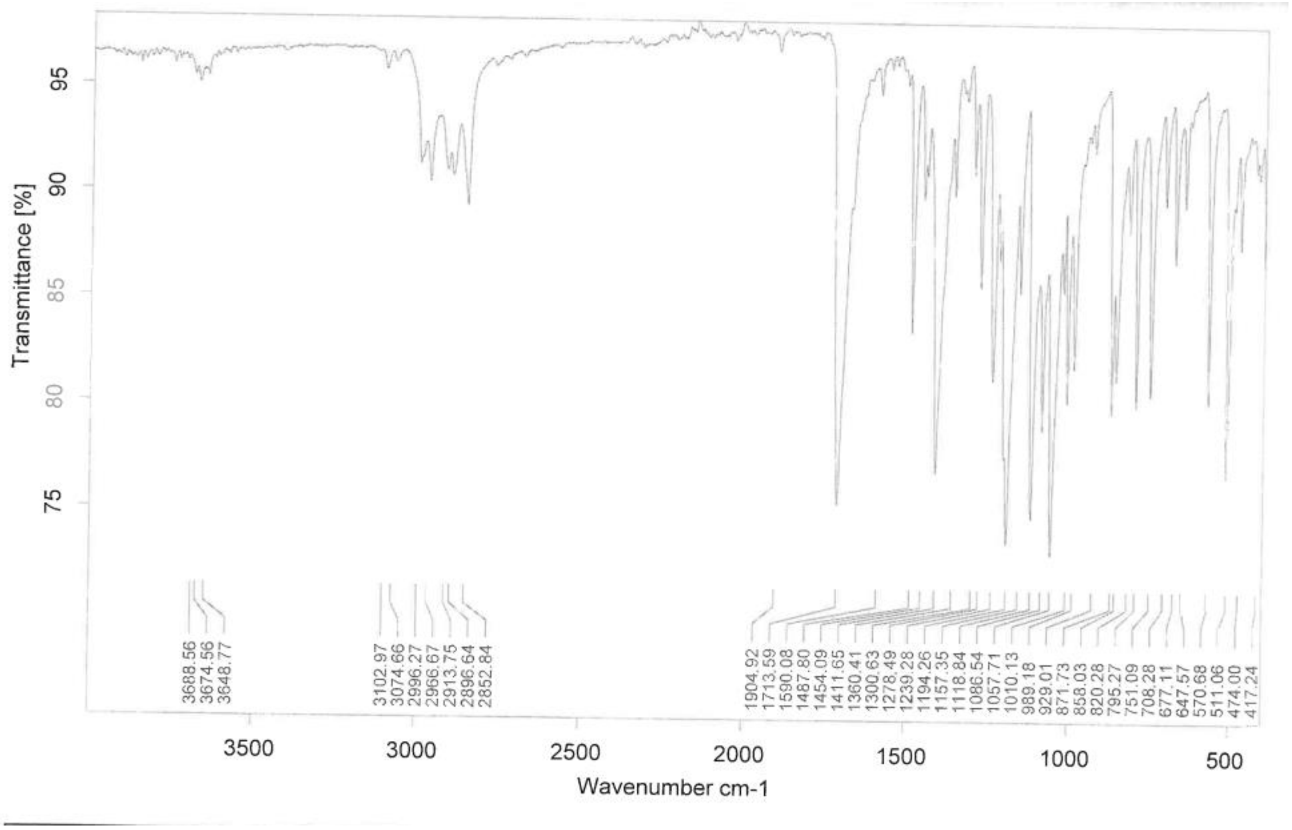
Transmittance [%]
95
90
85
80
75
3688.56
3674.56
3648.77
3102.97
3074.66
2996.27
2966.67
2913.75
2896.64
2852.84
1904.92
1713.59
1590.08
1487.80
1454.09
1411.65
1360.41
1300.63
1278.49
1239.28
1194.26
1157.35
1118.84
1086.54
1057.71
1010.13
989.18
929.01
871.73
858.03
820.28
795.27
751.09
708.28
677.11
647.57
570.68
511.06
474.00
417.24
3500
3000
2500
2000
1500
1000
500
Wavenumber cm-1

^{1}H and ^{13}C NMR spectra of 4-(4,4,5,5-tetramethyl-1,3,2-dioxaborolan-2-yl)phenyl morpholine-4-carboxylate (24)

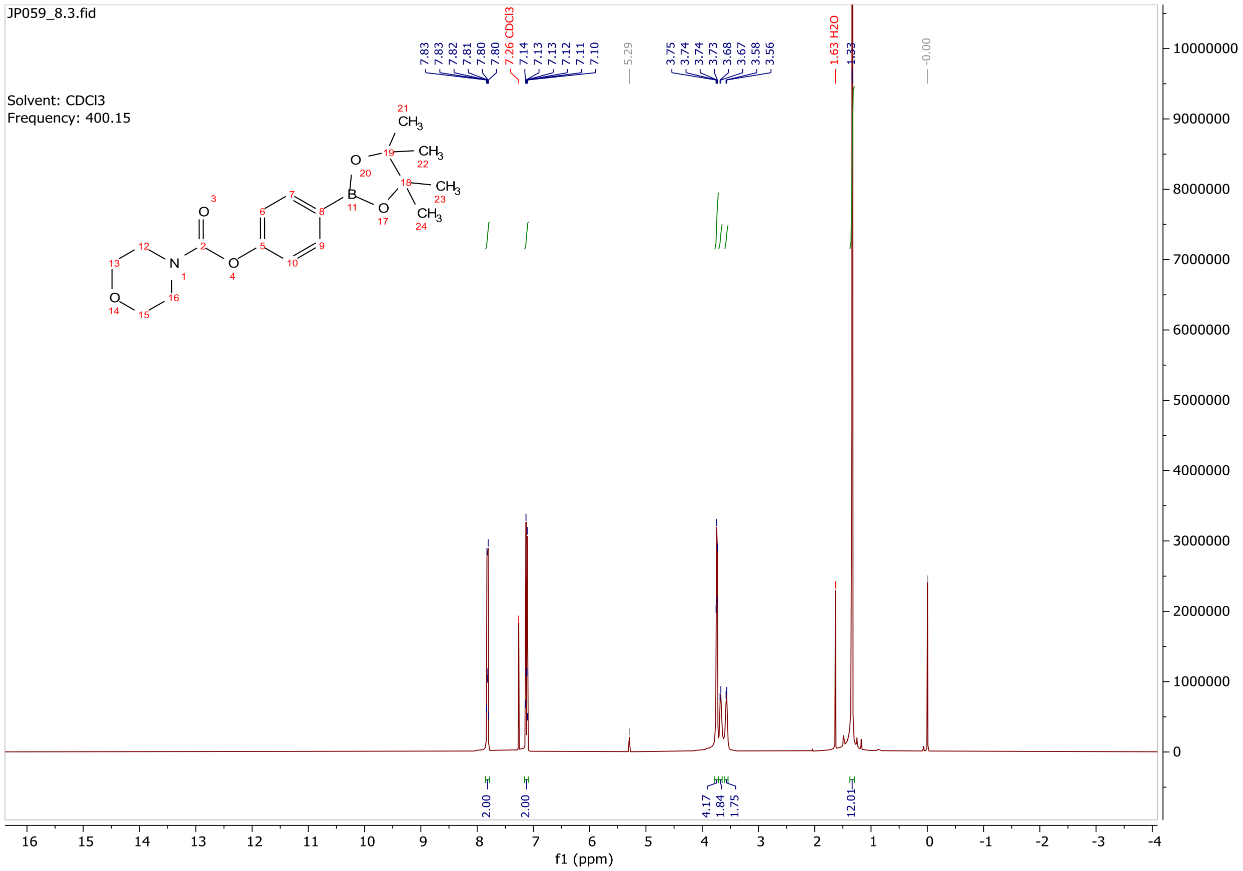

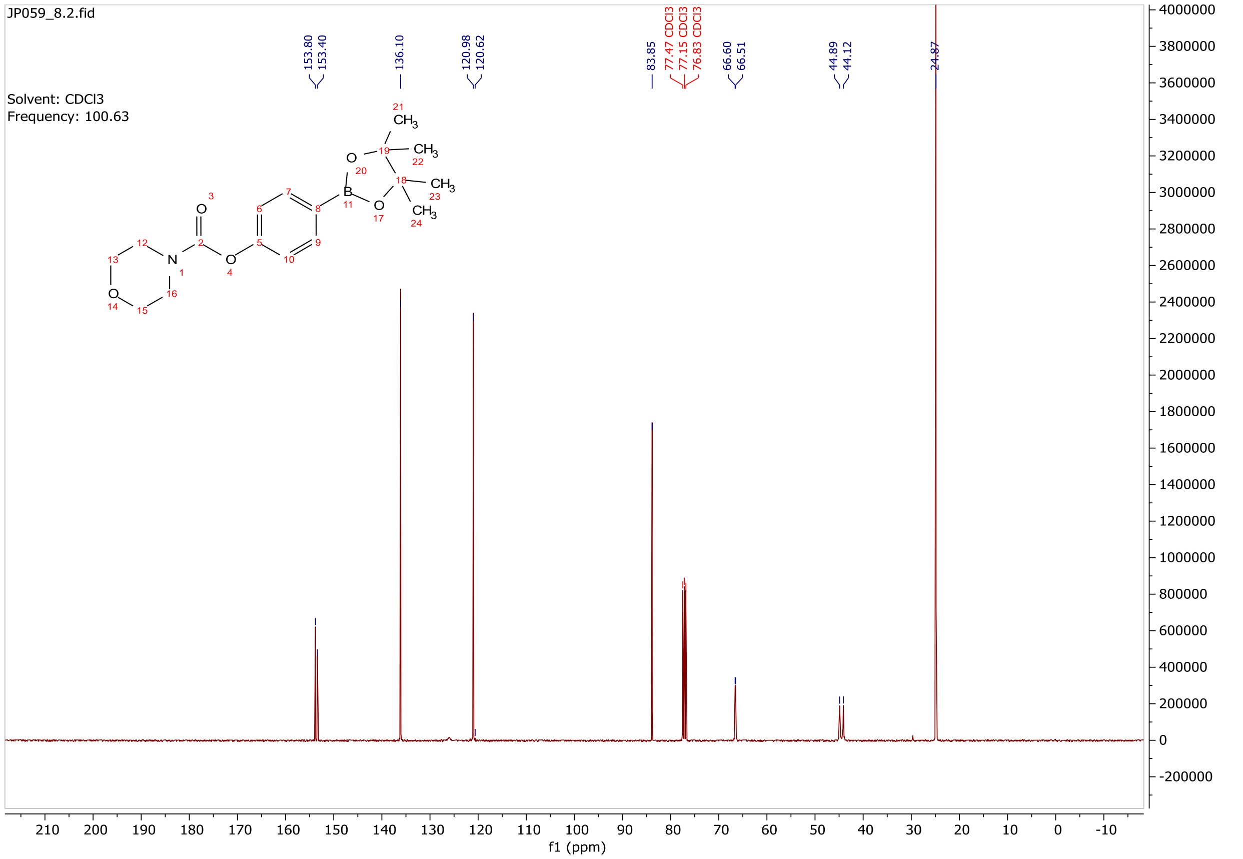

^{1}H and ^{13}C NMR and IR spectra of 4-bromophenyl morpholine-4-carboxylate (25)

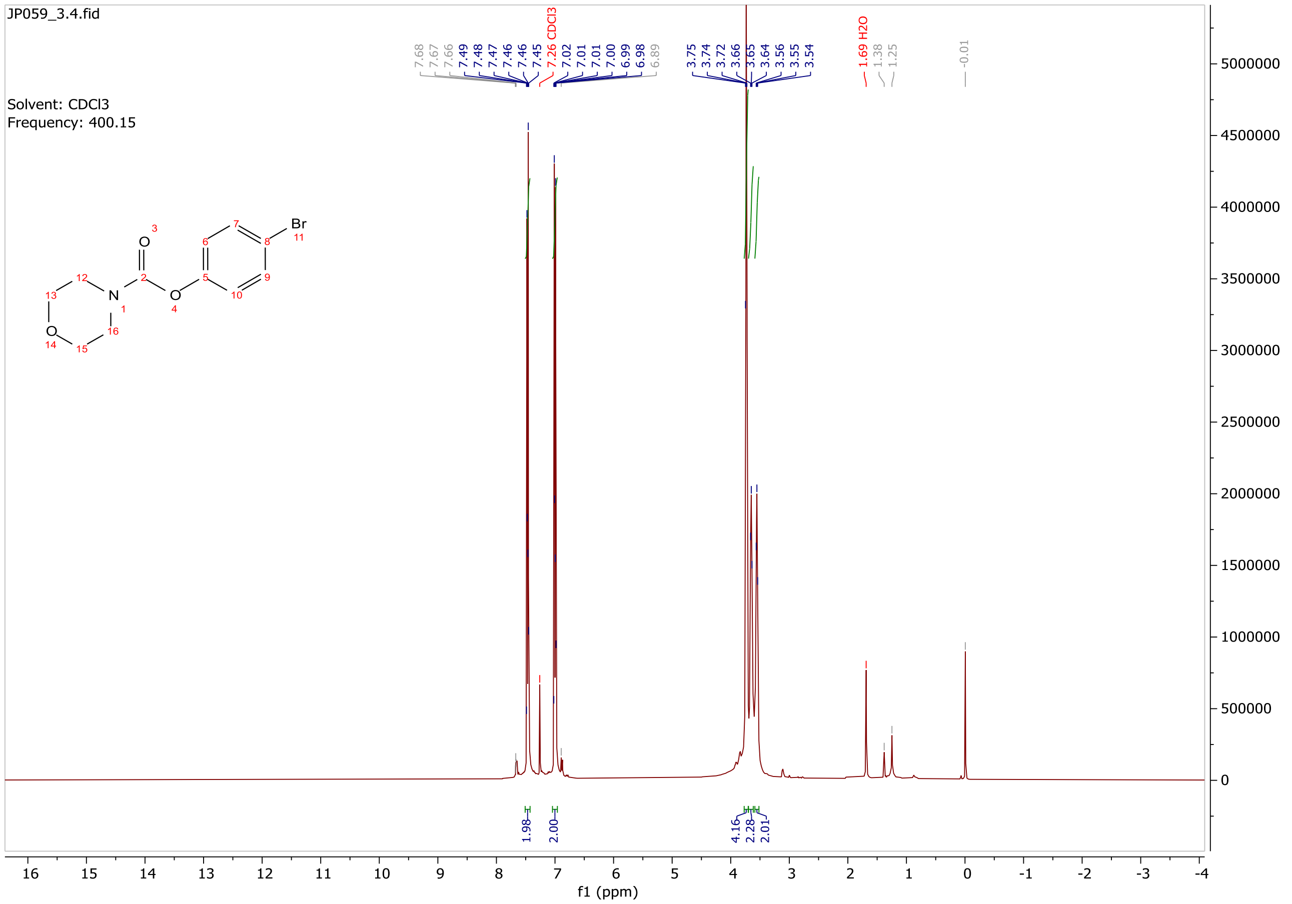

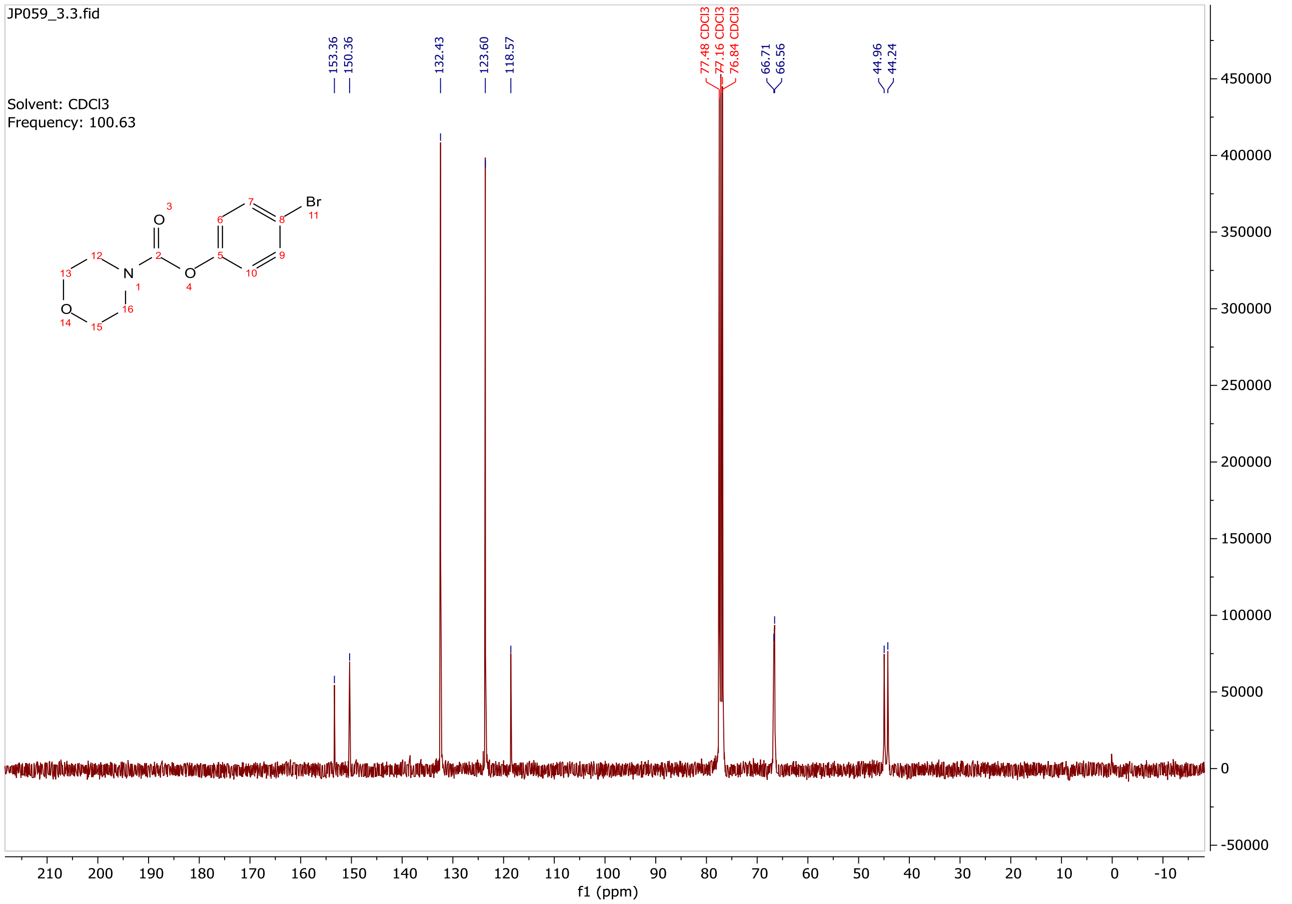

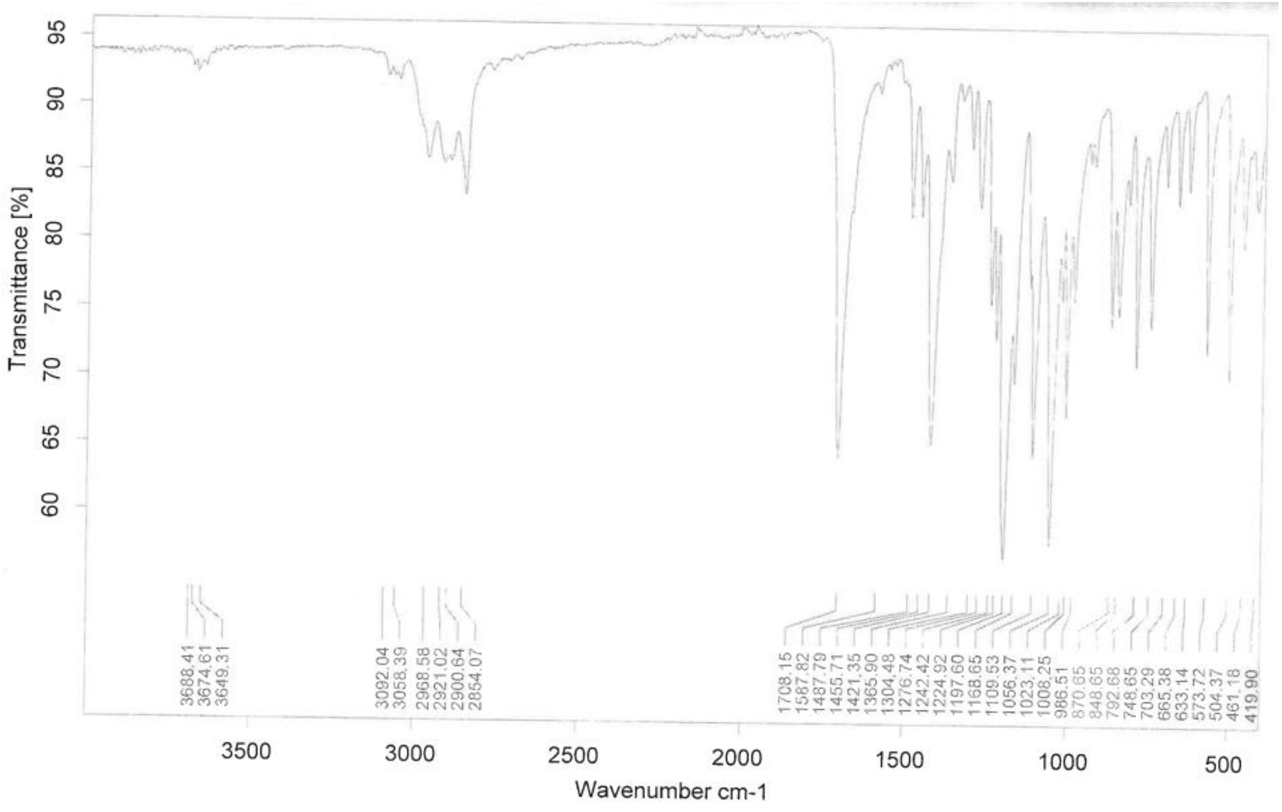

Transmittance [%]
95
90
85
80
75
70
65
60
3688.41
3674.61
3649.31
3092.04
3058.39
2968.58
2921.02
2900.64
2854.07
1708.15
1587.82
1487.79
1455.71
1421.35
1365.90
1304.48
1276.74
1242.42
1224.92
1197.60
1168.65
1109.53
1056.37
1023.11
1008.25
986.51
870.65
848.65
792.68
748.65
703.29
665.38
633.14
573.72
504.37
461.18
419.90
3500
3000
2500
2000
1500
1000
500
Wavenumber cm-1

^{1}H and ^{13}C NMR spectra of 3-bromophenyl morpholine-4-carboxylate (26)

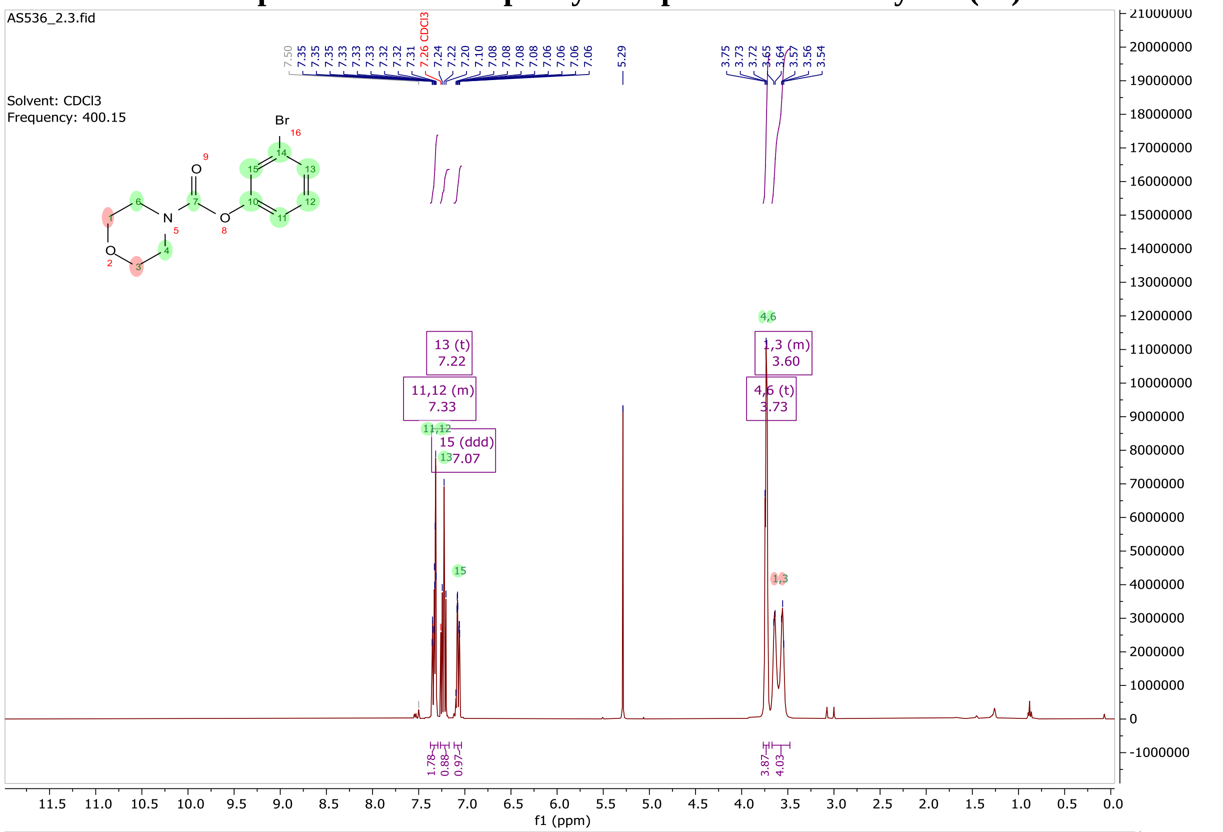

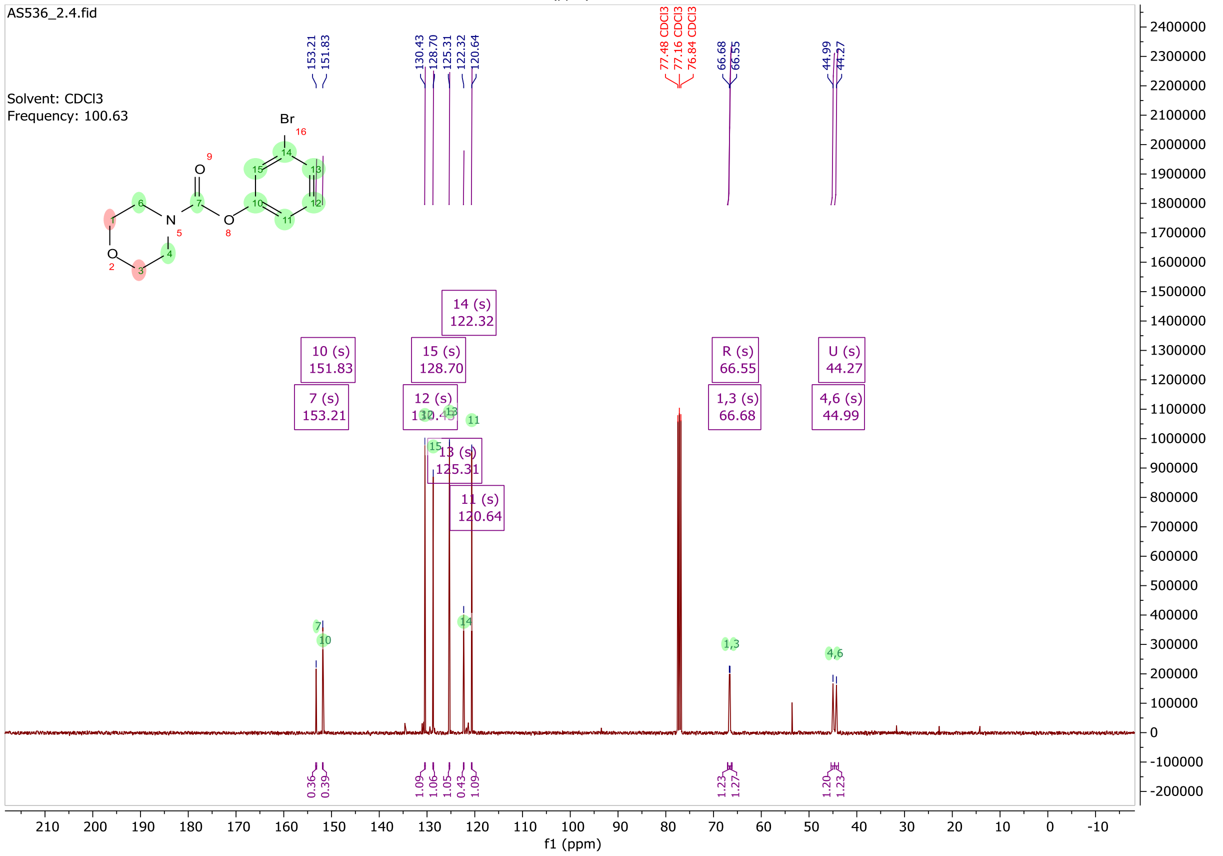

^{1}H and ^{13}C NMR spectra of 2-bromophenyl morpholine-4-carboxylate (27)

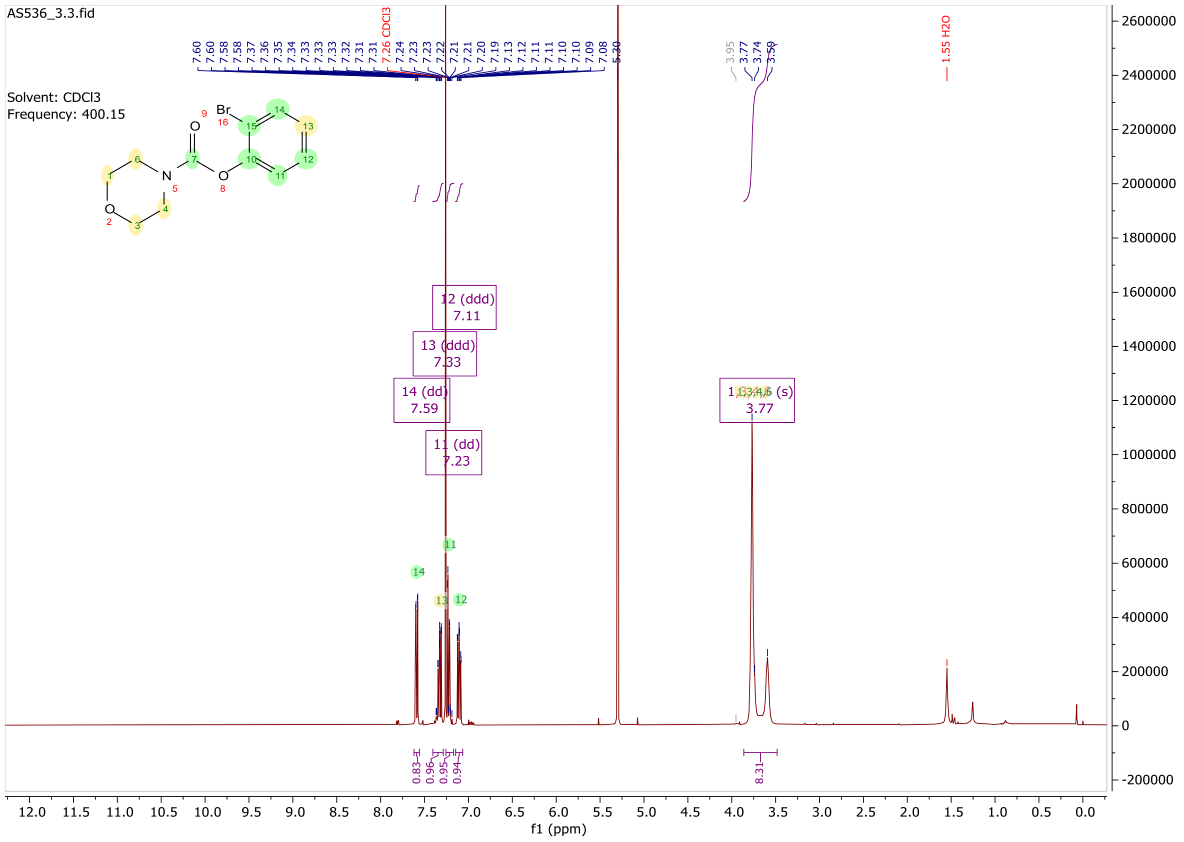

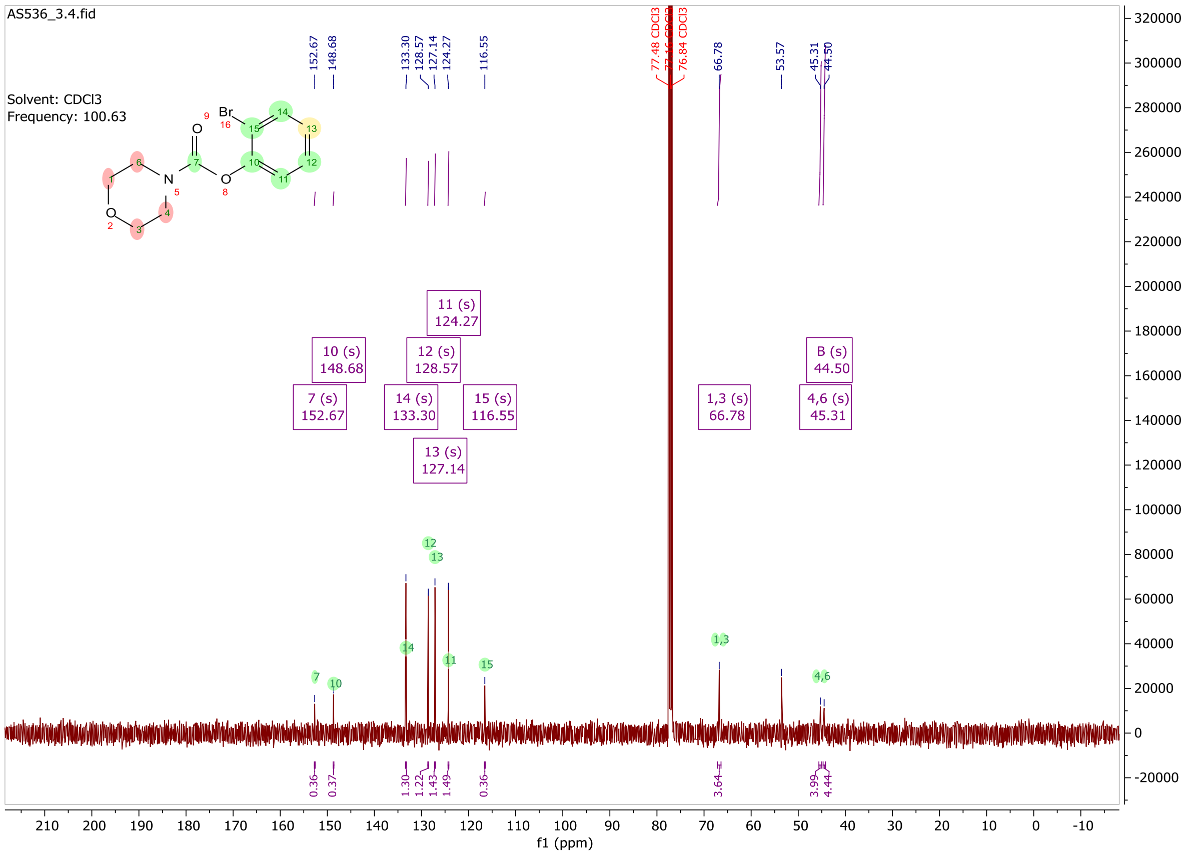

^{1}H and ^{13}C NMR spectra of phenyl morpholine-4-carboxylate (28)

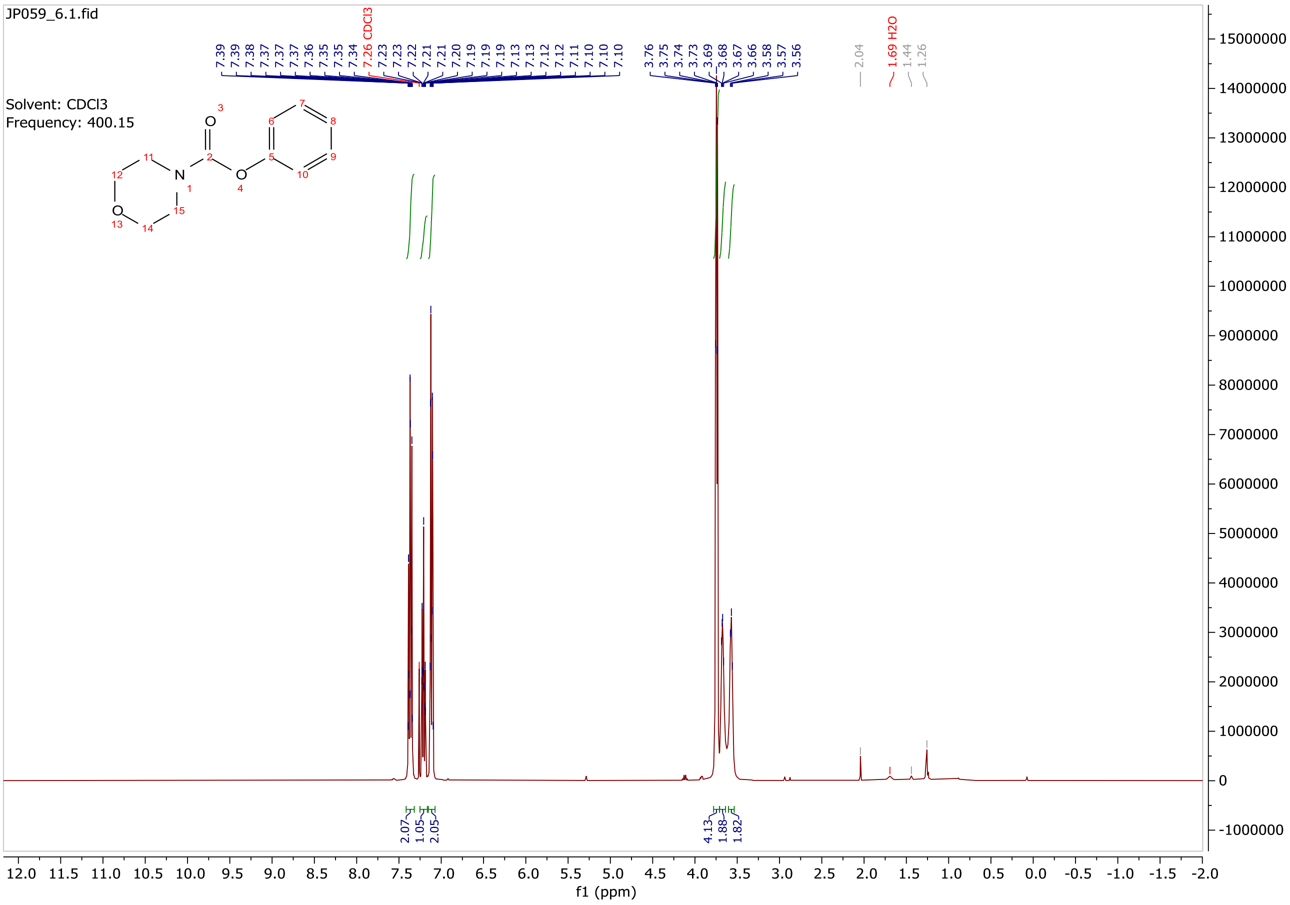

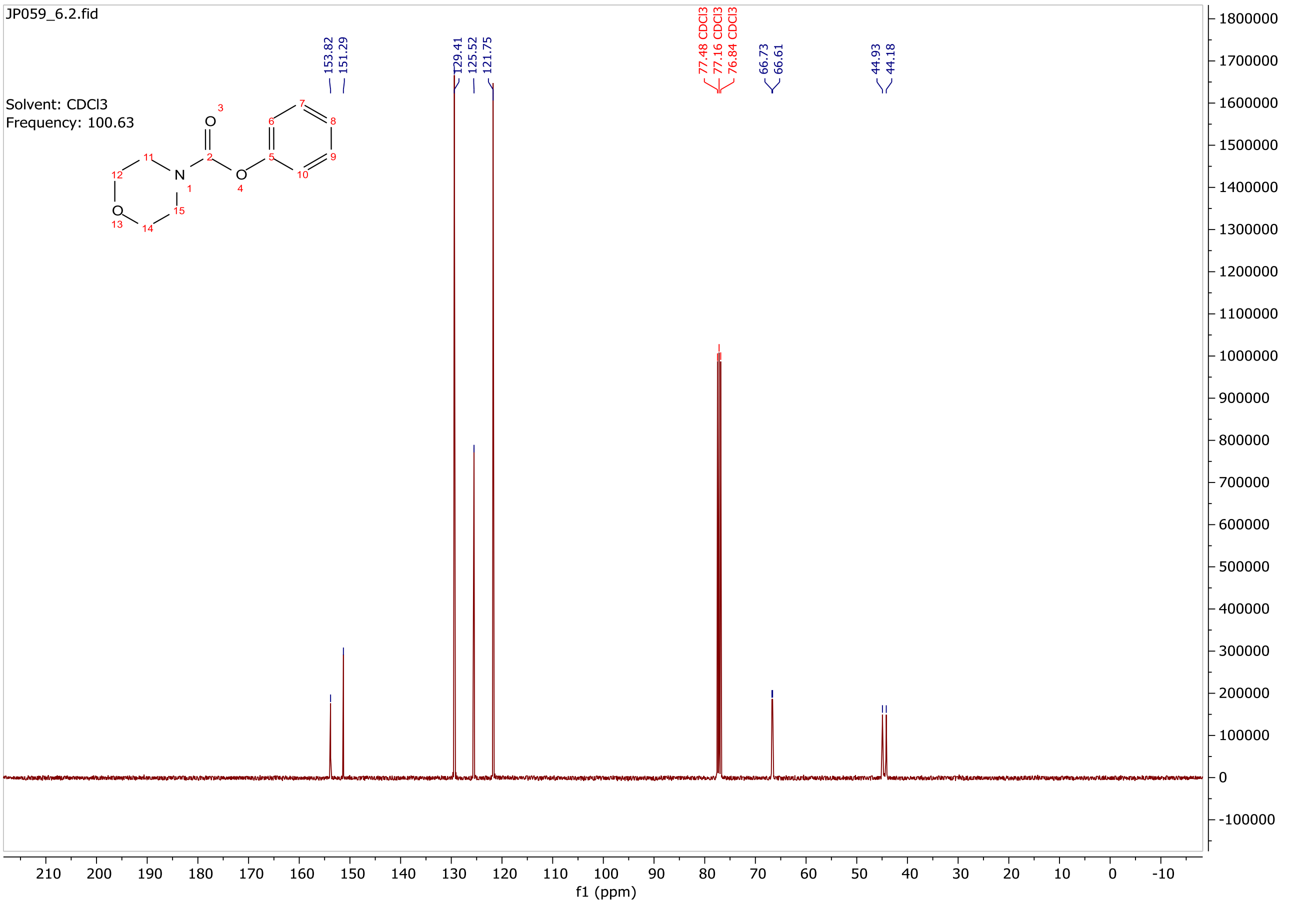

^{1}H and ^{13}C NMR spectra of m-tolyl morpholine-4-carboxylate (29)

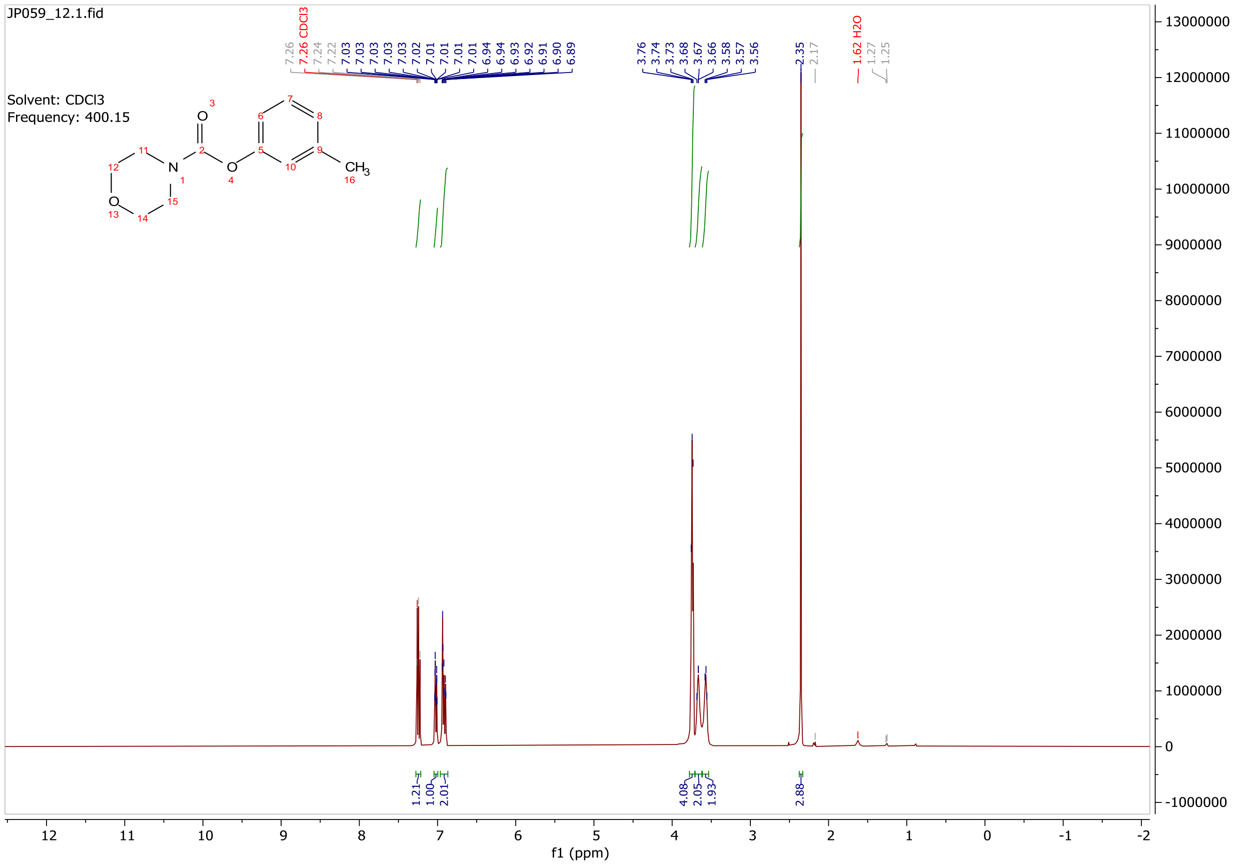

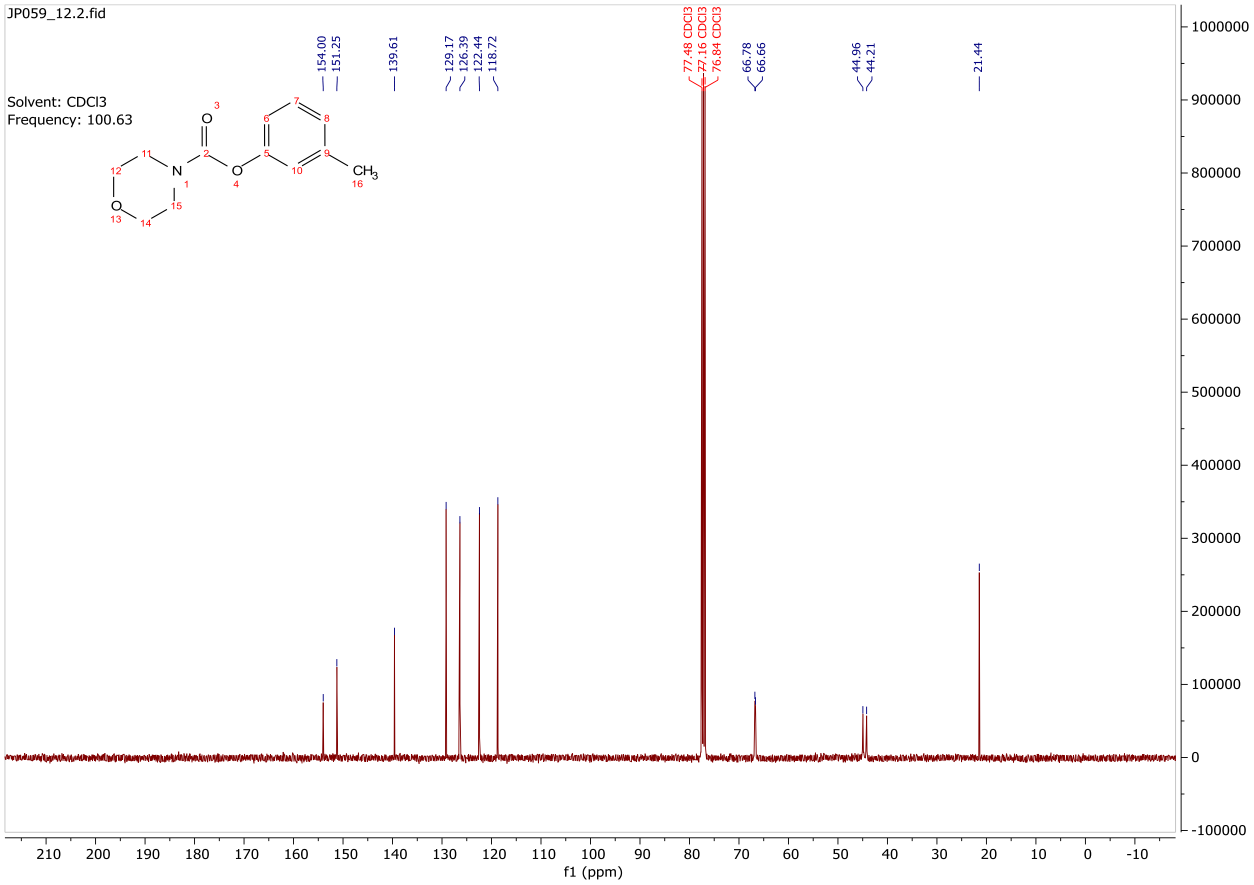

1^H and ^{13}C NMR spectra of 5-chloropyridin-2-yl morpholine-4-carboxylate (30)

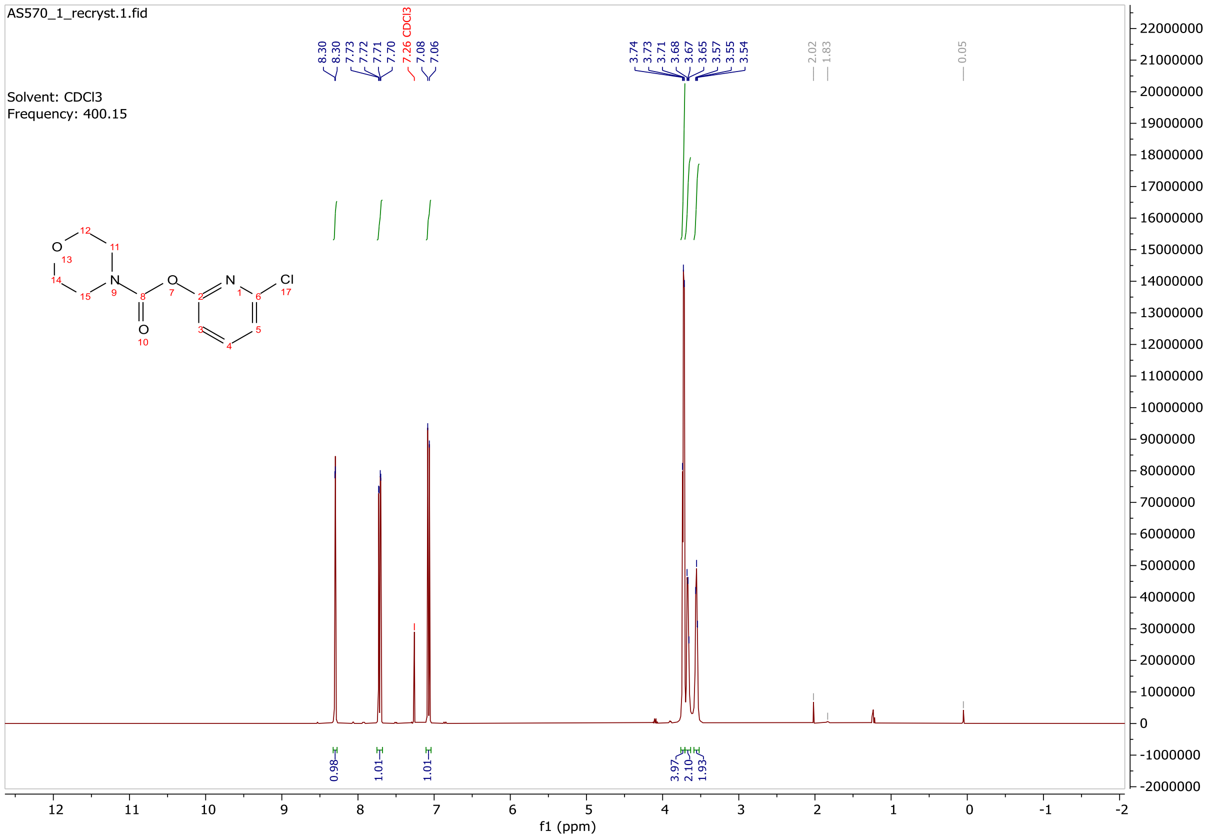

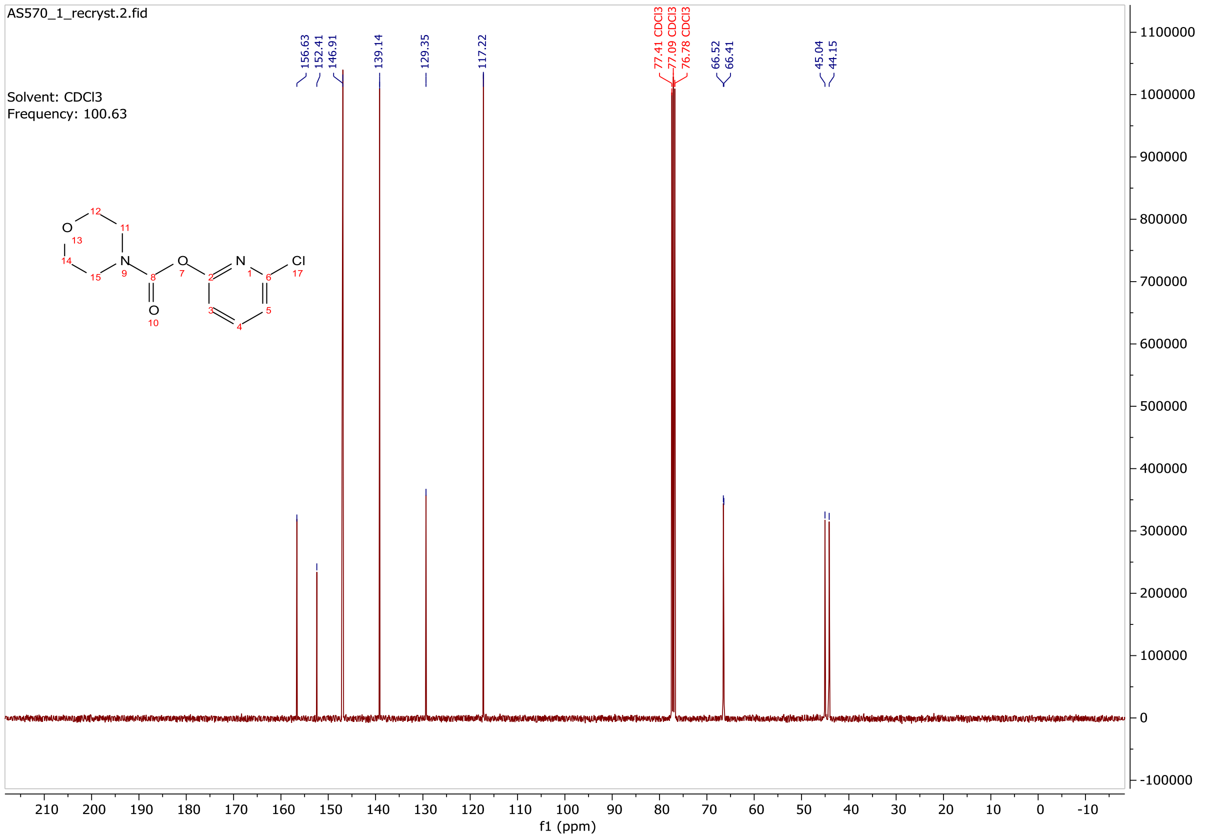

^{1}H and ^{13}C NMR spectra of pyridin-3-yl morpholine-4-carboxylate (31)

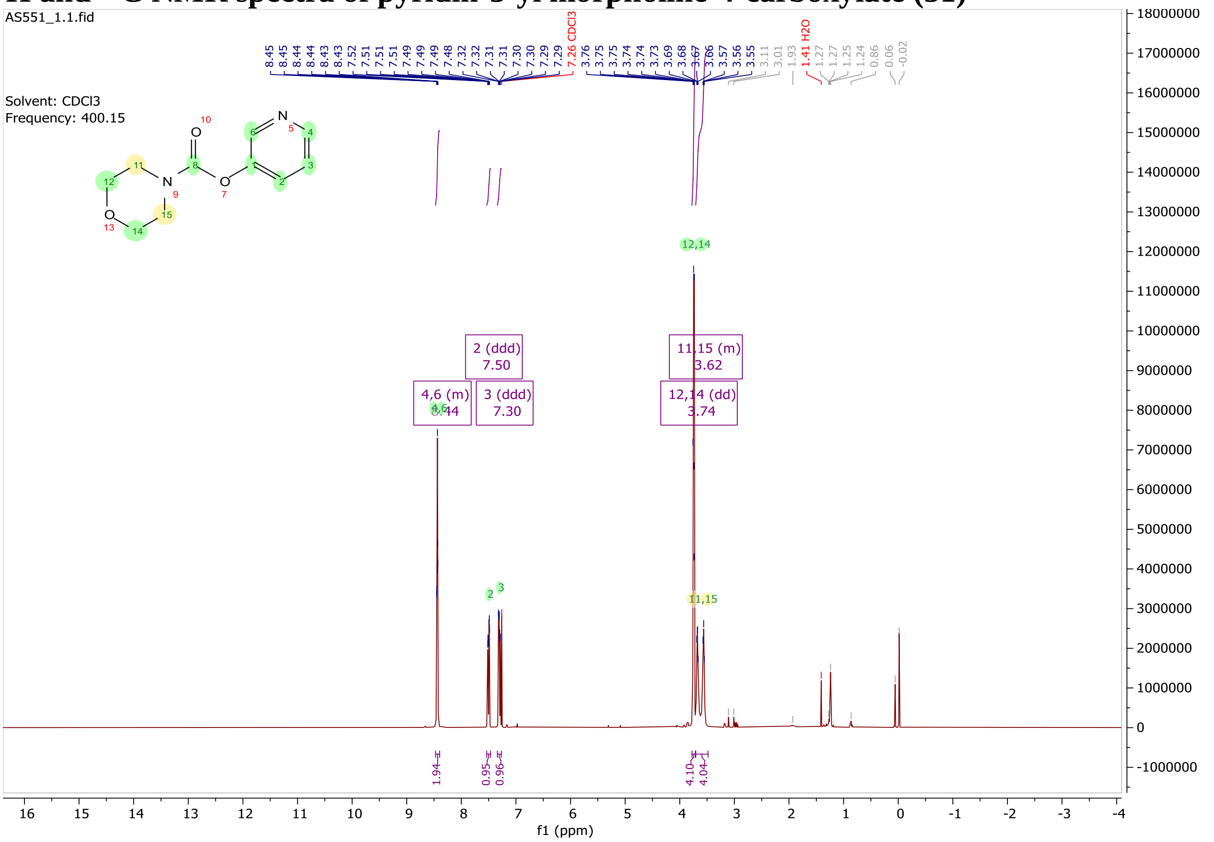

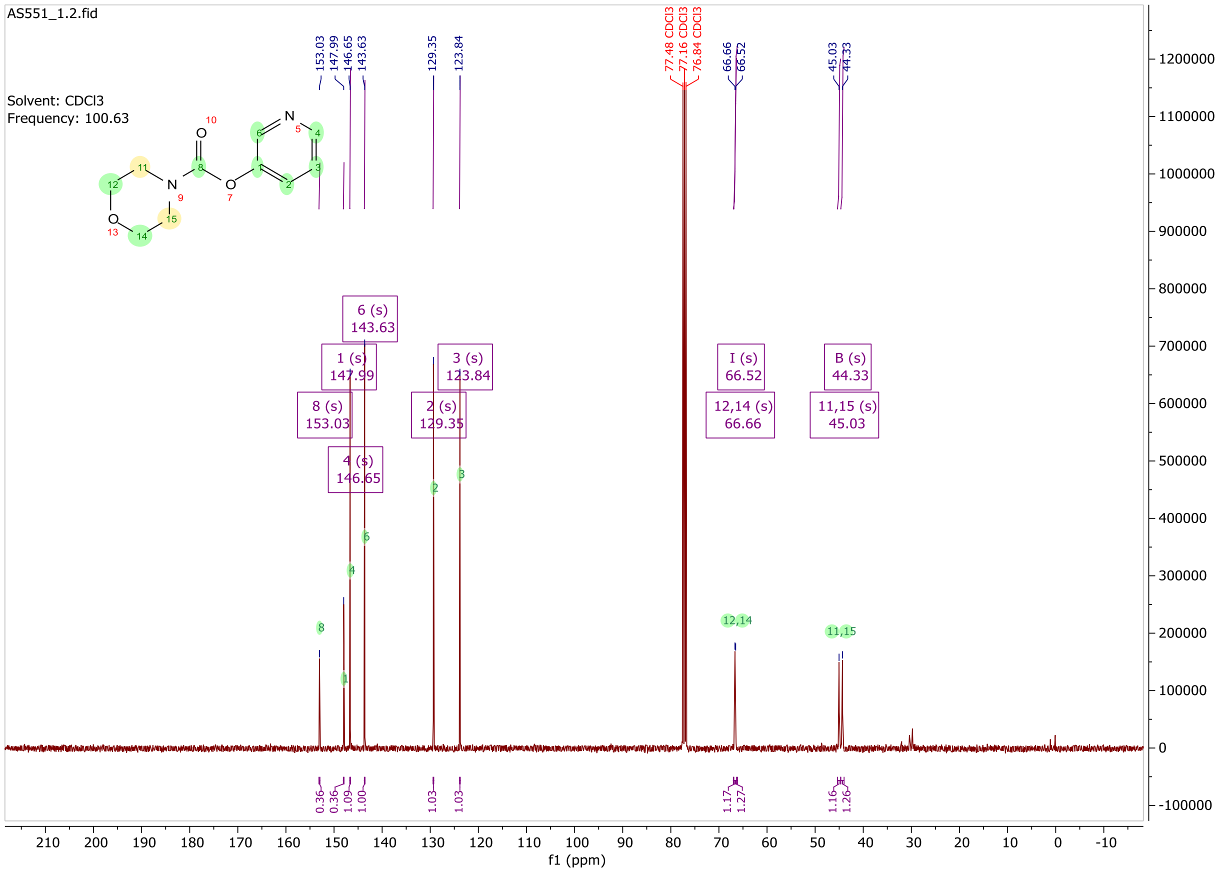

^{1}H and ^{13}C NMR spectra of 2-fluoropyridin-4-yl morpholine-4-carboxylate (32)

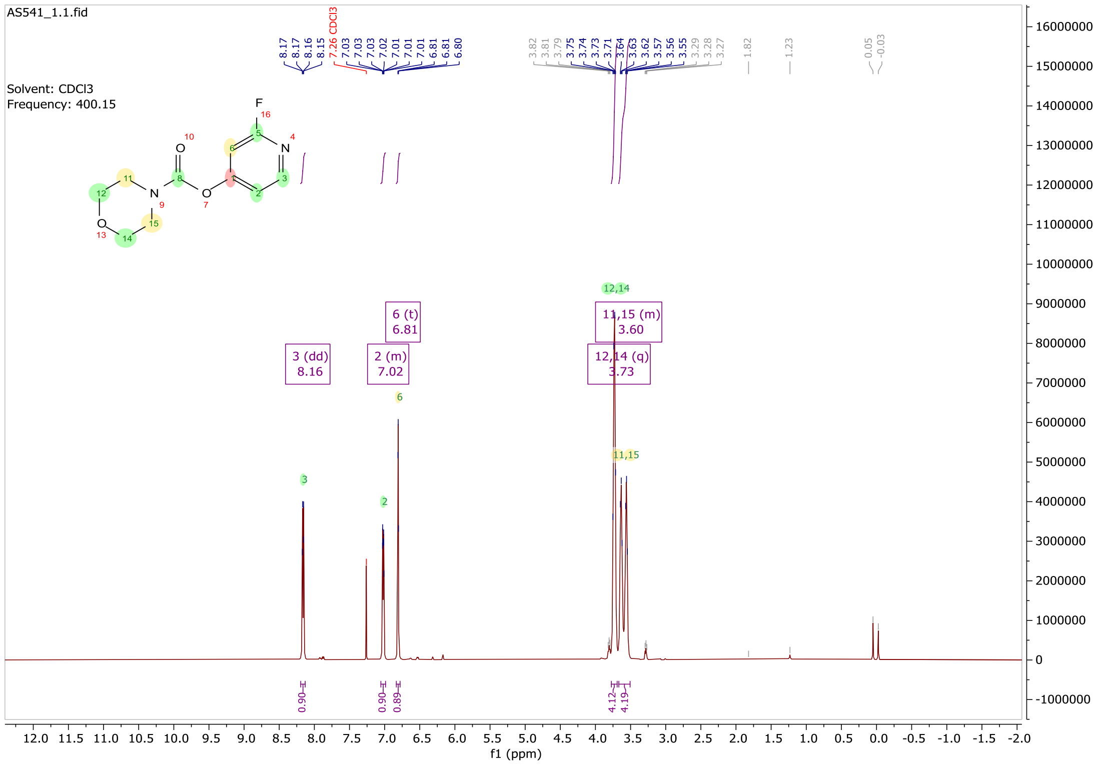

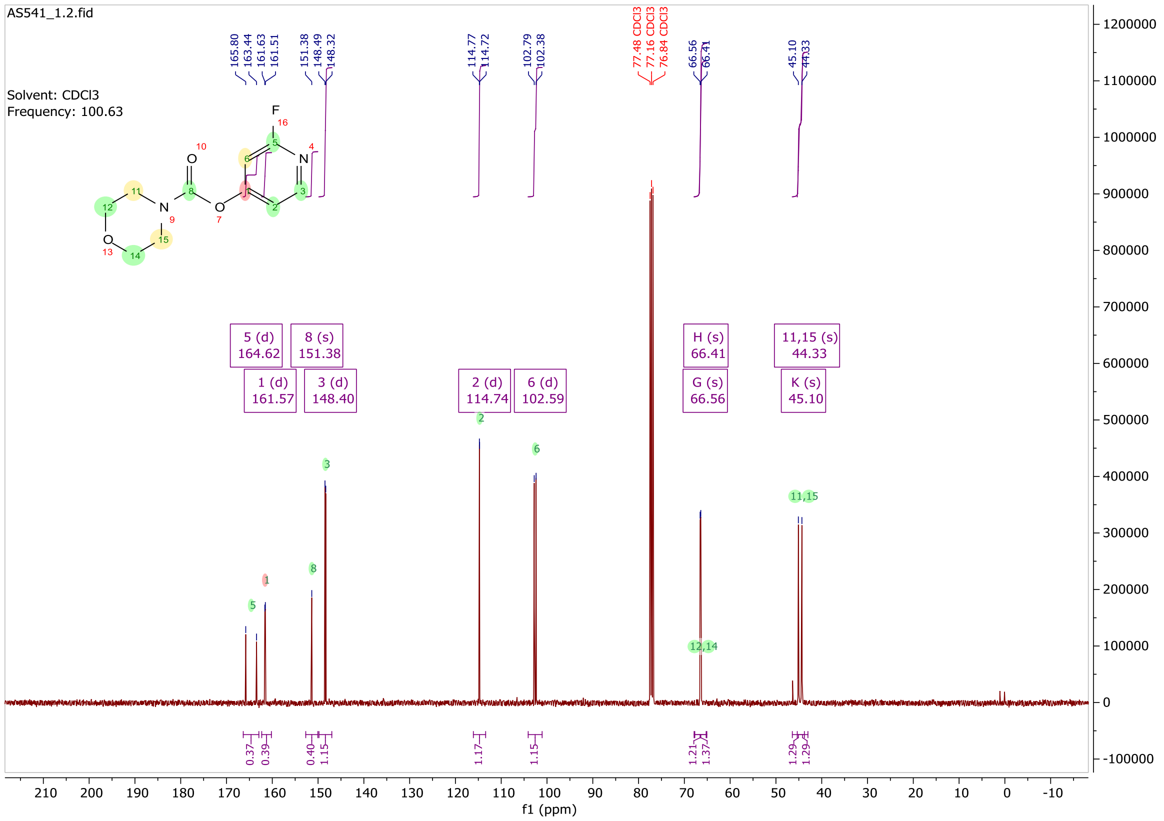

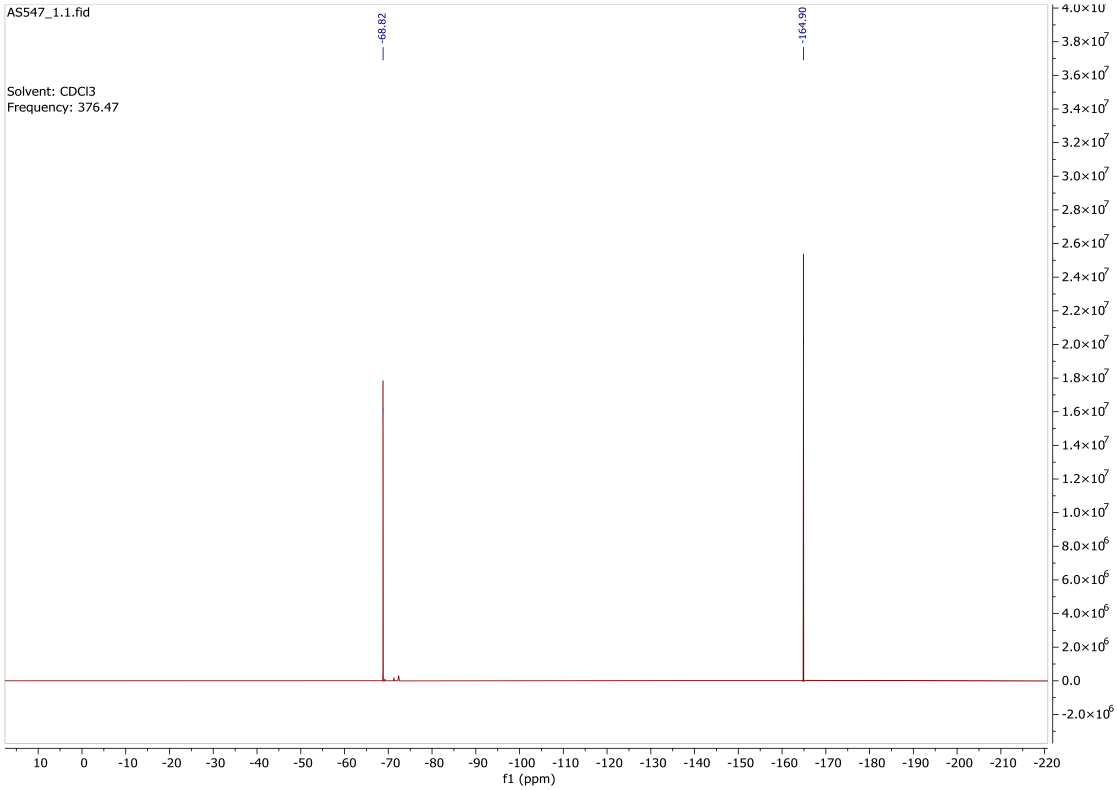

^{1}H and ^{13}C NMR spectra of 6-methoxypyridin-2-yl morpholine-4-carboxylate (33)

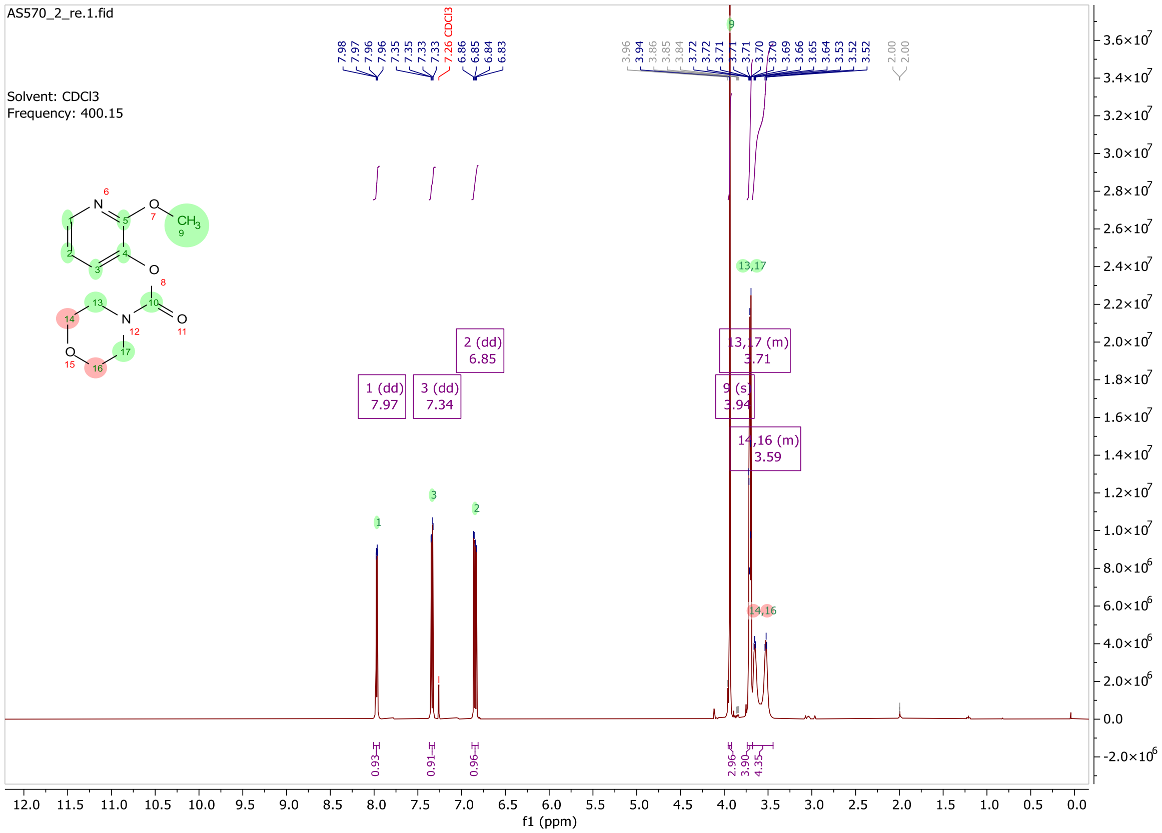

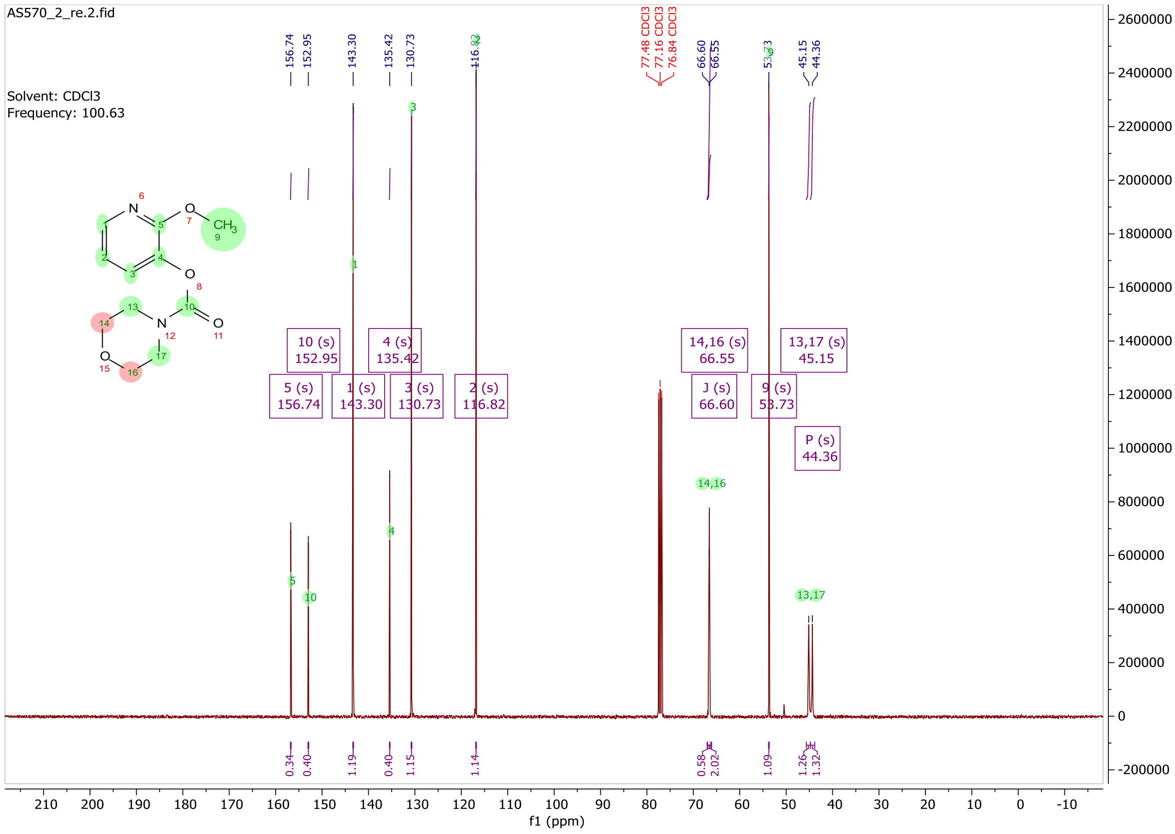
AS570_2_re.2.fid
Solvent: CDCl3
Frequency: 100.63
156.74
152.95
143.30
135.42
130.73
116.82
77.48 CDCl3
77.16 CDCl3
76.84 CDCl3
66.60
66.55
53.73
45.15
44.36
10 (s) 152.95
4 (s) 135.42
14,16 (s) 66.55
13,17 (s) 45.15
5 (s) 156.74
1 (s) 143.30
3 (s) 130.73
2 (s) 116.82
J (s) 66.60
9 (s) 53.73
P (s) 44.36
0.34
0.40
1.19
0.40
1.15
1.14
0.58
2.02
1.09
1.26
1.32
f1 (ppm)

17.3 OTHER NMR SPECTRA

^1H and ^{13}C NMR spectra of the Pinzer ligand (N2,N6-bis(2,6-diisopropylphenyl)pyridine-2,6-dicarboxamide)

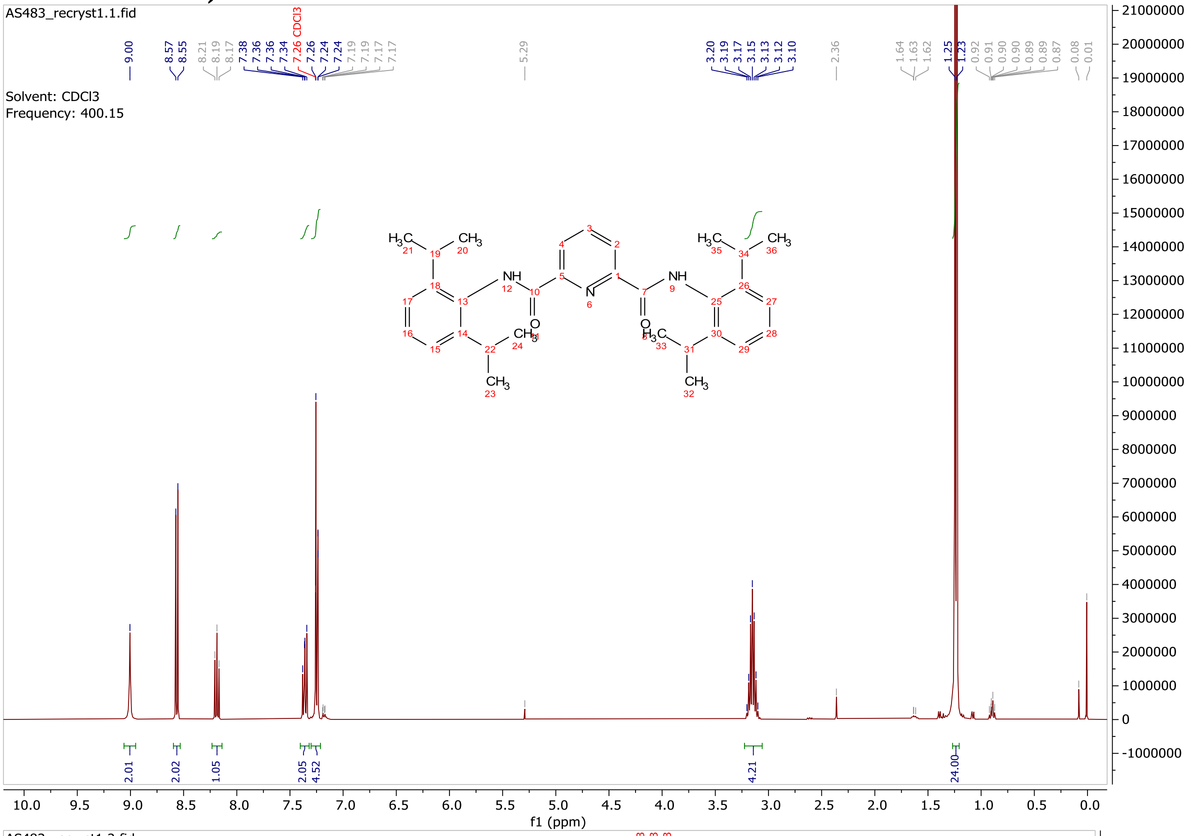

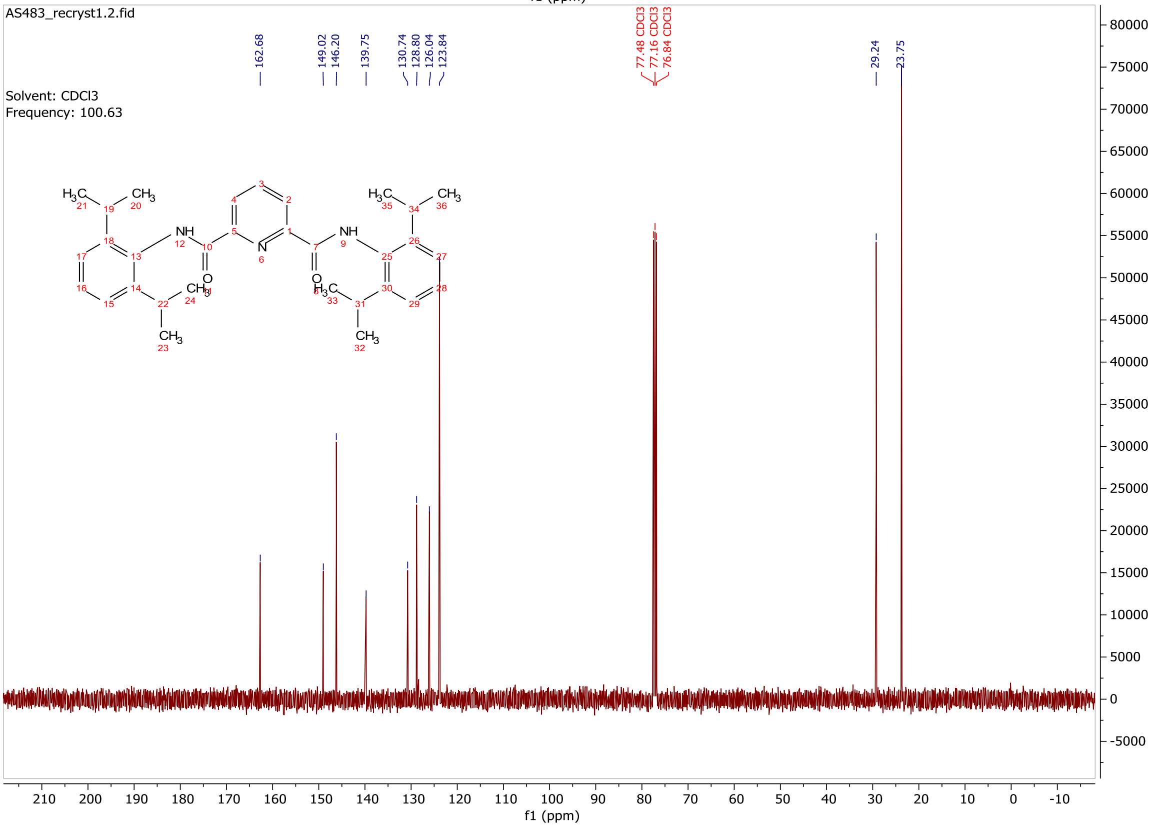

^{1}H spectra of N,N,N,N-tetramethyl-[2,2'-bipyridine]-4,4'-diamine

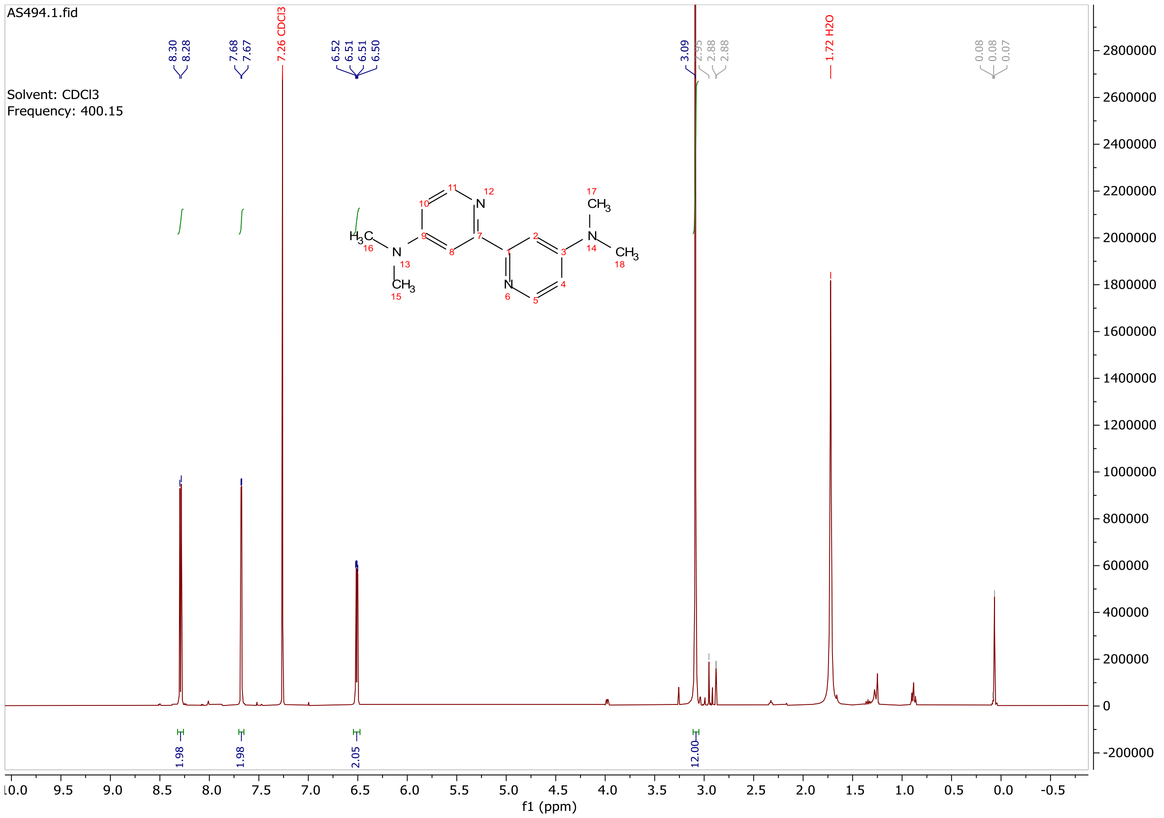

^{1}H spectra of 4,4'-di(pyrrolidin-1-yl)-2,2'-bipyridine

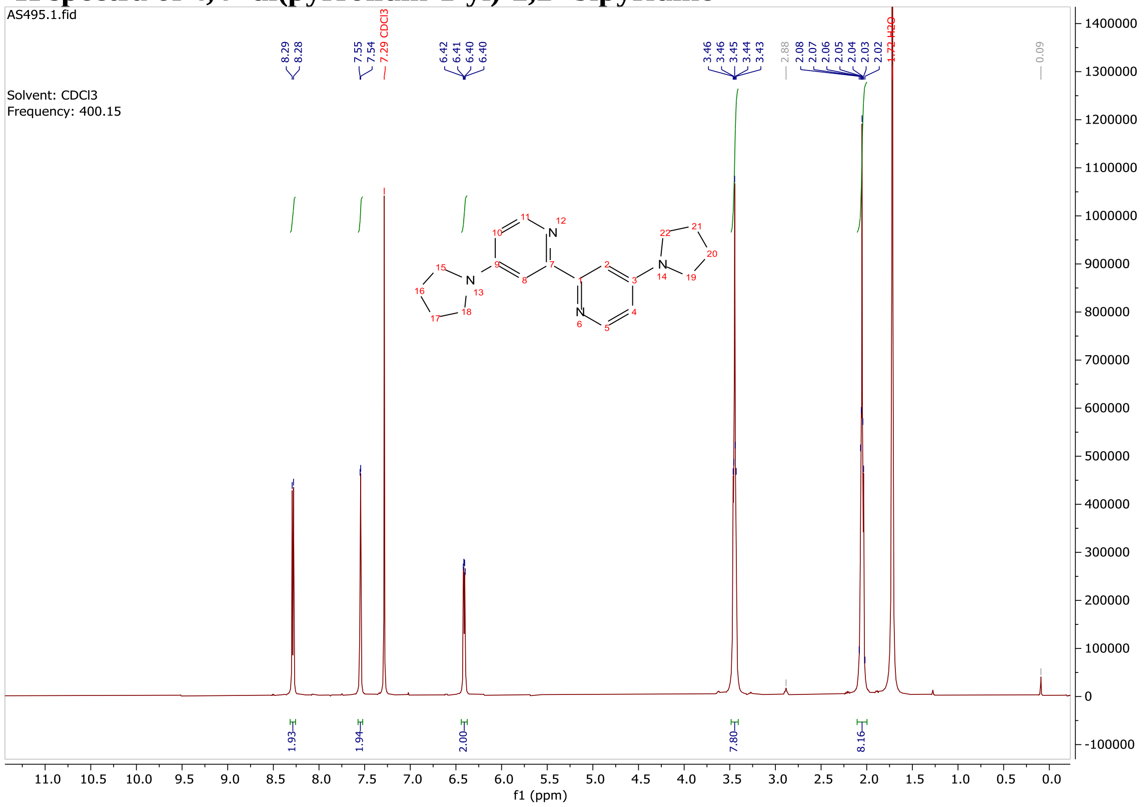

^{1}H NMR spectra of 4-(trifluoromethyl)phenyl butylcarbamate (34)

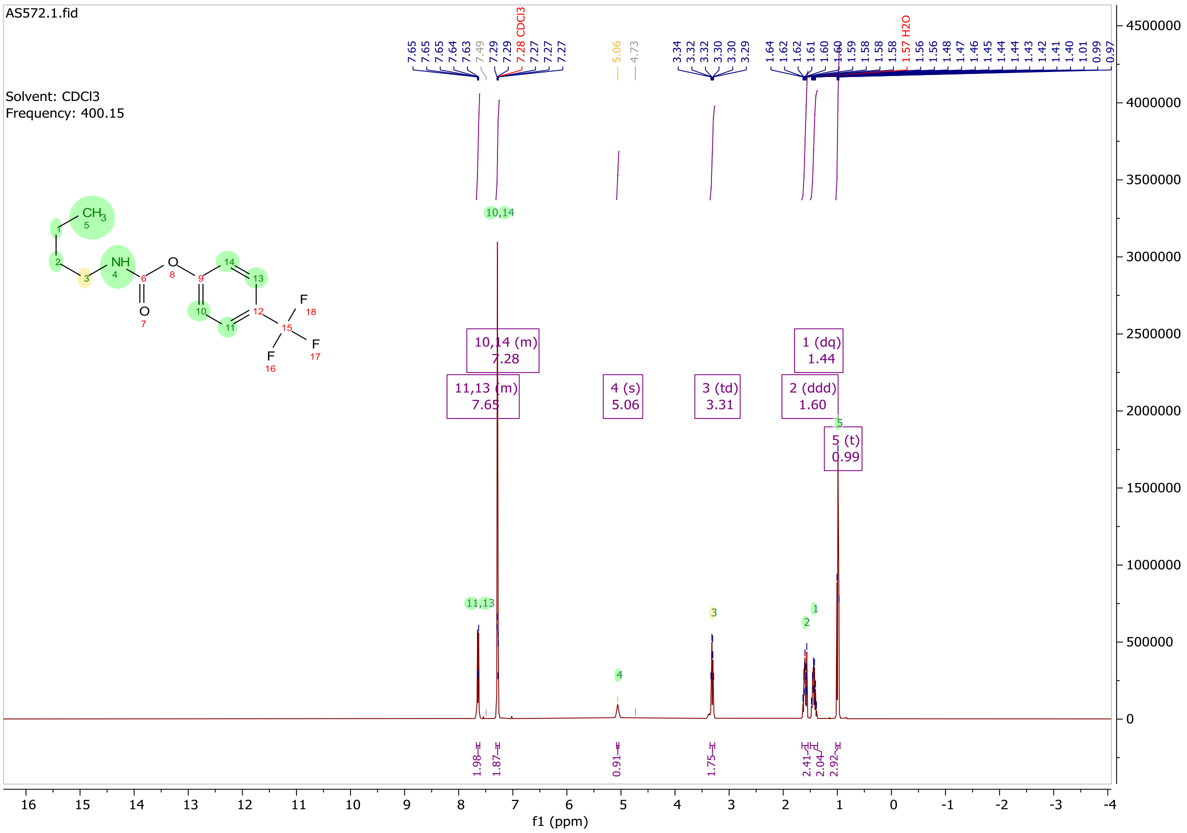

^{1}H and ^{13}C NMR spectra of 1-benzylpiperazine

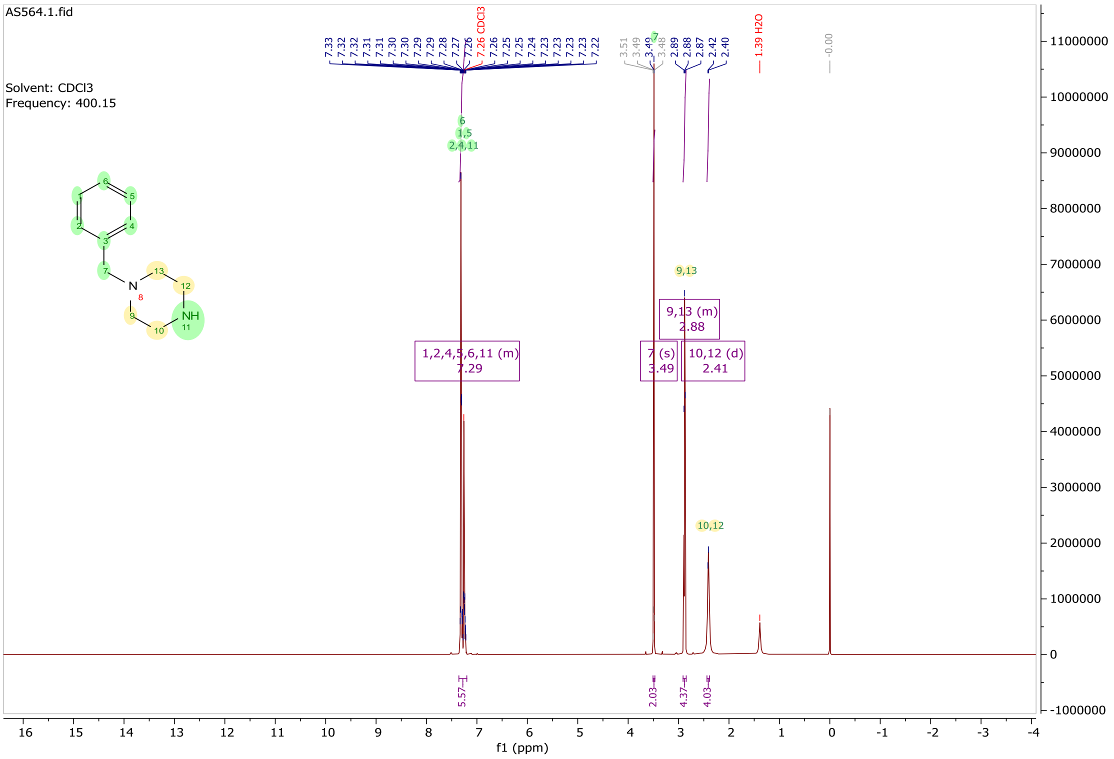

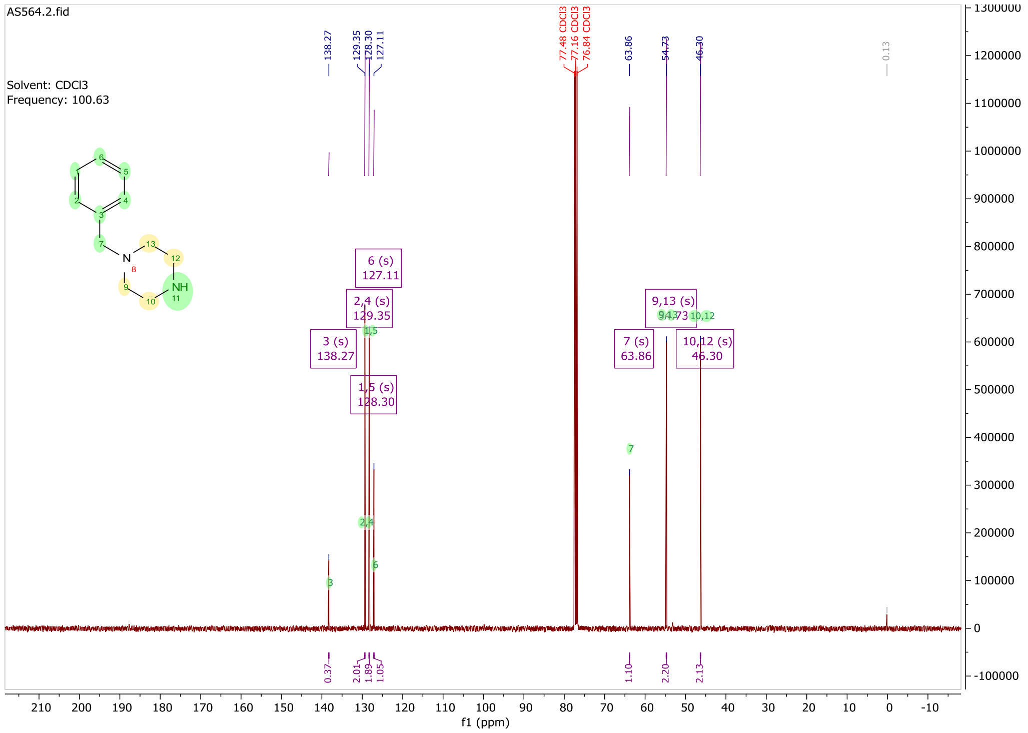
AS564.2.fid
Solvent: CDCl3
Frequency: 100.63
138.27
129.35
128.30
127.11
77.48 CDCl3
77.16 CDCl3
76.84 CDCl3
63.86
54.73
46.30
0.13
6 (s)
127.11
2,4 (s)
129.35
3 (s)
138.27
1,5 (s)
128.30
9,13 (s)
54.73
7 (s)
63.86
10,12 (s)
46.30
NH
N
0.37
2.01
1.89
1.05
1.10
2.20
2.13
f1 (ppm)

^{1}H and ^{31}P NMR spectra in C_6D_6 and ^{1}H and ^{13}C NMR spectra in $CDCl_3$ of $Ni(PPh_3)_2$(o-tolyl)Br

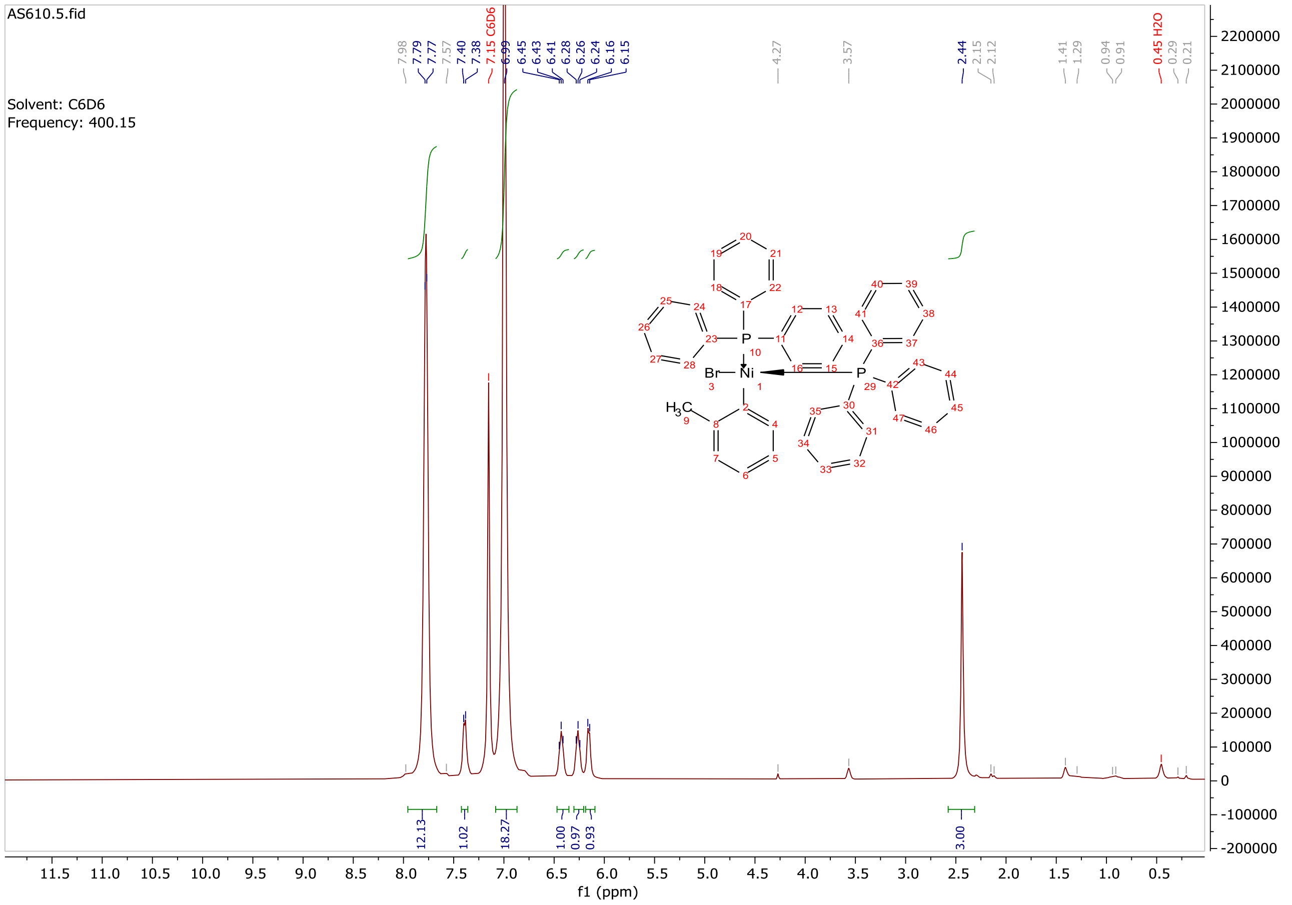

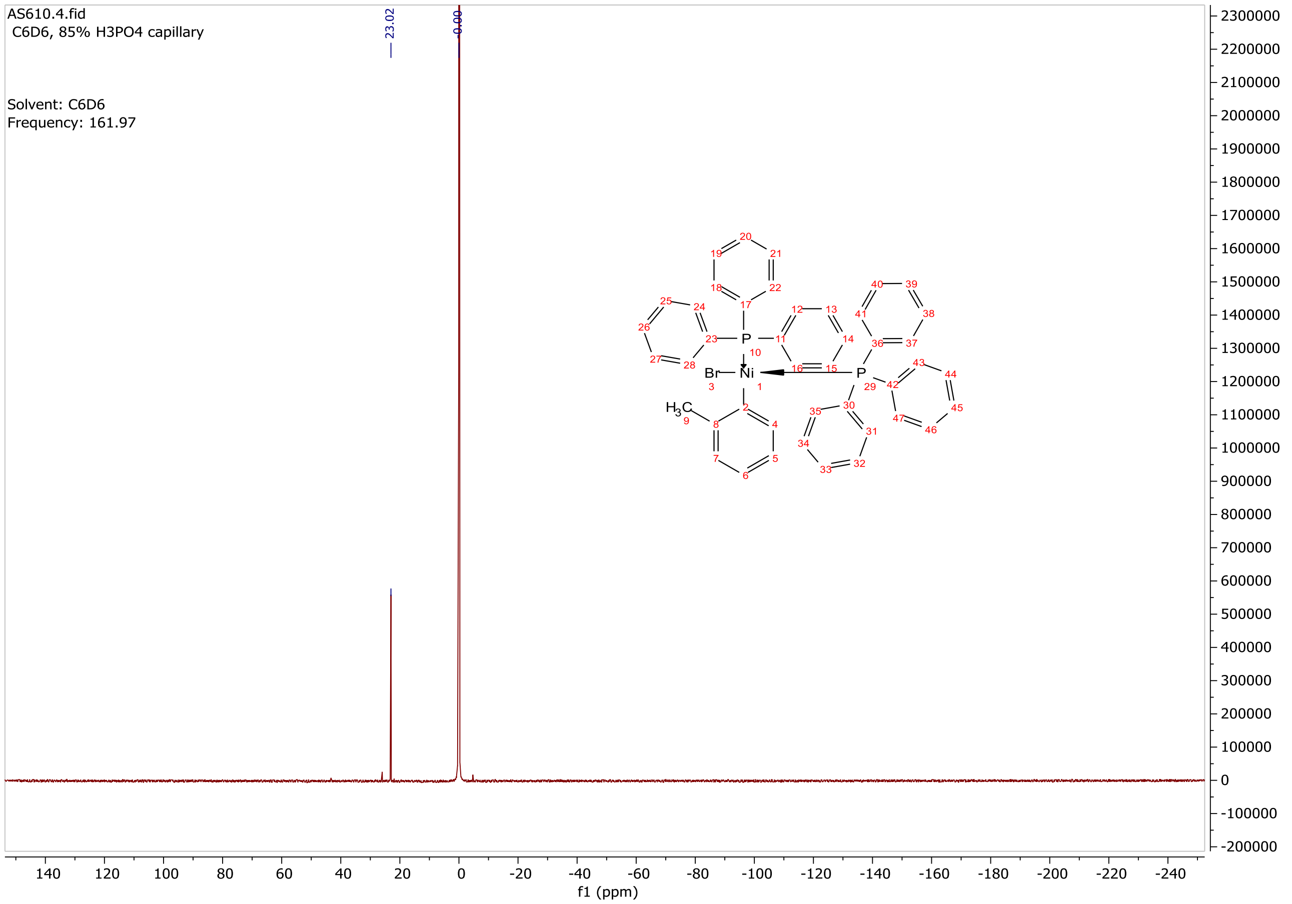

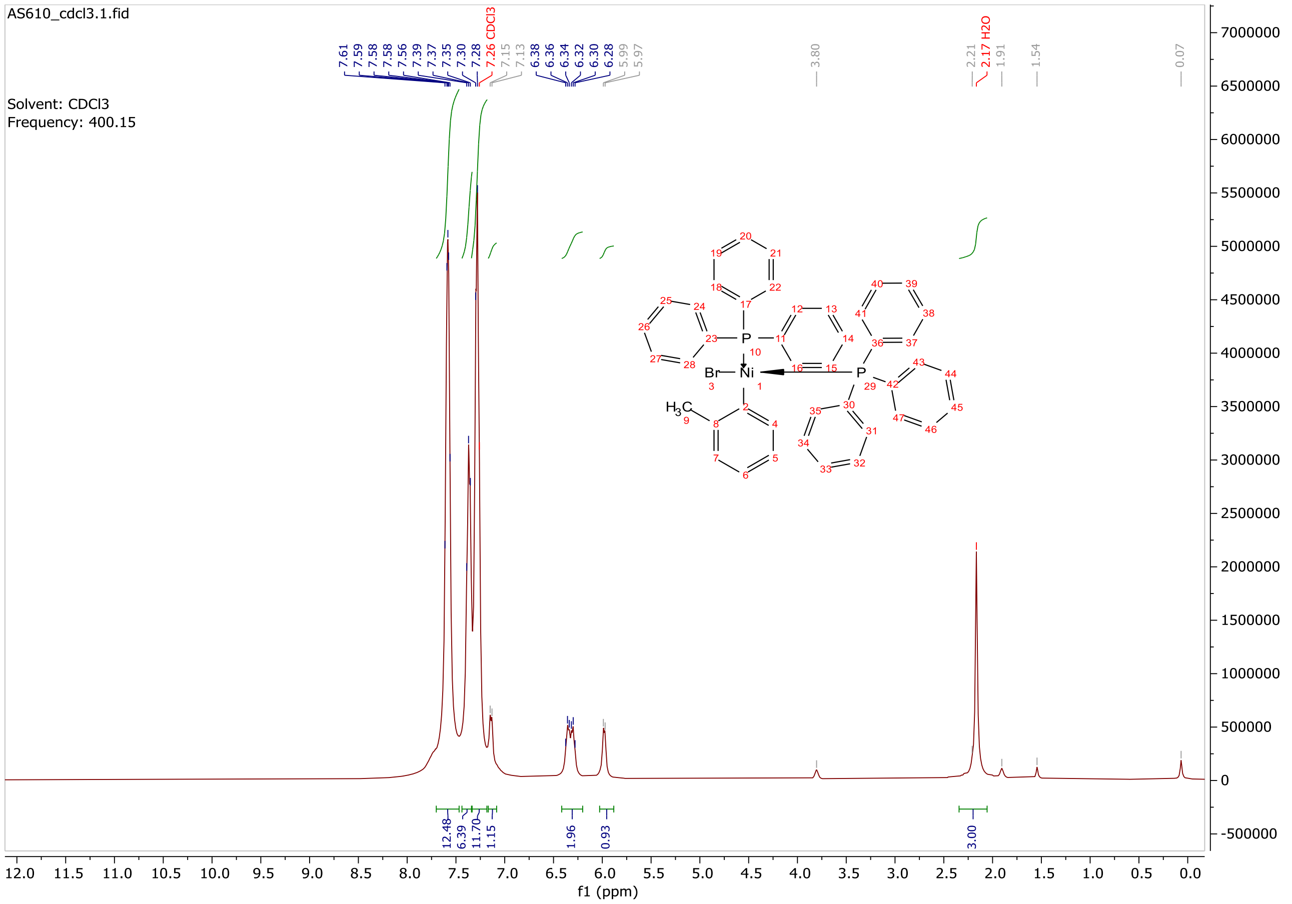
AS610_cdcl3.1.fid
Solvent: CDCl3
Frequency: 400.15
7.61 7.59 7.58 7.58 7.56 7.39 7.37 7.35 7.30 7.28 7.26 CDCl3 7.15 7.13 6.38 6.36 6.34 6.32 6.30 6.28 5.99 5.97 3.80 2.21 2.17 H2O 1.91 1.54 0.07
12.48 6.39 11.70 1.15 1.96 0.93 3.00
f1 (ppm)

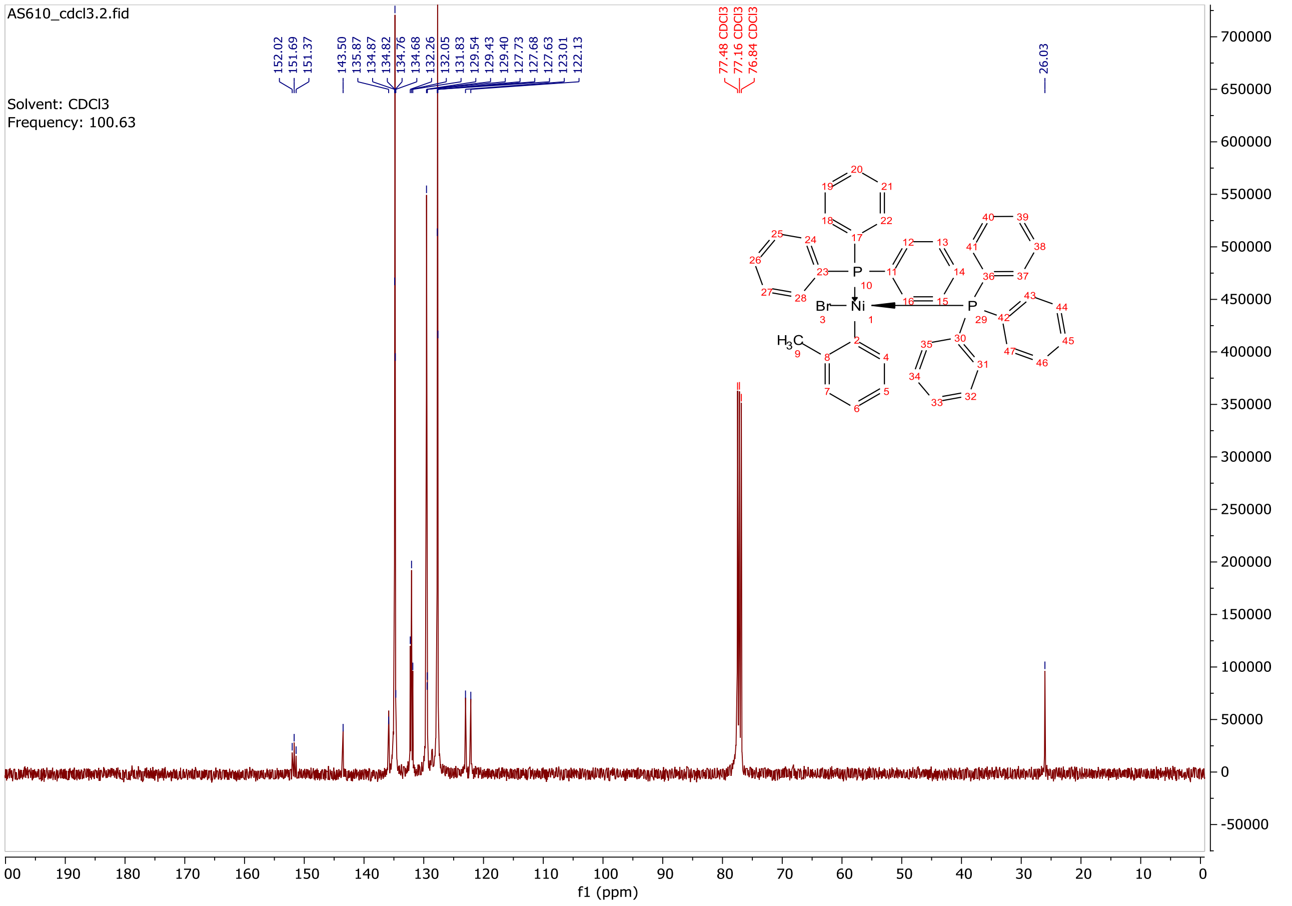
AS610_cdcl3.2.fid
Solvent: CDCl3
Frequency: 100.63
152.02 151.69 151.37 143.50 135.87 134.87 134.82 134.76 134.68 132.26 132.05 131.83 129.54 129.43 129.40 127.73 127.68 127.63 123.01 122.13 77.48 CDCl3 77.16 CDCl3 76.84 CDCl3 26.03
f1 (ppm)

^{1}H and ^{13}C NMR spectra of dtbbpyNi(o-tolyl)Br

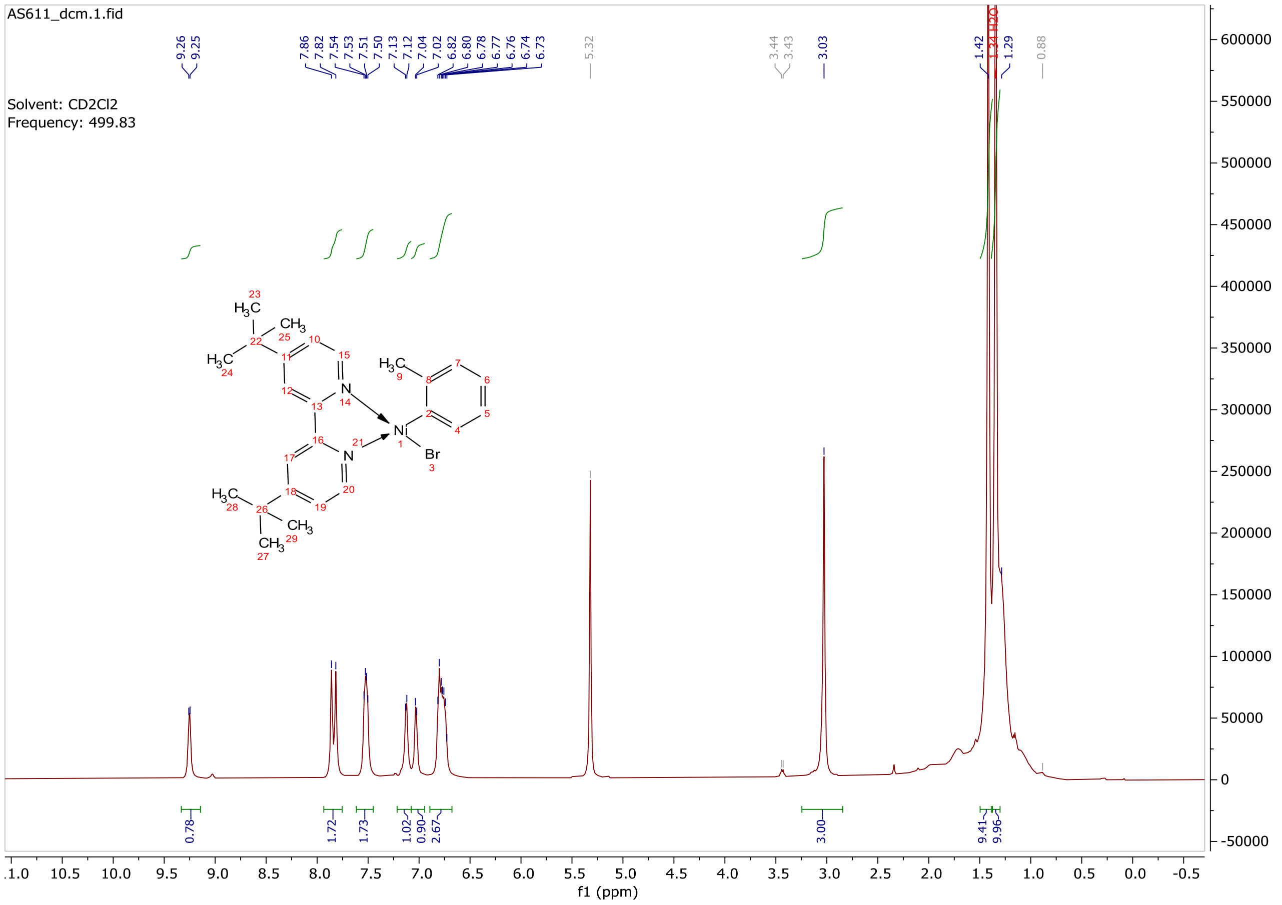

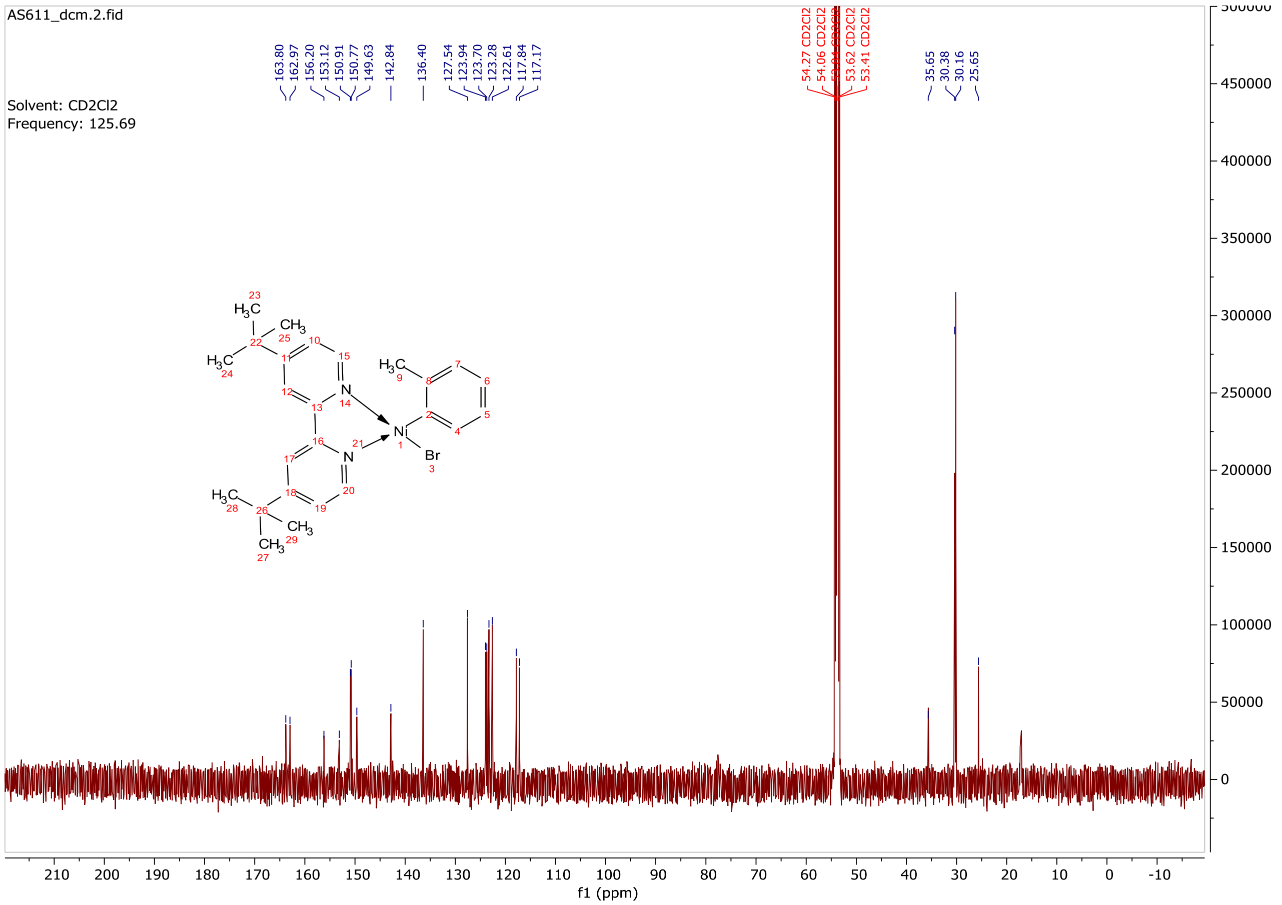

^{1}H and ^{13}C NMR and IR spectra of TMGH^{+} morpholine-N-carboxylate

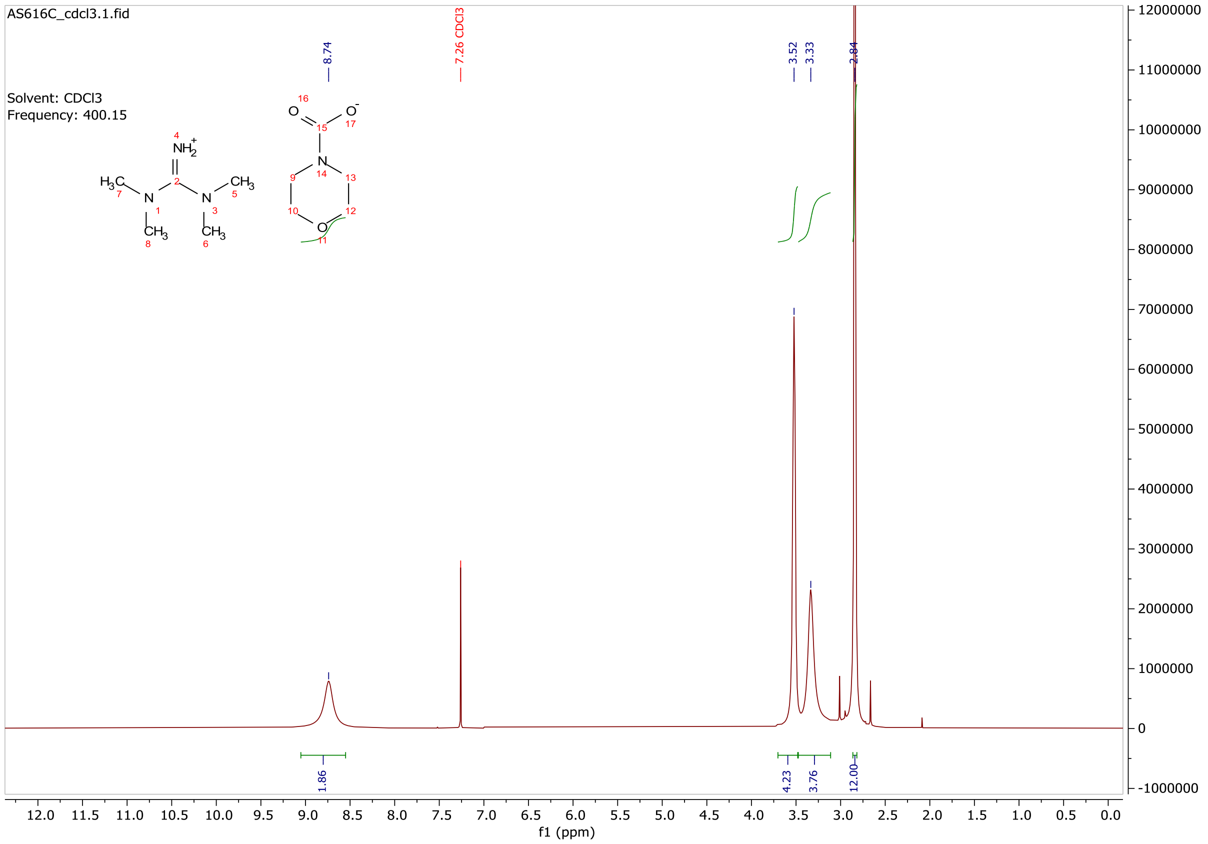

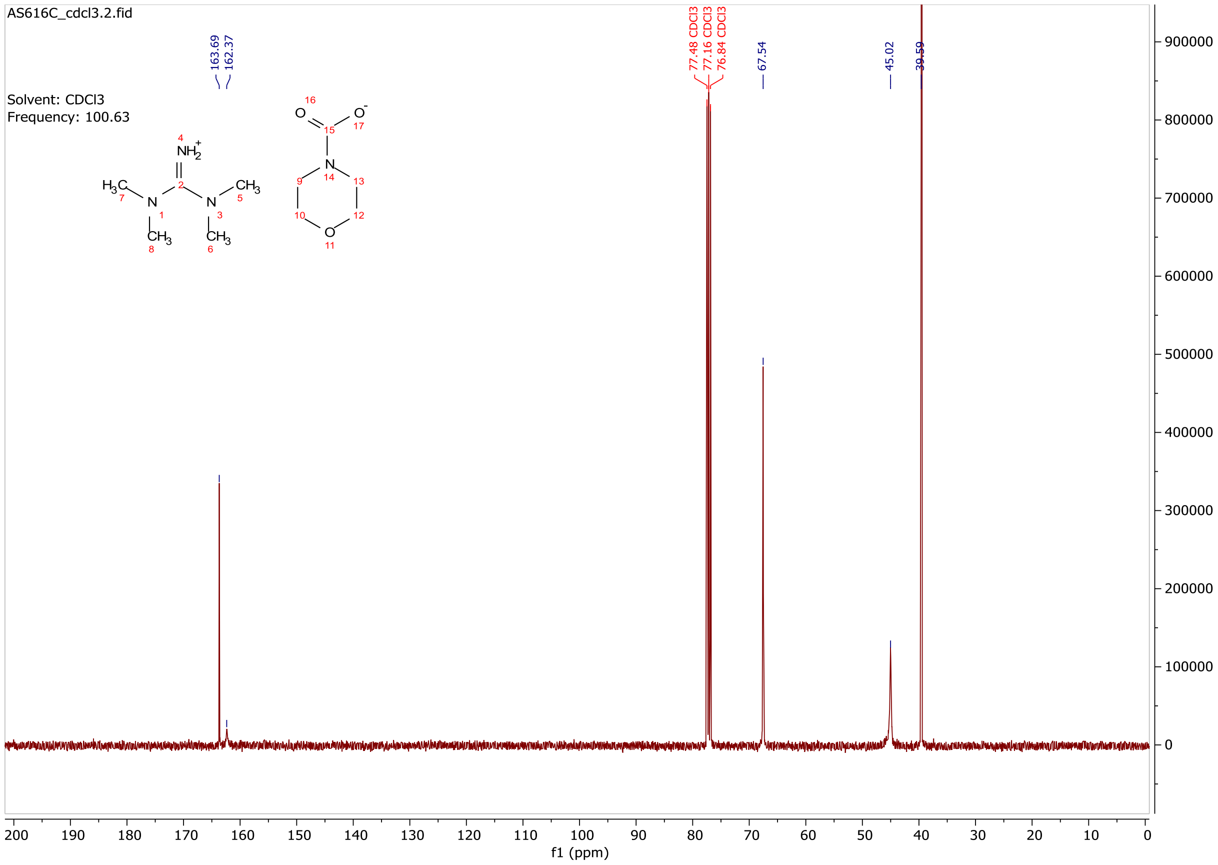

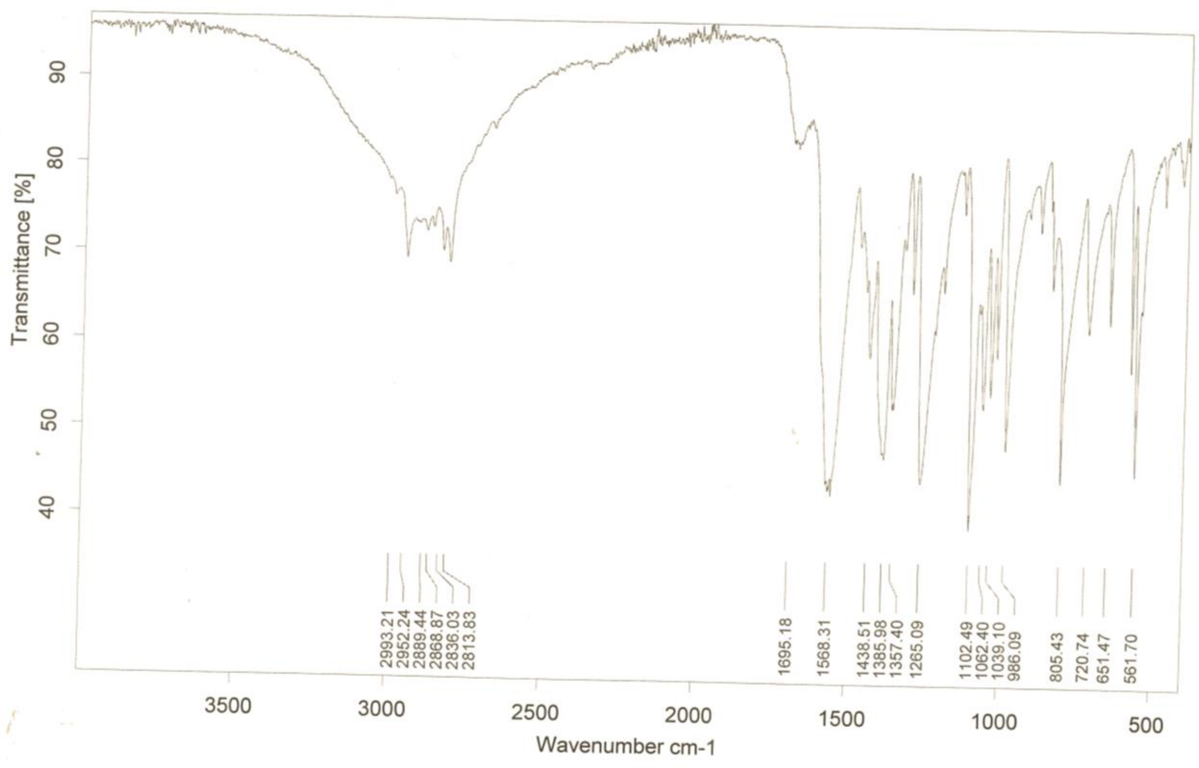

^{1}H and ^{13}C NMR spectra of o-tolyl morpholine-4-carboxylate

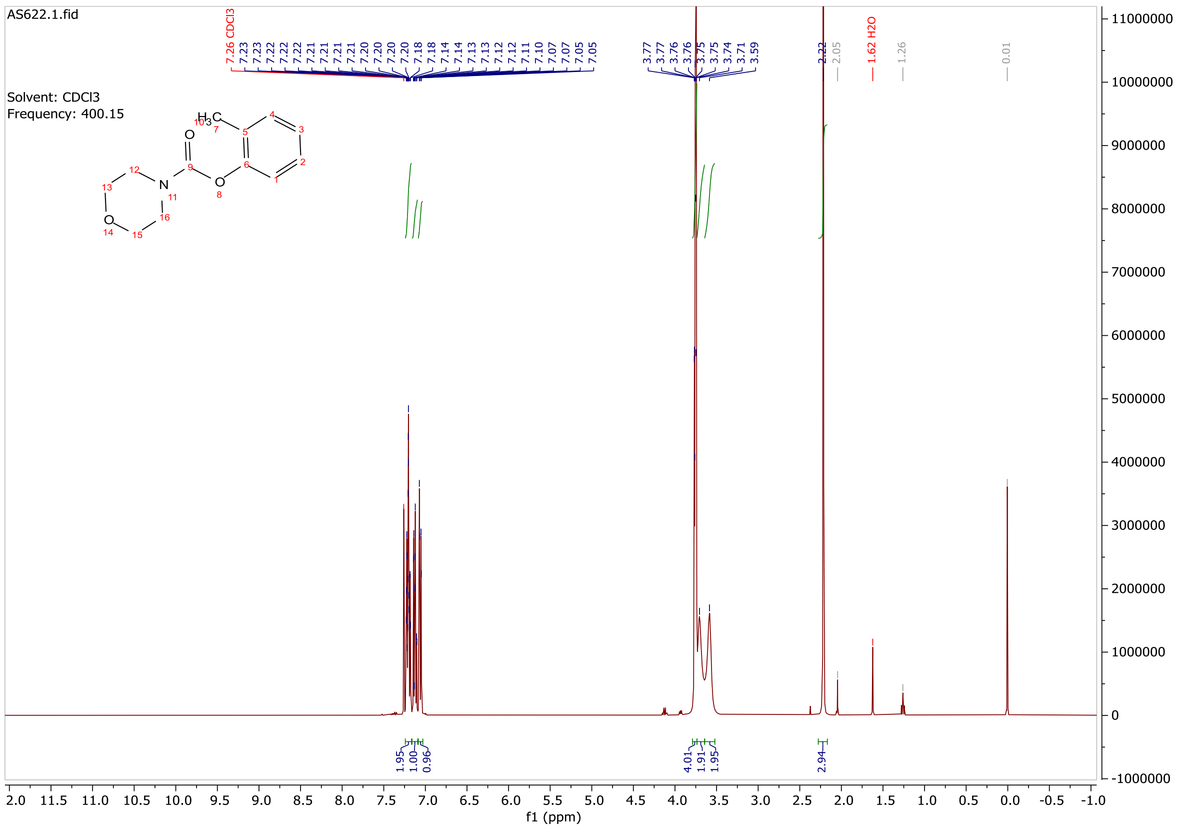

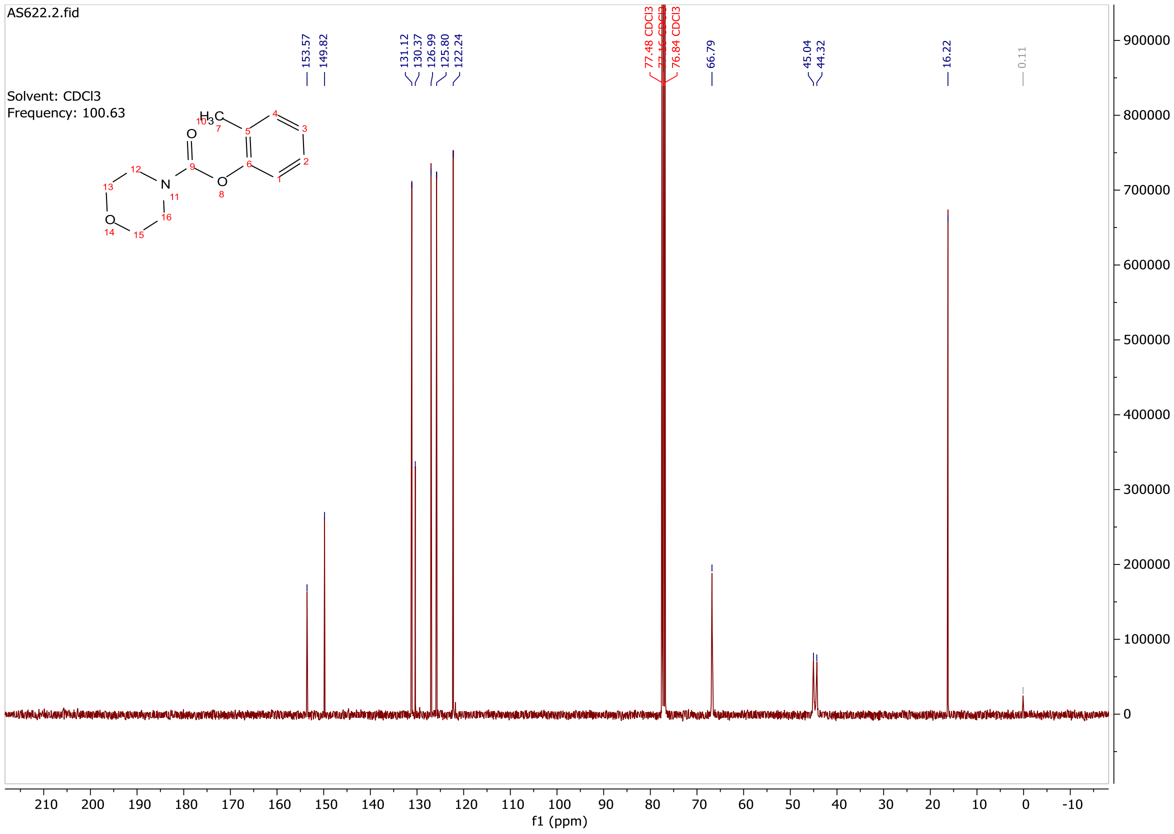
AS622.2.fid
Solvent: CDCl3
Frequency: 100.63
153.57
149.82
131.12
130.37
126.99
125.80
122.24
77.48 CDCl3
76.84 CDCl3
66.79
45.04
44.32
16.22
0.11
f1 (ppm)
